# Global Gridded Climate-Responsive Crop Selection: Sowing Dates and Crop Varieties in a Warming World

**DOI:** 10.1038/s41597-026-07164-9

**Published:** 2026-04-03

**Authors:** Sneha Chevuru, Rens L. P. H. van Beek, Michelle T. H. van Vliet, Marc F. P. Bierkens

**Affiliations:** 1 Department of Physical Geography, Utrecht University, Utrecht, The Netherlands; 2 Unit Subsurface & Groundwater Systems, Deltares, Utrecht, The Netherlands

**Keywords:** Hydrology, Climate-change impacts, Projection and prediction, Climate and Earth system modelling

## Abstract

Farmers face increasing challenges in maintaining stable crop production as climate change alters growing conditions through higher temperatures, variable rainfall, and extreme weather events. To adapt, farmers often select new crop varieties and adjust planting dates before changing crops, as these strategies involve lower costs and risks. To support assessments of future crop production under climate change, we developed a global gridded dataset that provides simulated yields and consumptive water use for multiple crop varieties and sowing dates for maize, soybean, winter wheat, spring wheat, and rice. The dataset is based on simulations with the WOrld FOod STudies crop growth model under five global climate models and three greenhouse gas concentration scenarios, at a spatial resolution of 0.5 by 0.5 degrees (~55 km at the equator), covering the years 1961 to 2100. It can help identify suitable crop varieties and planting dates that sustain yields and optimize water use. This dataset is intended for use in crop modelling, climate impact assessments, and agricultural adaptation planning.

## Background & Summary

Agriculture is one of the most vulnerable sectors to climate change, as crop production depends heavily on stable environmental conditions. However, rising temperatures, unpredictable rainfall patterns, and an increase in extreme weather events such as droughts, floods, and heatwaves are disrupting established crop production systems worldwide^1^. These climatic shifts pose a significant threat to food security, especially in regions that rely on rainfed agriculture, where even slight variations in precipitation can lead to substantial yield reductions^2^. As a result, farmers struggle to maintain stable crop production, making it harder to plan and manage risk effectively.

Beyond lowering overall yields, climate change is also altering the timing and duration of the growing season^3,4^, making crop development and productivity less predictable. While higher temperatures can accelerate plant growth, they also shorten the time available for crucial development stages, leading to reduced yields^5,6^. Prolonged exposure to high temperatures can induce heat stress, which reduces yields primarily by decreasing grain numbers (e.g., through impaired reproductive development) and by accelerating leaf senescence, thereby shortening the effective grain-filling period^7^. Conversely, cold stress^8^ reduces yield mainly through reduced stem numbers (tillering) and reduced grain numbers, while potentially delaying development and damaging sensitive growth stages such as seedlings and early reproductive phases, adding another challenge to farmers^9^.

In addition to temperature shifts, water availability changes associated with increasing water stress are becoming a pressing concern. While rainfed agriculture is the most directly affected by changing precipitation patterns, also irrigated farming is under threat. Irrigation water requirements are therefore likely to increase to sustain growth and is contributing to the depletion of water resources^10^, including rivers, lakes, and groundwater reserves, that are already affected by climate change. This depletion is further exacerbated by increasing competition for water from urban areas, industry, and energy production^11^. In many parts of the world, prolonged droughts have reduced water availability, forcing farmers to either cut back on irrigation or find alternative sources, both of which can be costly and unsustainable^12^.

Given the increasing uncertainties in climate conditions^13^, farmers must adapt their agricultural practices to maintain reliable yields. Adaptation strategies include adopting improved farming techniques, utilizing more resilient crops, and adjusting sowing dates to better align with changing climate conditions. However, these adjustments require knowledge, resources, and investments, creating barriers for many farmers, particularly smallholders with limited access to technology and capital^14^. Thus, while switching to entirely different crops may seem like a viable option, many farmers will seek to minimize the costs and risks associated with such drastic changes. Instead, they are more likely to experiment with different crop varieties (cultivars) or fine-tune their sowing or planting schedules before making significant modifications to their production systems.

As a consequence, future assessment studies projecting the vulnerability of crop systems to climate change must provide insights into the reliability of different crop varieties under present and future temperature and water constraints, i.e. by understanding how various cultivars respond to changing climate conditions and exploring the impact of changing sowing and planting dates. While previous research has primarily focused on long-term productivity or crop yield projections under different climate scenarios^15,16^, most studies have relied on a single cultivar and sowing (planting) date. There remains a significant research gap in examining how different cultivars and sowing or planting dates respond to changing climates, both in the present and the future, and how farmers could adapt in light of that uncertain information.

To address this gap, we have developed a new global gridded dataset that evaluates crop yields, biomass and consumptive water use for a large number of current cultivars and sowing (planting) dates across five major crops: maize, soybean, winter wheat, spring wheat, and rice. The developed dataset provides valuable insights to inform farmer decision-making and evaluate adaptation strategies in global earth system models. By selecting crop varieties and sowing (planting) dates that offer reliable yields and efficient water use, this research contributes to improving agricultural resilience and informing adaptation strategies to a changing climate.

## Methods

### Overview

Our newly developed dataset provides information on crop yields, crop water consumption, and crop biomass for a wide range of cultivars and sowing dates under both irrigated and rainfed conditions across different climate scenarios. Note that in the following, we will use the term “sowing” dates throughout, but depending on the crop, we may imply “planting” dates as well. The dataset covers the following crops: maize, soybean, winter wheat, spring wheat, and rice (two seasons) at a global scale, spanning the period 1961–2100 at a 0.5-degree spatial resolution. This dataset is constructed using bias-corrected output of five Global Climate Models (GCMs) and three combined Shared Socioeconomic Pathways (SSPs) – Representative Concentration Pathways (RCPs) based on CMIP6 following the protocol of the Inter-Sectoral Impact Model Intercomparison Project (ISIMIP3b) to account for varying climate scenarios (for more details on climate data see section 2.2).

**Fig. 1:**
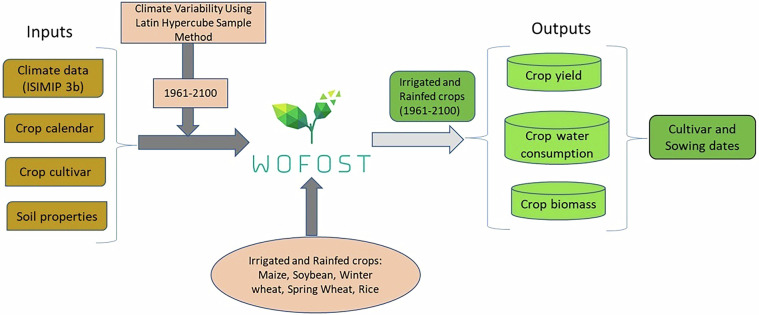
Model framework for simulating global crop yields, water consumption and biomass (1961–2100).

The crop growth simulations are based on the WOFOST (WOrld FOod STudies) crop model^17,18^, which integrates climate inputs, soil properties, crop calendars, and crop cultivars. The model framework follows a structured approach, shown below (Fig. 1), incorporating climate variability and scenario uncertainty using the Latin Hypercube Sampling (LHS) method to assess different future climatic conditions.

In our evaluation, we split the period 1961–2100 into seven 20-year consecutive time slices to enable long-term analysis of crop performance under future climate change. Combining the number of cultivars with five variations in the sowing/planting date (see below), this results in 35 different scenarios for maize and soybean, and 45 scenarios for winter wheat, spring wheat, and rice (two seasons). For each scenario, the changing climate and its uncertainty are sampled to capture variations in crop yield, crop water consumption, and crop biomass for both irrigated and rainfed conditions.

### Climate inputs

The Inter-Sectoral Impact Model Intercomparison Project phase 3b (ISIMIP3b) 10.48364/ISIMIP.842396.1 provides bias-corrected and statistically downscaled^19^ historical and future climate inputs from five climate models (GCMs): GFDL-ESM4^20^, IPSL-CM6A-LR^21^, MPI-ESM1-2-HR^22^, MRIESM2-0^23^, and UKESM1-0-LL^24^. These model experiments were derived from the latest Coupled Model Intercomparison Project (CMIP6)^25,26^. We focused on the combined RCP-SSP scenarios SSP1-RCP2.6, SSP3-RCP7.0, and SSP5-RCP8.5, each of which was simulated with bias-corrected CMIP6 climate input from these five GCMs to consider an uncertainty range of possible future conditions and uncertainties associated with the structure and parameterization of the GCMs. Please note that we follow the ISIMIP names for the climate change scenarios that include the corresponding names of the Shared Socio-economic Pathways (SSPs), but that socio-economic factors were not included in our analysis; rather, we evaluate the potential for crop production in light of future climate change.

To ensure consistency and reliability in climate projections, the data were bias-corrected using the WFDE5^27^ historical climate reference and provided at a 0.5° × 0.5° global grid resolution on a daily timescale. The dataset spans 1961–2100, with a historical reference from 1961–2014 and future projections from 2015–2100^26^.

The daily weather variables used as input for the WOFOST crop growth model include: minimum and maximum temperature, precipitation, short wave radiation, wind speed, and vapour pressure (calculated based on the variables relative humidity and minimum and maximum temperature). These variables are essential for simulating crop growth dynamics under varying climatic conditions. Instead of simulating full transient climate projections across scenarios and GCMs (15 runs 140-year runs for each combination of cultivar and sowing date combination), we divided the period of 1961–2100 in 20-year time slices and selected randomly 20 years across all GCMs and SSP-CP combinations over that period (so 20 of the 3 RCPs-SSPs x5 GCMs x20 years = 300 years) using Latin Hypercube Sampling strategy based on yearly mean temperature and yearly total precipitation (see hereafter), enabling a robust evaluation of different climatic conditions and climate variability and their impacts on crop growth while limiting the computational effort.

### Crop cultivars

The crop varieties (cultivars) used in this study are from the WOFOST crop parameter set^28^ https://github.com/ajwdewit/WOFOST_crop_parameters, a widely used resource that includes parameters for 23 different crop species. This dataset provides essential information on crop-specific attributes, including temperature requirements (temperature sums) for development stages and critical temperature thresholds, specifically the minimum, optimum and maximum temperatures for physiological processes like photosynthesis and growth. It also includes the conversion efficiency of assimilates, which refers to the portion of carbohydrates produced via photosynthesis that is converted into dry matter. This efficiency affects the total biomass production but is generally considered uniform across plant organs within WOFOST. The partitioning of assimilated biomass into different plant parts, such as leaves, stems, roots, and storage organs, is governed by partitioning coefficients that change over time, reflecting crop development stages.

For the current study, we focused on four crop species: maize, soybean, winter wheat, spring wheat, and rice, selecting all available cultivars from the WOFOST crop parameter set for each species. The selection of various cultivars enables a comparison of their respective responses to temperature, water availability and stress, and other environmental factors as a result of the genetic variations between cultivars.

Both winter wheat and spring wheat are included in this study. The cultivars used for these two types are the same, but with distinct growth requirements. Winter wheat cultivars require vernalization to flower, which is simulated by adjusting the temperature and photoperiod accordingly. Spring wheat, on the other hand, does not require vernalization and its growth stages are modelled based on thermal time accumulation. However, low temperatures can still slow phenological development and may reduce yield if cold stress occurs during sensitive stages. The distinction between winter wheat and spring wheat is vital to understanding their growth dynamics, particularly their pre-anthesis development phases.

Rice is cultivated over two distinct growing seasons, typically aligned with climatic conditions such as monsoon and dry periods. Including both seasons in the analysis allows for a more complete understanding of annual rice production and supports assessments of multi-cropping systems, which are important for food security. Therefore, in this study, one rice simulation is performed for each growing season; regions with two growing seasons therefore have two separate simulations, while regions with a single growing season have one simulation defined by the corresponding planting windows. The cultivars used in this study for each crop species are presented in Table S1 in the supplement.

### Crop calendar

To account for the need to adapt crop management under changing climatic conditions, we evaluated how shifts in sowing dates influence crop growth, yield and water use efficiency across historical and future climate scenarios. Varying sowing dates is a key adaptation strategy to mitigate climate-related stresses and optimize crop performance under altered temperature and precipitation regimes.

For this purpose, five sowing dates were selected for each grid cell to assess the variability in crop growth and yield. These sowing dates were chosen at intervals of 15 and 30 days, both earlier and later than the current sowing date. The current sowing date dataset was sourced from the Global Gridded Crop Model Intercomparison (GGCMI) phase 3 simulations^29^ 10.5281/zenodo.5062513, which provides information on global crop sowing and maturity dates for 18 different crops, separating irrigated and rainfed systems. The GGCMI dataset derives its information based on national governmental data, where available, providing the variables at each half-degree grid cell. In regions where national data is lacking, the dataset is supplemented by existing global growing season datasets. For further details on the GGCMI crop calendar dataset, including data sources and gap-filling methods, see^29^.

Each crop in this study was simulated using five different sowing dates for every grid cell, providing a robust dataset to examine how different sowing times influence crop growth and yield under climate variability. This analysis contributes to understanding the optimal selection of crop cultivars and sowing dates that optimize yields and water use efficiency in the face of climate change.

### Capturing climate variability and scenario uncertainty using Latin Hypercube Sampling

**Fig. 2:**
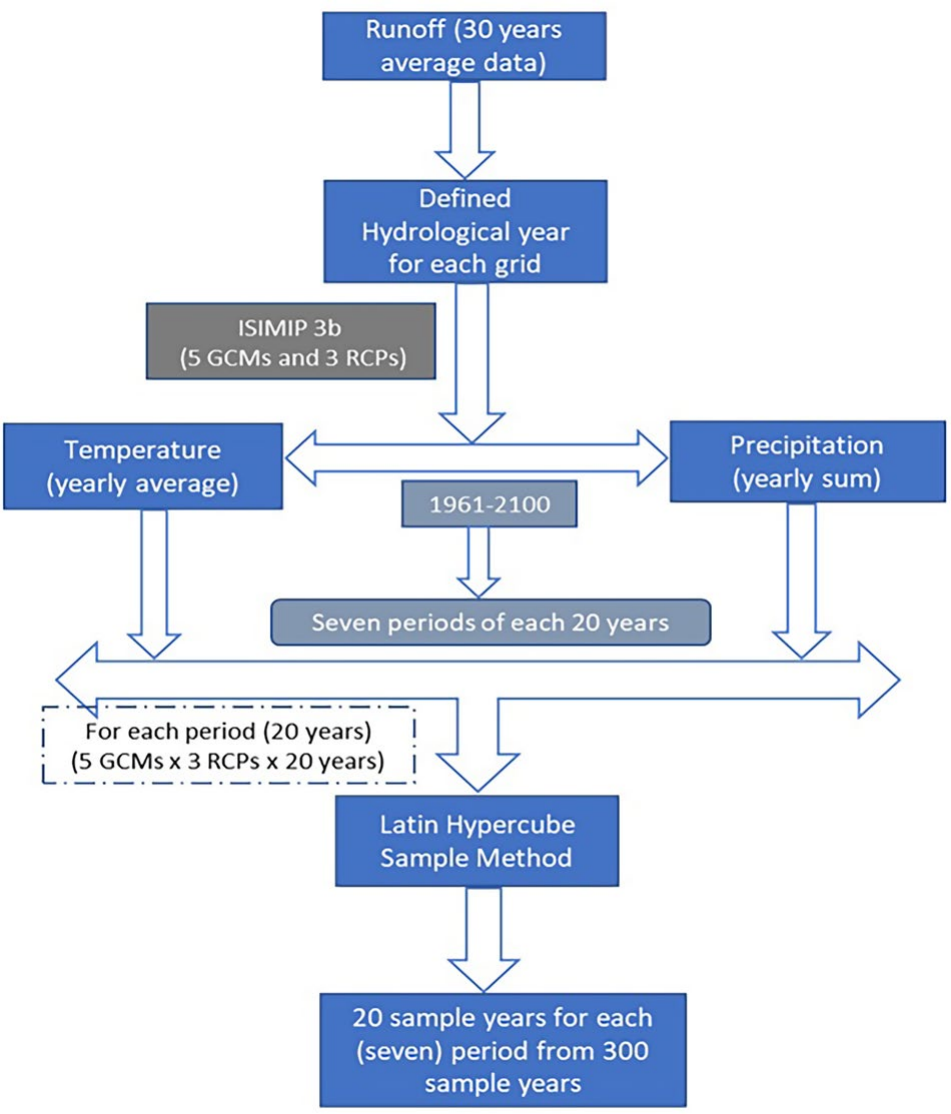
Workflow for sampling years to capture climate variability and scenario uncertainty using Latin Hypercube Sampling.

Figure 2 illustrates the workflow for capturing climatic variability and scenario uncertainty across seven 20-year periods (see Fig. S1; Supplement) using Latin Hypercube Sampling (LHS) to assess different climatic conditions. To better align with regional hydrological cycles, the start month of the hydrological year for each grid cell was determined based on the 30-year average runoff values. This approach ensures that the climate variables are seasonally relevant for agricultural and hydrological processes, which often do not follow the calendar year.

To systematically account for climate variability, we subdivided the ISIMIP3b bias-corrected climate dataset into seven 20-year periods, spanning from 1961 to 2100. The seven periods considered are: 1961–1980, 1981–2000, 2001–2020, 2021–2040, 2041–2060, 2061–2080, and 2081–2100 (see Fig. S1; Supplement). This division enables us to examine how the variability in temperature and precipitation across scenarios influences crop growth across different timeframes.

For each time slice, we calculated the average temperature and total precipitation over the hydrological year, using the data from five Global Climate Models (GCMs) and three Representative Concentration Pathways (RCPs). This combination captures a wide range of greenhouse gas (GHG) emission scenarios from low (RCP2.6) to high (RCP8.5), and reflects uncertainties in climate sensitivity across different GCMs. Although more GCMs are available, we limited our analysis to those included in the bias-corrected ISIMIP dataset to ensure internal consistency across models and variables. Additionally, computational constraints made it impractical to simulate all available GCM-RCP combinations for the set of all crops, cultivars and sowing dates.

To ensure that the selected climate data spans the full spectrum of climate variability and scenario uncertainty, while limiting the computational effort needed, we applied the Latin Hypercube Sampling (LHS) method^30^. Latin Hypercube Sampling allows for a more complete representation of the climate extremes by ensuring the inclusion of both low and high extremes, rather than relying on random sampling. The LHS method is a statistical sampling technique that ensures a comprehensive representation of the full range of variability in a dataset by evenly distributing sample points across all determining factors, precipitation and temperature in this case.

**Fig. 3:**
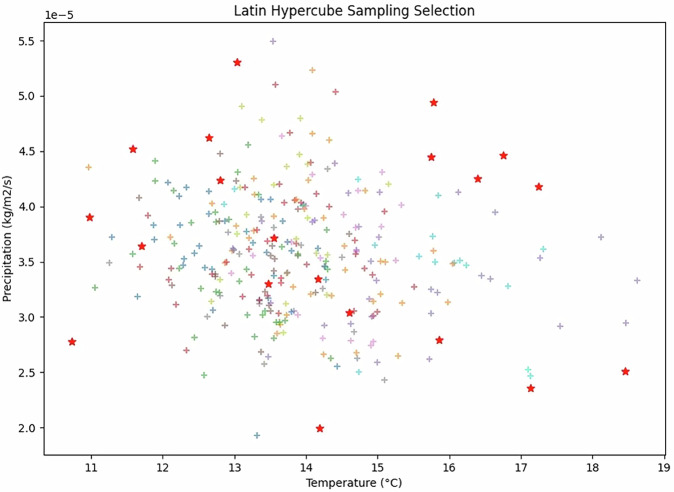
Latin Hypercube Sampling. The 20 selected years (red stars) encompass the complete range of yearly temperatures and precipitation totals per hydrological year as present in the total sample across different scenarios and GCMs (see text).

Starting from a total of 300 hydrological-year climate realizations per period (5 GCMs × 3 RCP-SSP combinations × 20 years) of temperature and precipitation, 20 representative realizations were selected by the Latin Hypercube Sampling shown in Fig. 3. Each selected realization is tied to specific GCM-scenario-year combinations; however, the sampling is performed in temperature and precipitation space rather than by replicating specific years across all GCMs or scenarios (i.e. RCP-SSP combinations). As a result, the selected samples collectively span the full range of projected temperature and precipitation conditions for a given period and can be analyzed independently of individual GCMs, scenarios, or specific calendar years.

For the two historical periods, 1961–1980 and 1981–2000, only historical climate data were available, resulting in 100 samples (5 GCMs × 1 Historical × 20 years). From these, 20 representative realizations were selected using the same LHS approach to maintain methodological consistency between historical and future periods.

This selection of 20 years from the LHS was used for the crop modelling simulations for each crop/ cultivar/ sowing date combinations. This ensures a detailed representation of how crop growth may respond to projected climate change and allows evaluation of crop performance in terms of yield and crop water requirements across a wide range of climatic conditions.

### WOFOST crop growth model

Using the specific model parameters of each cultivar, the selected climate forcing was evaluated for all crops using the WOFOST (WOrld FOod STudies) crop growth model^17,18^. WOFOST simulates crop growth and potential production under varying climatic and environmental conditions based on detailed physiological and phenological processes. As a widely used field-scale model, WOFOST typically assumes constant soil properties for its simulations. However, to ensure the accuracy and reliability of our results, we modified the model to incorporate spatially variable soil properties at each grid level. The soil properties were sourced from the FAO Digital Soil Map of the World (DSMW) database^31^ https://www.fao.org/land-water/land/land-governance/land-resources-planning-toolbox/category/details/en/c/1026564/. This modification allows for a better representation of local soil conditions, which can vary significantly across different regions and impact crop growth.

The WOFOST model was run for all 0.5 arc degree grid cells across the global land mass, except Greenland and Antarctica, regardless of the underlying land cover type, ensuring that the analysis captured a wide range of potential agricultural futures, including potential extension of crops to areas that are currently not suitable for a given crop or cultivar.

The WOFOST model operates at a daily time step and simulates key physiological processes such as photosynthesis, respiration, transpiration, and biomass accumulation. Crop phenological development is governed by temperature sums (thermal time), and the model simulates the partitioning of assimilated biomass into plant organs, including leaves, stems, roots, and grains. This partitioning is determined by the conversion efficiency of assimilates, which varies by cultivar and is a critical parameter for predicting crop yield under different climatic conditions. Thus, the model can realistically simulate how different cultivars perform under varying climatic and soil conditions.

### Dataset structure and example outputs

This section describes the dataset dimensions and presents representative examples, supported by visual summaries, to illustrate how users can access, subset, and summarize the data to derive common outputs. The dataset is organized by crop, cultivar, sowing date, irrigation regime (rainfed/irrigated), climate realization, time slice (20-year periods spanning 1961–2100), and grid cell (global gridded domain). The simulations are provided for all grid cells in the domain, independent of land cover, enabling exploration of potential future suitability and expansion of cropping areas. Users can then apply spatial filters (e.g., crop masks or harvested-area products) to restrict analyses to cultivated areas where appropriate. Details on file formats, variable definitions, and naming conventions are provided in the Data Records section. Reproducible scripts for extraction, masking, and aggregation are provided in the accompanying GitHub repository, and the full dataset is hosted on YODA.

Because the dataset is generated for all grid cells, many applications will require restricting analyses to locations where the crop is actually cultivated. To support this, crop-specific masks are provided alongside the dataset and can be applied prior to mapping, aggregation, and cross-region comparisons to focus on cultivated areas. Users may also apply harvested-area masks from external products (e.g., CROPGRIDS^32^) depending on the application. A ready-to-use workflow for applying the provided crop masks and generating crop-filtered extracts is included in the GitHub repository.

**Fig. 4:**
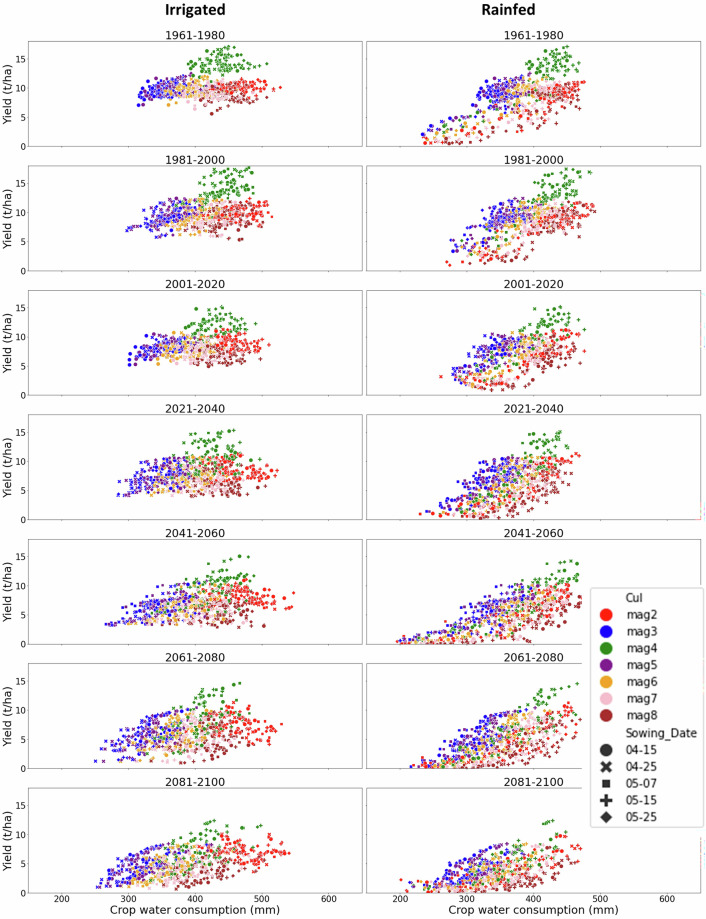
Scatter plot showing maize crop yield versus crop water consumption across seven cultivars and five sowing dates under irrigated and rainfed conditions for the seven time periods at a grid point located at 40.25° N, 91.75° W in the USA.

**Fig. 5:**
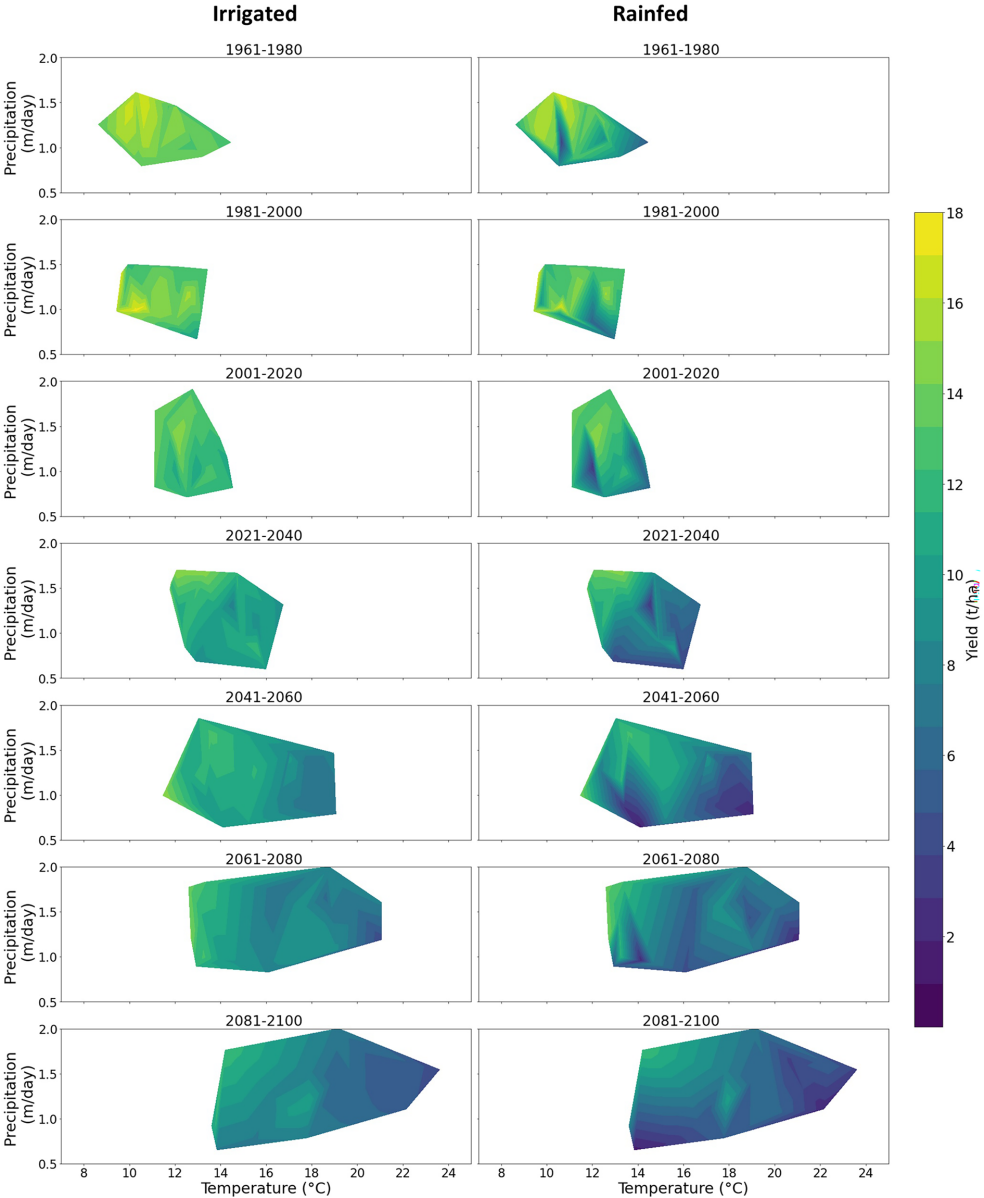
Maize crop yield for a single scenario (cultivar and sowing date) plotted against temperature and precipitation across seven time periods under irrigated and rainfed conditions. Data shown for a grid point located at 40.25° N, 91.75° W in the USA.

To illustrate the dataset dimensions, we demonstrate the internal structure of the dataset using a representative grid cell (40.25° N, 91.75° W), located in a major maize-growing region of the USA. At this initial level, we examine crop yield and crop water consumption for all combinations of cultivar and sowing date, under both irrigated and rainfed conditions, across the seven time slices. This demonstrates how variability across management options and climate conditions is represented. Relationships between yield, crop water consumption, temperature, and precipitation are shown to demonstrate the range of simulated conditions captured by the dataset (Figs. 4, 5). In this way users can identify the best combination of cultivar and sowing date which gives optimal or reliable crop yield or crop water consumption.

At the global scale, the dataset can be summarized using three commonly derived performance metrics, including productivity, reliability, and water use efficiency. Productivity is defined as the mean yield over each 20-year period for a given cultivar-sowing-date combination and represents average production potential under the prevailing climate conditions. Reliability as the minimum yield over the same 20-year period and provides a conservative measure of yield stability, capturing the risk of low-yield years under adverse climatic conditions. These two metrics therefore reflect different management objectives, as combinations that maximize productivity do not necessarily maximize reliability. Water use efficiency as the ratio of yield to crop water consumption and quantifies how efficiently a crop converts water into yield, which is particularly relevant for irrigated systems and water-limited regions. Together, these metrics allow users to evaluate trade-offs between average yield, yield stability, and water use.

**Fig. 6:**
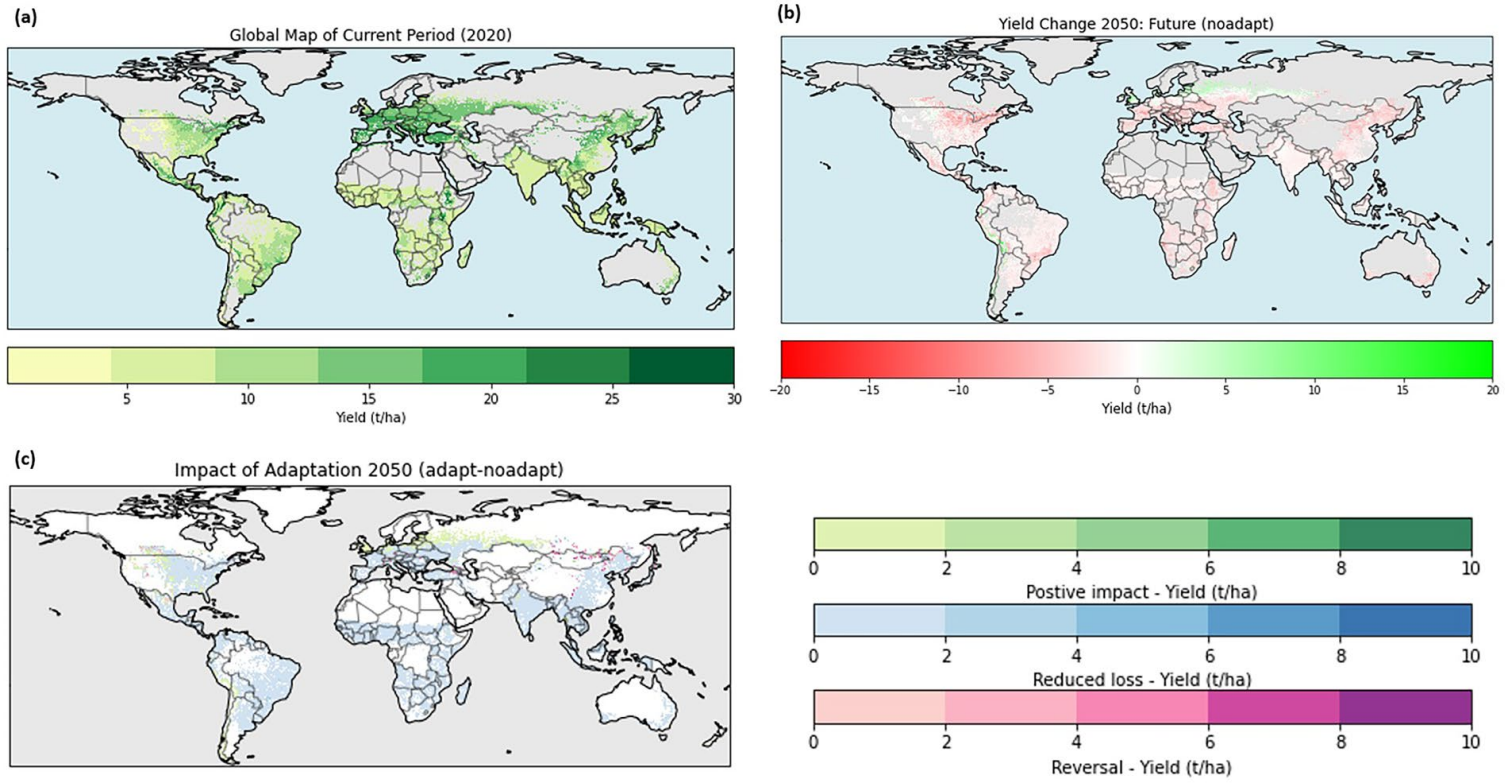
Global rainfed maize productivity: (**a**) Baseline yield in 2020, (**b**) Projected yield change by 2050 without adaptation and (**c**) Impact of adaptation on 2050 yields. Shown for crop-specific masks.

These metrics can be computed for each grid cell and time slice, allowing spatial comparisons across regions and scenarios. Figure 6a presents global rainfed maize map for the productivity metric summarized over crop-filtered areas for the historical time slice (2001–2020).

Finally, a scenario-based comparison can be derived from the dataset by contrasting management strategies across time periods. For example, in the no-adaptation case, the cultivar and sowing date selected for 2001–2020 are retained under 2041–2060 climate conditions. In the adaptation case, cultivar and sowing date are re-selected for 2041–2060 based on the chosen performance metric (e.g., productivity, reliability or water use efficiency). Differences between these strategies can be mapped to quantify potential adaptation effects across space and time. Figure 6b provides an example of projected mid-century rainfed maize yield changes under a no-adaptation using the productivity metric, while Fig. 6c illustrates how the dataset can be used to assess the impact of adaptation in the mid-century period. Additional examples for other crops and irrigation regimes are provided in the Supplementary Information.

## Data Record

The dataset is stored in Yoda repository^33^. It provides global gridded data at a 0.5 × 0.5-degree grid resolution covering 64,055 grid cells globally (excluding Greenland). The dataset includes five major crops: maize, soybean, winter wheat, spring wheat, and rice, with a separate record for two growing seasons (season1; rice1 and season2; rice2). In regions with a single cropping season, rice simulations are stored as rice1. The data spans the period from 1961 to 2100 and are aggregated into seven time slices (1961–1980, 1981–2000, 2001–2020, 2021–2040, 2041–2060, 2061–2080, 2081–2100).

### File format and naming convention

All simulation outputs are provided as tab-delimited.txt files (CSV-compatible), with one file per crop, time slice, and water management condition (irrigated and rainfed). Files follow the naming convention:

{cropname}_{period}_{condition}.txt

Where, crop name = {Maize, Soybean, Springwheat, Winterwheat, Rice1, and Rice2}, period = {one, two, three, four, five, six, and seven}corresponding to one: 1961–1980, two: 1981–2000, three: 2001–2020, four: 2021–2040, five: 2041–2060, six: 2061–2080, and seven: 2081–2100, condition = {irr, rf}corresponding to irr: irrigated, and rf: rainfed.

### Data content and variables

Each csv file contains gridded annual simulation outputs generated using the WOFOST crop model driven by multiple Global Climate Models (GCMs) and greenhouse gas concentration scenarios (RCP-SSP combinations).

**Fig. 7:**
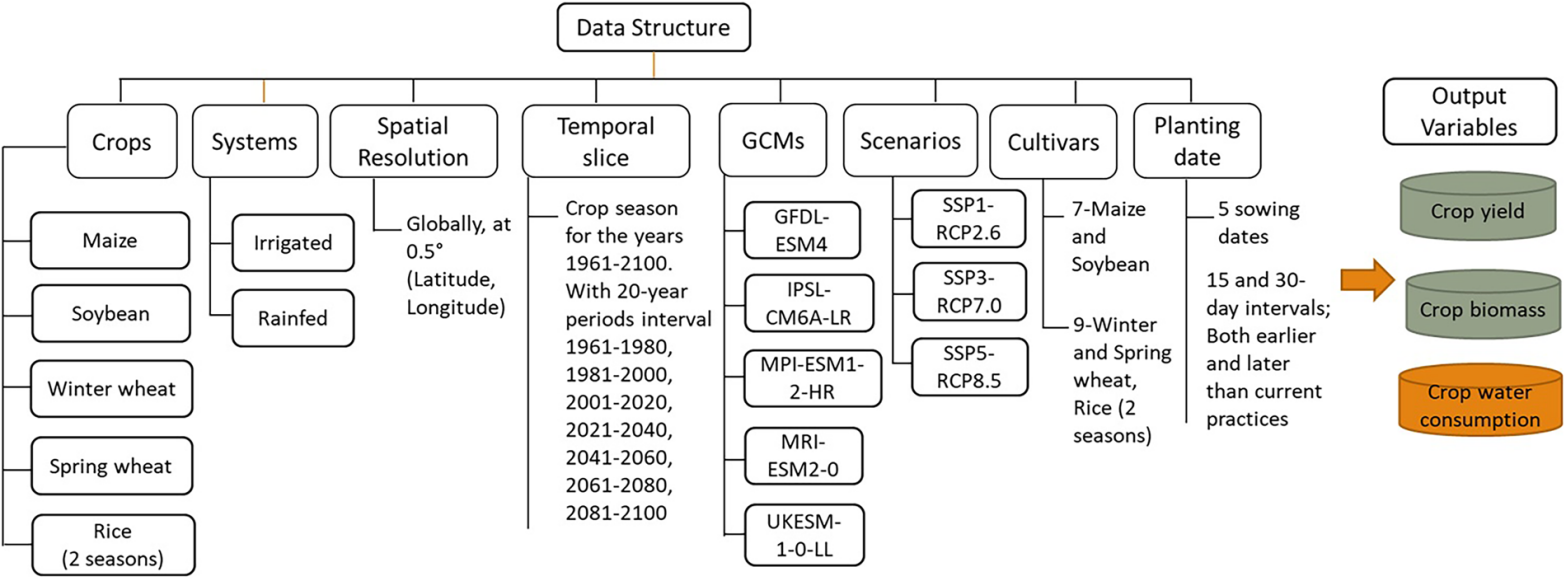
Data structure of simulated crop outputs across different scenarios (based on the Shared Socioeconomic Pathways (SSPs) and Representative Concentration Pathways (RCPs) for five different Global Climate Models (GCMs)), considering different cultivars and sowing dates.

Each row represents one grid cell latitude (Lat), longitude (Lon), year, climate model (GCM), scenario (RCP), cultivar, and sowing date combination, yield, crop water consumption (CWR), and biomass. The columns included in every file are listed in Table 1. An overview of the dataset structure is shown in Fig. 7.

**Table 1:** Documentation of variables.

Variable	Description
Lat	Latitude of the grid cell (decimal degrees)
Lon	Longitude of the grid cell (decimal degrees)
Cultivar	Crop variety used; corresponds to variety definitions.
Sowing	Sowing/planting date (MM-DD format)
GCM	Global Climate Model used for the simulation
RCP	Climate scenario: RCP-SSP combination (e.g., ssp370, ssp585)
Year	Simulation year
Yield	Crop yield (kg/ha)
CWR	Crop water requirement or consumption (mm)
Biomass	Total aboveground crop biomass (kg/ha)

### Repository structure and contents

The repository is organized into the following directories:

- **data/**

Contains two compressed folders:

- Irrigated.zip — crop simulation outputs under irrigated conditions
- Rainfed.zip — crop simulation outputs under rainfed conditions

Each zip archive includes crop-specific text files for all seven time periods.

- **data/crop_variety/**
  Contains crop-specific files documenting the cultivar (variety) information used in the simulations. Files listing the cultivars used for each crop are included.
- **crop_masks/**
  Contains crop-specific mask files describing harvested areas, which are used to define the spatial extent of each crop.
- **Hy_month/**
  Contains a global NetCDF file providing the start month of the hydrological year for each grid cell.
- **LHS/**

Contains the Latin Hypercube Sampling data for each grid cell per time slice in a zip folder. These include information on representative sample years, respective GCM, RCP-SSP scenario and corresponding precipitation and temperature data.

- **README.md**

Provides an overview of the dataset, repository structure, and instructions for accessing and processing the data.

## Technical Validation

The dataset released here includes simulations for all available WOFOST cultivars and five sowing-date variants per grid cell for both historical and future periods. Given the global scope and the size of the simulation ensemble, it is not feasible and not the aim of a Data Descriptor to validate every cultivar-sowing-date combination against observations globally. Instead, our technical validation focuses on two aspects that are most relevant for data quality: (i) the credibility of the underlying WOFOST model and crop-parameter set, and (ii) whether WOFOST can reproduce the observed magnitude and spatiotemporal variability of yields under a consistent set of assumptions in a well-observed region.

In terms of model credibility, WOFOST has been widely applied for global and regional studies to simulate crop production under varying management and climate conditions. Over Europe, WOFOST was used to assess crop production under both present^34,35^ and projected climate conditions^35–37^. For Africa and China, WOFOST was used for land evaluation, assessment and crop yield forecasting^35,38–42^. Numerous studies have validated WOFOST’s simulation results against experimental or observed yield data^37,42,43^, while additional model comparison exercises have also confirmed its performance^44–46^.

All cultivars used in this study have been fine-tuned against field trial data^47^, and are consolidated in the WOFOST crop parameter set^28^. This parametrization provides a consistent, trial-calibrated cultivar library for simulating crop growth and production across diverse environments, while not implying local calibration for every grid cell worldwide.

**Fig. 8:**
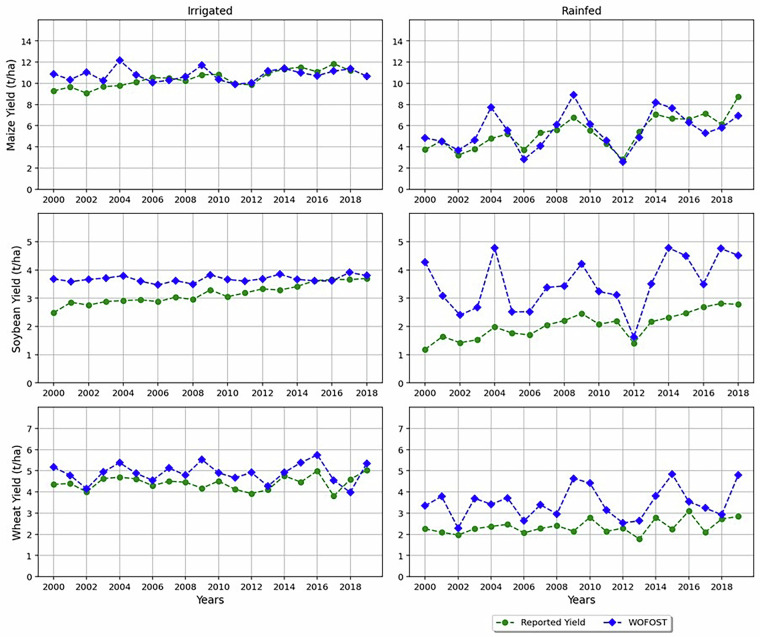
Temporal analysis of irrigated and rainfed crops of maize, soybean and wheat for the years 2000 to 2019 of the CONUS region. Reported yields are derived from county-level data obtained from the USDA National Agricultural Statistics Service (USDA-NASS). For each crop, we calculate a weighted average yield per year, where weights are based on the harvested area in each county. These annual weighted averages are then aggregated across all states within the CONUS.

**Fig. 9:**
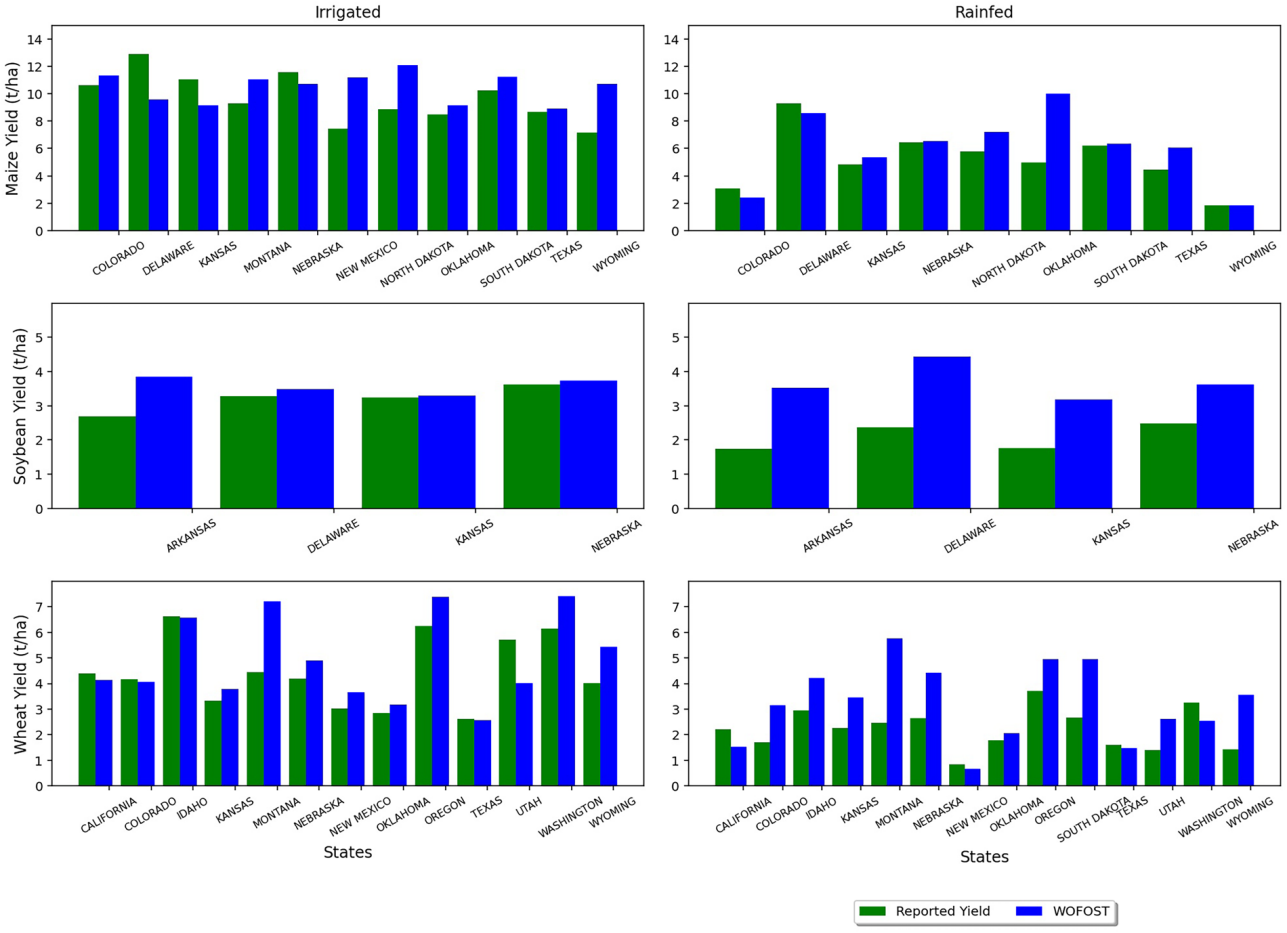
Spatial (i.e., state level) analysis of irrigated and rainfed crops of maize, soybean and wheat for the years 2000 to 2019 for the CONUS region.

As an observational based consistency check relevant to the present dataset, Figs. 8, 9 summarize spatial and temporal comparisons from the present author’s earlier work^48^, in which WOFOST was applied over the Contiguous United States (CONUS) for the period 2000–2019 and was evaluated against United States Department of Agriculture (USDA)^49^ National Agricultural Statistics Service reported yields for maize, soybean, and wheat. A comparison was performed using a single cultivar parameterization per crop (selected as the best-matching one for CONUS based on agreement with reported yields). Our validation results therefore show the overall model behaviour and parameter sensitivity at regional scale, rather than individually validating each cultivar and each sowing-date variant included in the present dataset. We use this evidence to support that the modelling system underlying the dataset produces plausible yields estimate and yield variability. Thus, the ensemble of cultivars and sowing-date variants in the released dataset should be interpreted as a structured exploration of cultivar choice and planting-date uncertainty and as well as potential adaptation space under changing climate conditions.

### Reflection on potential applications

Beyond its primary use in assessing climate impacts on crop yields and adaptation strategies, the newly developed dataset holds potential for a wide range of additional applications in agricultural, environmental, and socio-economic research. First, it allows for including local adaptation by farmers when assessing the impacts of climate change on crop yield and crop water use. Second, it can be used to explore water-use efficiency trends under climate change across time and regions. By analysing the relationship between yield and crop water consumption, researchers can identify areas where improvements in irrigation efficiency or drought-tolerant cultivars have the greatest potential to conserve water while maintaining or improving productivity Third, the data can support agricultural risk assessment and insurance modelling by offering a long-term view of yield variability under different climate scenarios and management practices, considering different future periods. This can aid in designing more robust financial safety nets for farmers at a large scale, facing increasingly uncertain growing conditions. Fourth, the dataset can inform land use planning, food security assessments, and economic modelling. For instance, linking crop yield outcomes with population projections and trade models can support the modelling of future supply-demand dynamics and identify potential food-insecure regions. Finally, the dataset may be used as a valuable resource for training, scenario analysis, and machine learning applications. A caveat of ML applications is that the dataset is entirely based on the WOFOST model rather than on observed crop yields and therefore reflects WOFOST’s assumptions. However, its large spatial and temporal coverage, combined with consistent formatting, makes it suitable for developing predictive models and testing algorithm performance in agricultural contexts, provided historic information on yields, cultivars etc., is ingested to constrain such models.

### Uncertainties

The presented dataset offers an extensive and valuable resource for understanding crop performance and crop water consumption across a wide range of climatic conditions. Still, it is important to recognize the inherent uncertainties and limitations that influence its interpretation and application.

Although the underlying simulations are based on the process-based WOFOST model and include key biophysical processes (e.g., soil water balance, crop development through crop calendars, and atmospheric demand effects), the current dataset presented provides precomputed outputs rather than a model interface that users can run and modify. This means that, using the dataset standalone, users cannot change individual factors such as soil water-holding characteristics, humidity, wind, or extreme-event conditions beyond what is already included in the simulation design. The dataset should therefore not be seen as a replacement for process-based crop models rather as a complement when mechanistic attribution or targeted sensitivity analyses are required. Instead, it is intended for large-scale comparison studies and assessments of yield and crop water consumption across cultivars, sowing-date options, climate scenarios, and water-management assumptions. Promising cultivar-sowing-date combinations identified from these comparisons can then be tested in follow-up process-based simulations where additional parameters are changed explicitly.

One major source of uncertainty stems from the use of a single crop simulation model. Although the WOFOST crop growth model has been calibrated and validated against observed data, relying on one model may limit the robustness of the projections. Multi-model ensembles, as used in other large-scale agricultural assessments such as initiatives within ISIMIP and AgMIP^50^, can better capture the range of possible outcomes by accounting for differences in the structure and parameterization among different models. Incorporating a multi-model approach in future work would enhance certainty in the findings and better reflect the variability in crop growth responses to climate change. While the validity of our results is dependent on WOFOST, our approach can be easily expanded and evaluated by other crop models.

Beyond the model structure, other limitations arise from the exclusion of certain critical agricultural inputs and socio-economic factors. The current dataset focuses primarily on water availability (irrigated and rainfed) without explicitly accounting for fertilizer availability, labour constraints, or energy inputs, all of which significantly influence crop productivity. These factors can vary widely across regions and are subject to both market dynamics and policy interventions, making them important components for future dataset enhancements.

Technological advancements, particularly the development and adoption of new cultivars, also play a pivotal role in shaping future agricultural outcomes. While the dataset includes a variety of existing cultivars and sowing dates, these may not fully represent locally adapted varieties or optimal management practices across diverse agro-climatic regions. In particular, the use of five prescribed sowing dates does not capture dynamic adjustments that farmers may adopt under future climate conditions. Moreover, the dataset does not account for the potential impact of future innovations such as drought-resistant or heat-tolerant crop varieties, precision agriculture technologies, or improved soil management practices. Integrating these potential technological trajectories could provide a more forward-looking assessment of adaptation capacity and yield potential.

Moreover, the dataset operates under the assumption of fixed management practices over time, which may not reflect the dynamics of real-world farming systems. In reality, farmers are likely to adjust their strategies in response to both climate signals and policy incentives. Accounting for such adaptive behaviour through agent-based modelling or socio-economic scenario integration would enhance the accuracy and policy relevance of the projections. Our presented dataset should be interpreted as exploring relative performance across a library of cultivar parameter sets, rather than directly prescribing locally adapted cultivar recommendations for farmers in specific region.

Despite these limitations, the dataset can be a valuable resource for long-term agricultural planning. It provides critical insights into spatial and temporal variations in crop performance and crop water consumption that offer a strong basis for exploring adaptation strategies. Continued refinement and expansion of the dataset, especially through multi-model integration and inclusion of socio-economic and technological dimensions, will be essential for improving its utility in addressing global food security challenges under climate change.

## Supplementary information

Supplementary Information

## Data Availability

The global dataset used in this study is available for 64,055 (excluding Greenland) grid cells at a 0.5 × 0.5-degree spatial resolution for each of the six crops (maize, soybean, winter wheat, spring wheat, rice1, and rice2) over the 1961–2100 period. The data are provided in a TXT format and can be accessed via the landing page: https://public.yoda.uu.nl/geo/UU01/8V0A4N.html or through the 10.24416/UU01-WUCN2F^33^. A detailed description of the dataset, including structure, variables and usage guidelines, is also available at the DOI link. All external datasets used as inputs to generate the results of this study are publicly accessible and were obtained from established repositories or official institutional sources. Climate forcing data were sourced from the Inter-Sectoral Impact Model Intercomparison Project (ISIMIP) database and are available via the 10.48364/ISIMIP.842396.1. Crop calendar information was obtained from the AgMIP-GGCMI crop calendars repository 10.5281/zenodo.5062513. Crop cultivar parameters were sourced from the WOFOST crop parameter repository https://github.com/ajwdewit/WOFOST_crop_parameters. Soil properties were derived from the FAO Digital Soil Map of the World (DSMW), available through the Food and Agriculture Organization of the United Nations https://www.fao.org/land-water/land/land-governance/land-resources-planning-toolbox/category/details/en/c/1026564/. All external datasets are openly available for research use. Reuse, redistribution, and potential commercial use are subject to the specific licensing terms of each data provider, and users are advised to consult the original repositories for detailed license conditions and attribution requirements.

## Code availability

An Python executable scripts used to process the data, test, and validate yield outputs, and filter the dataset using crop specific masks (e.g., restricting analyses to currently cultivated areas or regions of interest based on latitude and longitude selection) are available in the GitHub repository https://github.com/SnehaChevuru/Climate_Responsive_Crop_Selection_Global_Dataset.
