# Supplementary material for: Global Gridded Climate-Responsive Crop Selection: Sowing Dates and Crop Varieties in a Warming World
Source: Sci Data. 2026 Apr 3;13:795. doi: 10.1038/s41597-026-07164-9 (PMC13219509; doi:10.1038/s41597-026-07164-9)
Supplement: Supplementary file 1 — Supplementary Information [file 41597_2026_7164_MOESM1_ESM.pdf]

Global Gridded Climate-Responsive Crop Selection: Sowing Dates and Crop Varieties in a Warming World

Sneha Chevuru[1], L.P.H. (Rens) van Beek[1], Michelle T.H. van Vliet[1], Marc F.P. Bierkens[1&2]

1 Department of Physical Geography, Utrecht University, The Netherlands.
2 Unit Subsurface & Groundwater Systems, Deltares, Utrecht, The Netherlands.

S1. Cultivar information

Table S1. Number of cultivars for each crop used in the study

S.No	List of crops	Number of cultivars listed in the study
1	Maize	7
2	Soybean	7
3	Winter Wheat	9
4	Spring Wheat	9
5	Rice 1	9
6	Rice 2	9

S2. Time frame

The following seven time periods, spanning from 1961-2100, were selected to analyze the variability of temperature and precipitation and their impact on crop growth.

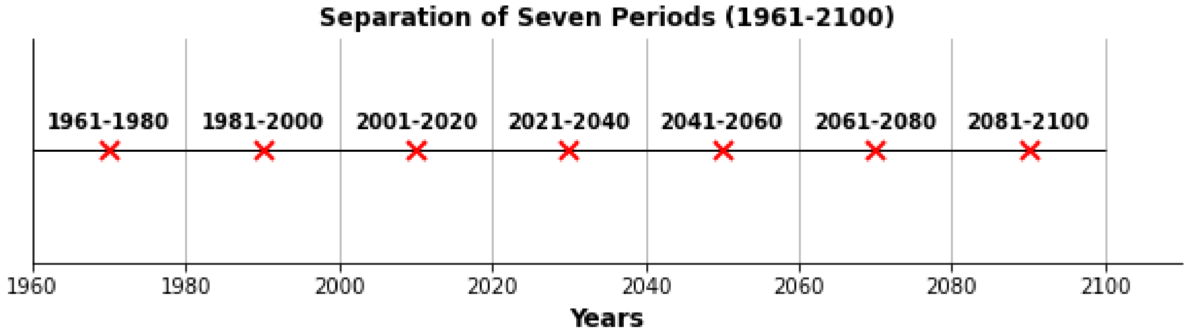

Figure S1: Separation of seven periods (1961-2100)

S3. Scatter plots

We selected the same representative grid cell at 40.25° N, 91.75° W for maize and soybean (see section 2.7), but used a different cell at 40.25° N, 88.75° W for winter wheat, spring wheat, and rice (two seasons). This is because the representative grid cell chosen for maize is not a major production area for the other crops. To ensure a consistent and representative analysis, we chose a second location where all the other crops are significantly present.

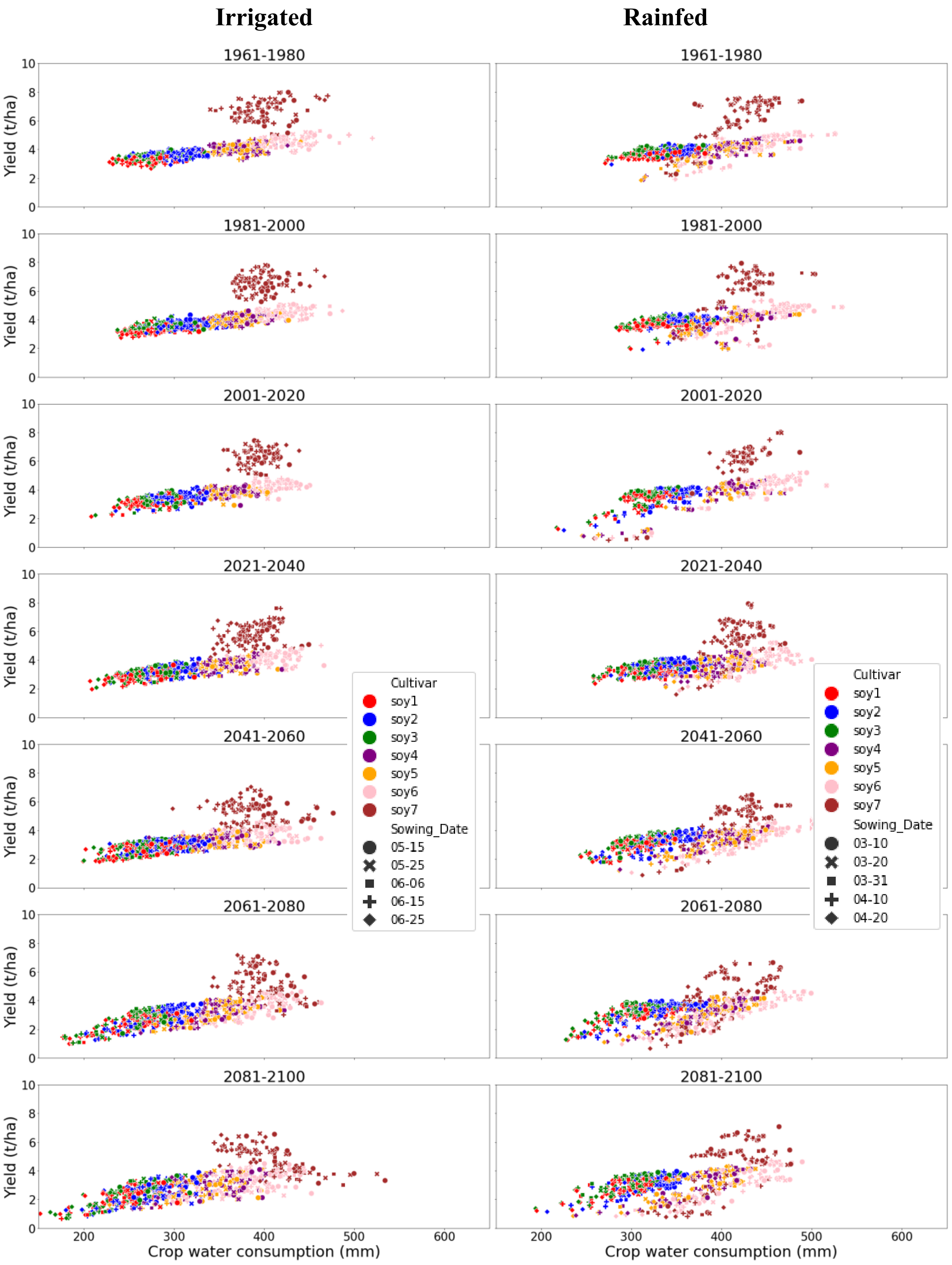

Figure S2: Scatter plot showing soybean crop yield versus crop water consumption across seven cultivars and five sowing dates under irrigated and rainfed conditions for the seven time periods at a grid point located at 40.25° N, 91.75° W in the USA.

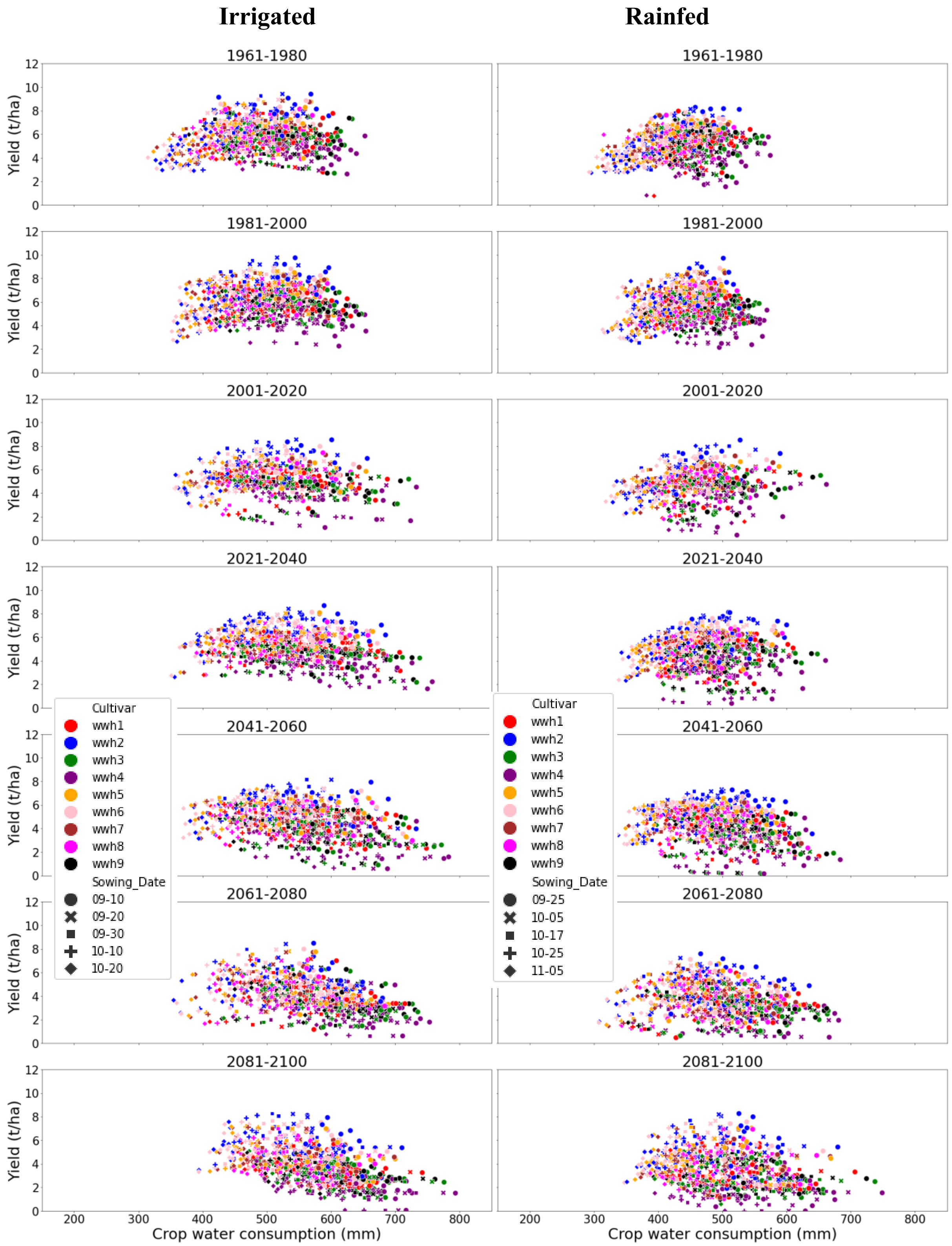

Figure S3: Scatter plot showing winter wheat crop yield versus crop water consumption across seven cultivars and five sowing dates under irrigated and rainfed conditions for the seven time periods at a grid point located at 40.25° N, 88.75° W in the USA.

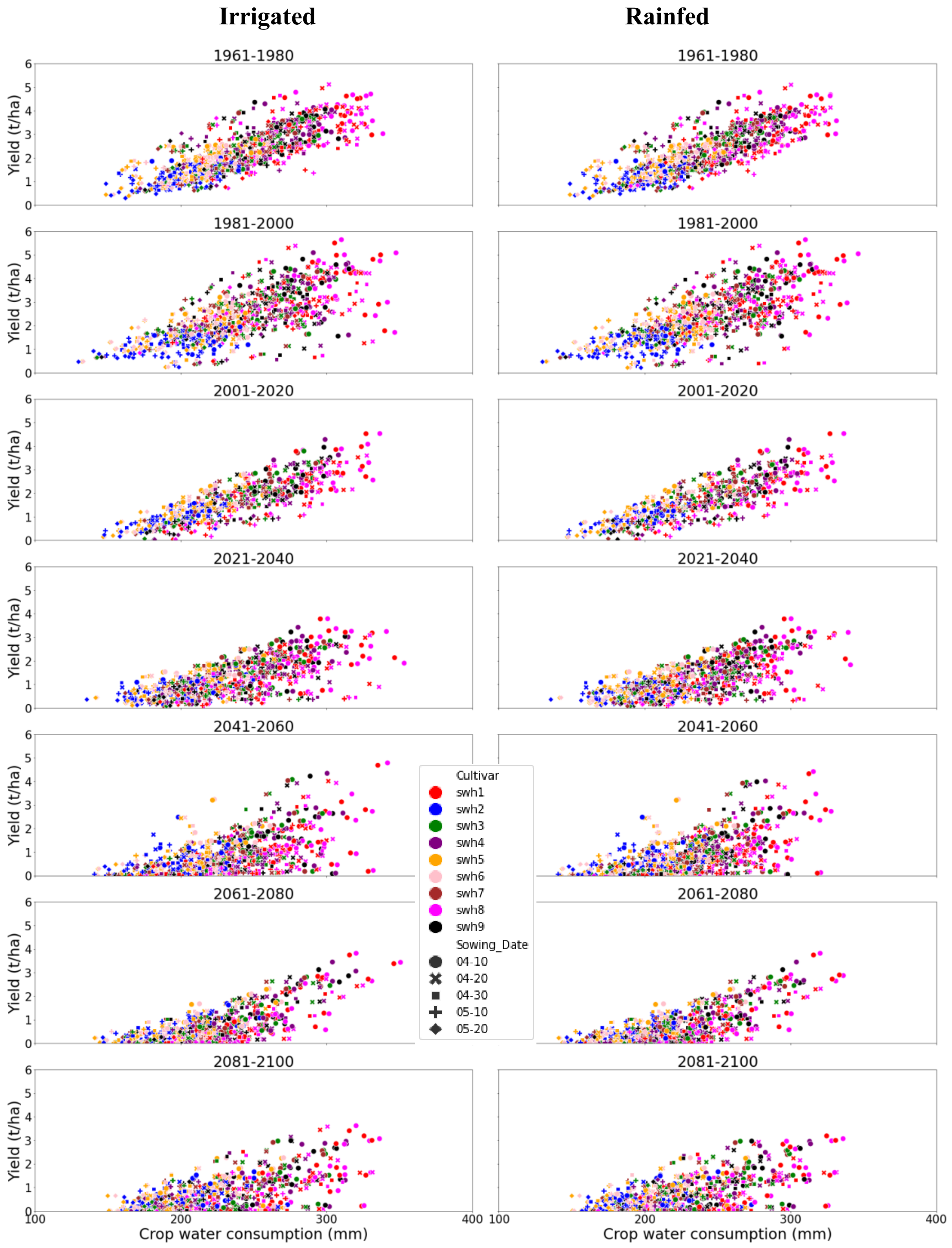

Figure S4: Scatter plot showing spring wheat crop yield versus crop water consumption across seven cultivars and five sowing dates under irrigated and rainfed conditions for the seven time periods at a grid point located at 40.25° N, 88.75° W in the USA.

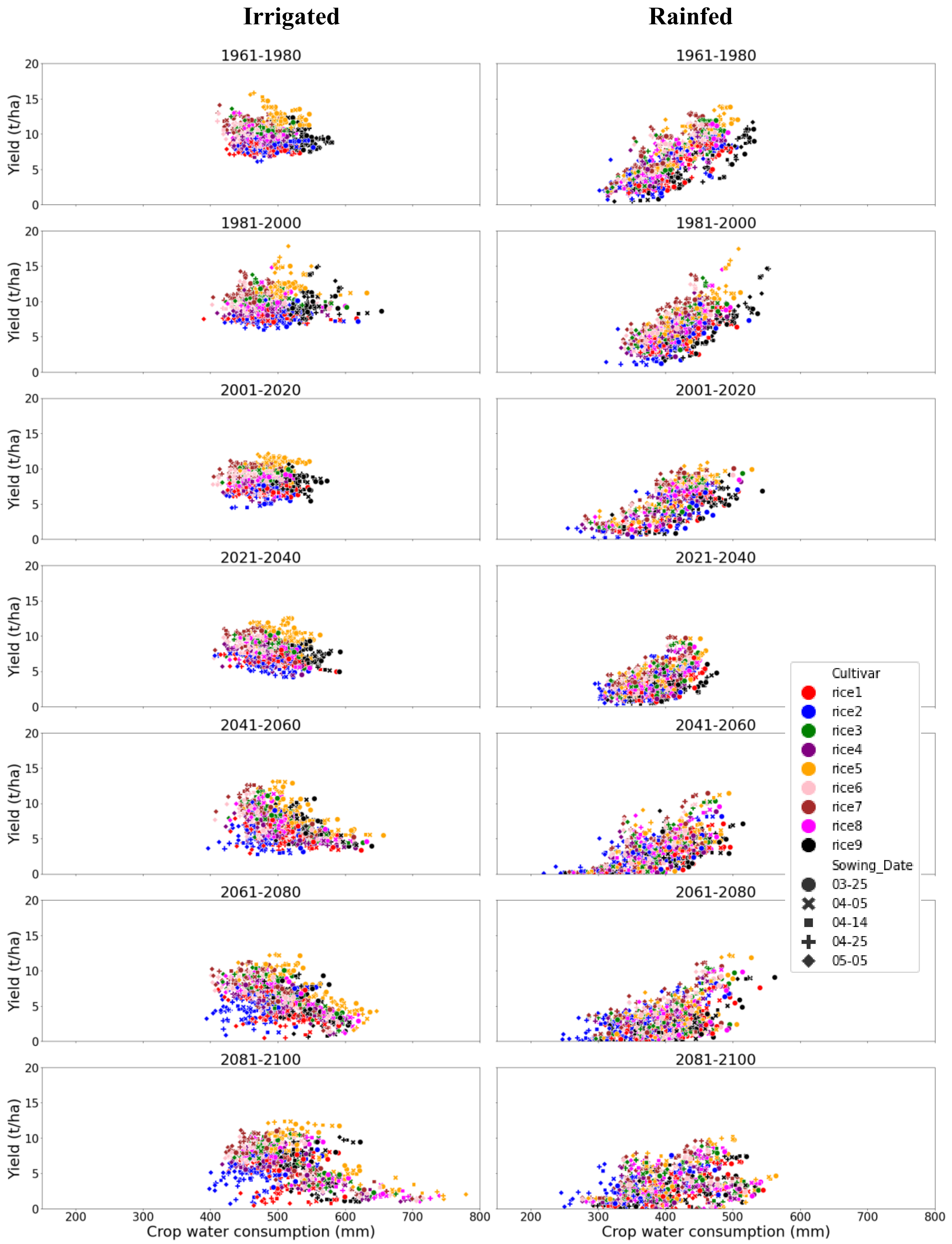

Figure S5: Scatter plot showing rice season-1 crop yield versus crop water consumption across seven cultivars and five sowing dates under irrigated and rainfed conditions for the seven time periods at a grid point located at 40.25° N, 88.75° W in the USA.

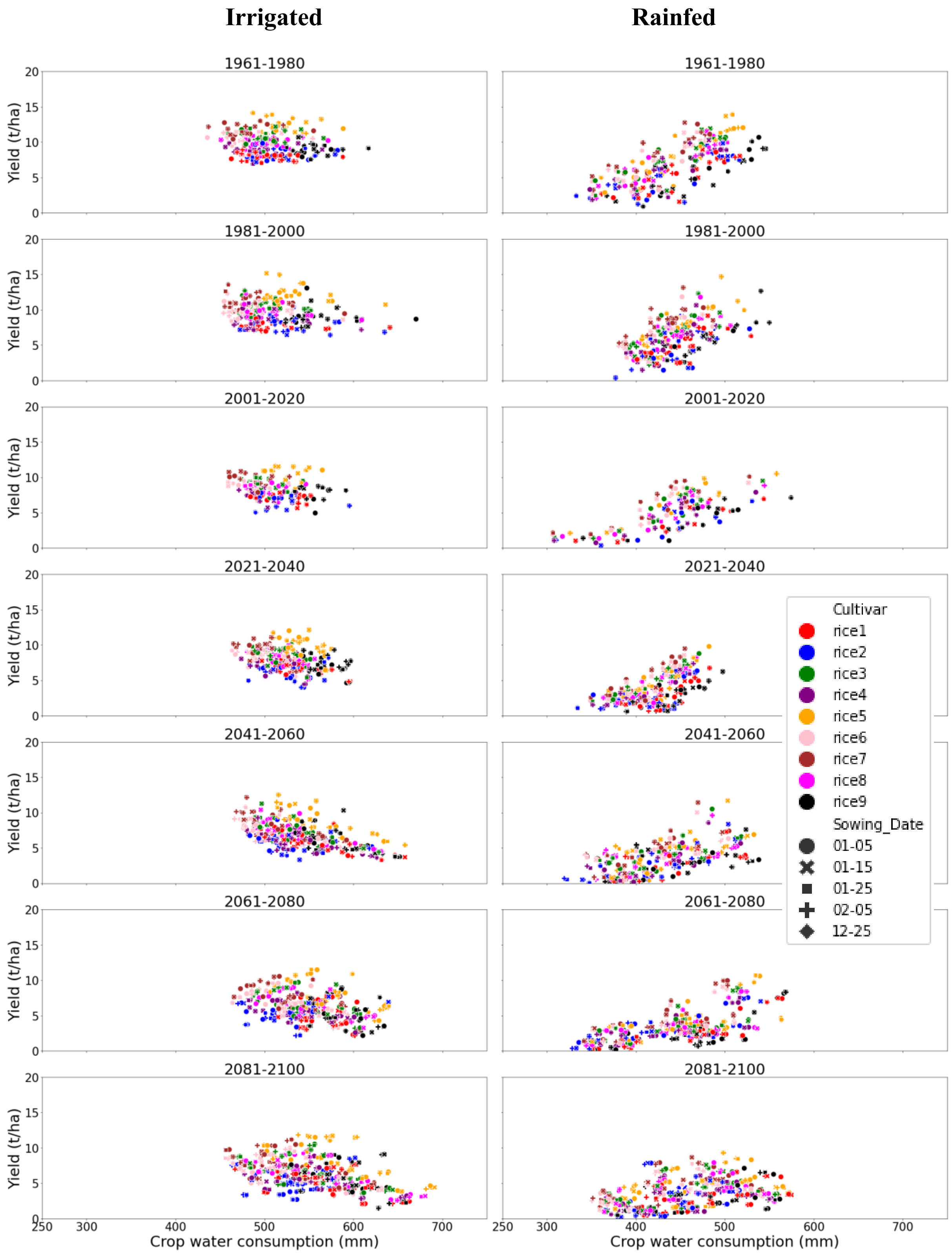

Figure S6: Scatter plot showing rice season-2 crop yield versus crop water consumption across seven cultivars and five sowing dates under irrigated and rainfed conditions for the seven time periods at a grid point located at 40.25° N, 88.75° W in the USA.

S4. Contour plots

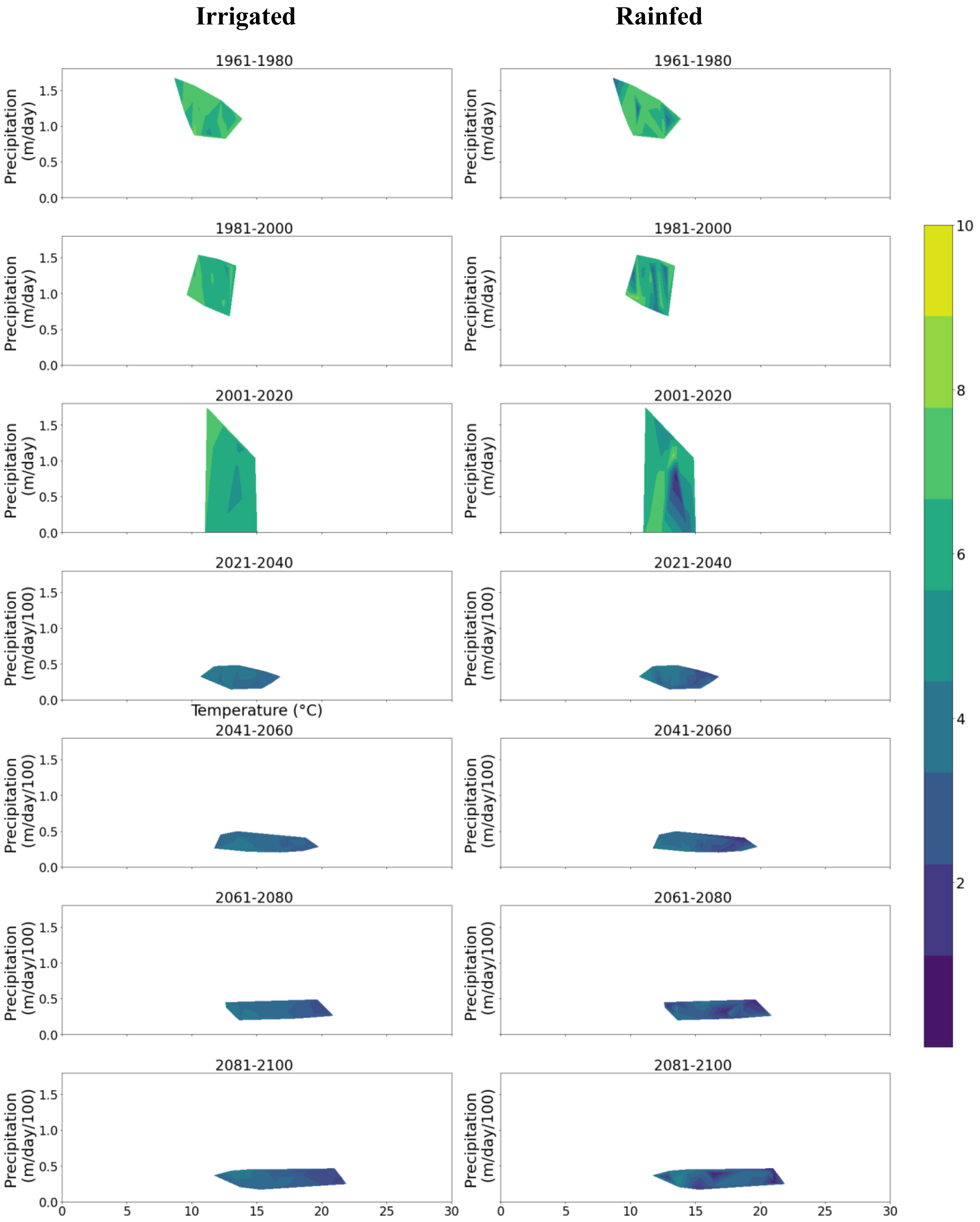

Figure S7: Soybean crop yield for a single scenario (cultivar and sowing date) plotted against temperature and precipitation across seven time periods under irrigated and rainfed conditions. Data shown for a grid point located at 40.25° N, 91.75° W in the USA.

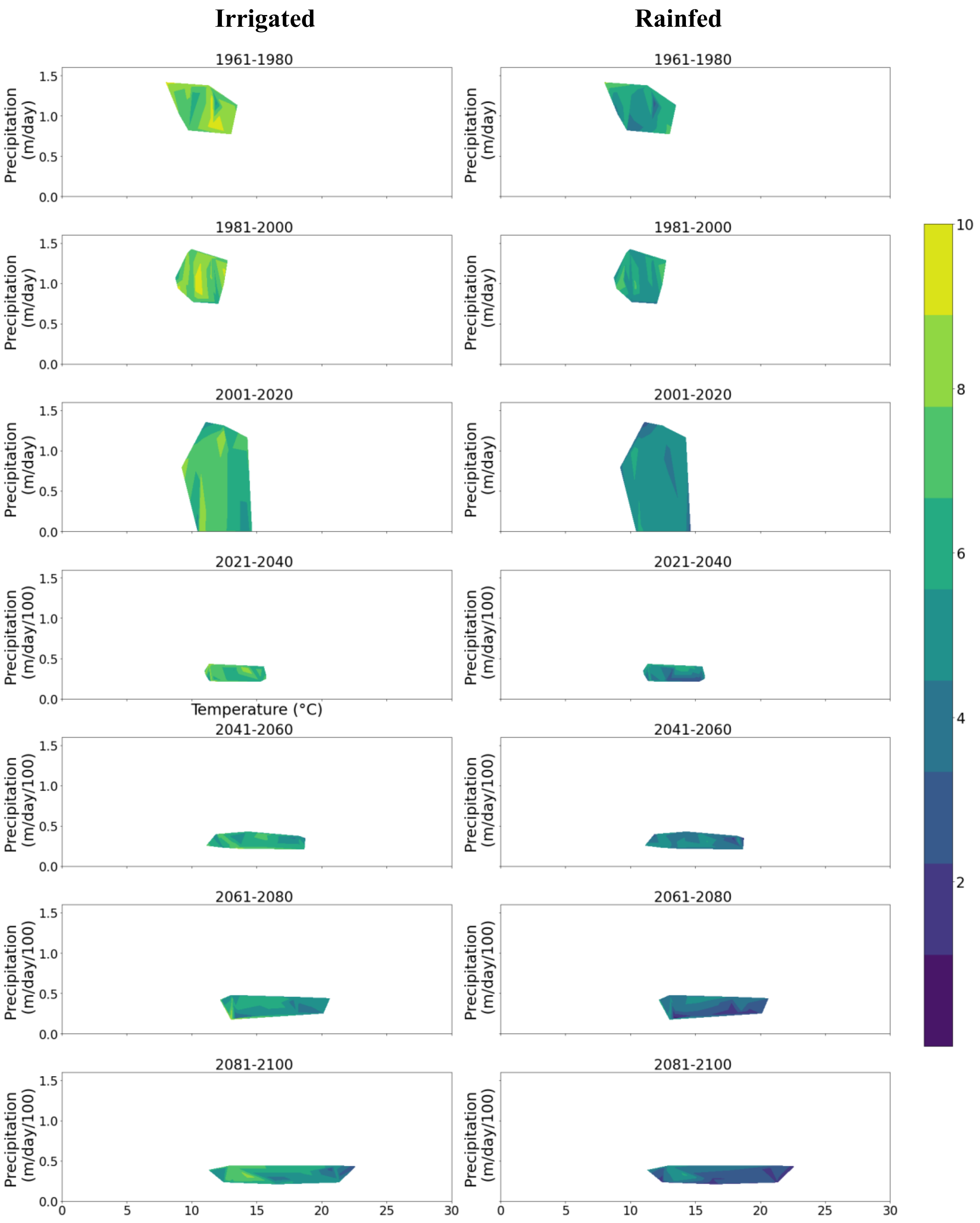

Figure S8: Winter wheat crop yield for a single scenario (cultivar and sowing date) plotted against temperature and precipitation across seven time periods under irrigated and rainfed conditions. Data shown for a grid point located at 40.25° N, 88.75° W in the USA.

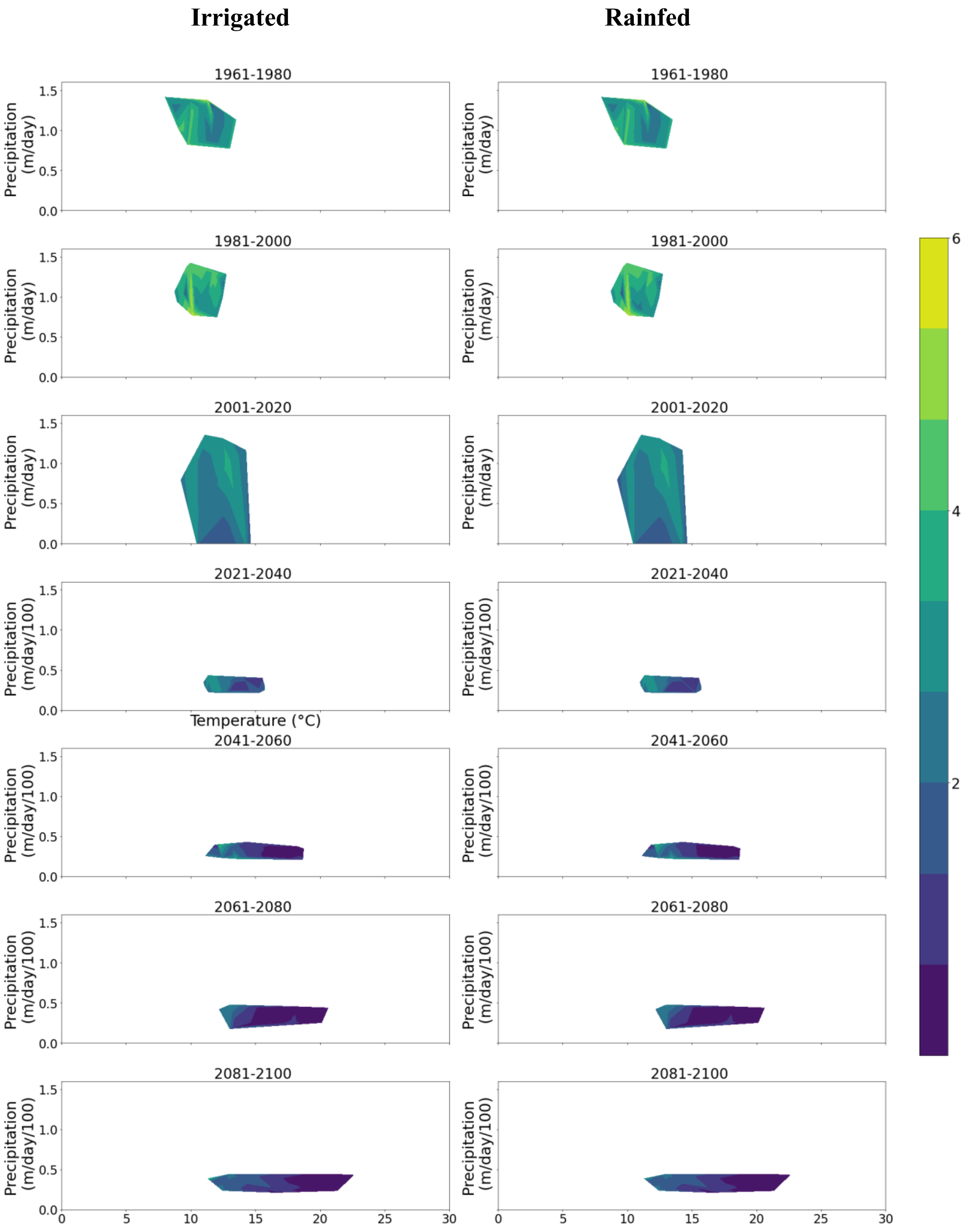

Figure S9: Spring wheat crop yield for a single scenario (cultivar and sowing date) plotted against temperature and precipitation across seven time periods under irrigated and rainfed conditions. Data shown for a grid point located at 40.25° N, 88.75° W in the USA.

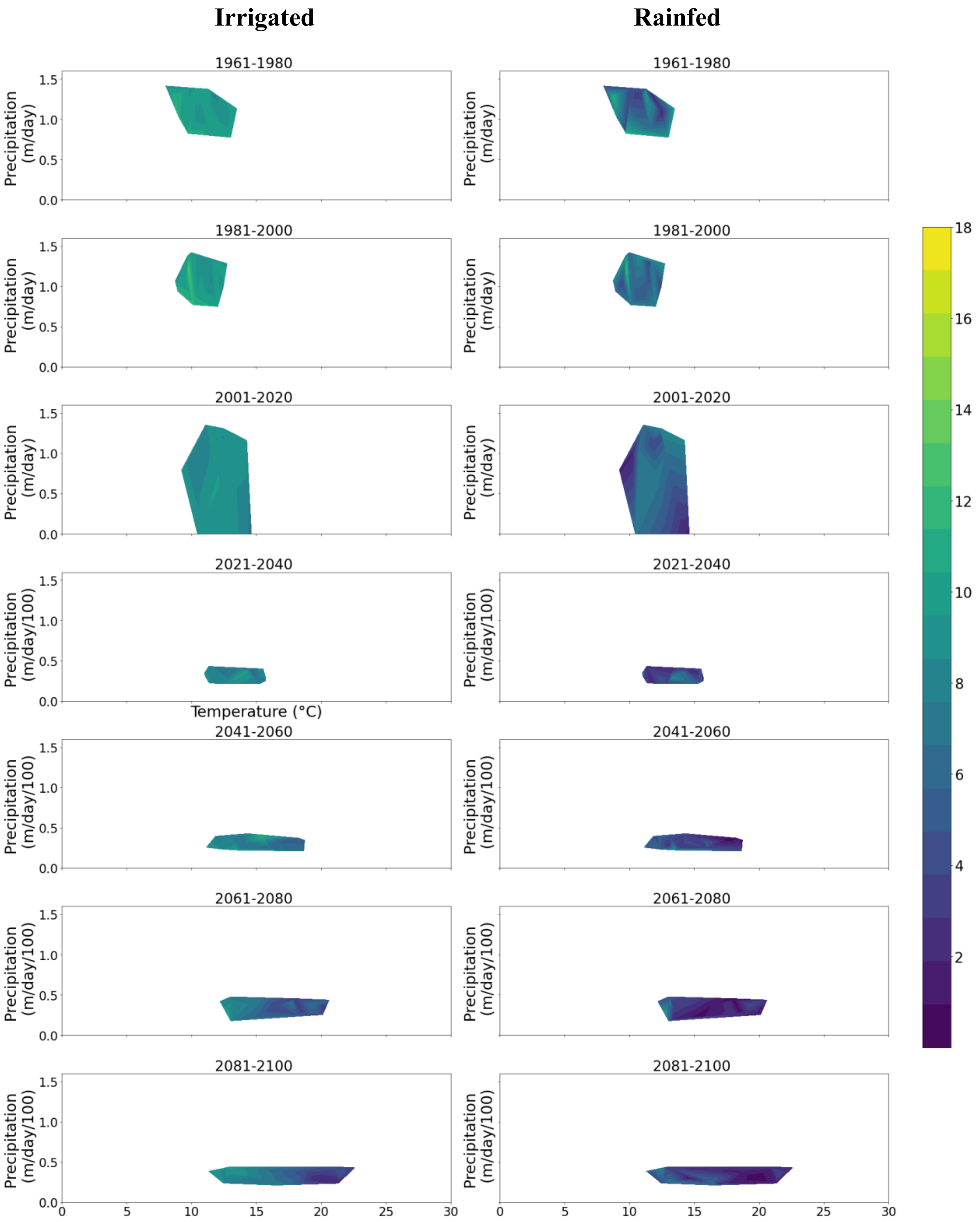

Figure S10: Rice (season-1) crop yield for a single scenario (cultivar and sowing date) plotted against temperature and precipitation across seven time periods under irrigated and rainfed conditions. Data shown for a grid point located at 40.25° N, 88.75° W in the USA.

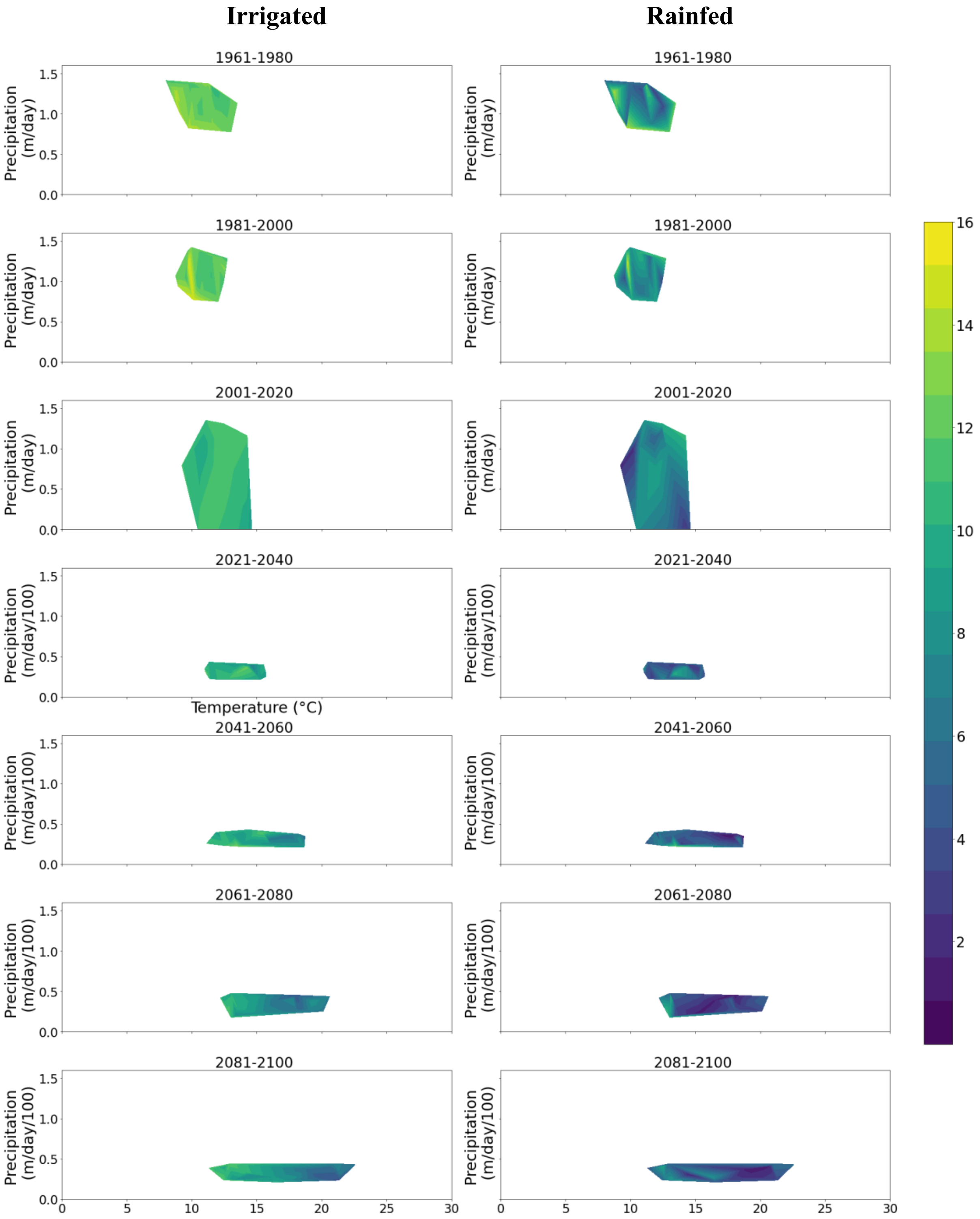

Figure S11: Rice (season-2) crop yield for a single scenario (cultivar and sowing date) plotted against temperature and precipitation across seven time periods under irrigated and rainfed conditions. Data shown for a grid point located at 40.25° N, 88.75° W in the USA.

S5. Scenario-based analysis

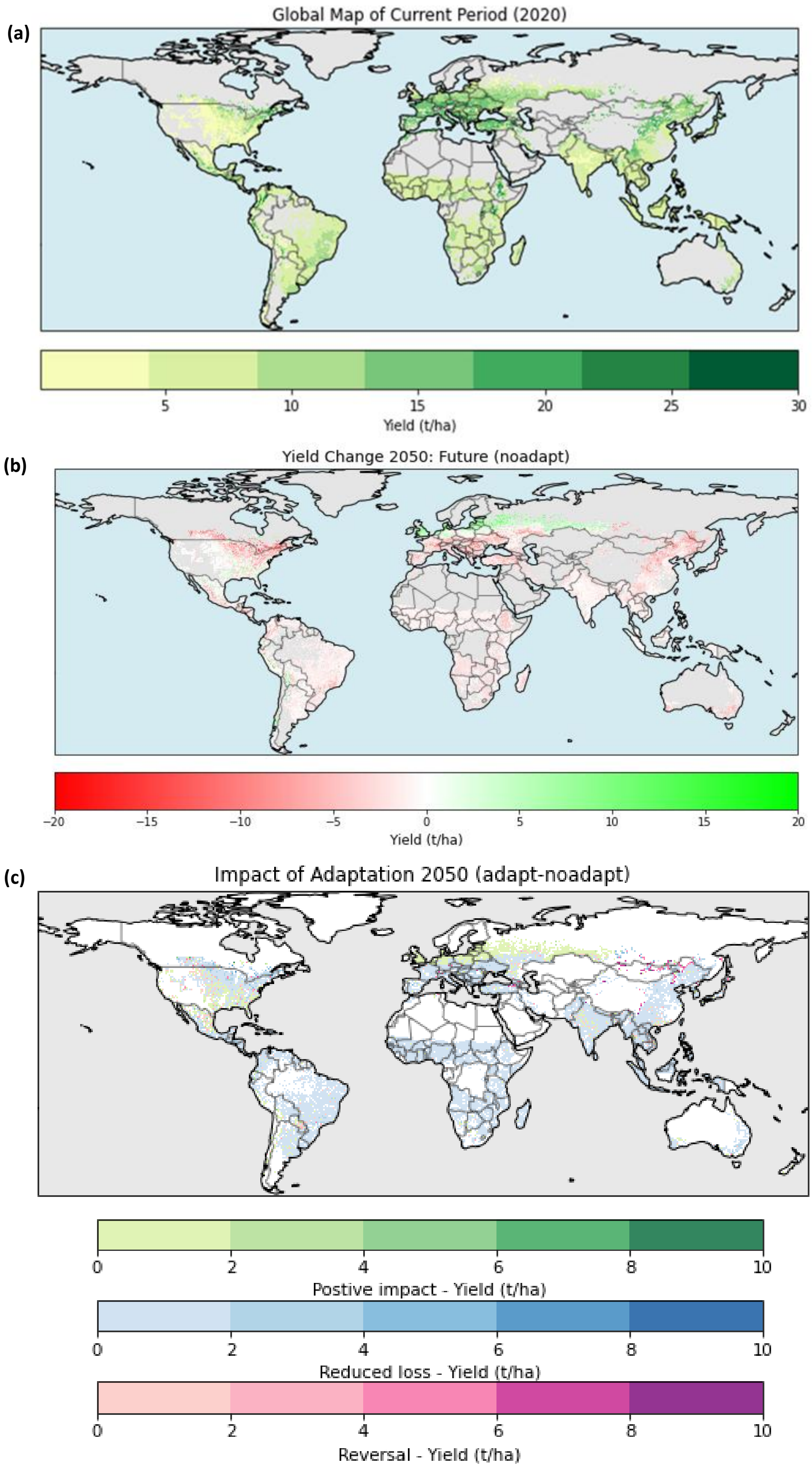

Figure 12: Global rainfed maize reliability: a) Baseline yield in 2020, b) Projected yield change by 2050 without adaptation and c) Impact of adaptation on 2050 yields. Shown for crop-specific masks.

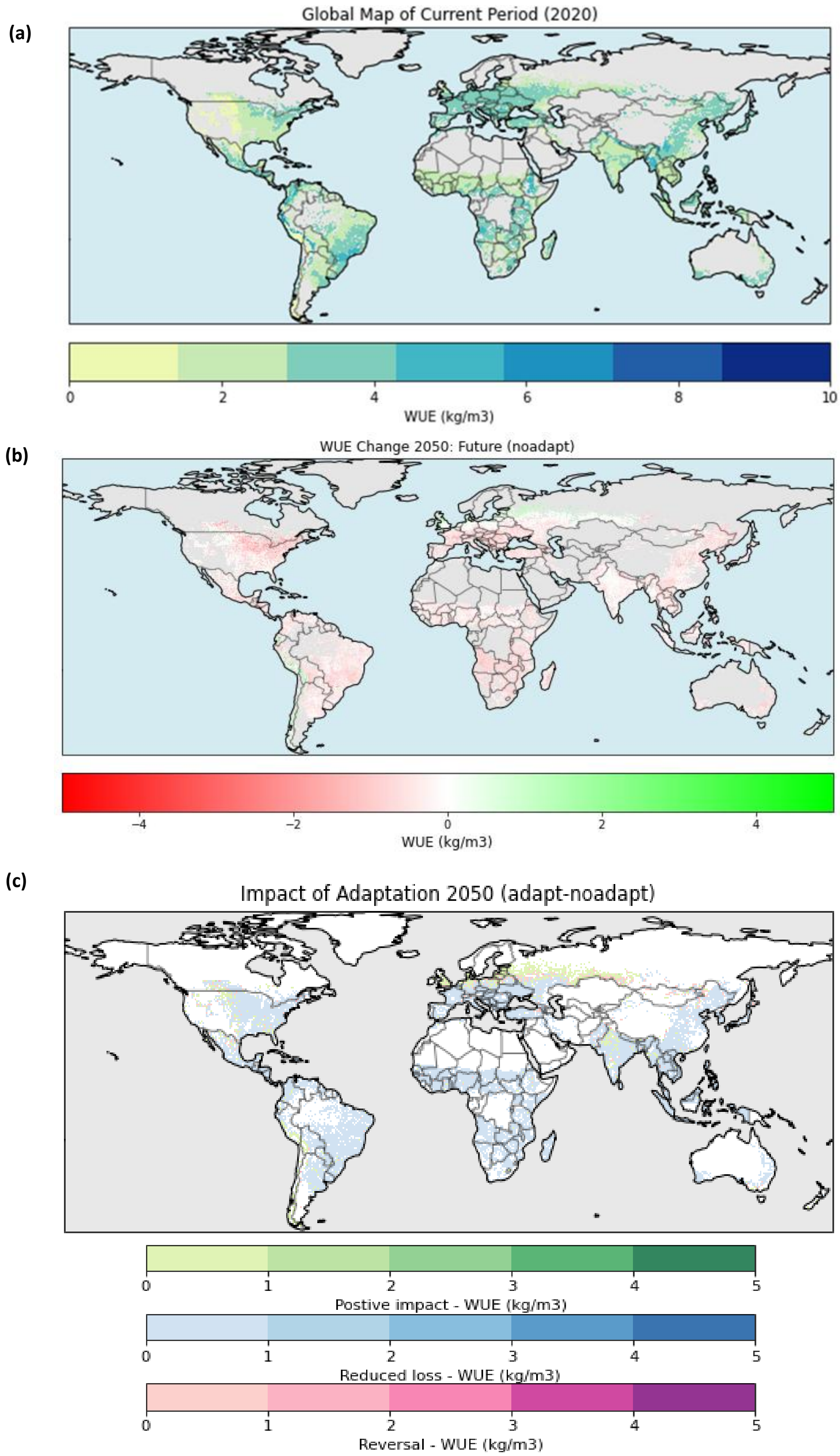

Figure 13: Global rainfed maize water use efficiency: a) Baseline water use efficiency in 2020, b) Projected water use efficiency change by 2050 without adaptation and c) Impact of adaptation on 2050 water use efficiency. Shown for crop-specific masks.

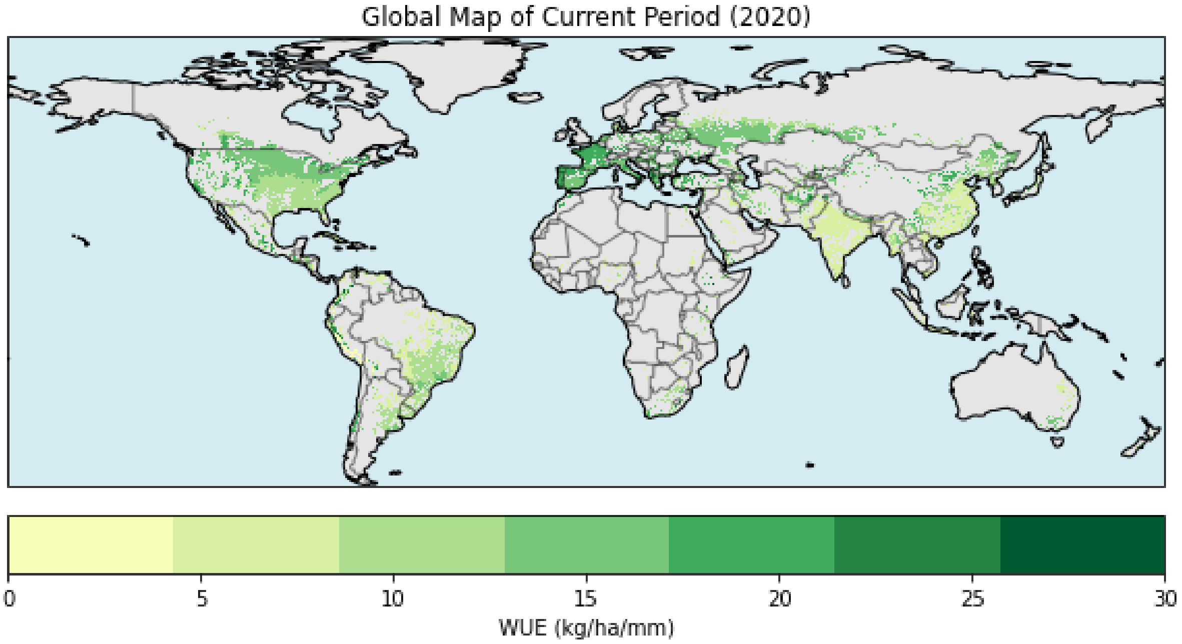

Figure S14: Global irrigated maize productivity: Baseline yield in 2020

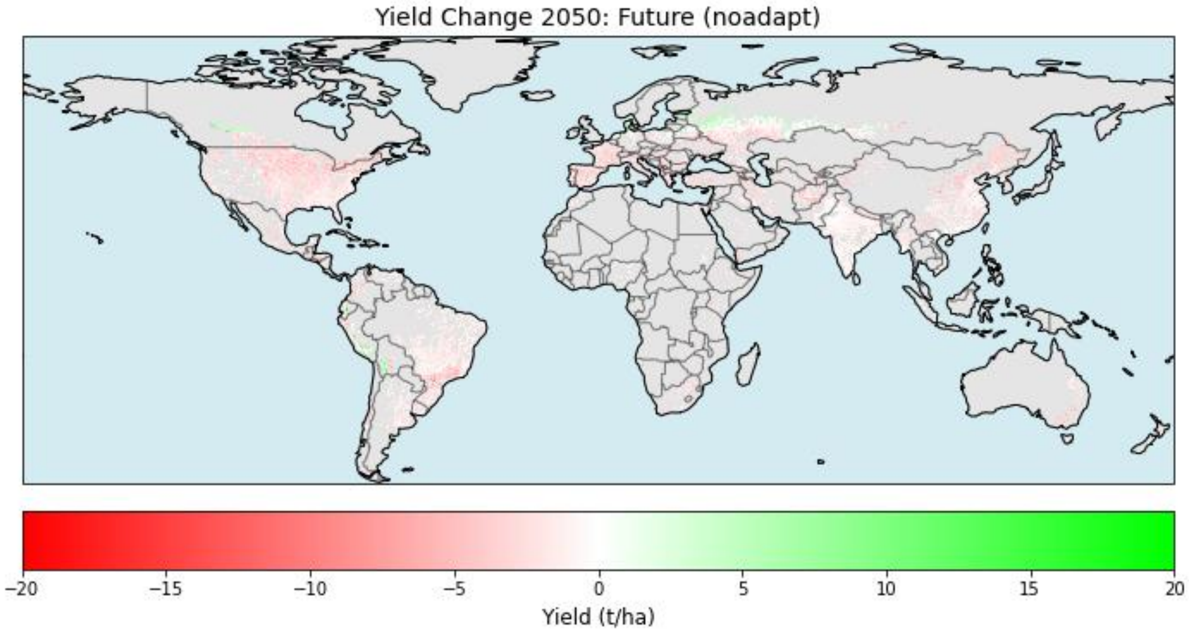

Figure S15: Global irrigated maize productivity: Projected yield change by 2050 without adaptation

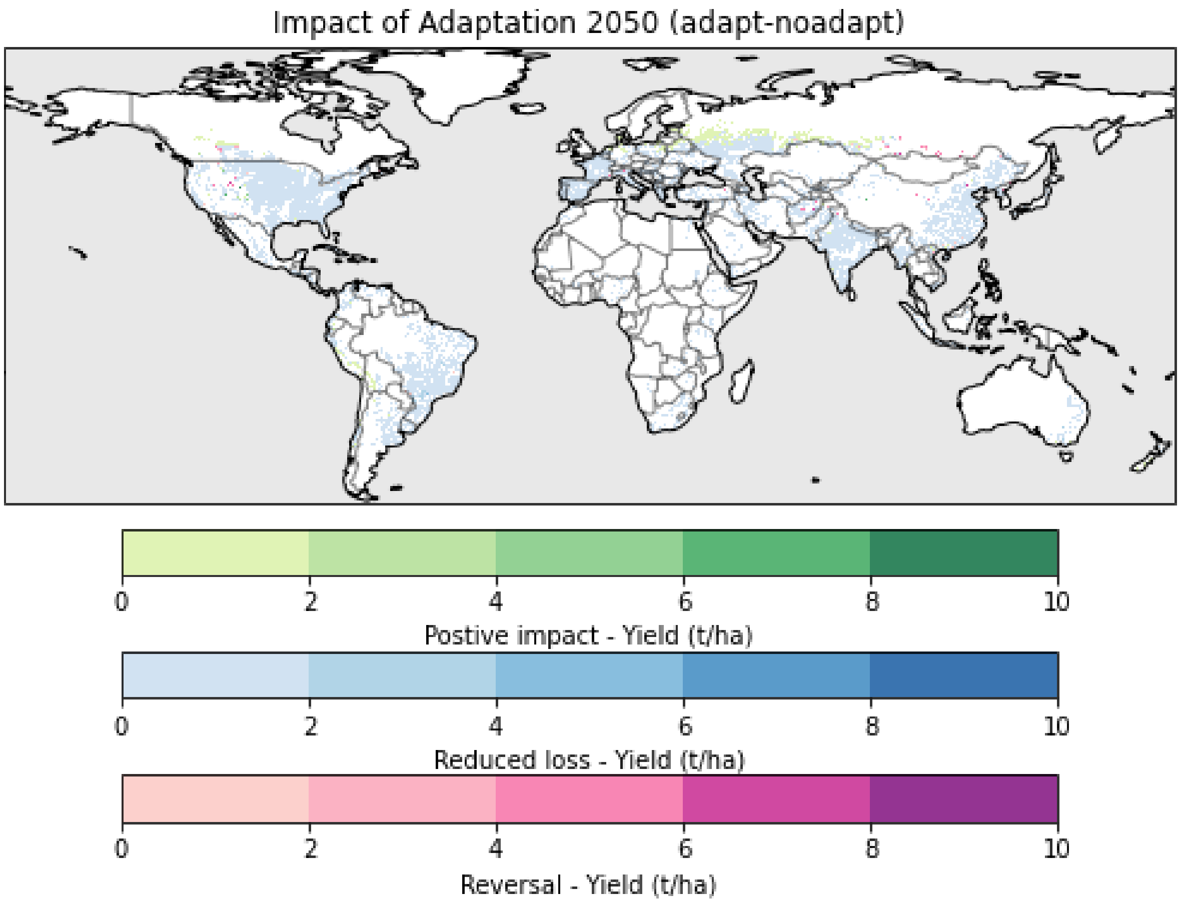

Figure S16: Global irrigated maize productivity: Impact of adaptation on 2050 yields

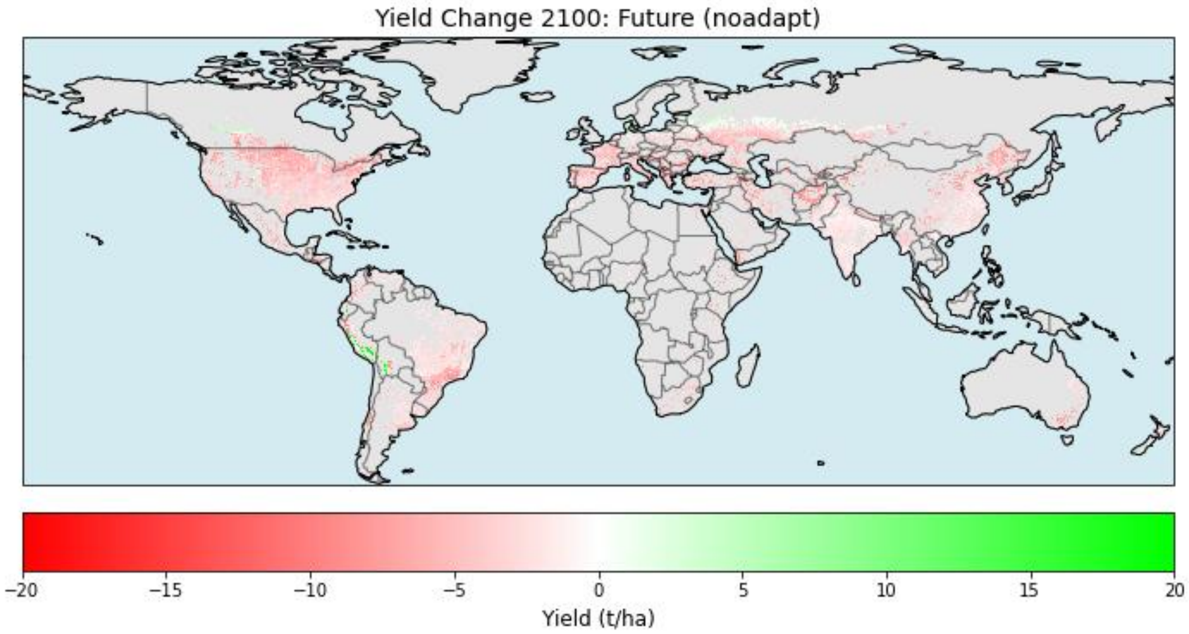

Figure S17: Global irrigated maize productivity: Projected yield change by 2100 without adaptation

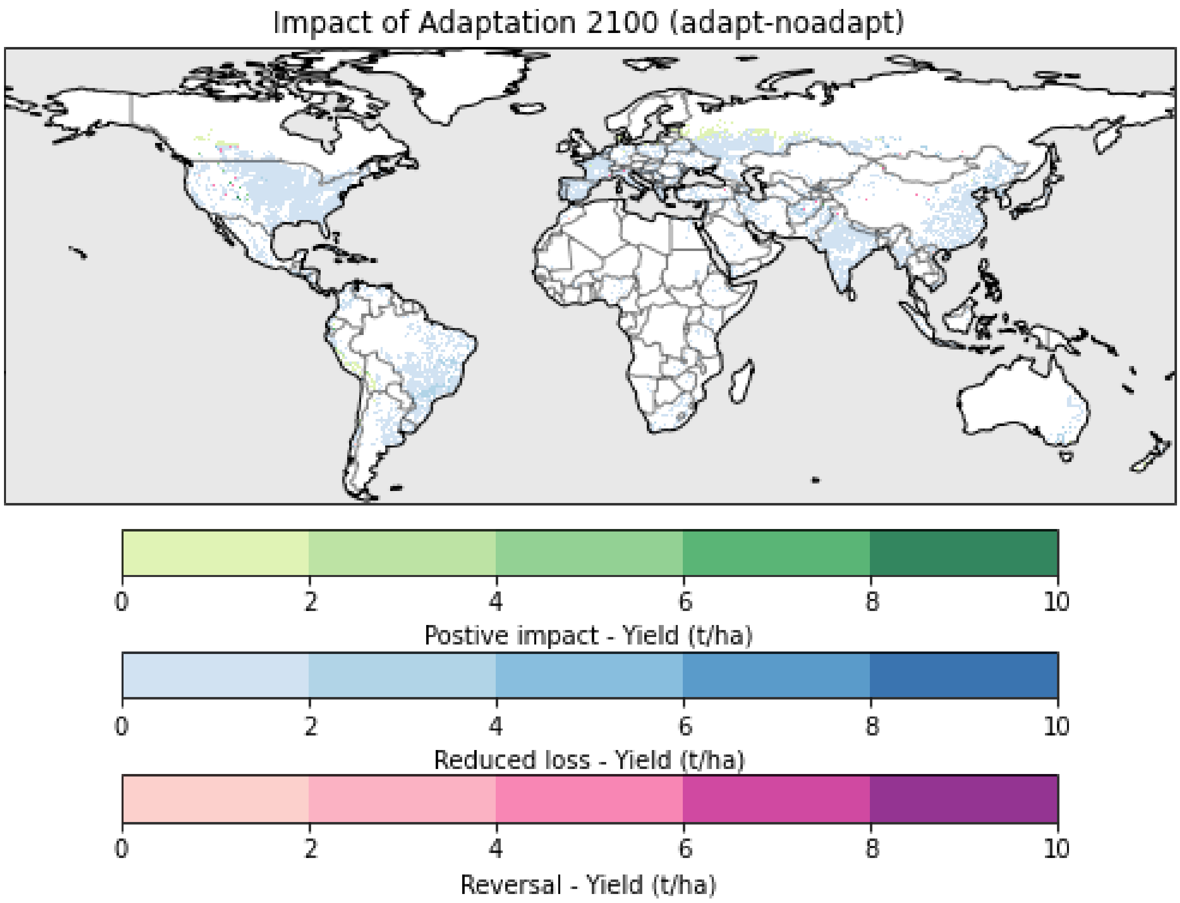

Figure S18: Global irrigated maize productivity: Impact of adaptation on 2100 yields

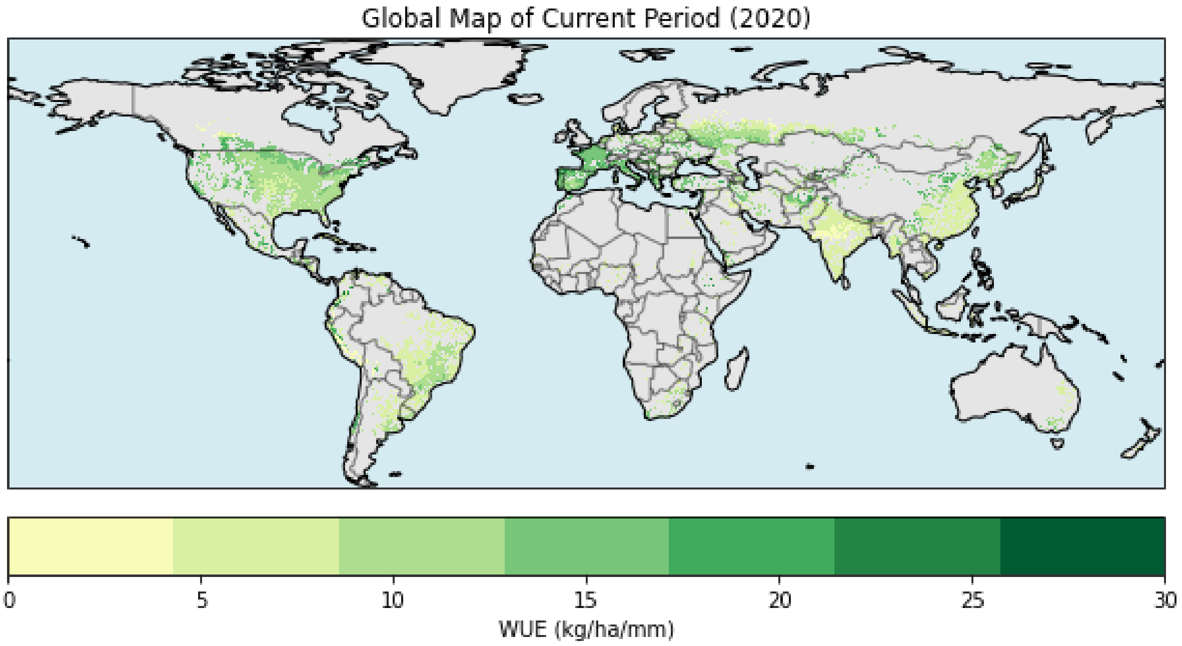

Figure S19: Global irrigated maize reliability: Baseline yield in 2020

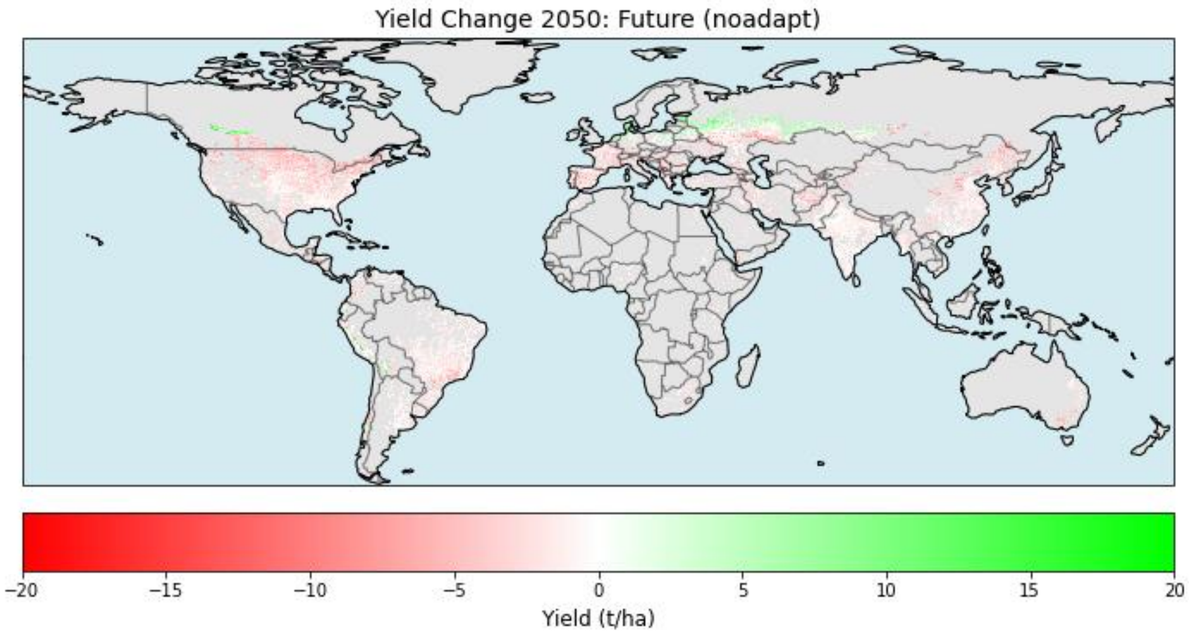

Figure S20: Global irrigated maize reliability: Projected yield change by 2050 without adaptation

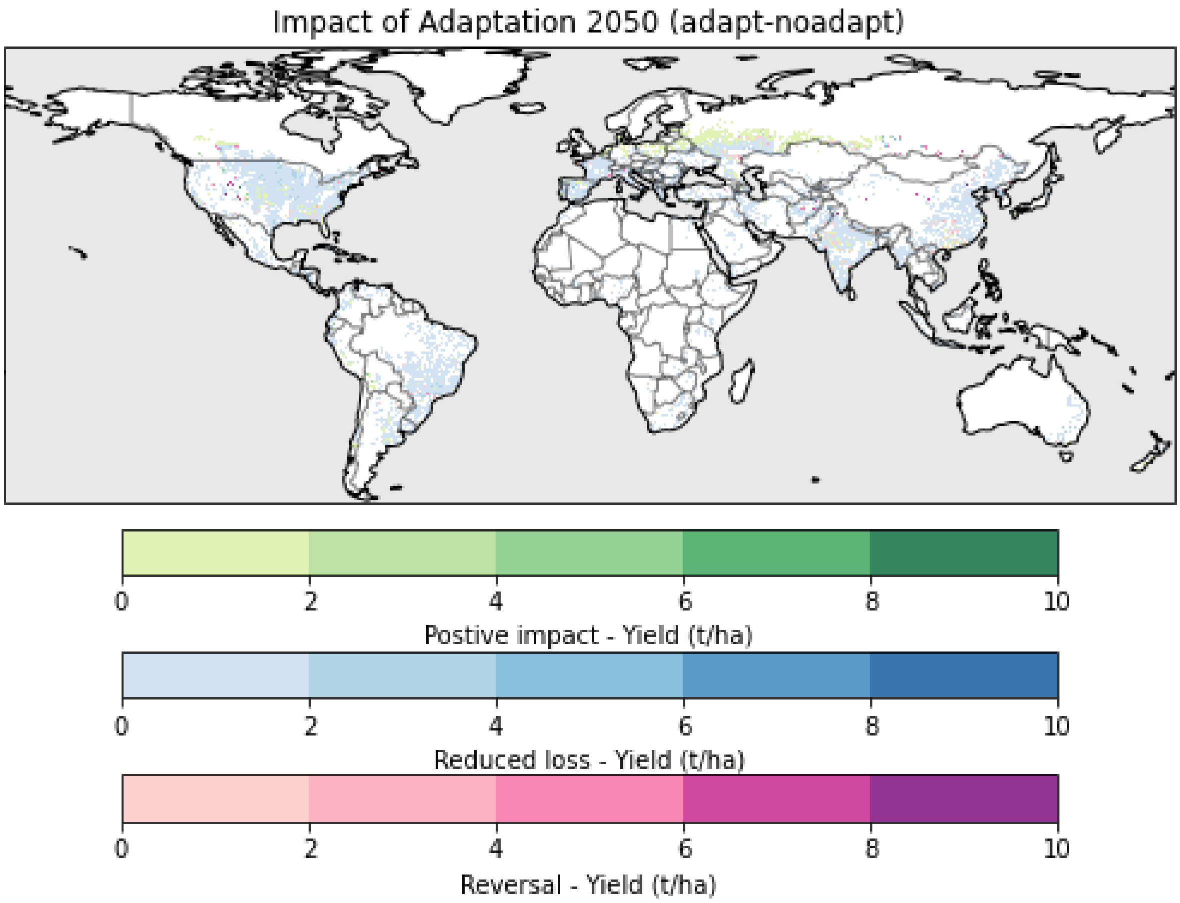

Figure S21: Global irrigated maize reliability: Impact of adaptation on 2050 yields

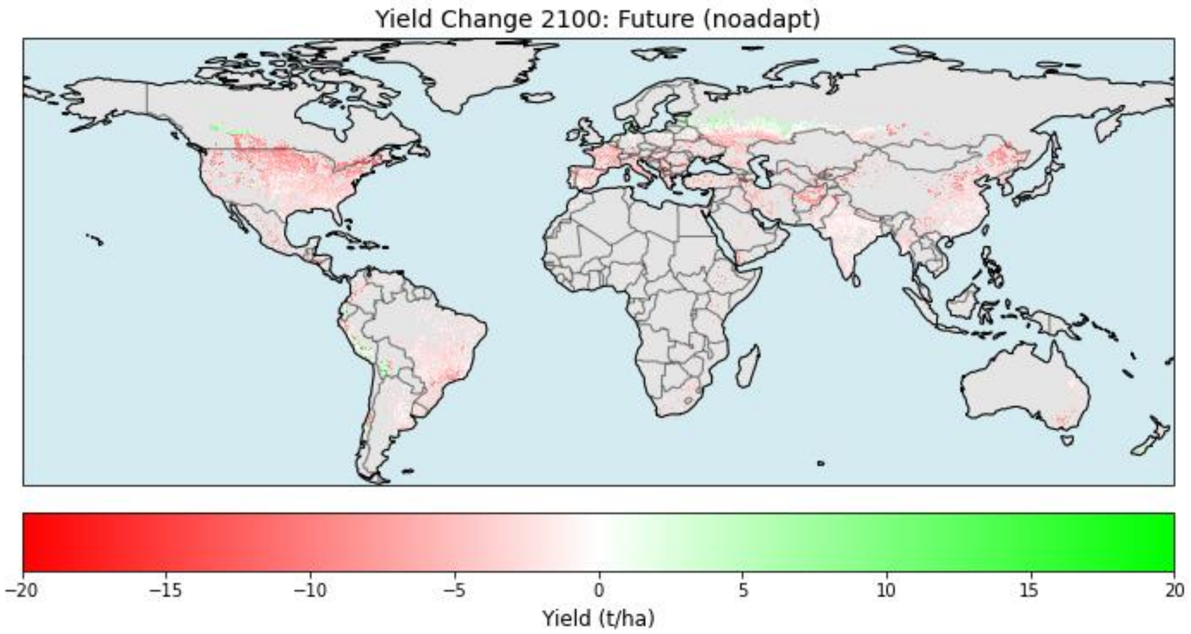

Figure S22: Global irrigated maize reliability: Projected yield change by 2100 without adaptation

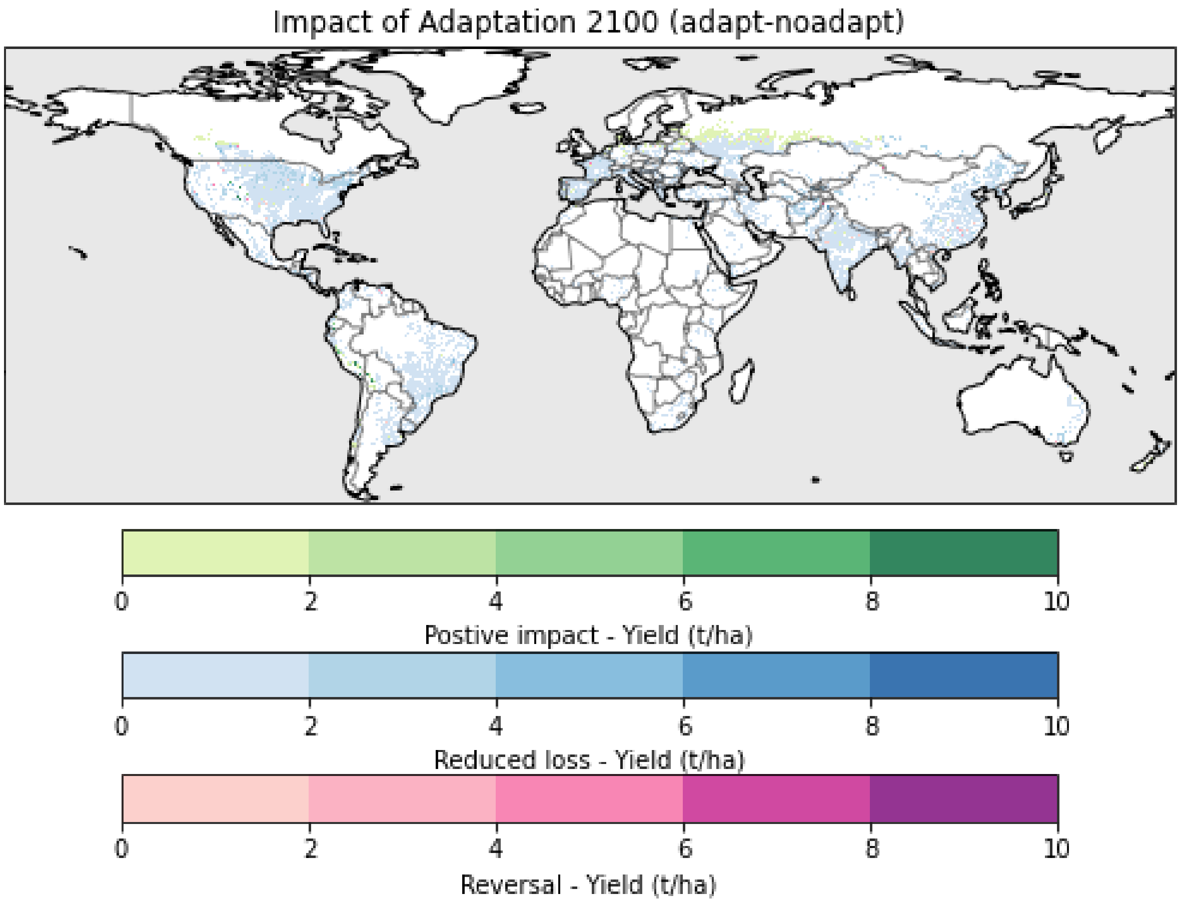

Figure S23: Global irrigated maize reliability: Impact of adaptation on 2100 yields

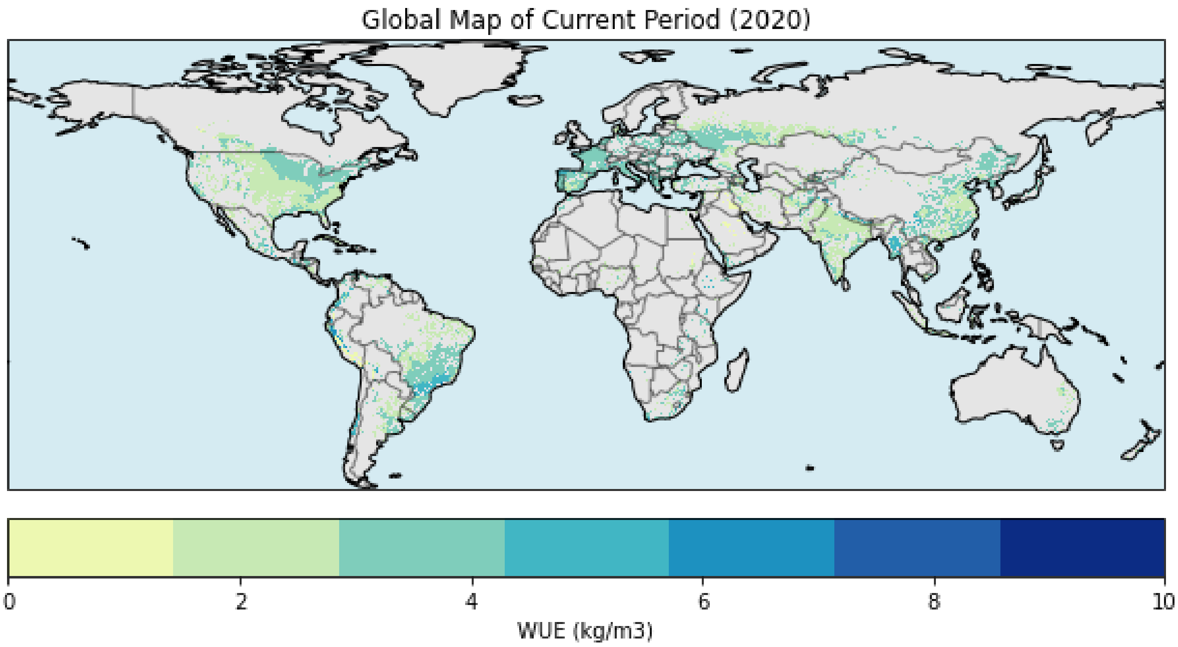

Figure S24: Global irrigated maize water use efficiency: Baseline water use efficiency in 2020

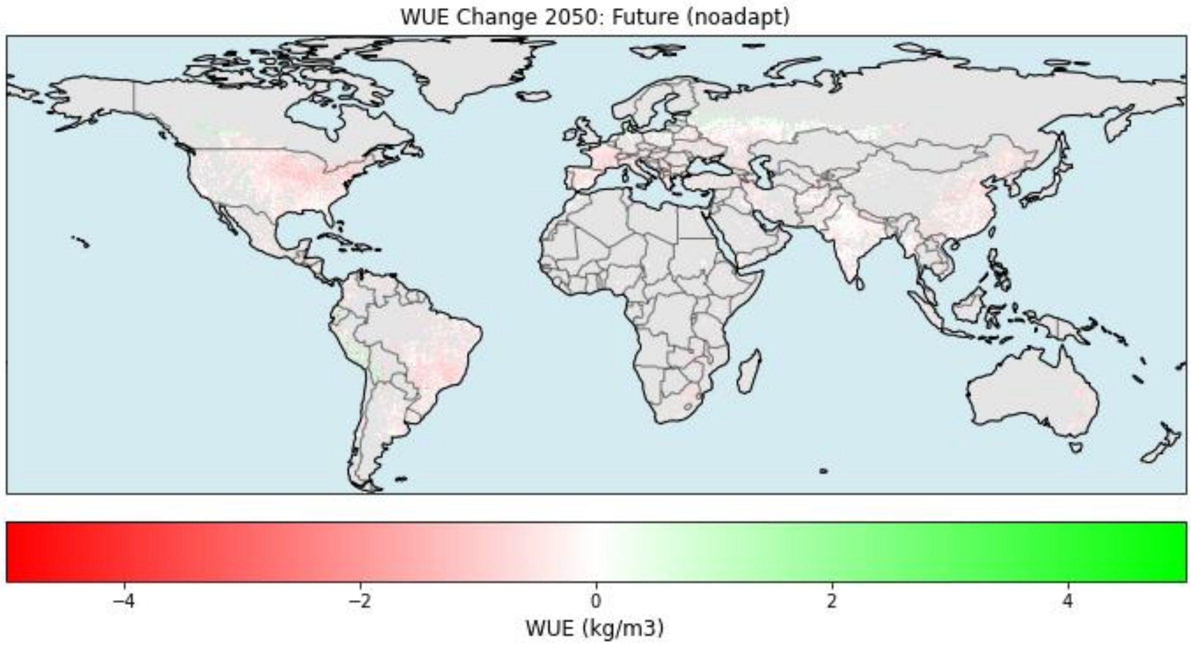

Figure S25: Global irrigated maize water use efficiency: Projected water use efficiency change by 2050 without adaptation

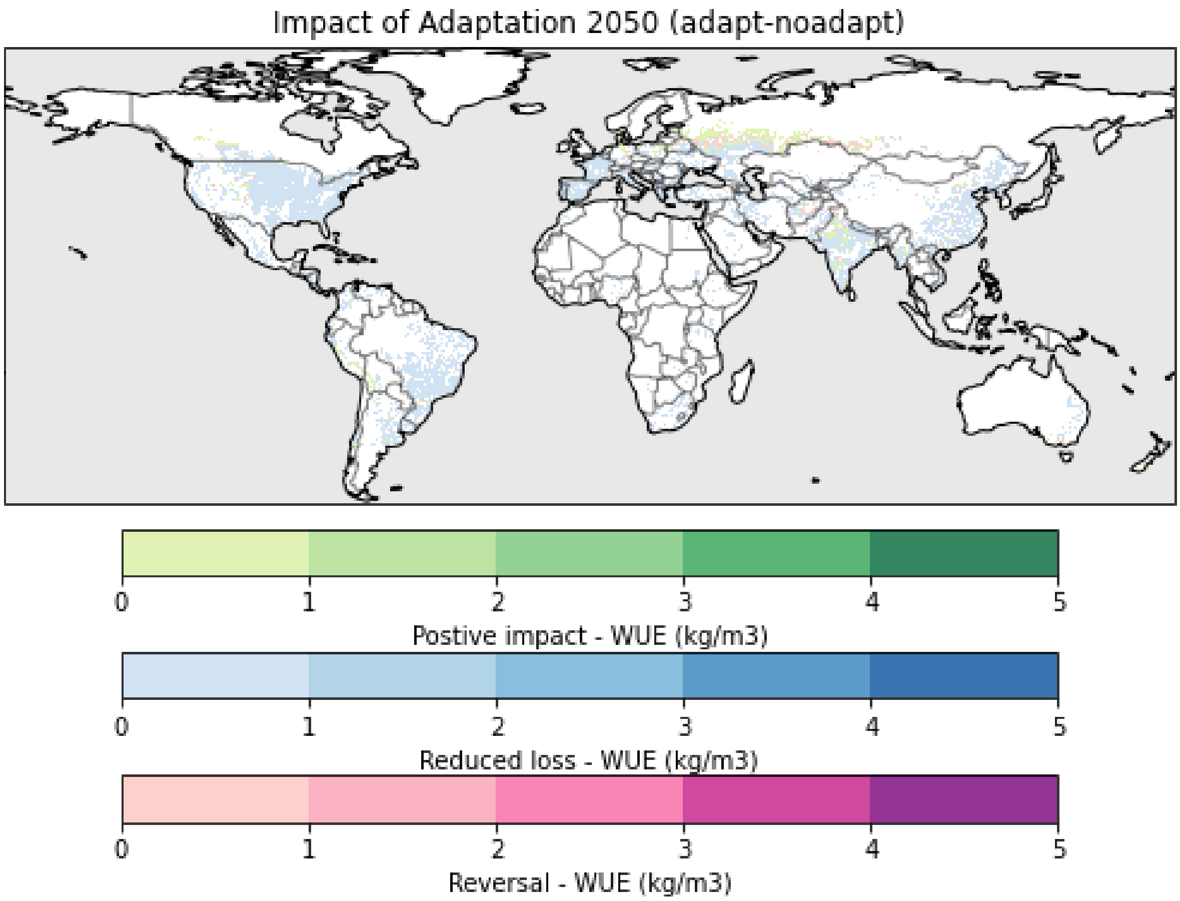

Figure S26: Global irrigated maize water use efficiency: Impact of adaptation on 2050 water use efficiency

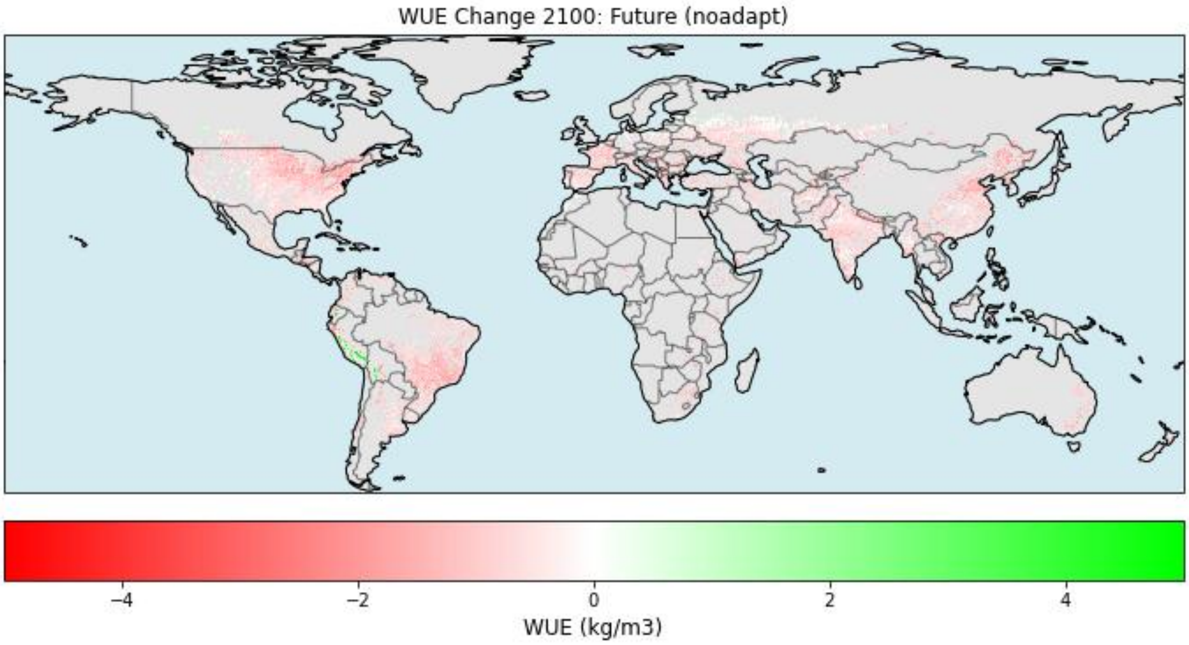

Figure S27: Global irrigated maize water use efficiency: Projected water use efficiency change by 2100 without adaptation

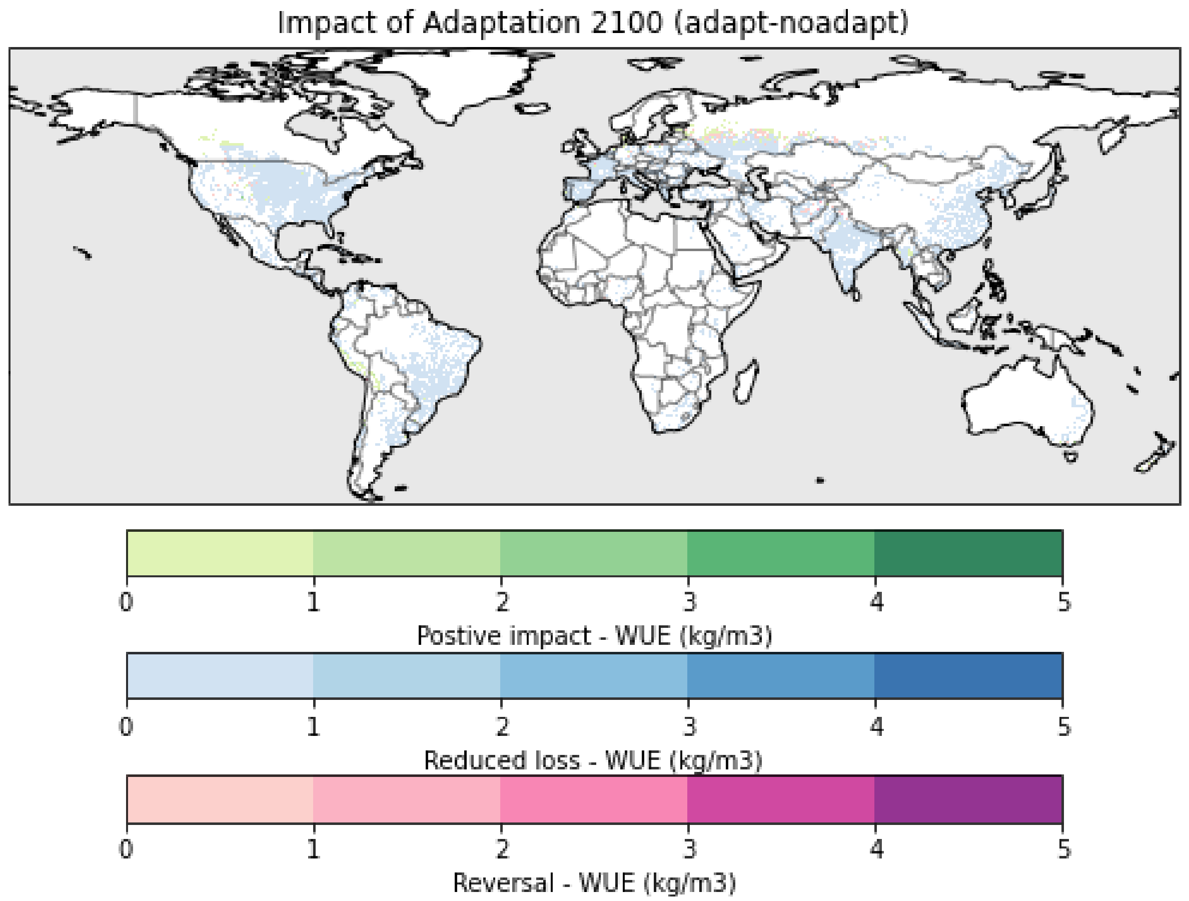

Figure S28: Global irrigated maize water use efficiency: Impact of adaptation on 2100 water use efficiency

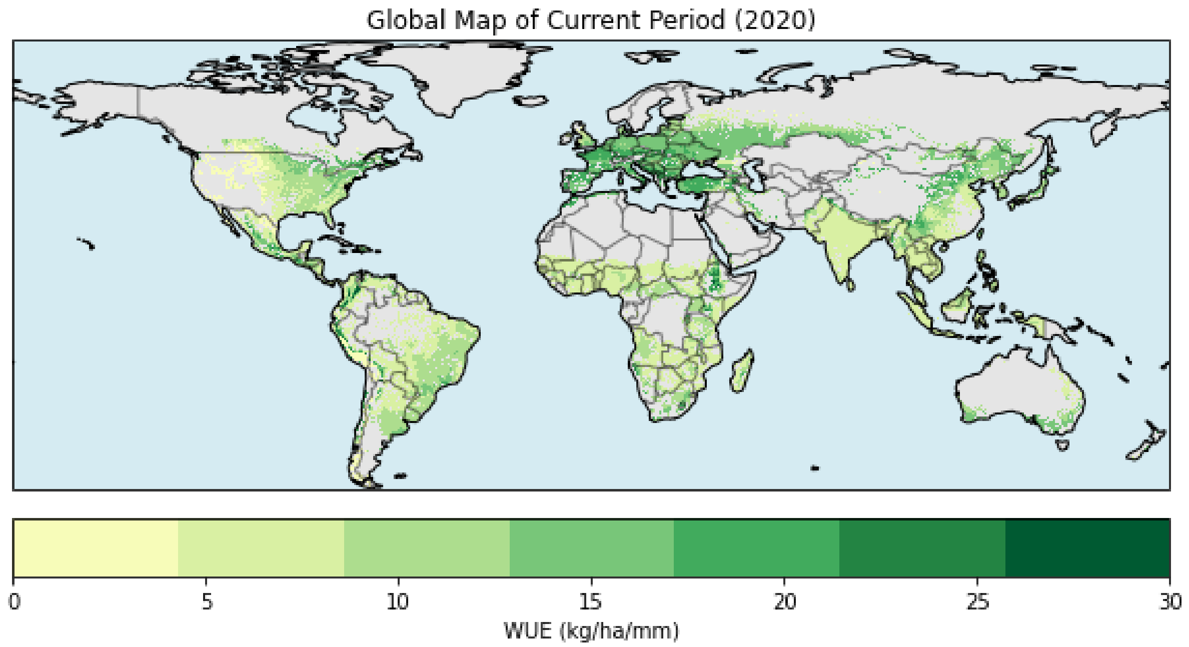

Figure S29: Global rainfed maize productivity: Baseline yield in 2020

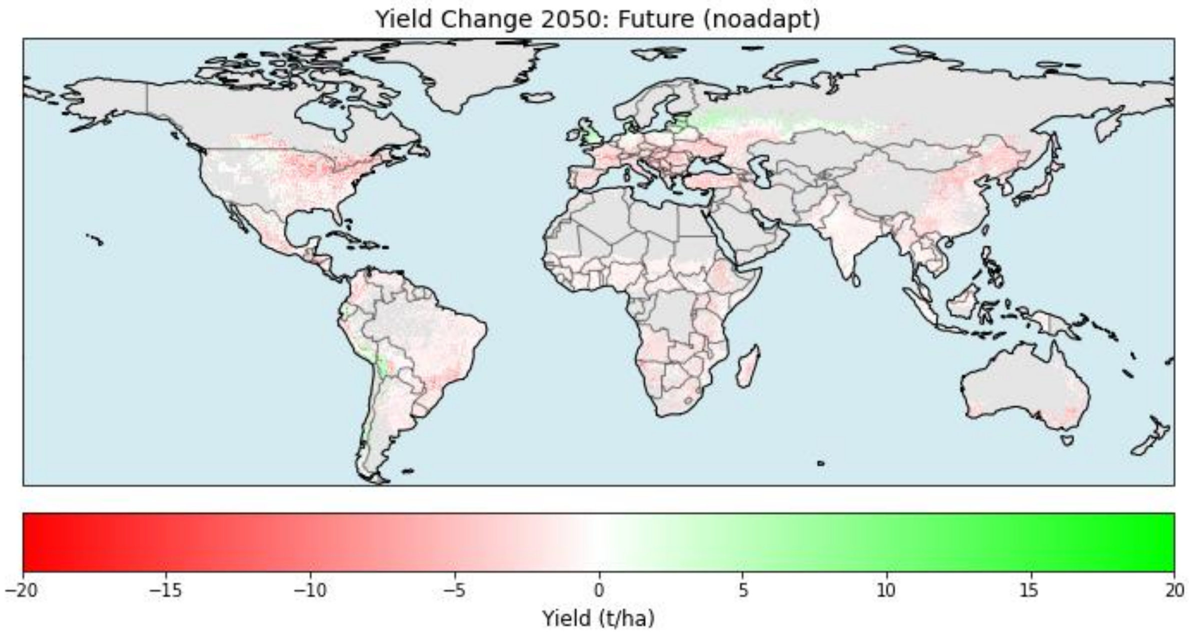

Figure S30: Global rainfed maize productivity: Projected yield change by 2050 without adaptation

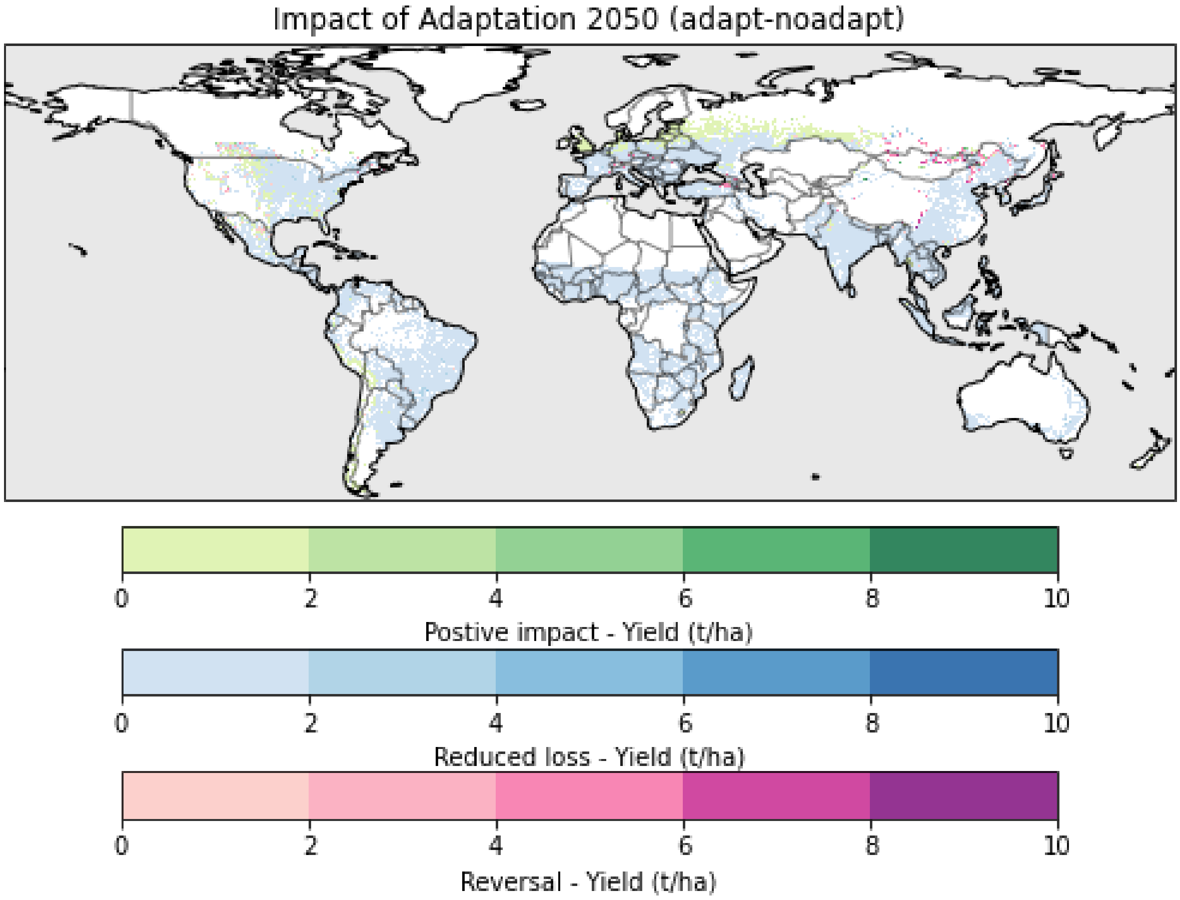

Figure S31: Global rainfed maize productivity: Impact of adaptation on 2050 yields

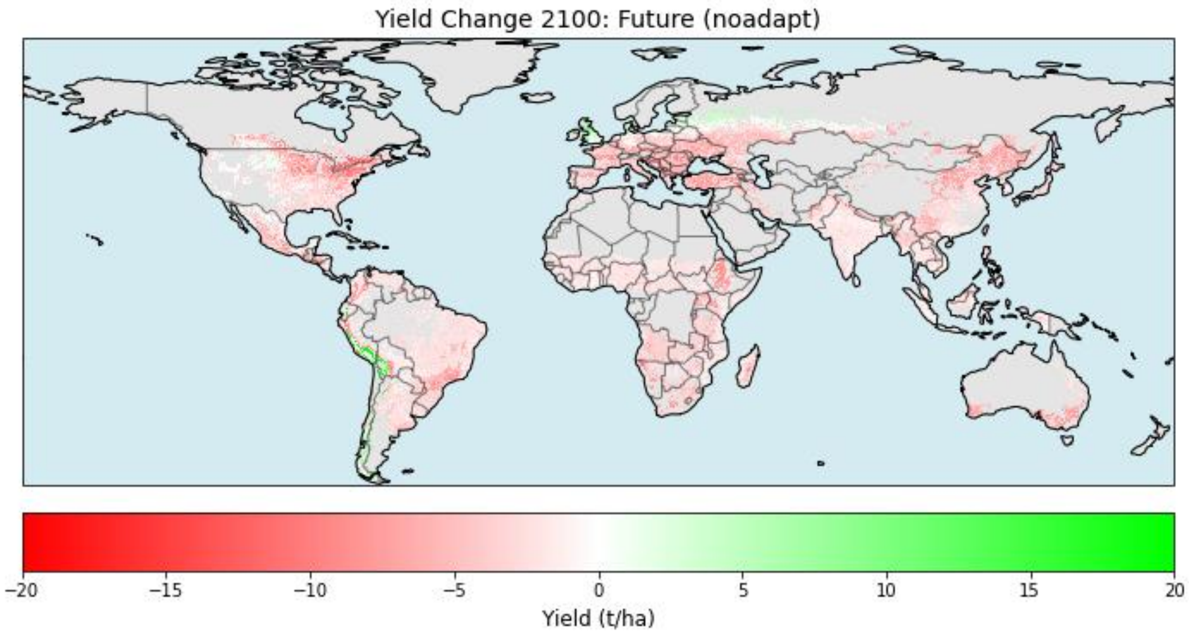

Figure S32: Global rainfed maize productivity: Projected yield change by 2100 without adaptation

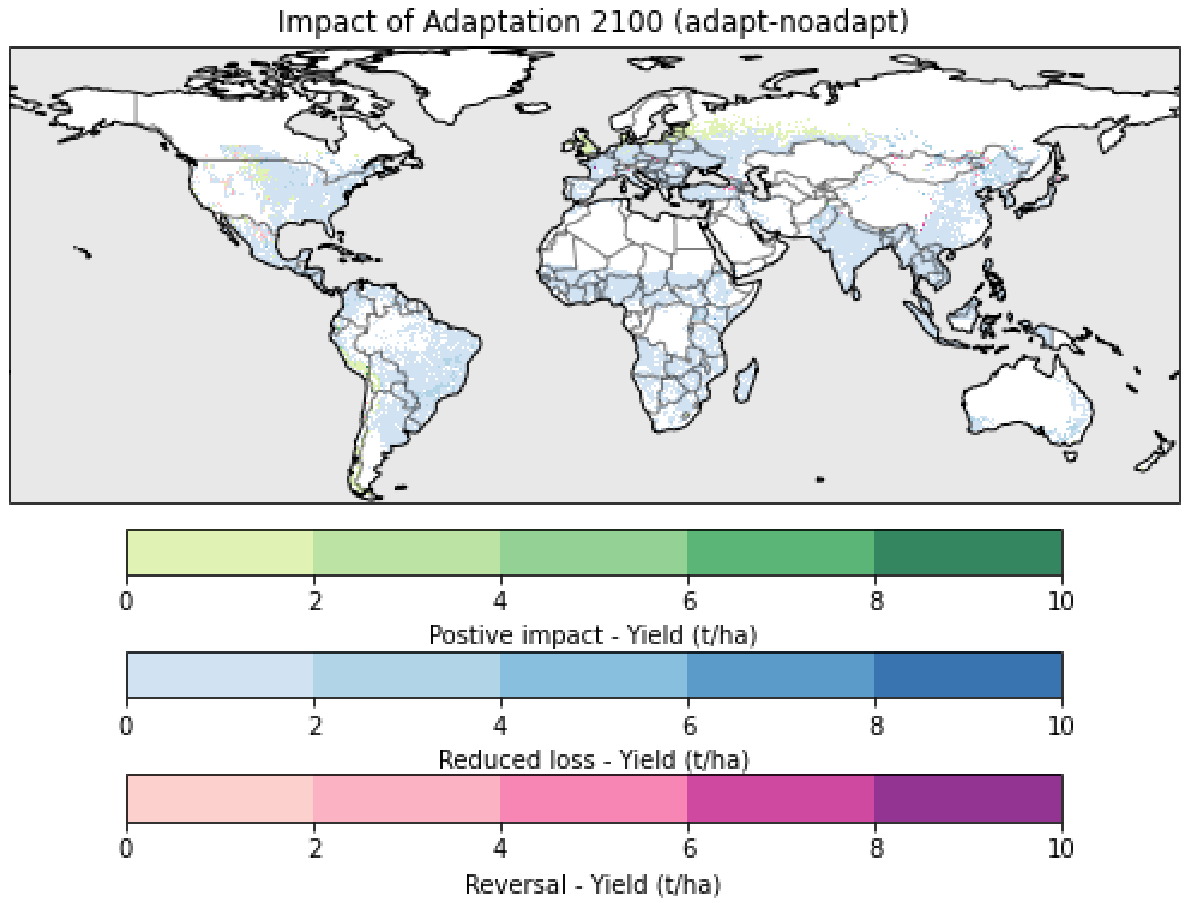

Figure S33: Global rainfed maize productivity: Impact of adaptation on 2100 yields

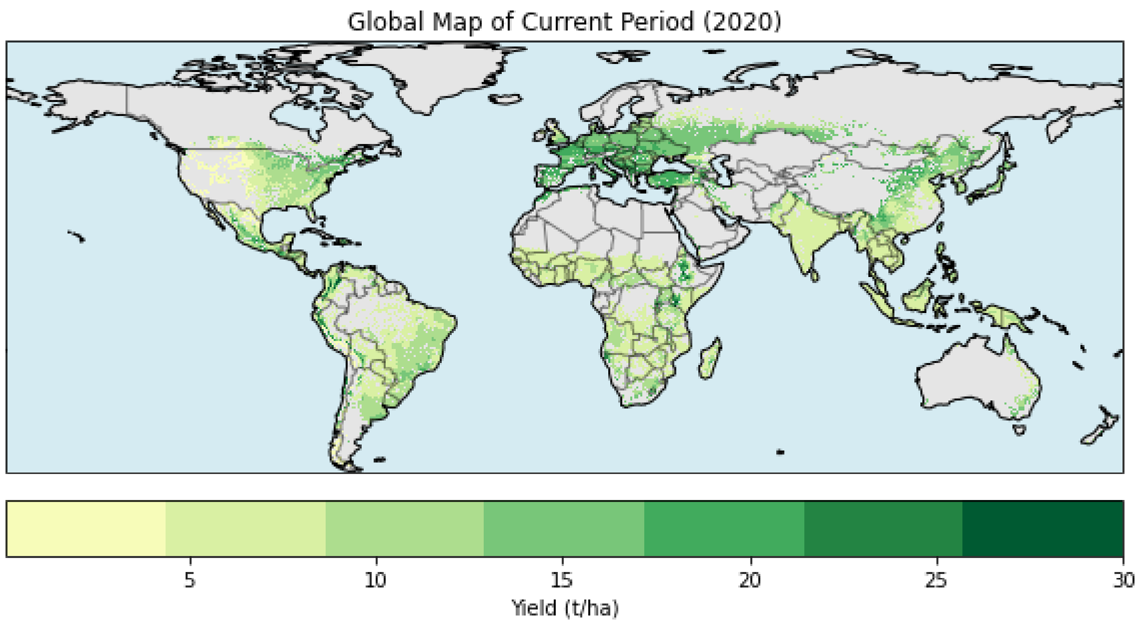

Figure S34: Global rainfed maize reliability: Baseline yield in 2020

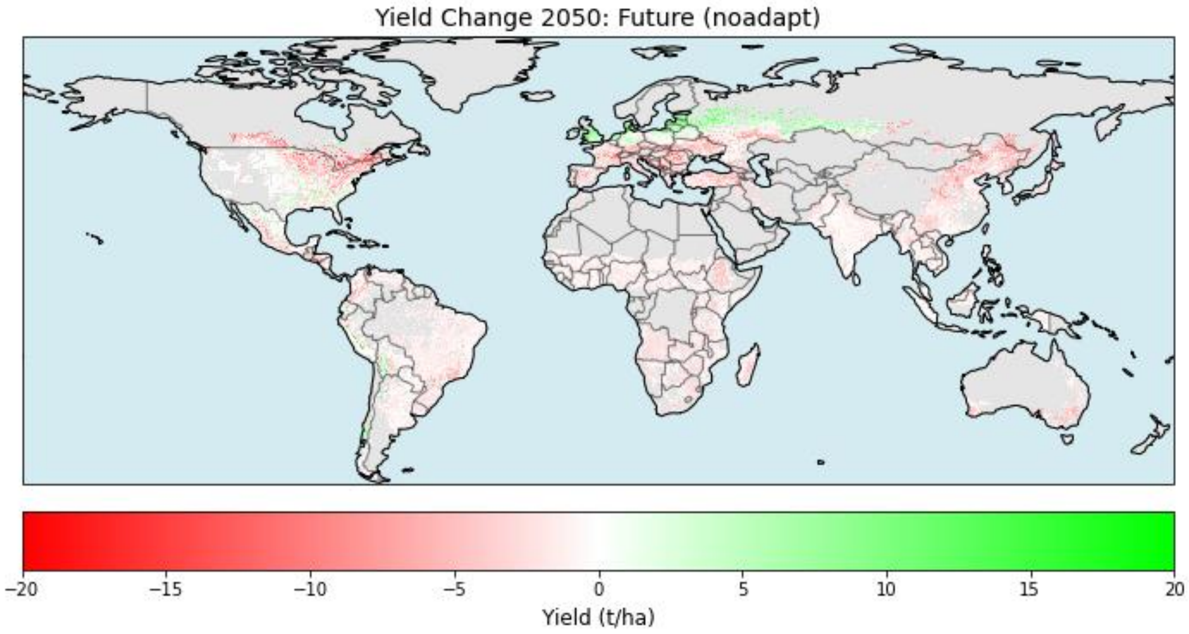

Figure S35: Global rainfed maize reliability: Projected yield change by 2050 without adaptation

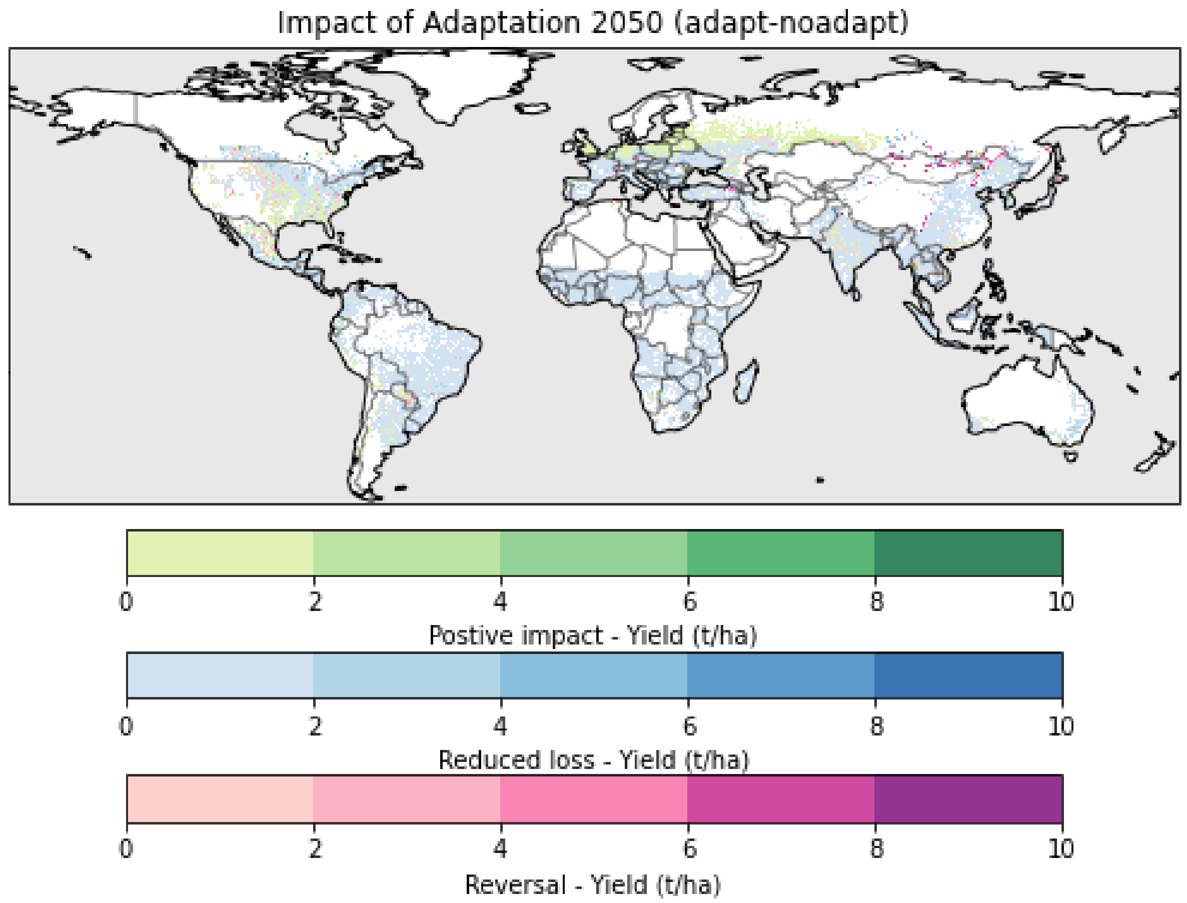

Figure S36: Global rainfed maize reliability: Impact of adaptation on 2050 yields

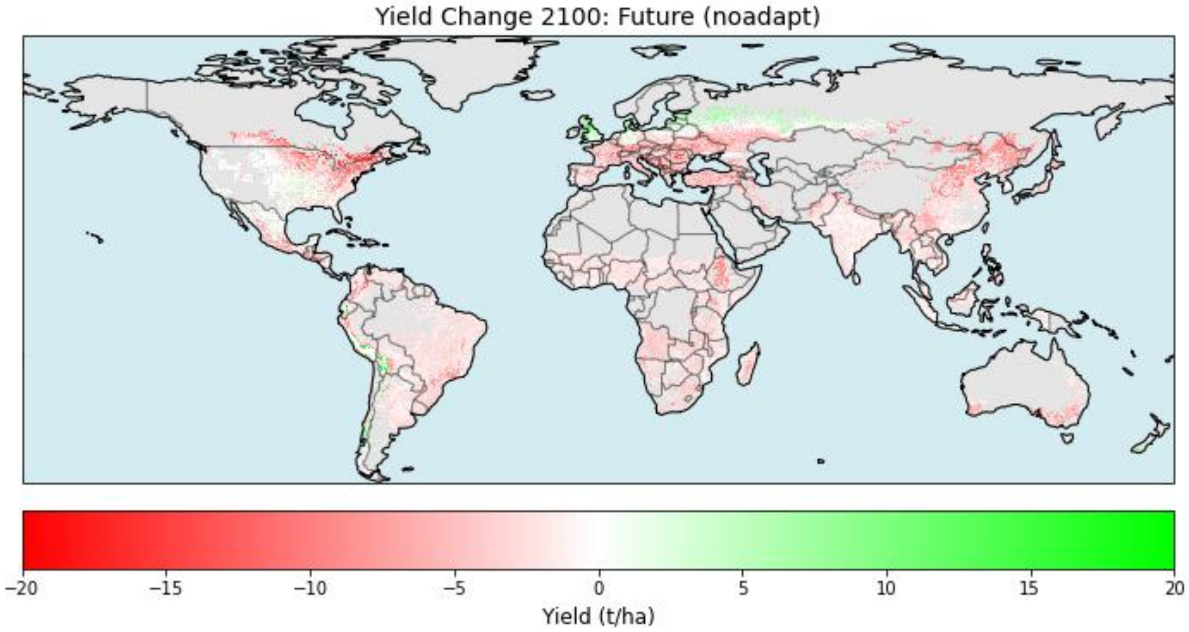

Figure S37: Global rainfed maize reliability: Projected yield change by 2100 without adaptation

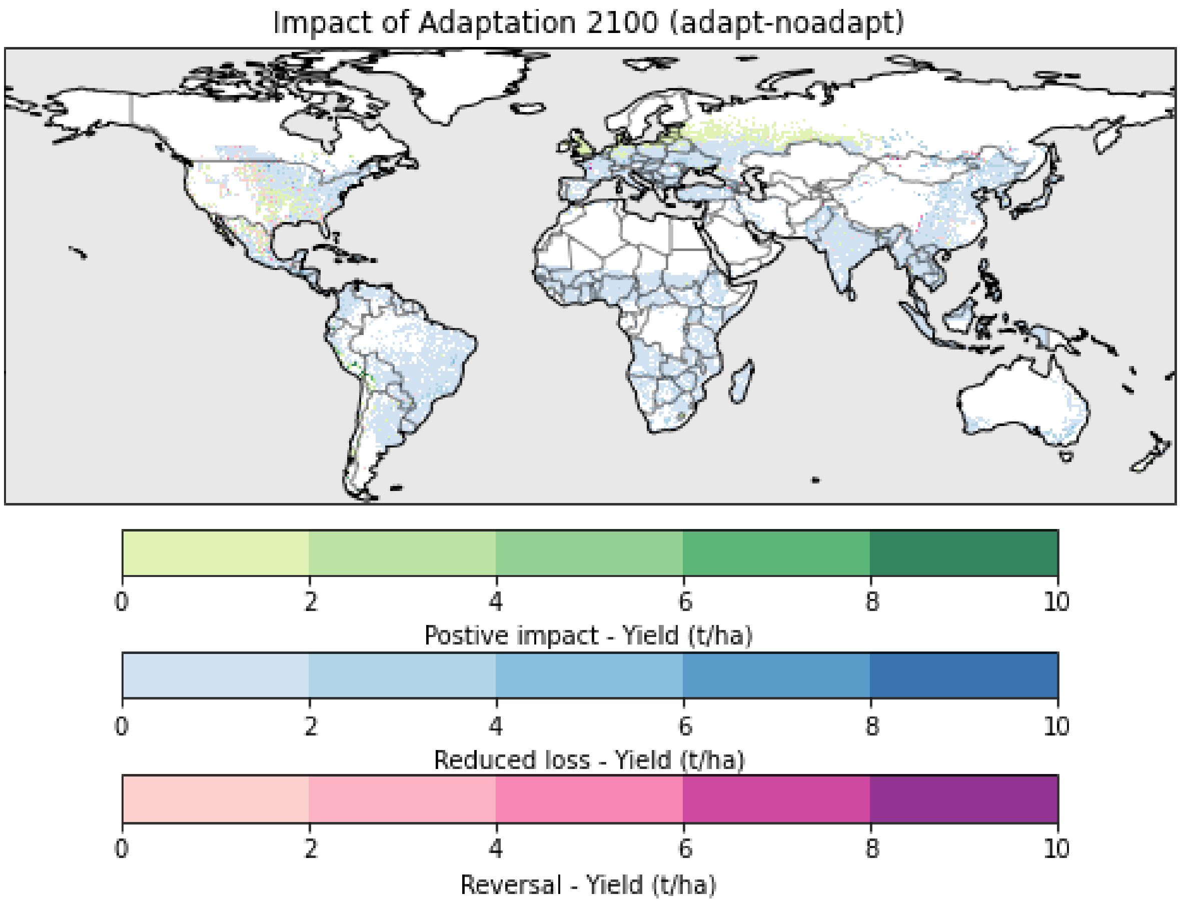

Figure S38: Global rainfed maize reliability: Impact of adaptation on 2100 yields

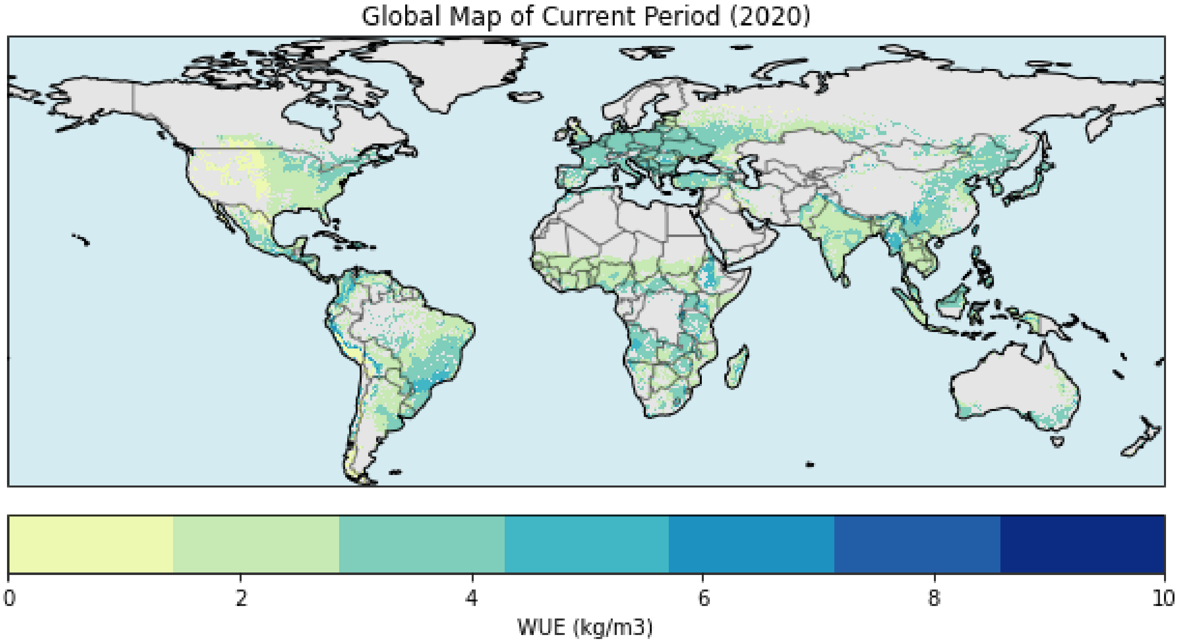

Figure S39: Global rainfed maize water use efficiency: Baseline water use efficiency in 2020

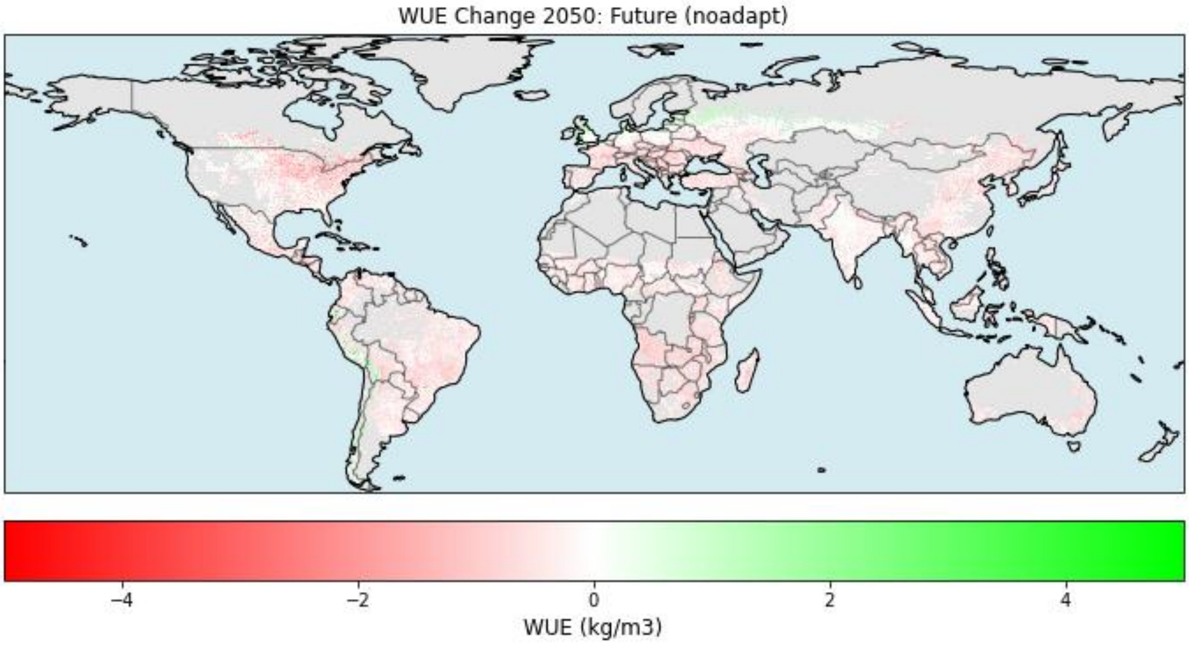

Figure S40: Global rainfed maize water use efficiency: Projected water use efficiency change by 2050 without adaptation

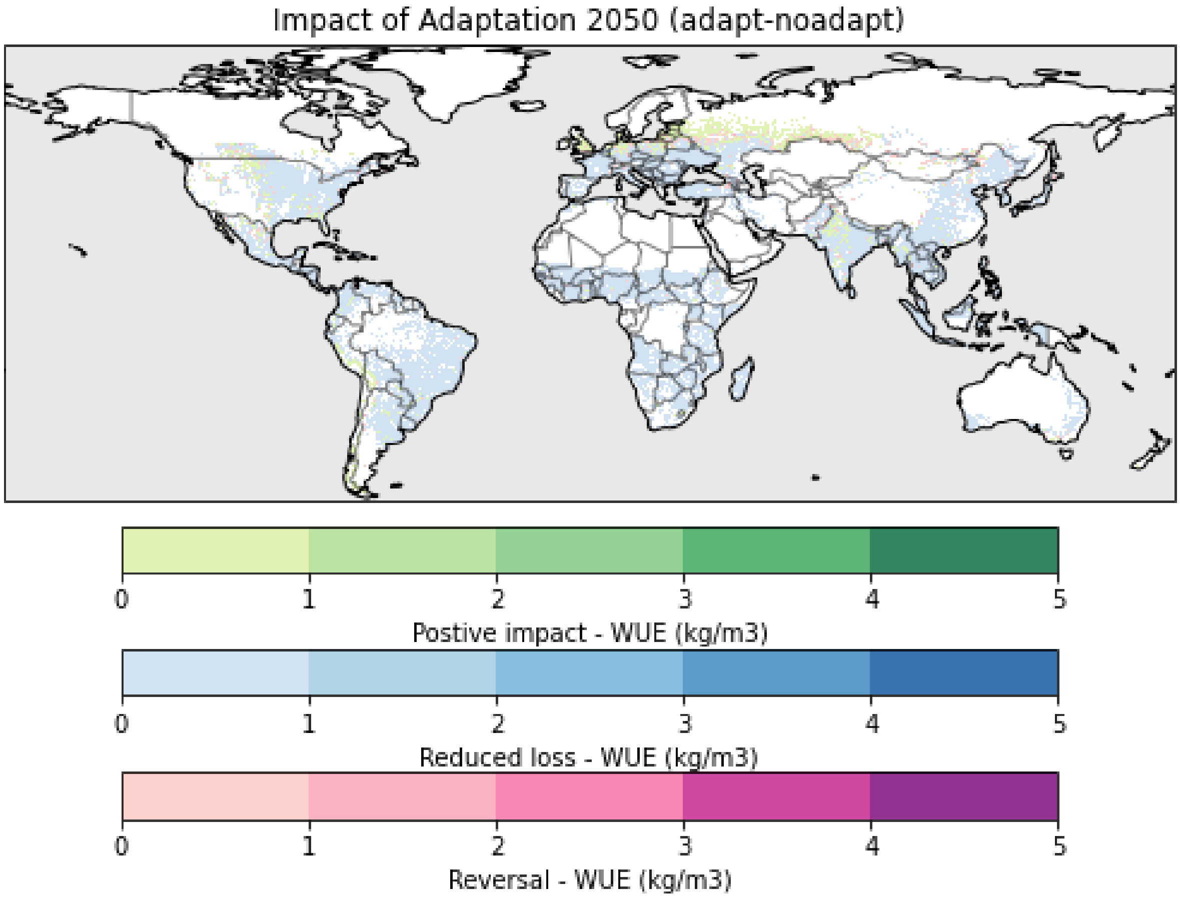

Figure S41: Global rainfed maize water use efficiency: Impact of adaptation on 2050 water use efficiency

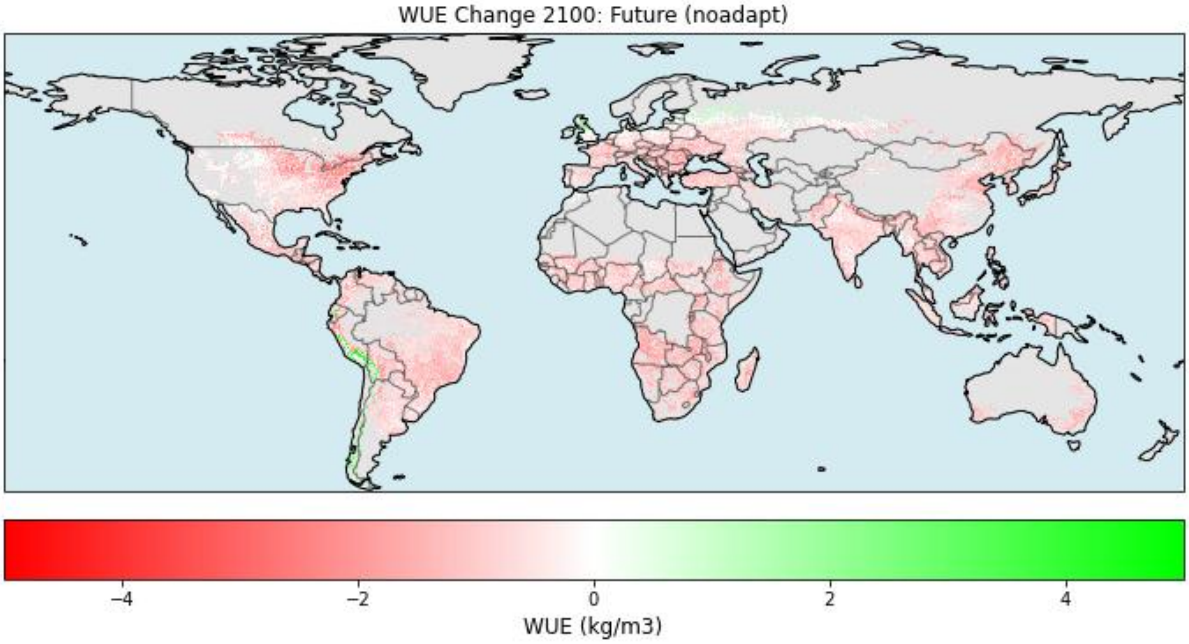

Figure S42: Global rainfed maize water use efficiency: Projected water use efficiency change by 2100 without adaptation

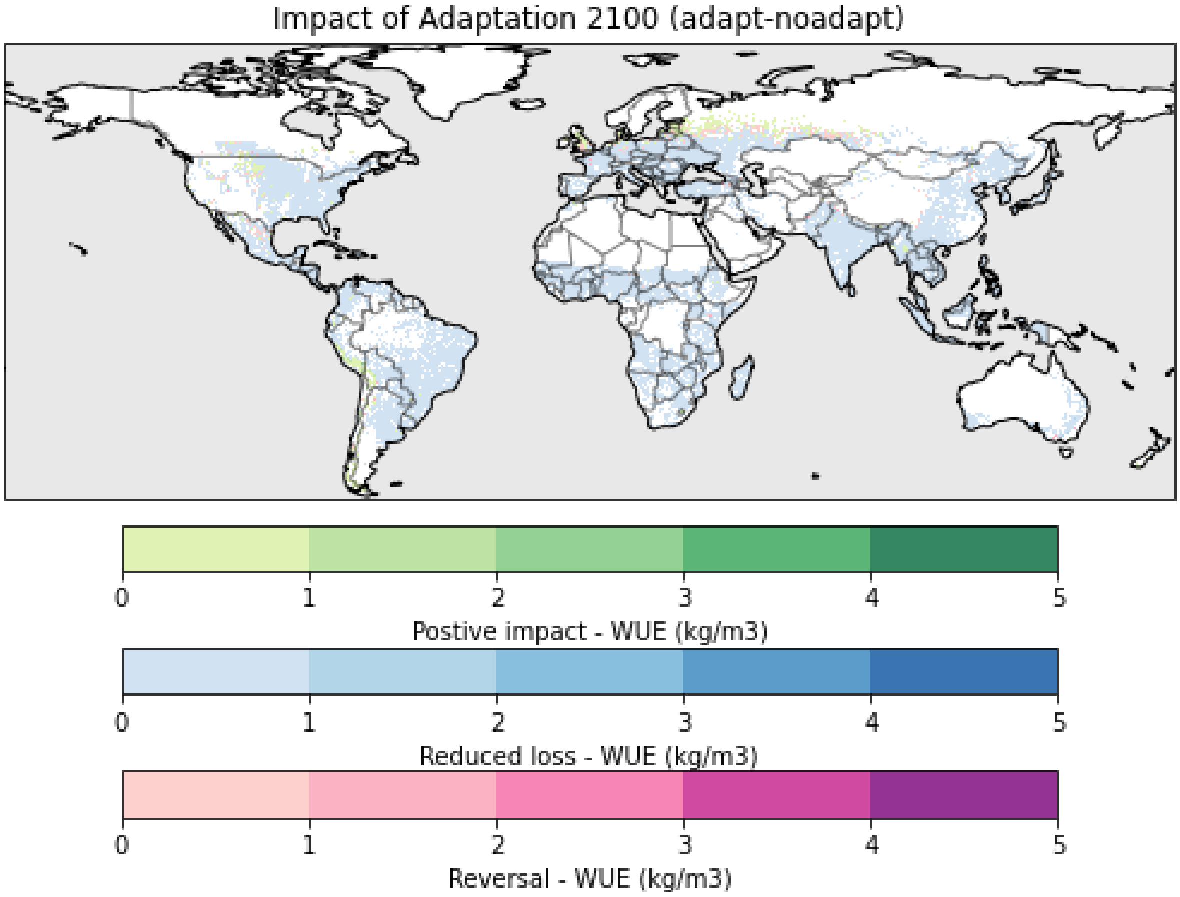

Figure S43: Global rainfed maize water use efficiency: Impact of adaptation on 2100 water use efficiency

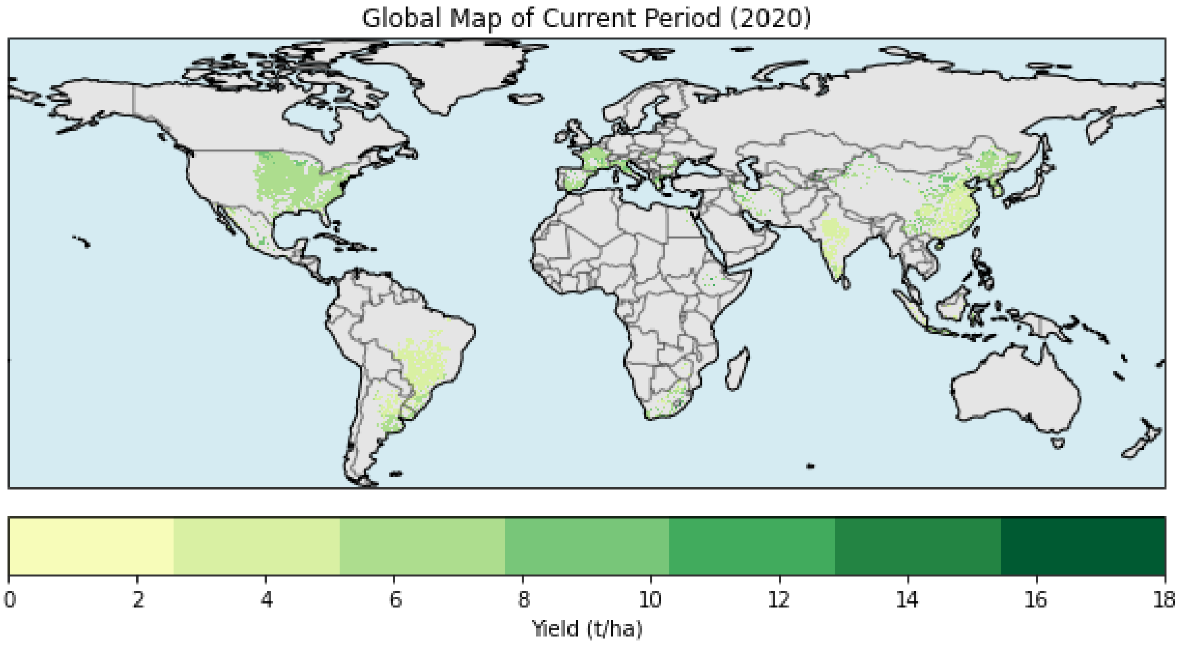

333

334 **Figure S44: Global irrigated soybean productivity: Baseline yield in 2020**

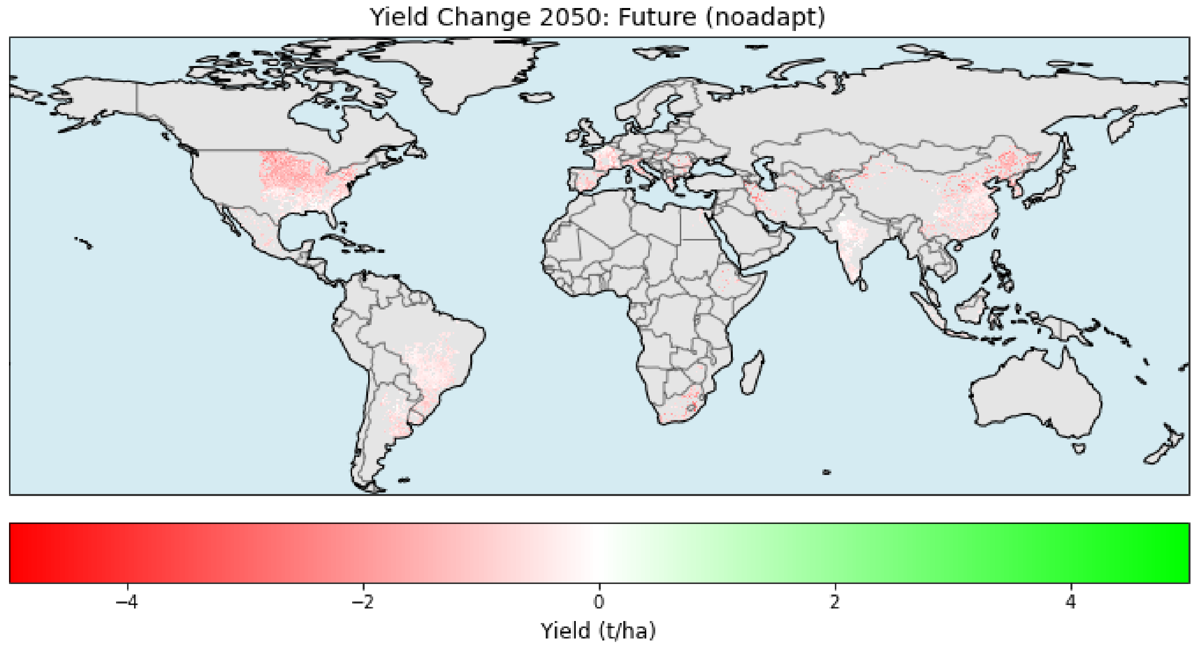

335

336 **Figure S45: Global irrigated soybean productivity: Projected yield change by 2050 without**
337 **adaptation**

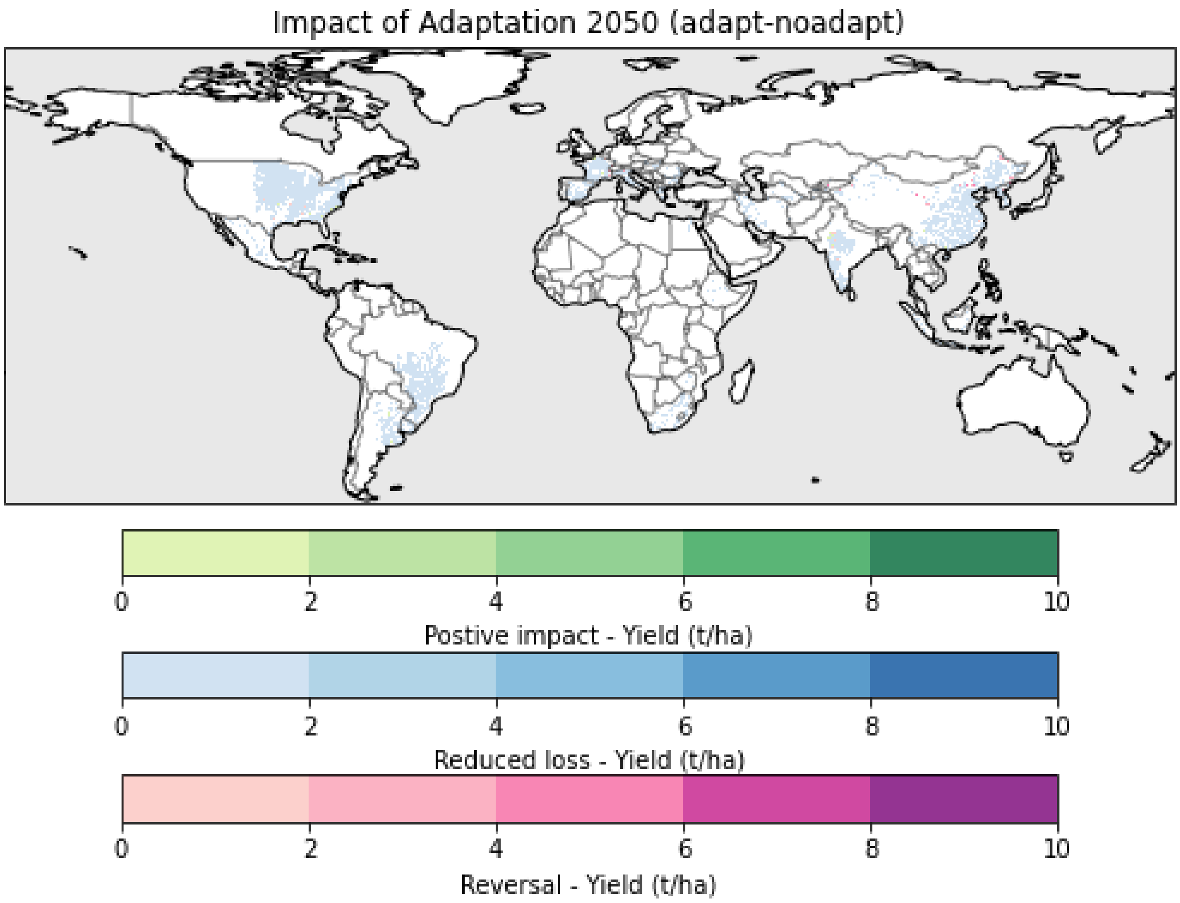

Figure S46: Global irrigated soybean productivity: Impact of adaptation on 2050 yields

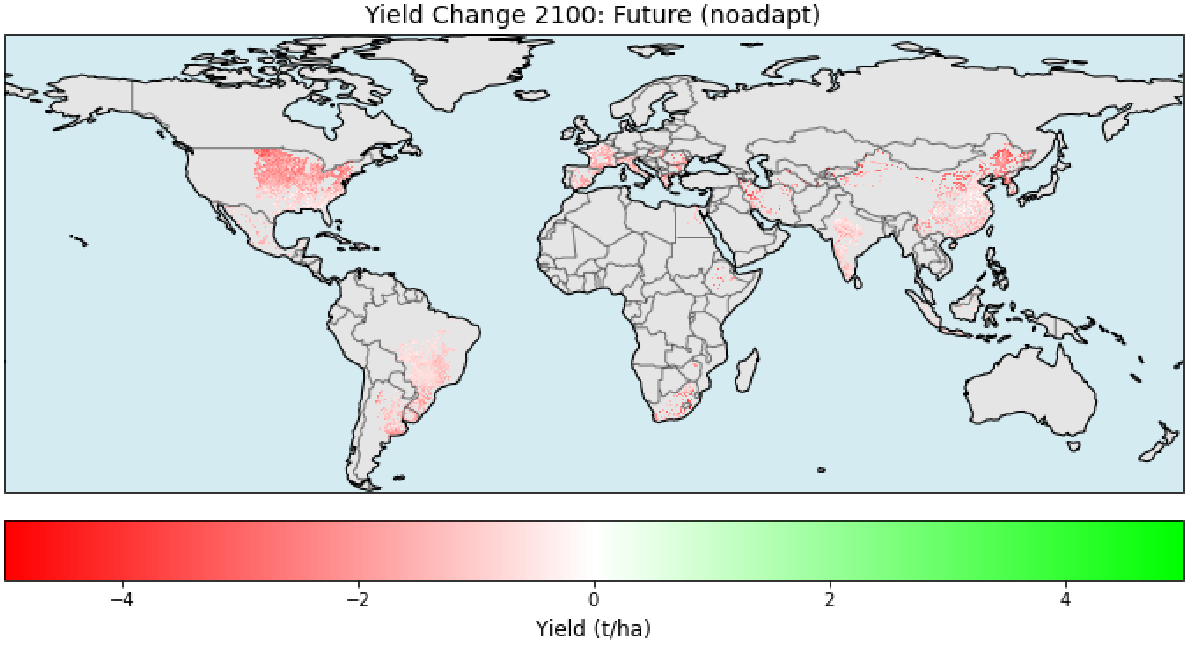

Figure S47: Global irrigated soybean productivity: Projected yield change by 2100 without adaptation

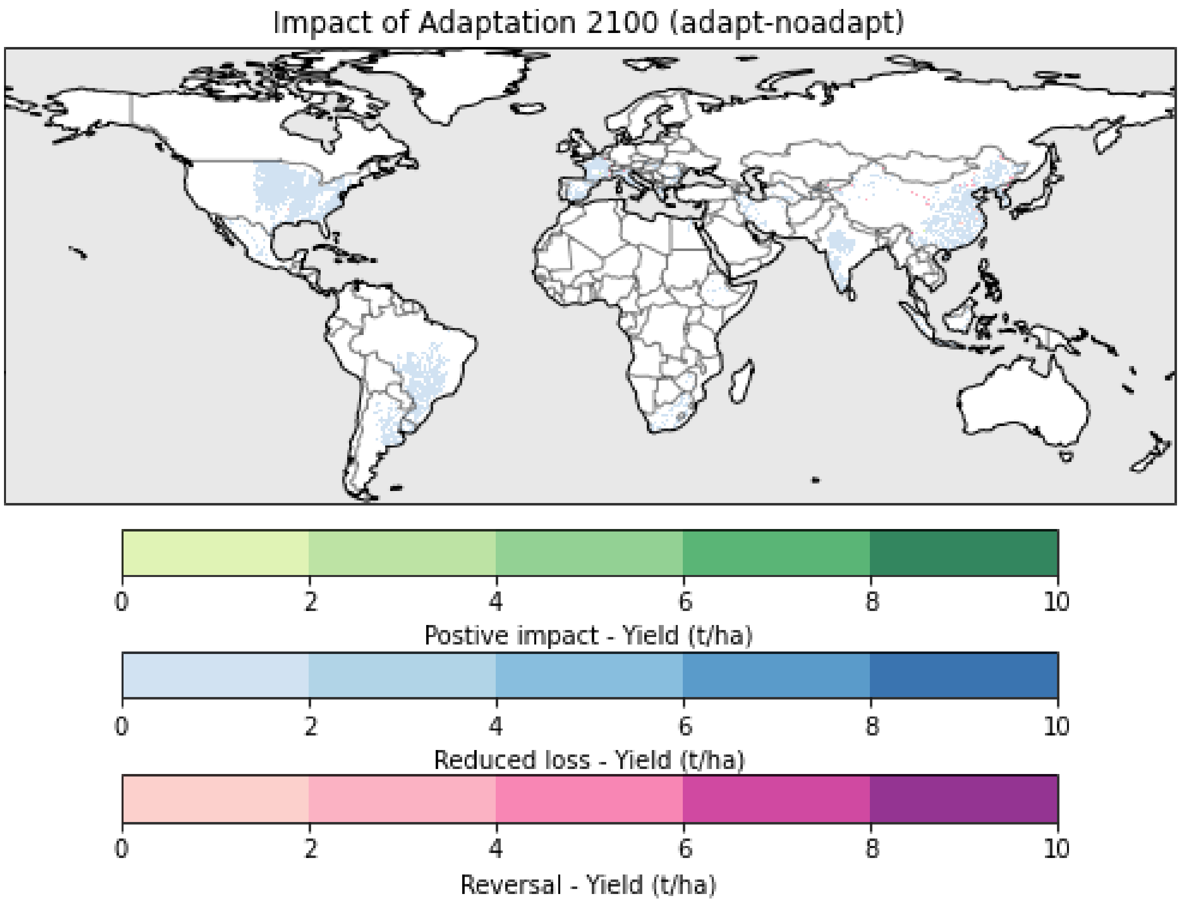

Figure S48: Global irrigated soybean productivity: Impact of adaptation on 2100 yields

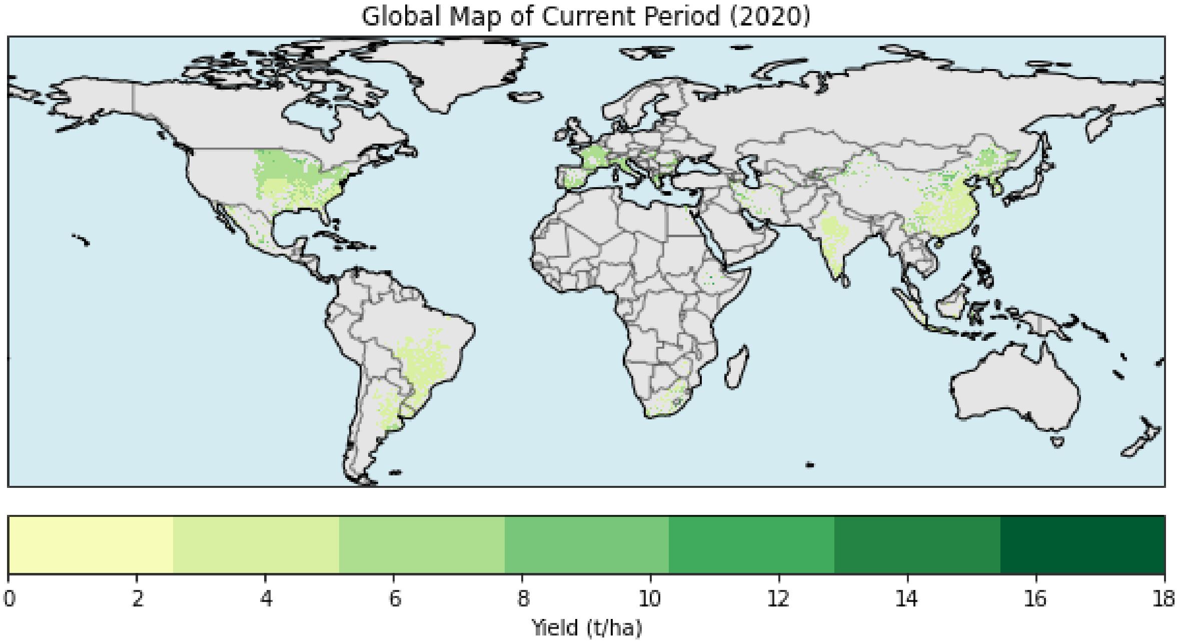

Figure S49: Global irrigated soybean reliability: Baseline yield in 2020

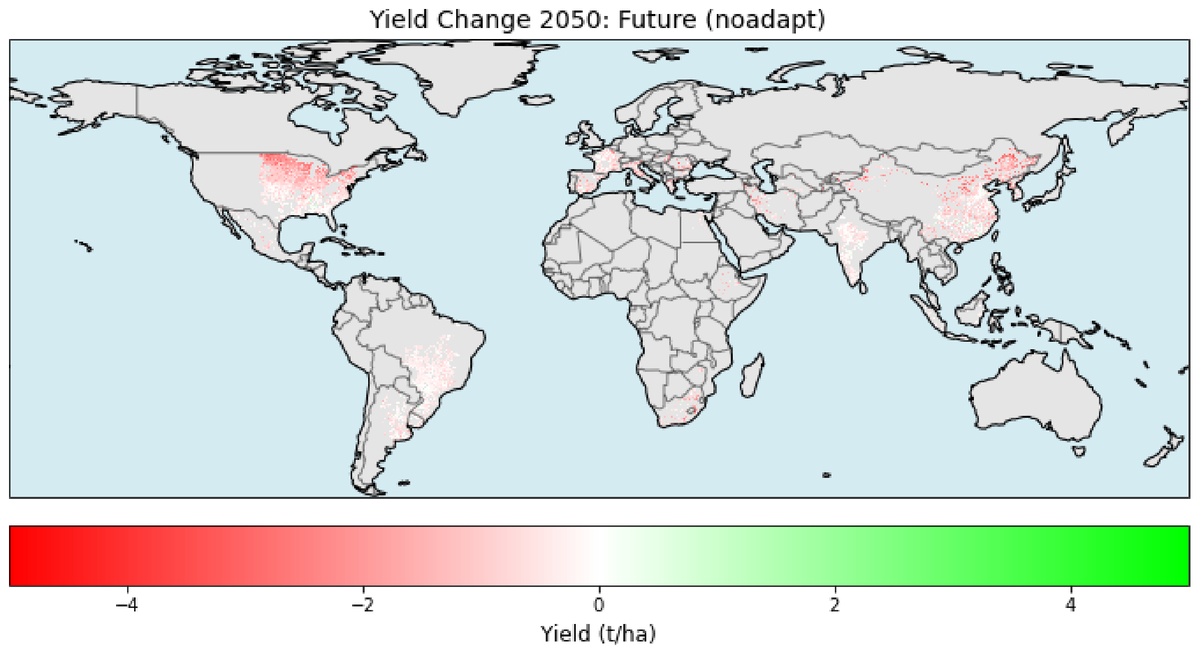

Figure S50: Global irrigated soybean reliability: Projected yield change by 2050 without adaptation

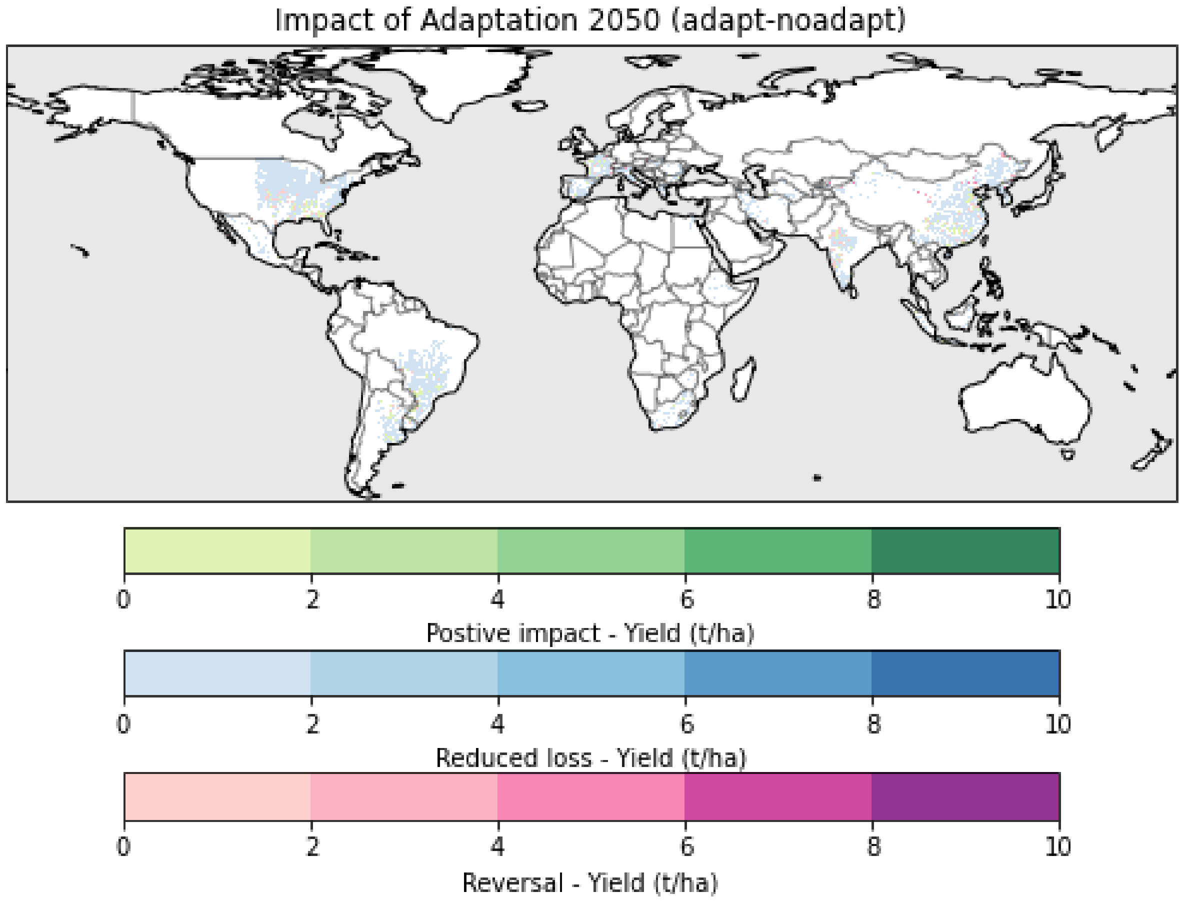

Figure S51: Global irrigated soybean reliability: Impact of adaptation on 2050 yields

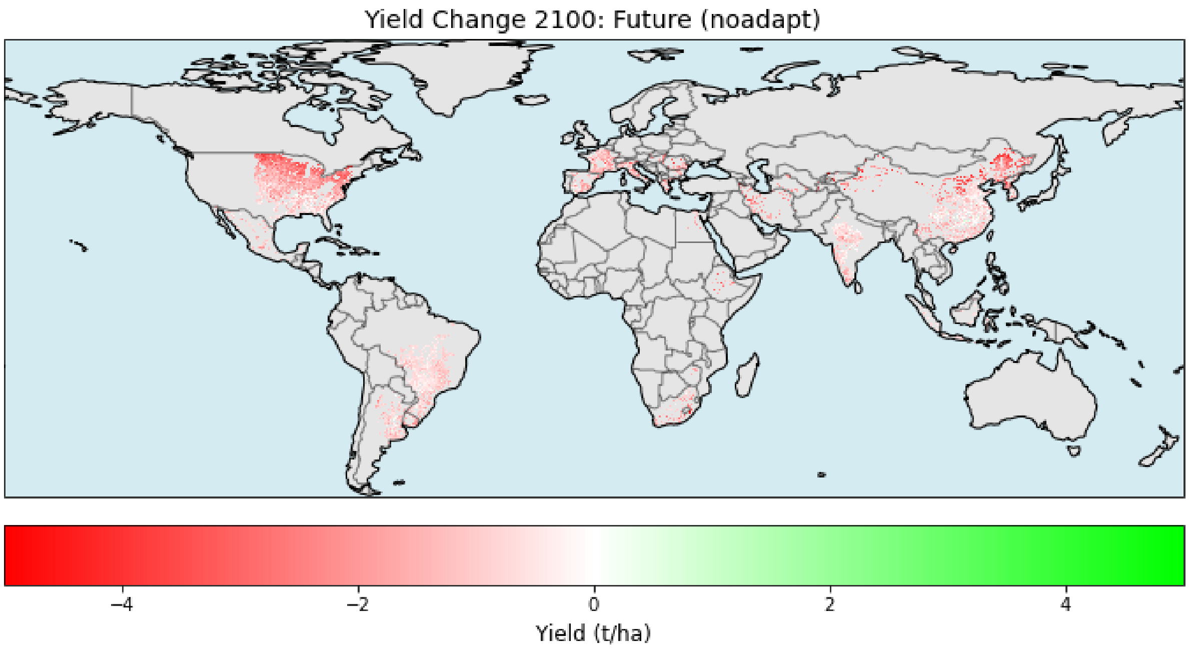

Figure S52: Global irrigated soybean reliability: Projected yield change by 2100 without adaptation

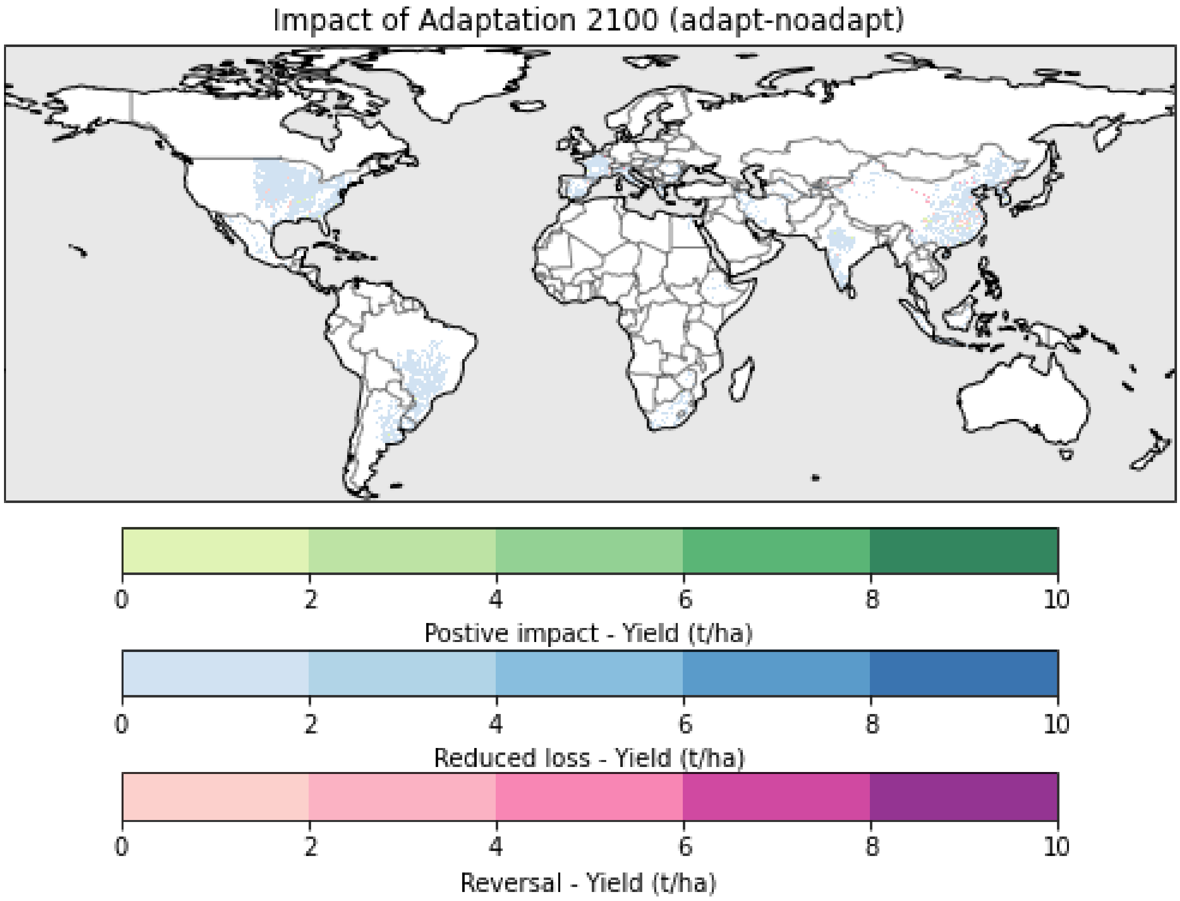

Figure S53: Global irrigated soybean reliability: Impact of adaptation on 2100 yields

357

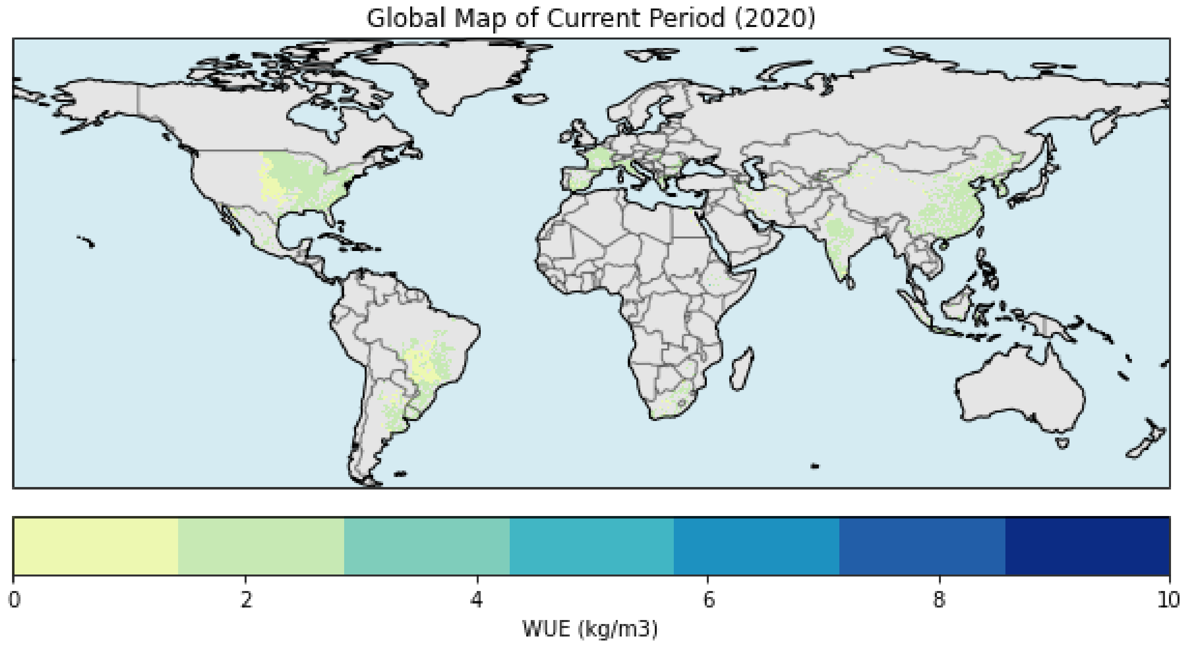

358 **Figure S4: Global irrigated soybean water use efficiency: Baseline water use efficiency in 2020**

359

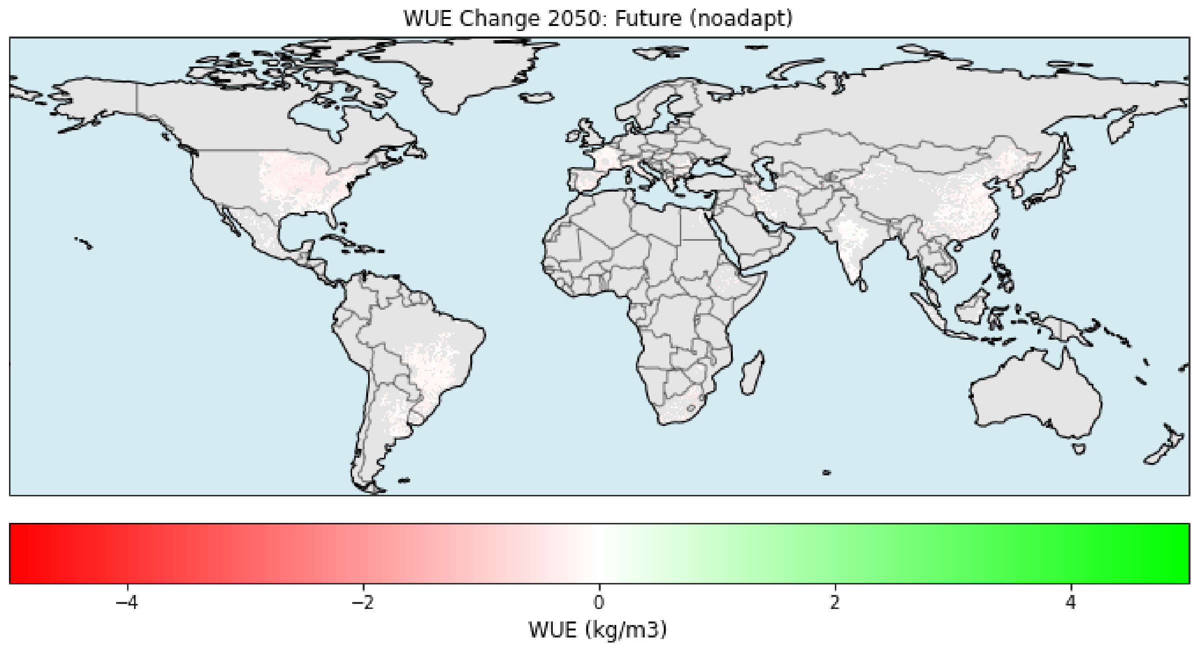

360 **Figure S55: Global irrigated soybean water use efficiency: Projected water use efficiency**
361 **change by 2050 without adaptation**

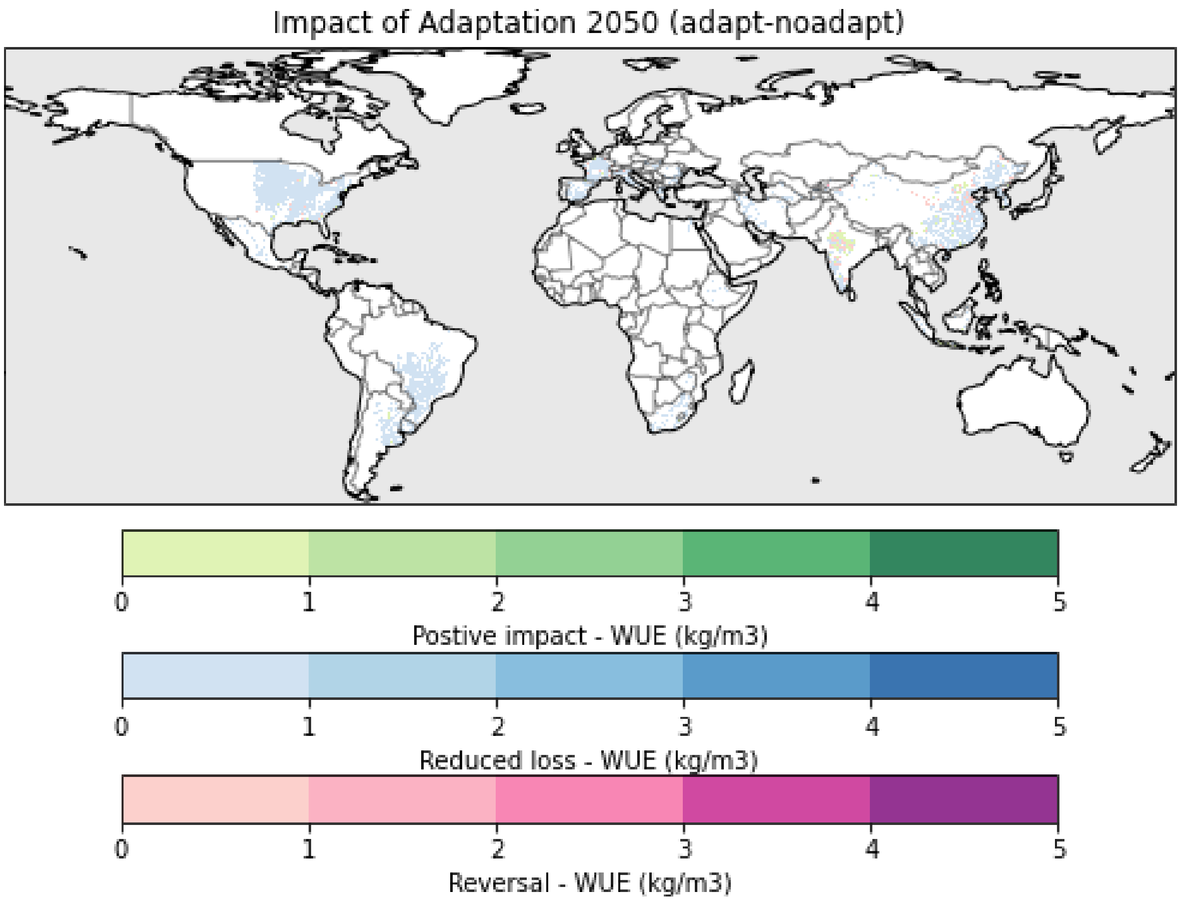

Figure S56: Global irrigated soybean water use efficiency: Impact of adaptation on 2050 water use efficiency

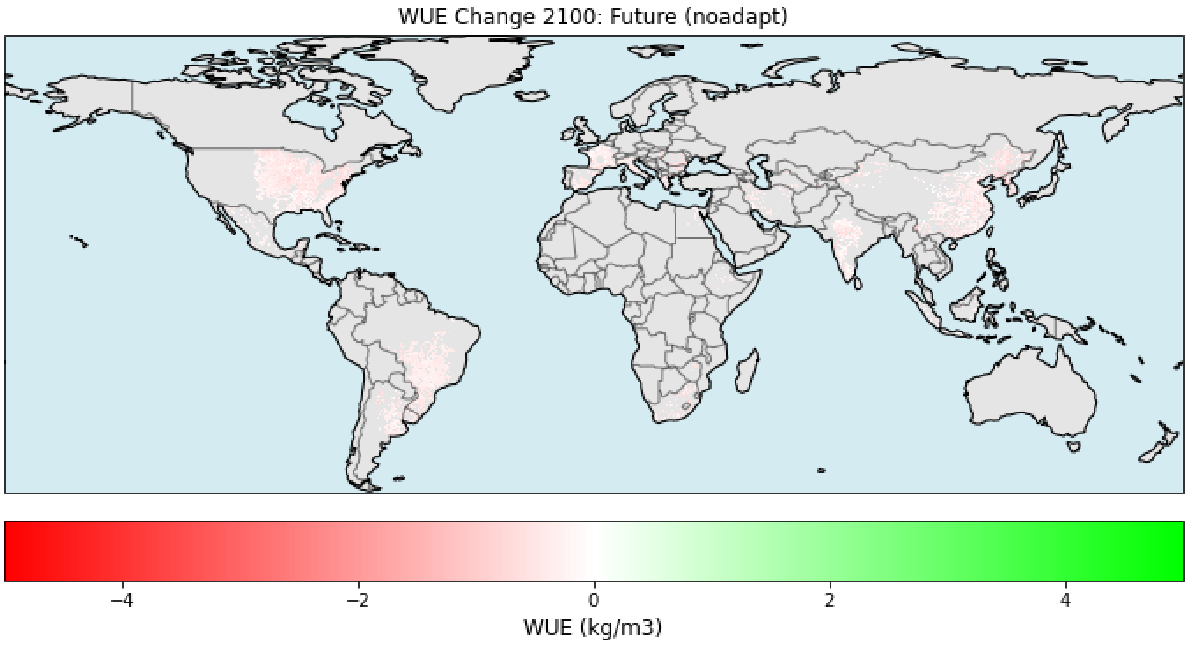

Figure S57: Global irrigated soybean water use efficiency: Projected water use efficiency change by 2100 without adaptation

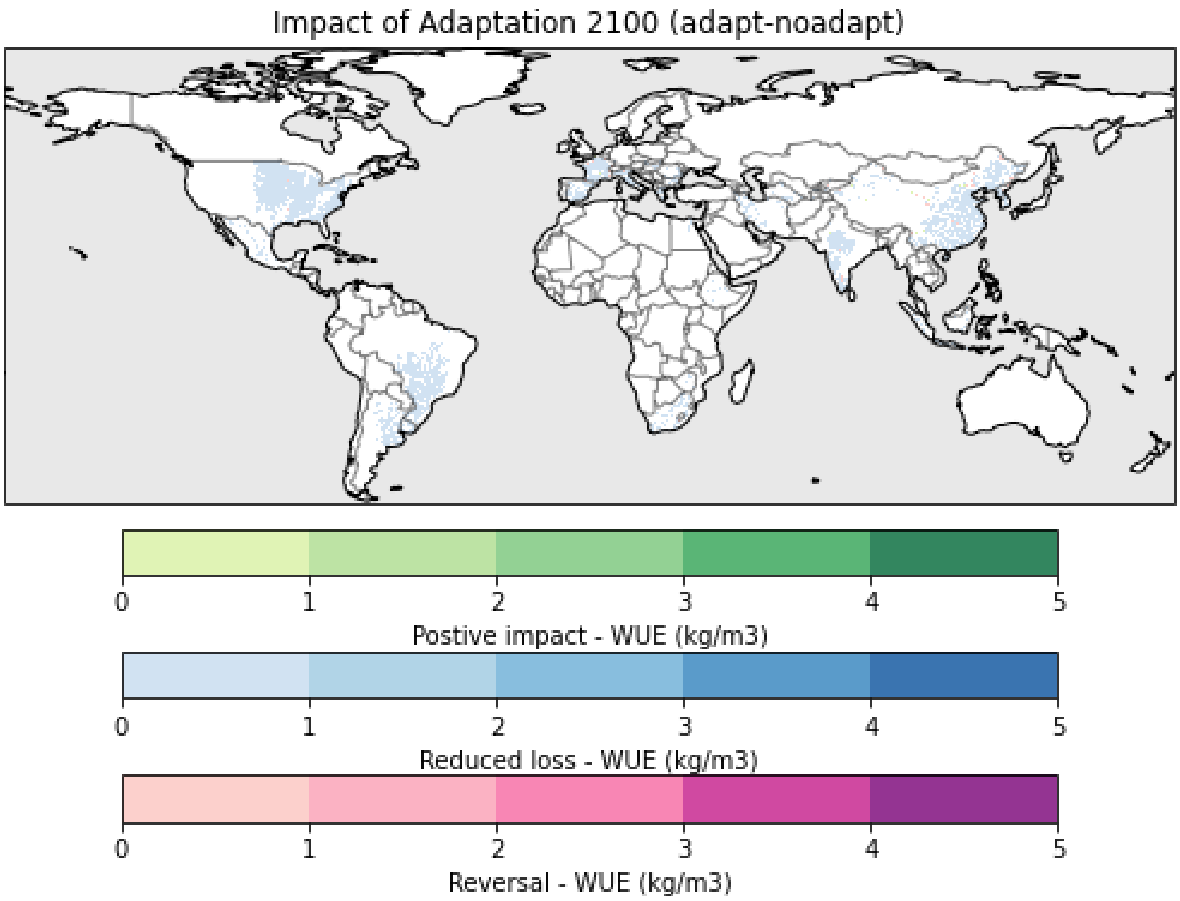

Figure S58: Global irrigated soybean water use efficiency: Impact of adaptation on 2100 water use efficiency

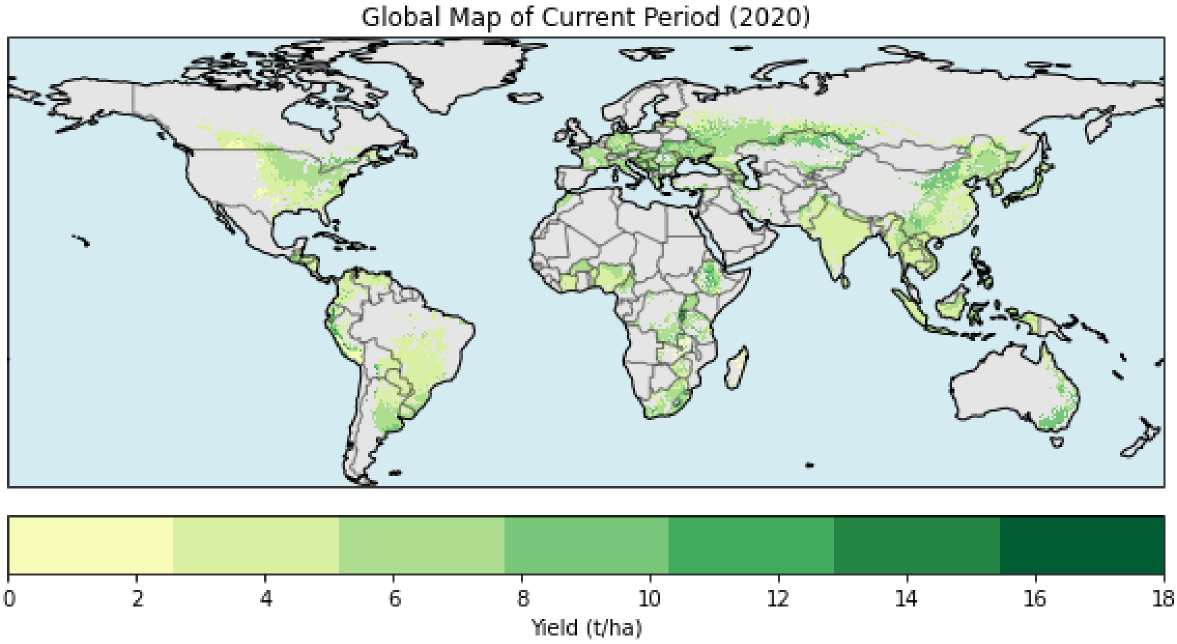

Figure S59: Global rainfed soybean productivity: Baseline yield in 2020

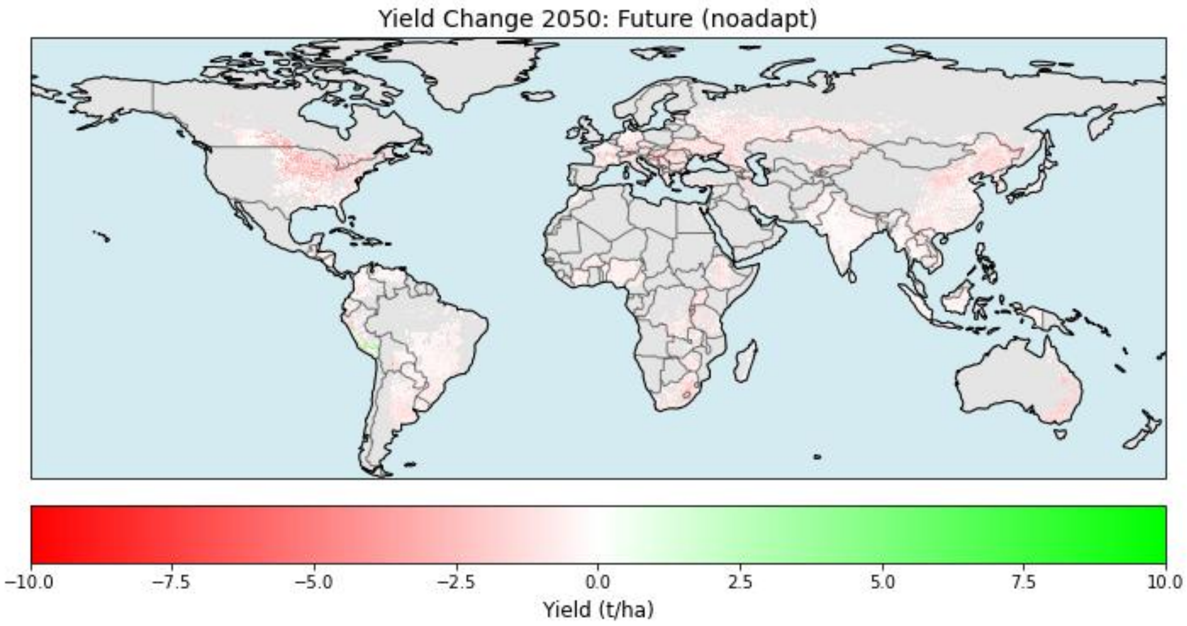

Figure S60: Global rainfed soybean productivity: Projected yield change by 2050 without adaptation

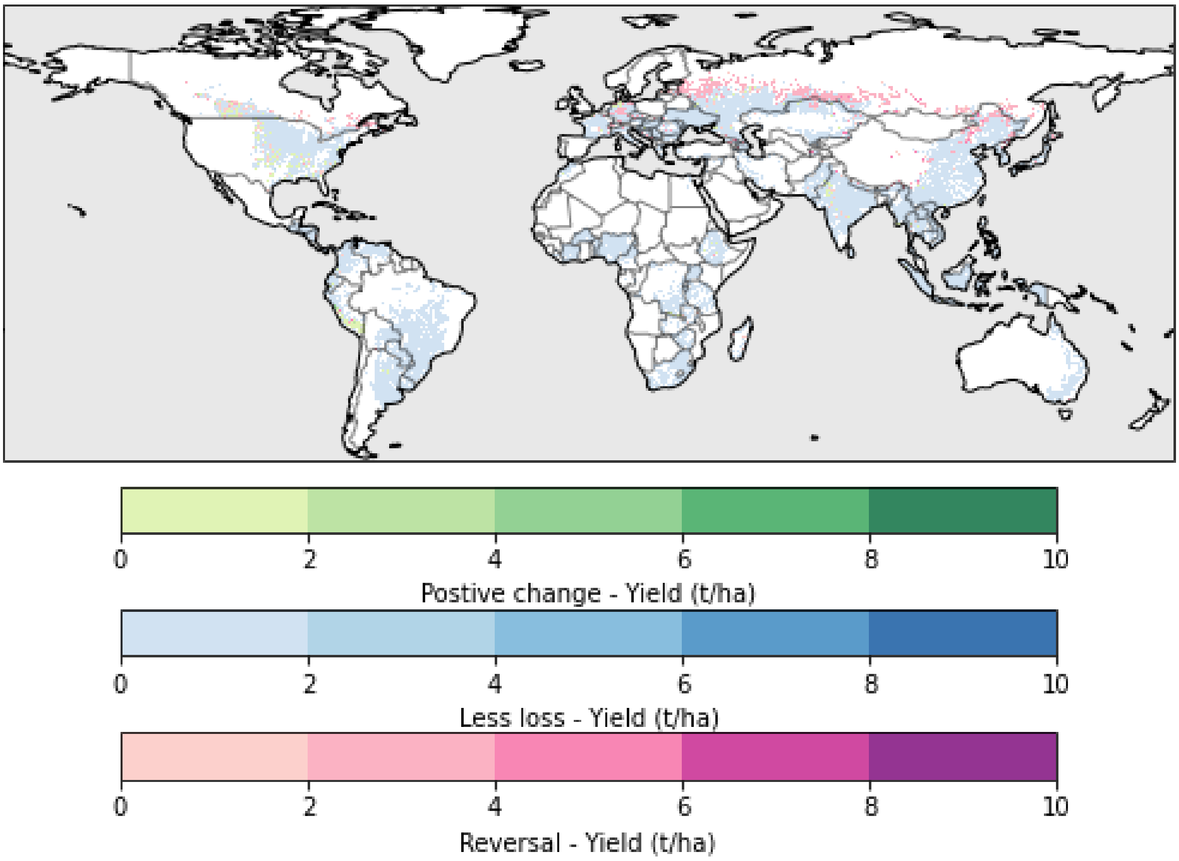

Figure S61: Global rainfed soybean productivity: Impact of adaptation on 2050 yields

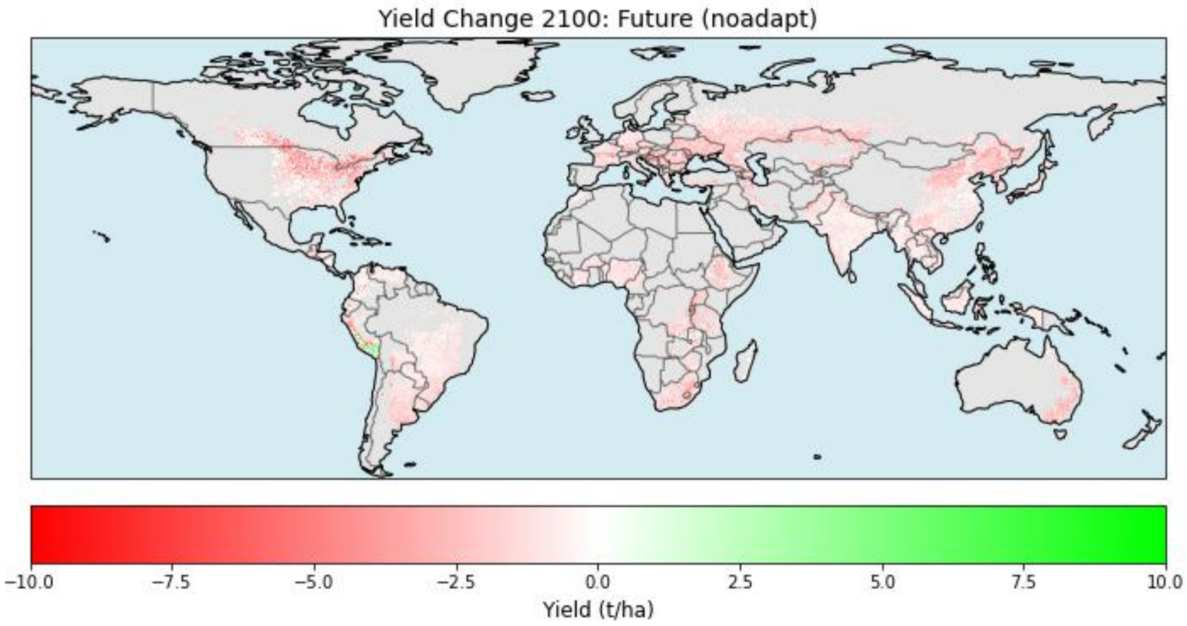

Figure S62: Global rainfed soybean productivity: Projected yield change by 2100 without adaptation

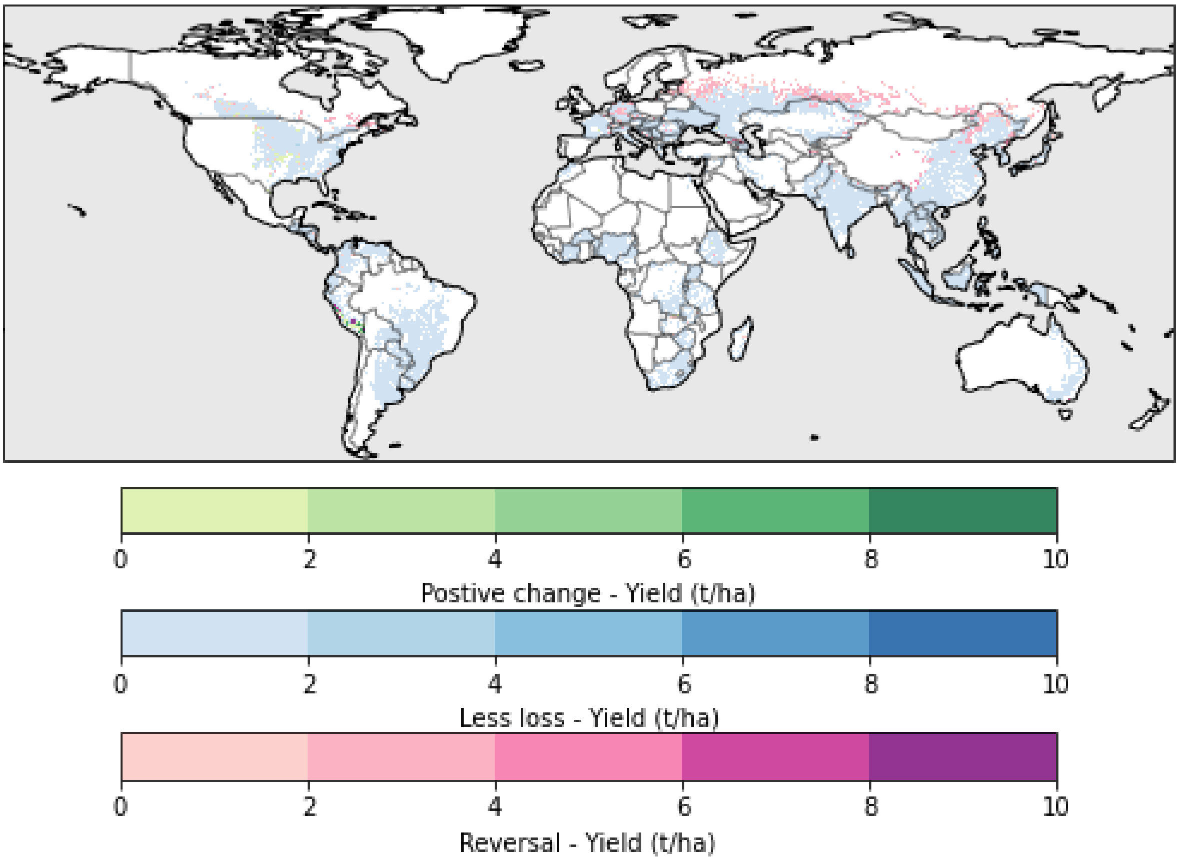

Figure S63: Global rainfed soybean productivity: Impact of adaptation on 2100 yields

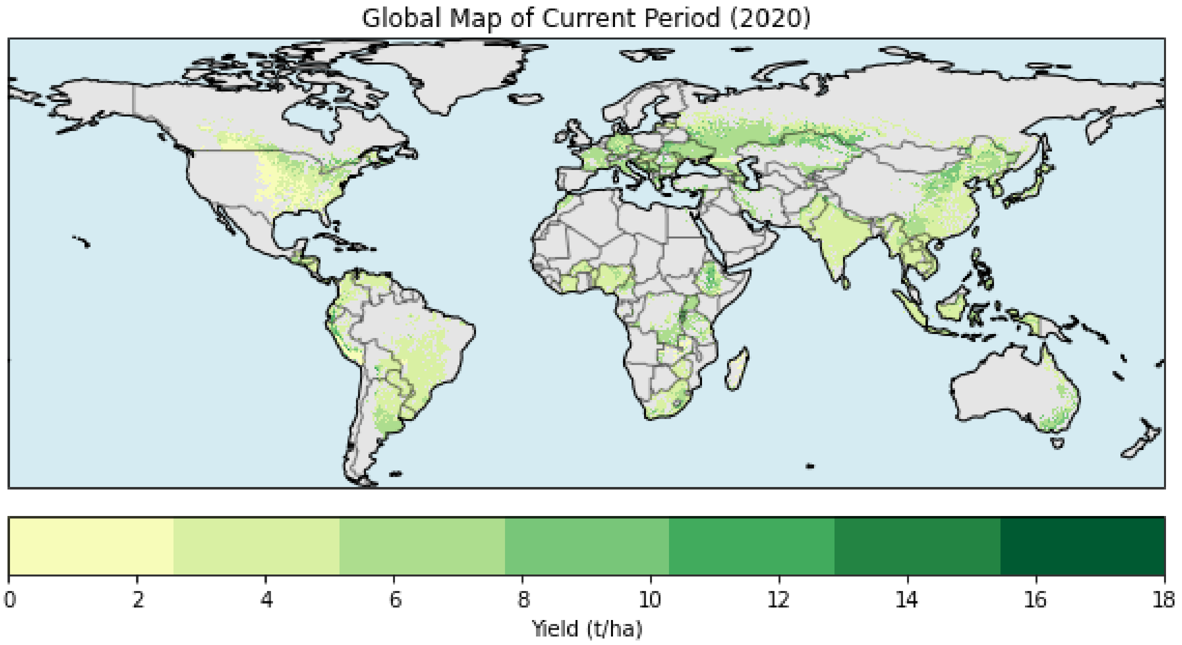

Figure S64: Global rainfed soybean reliability: Baseline yield in 2020

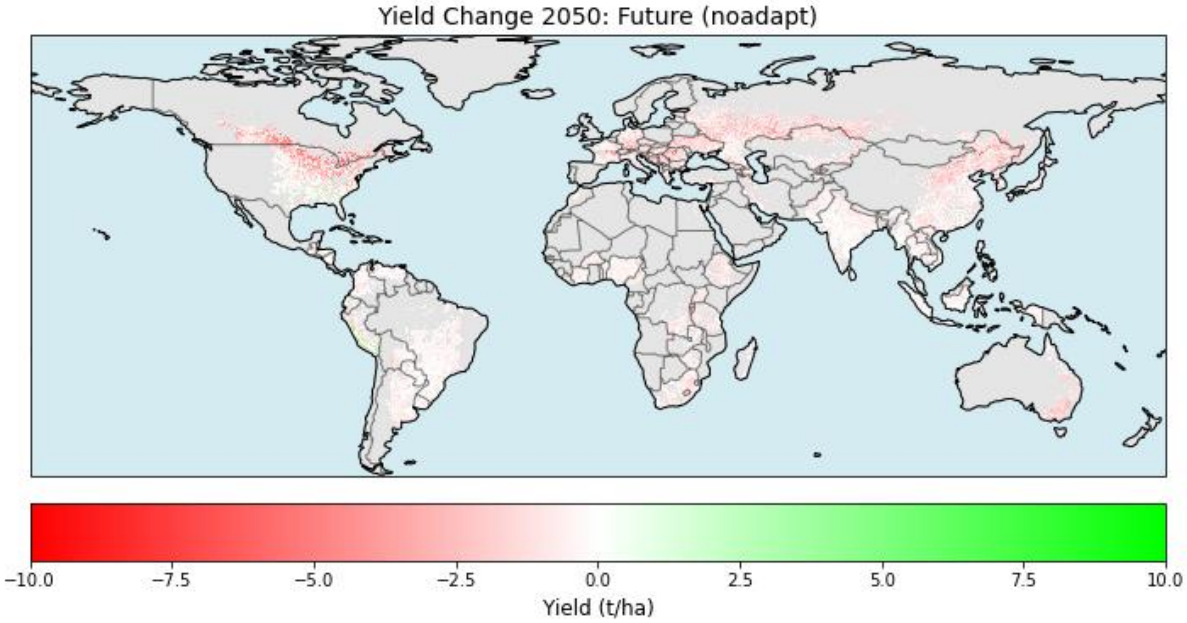

Figure S65: Global rainfed soybean reliability: Projected yield change by 2050 without adaptation

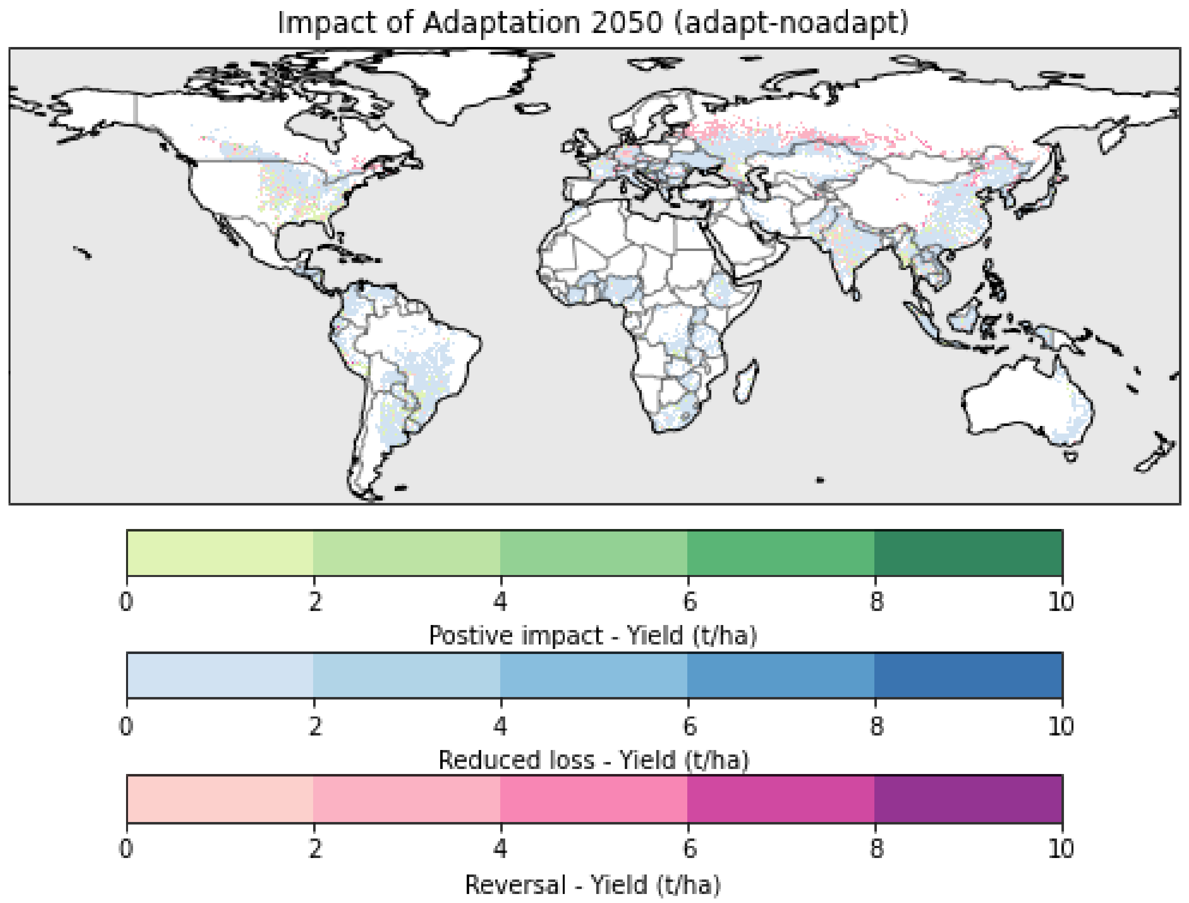

Figure S66: Global rainfed soybean reliability: Impact of adaptation on 2050 yields

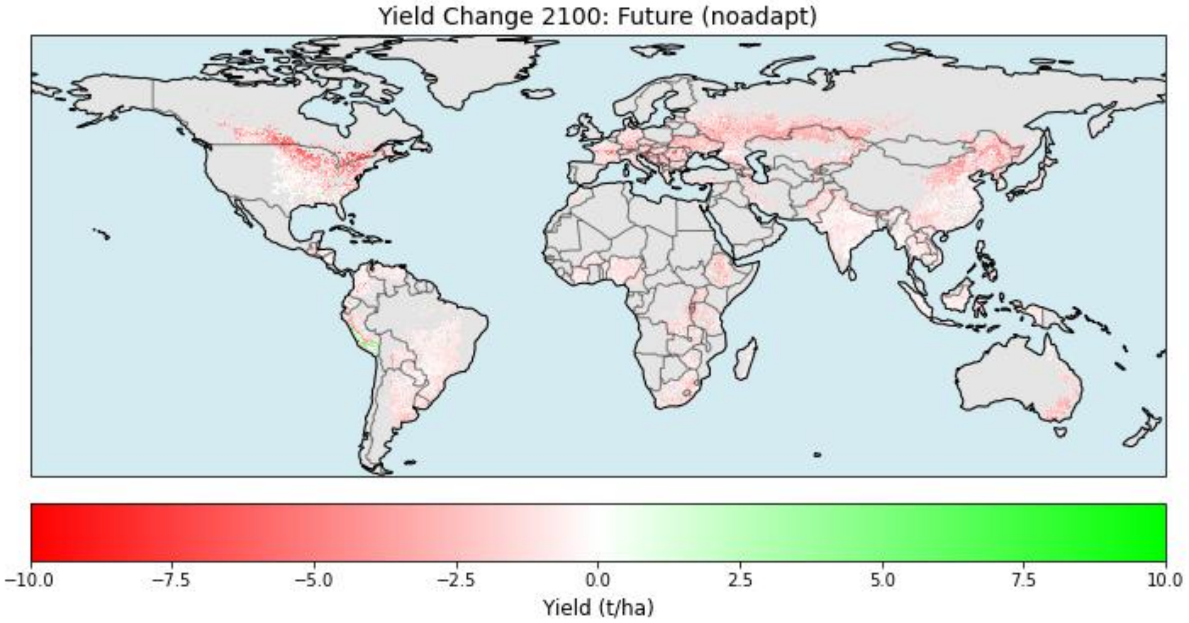

Figure S67: Global rainfed soybean reliability: Projected yield change by 2100 without adaptation

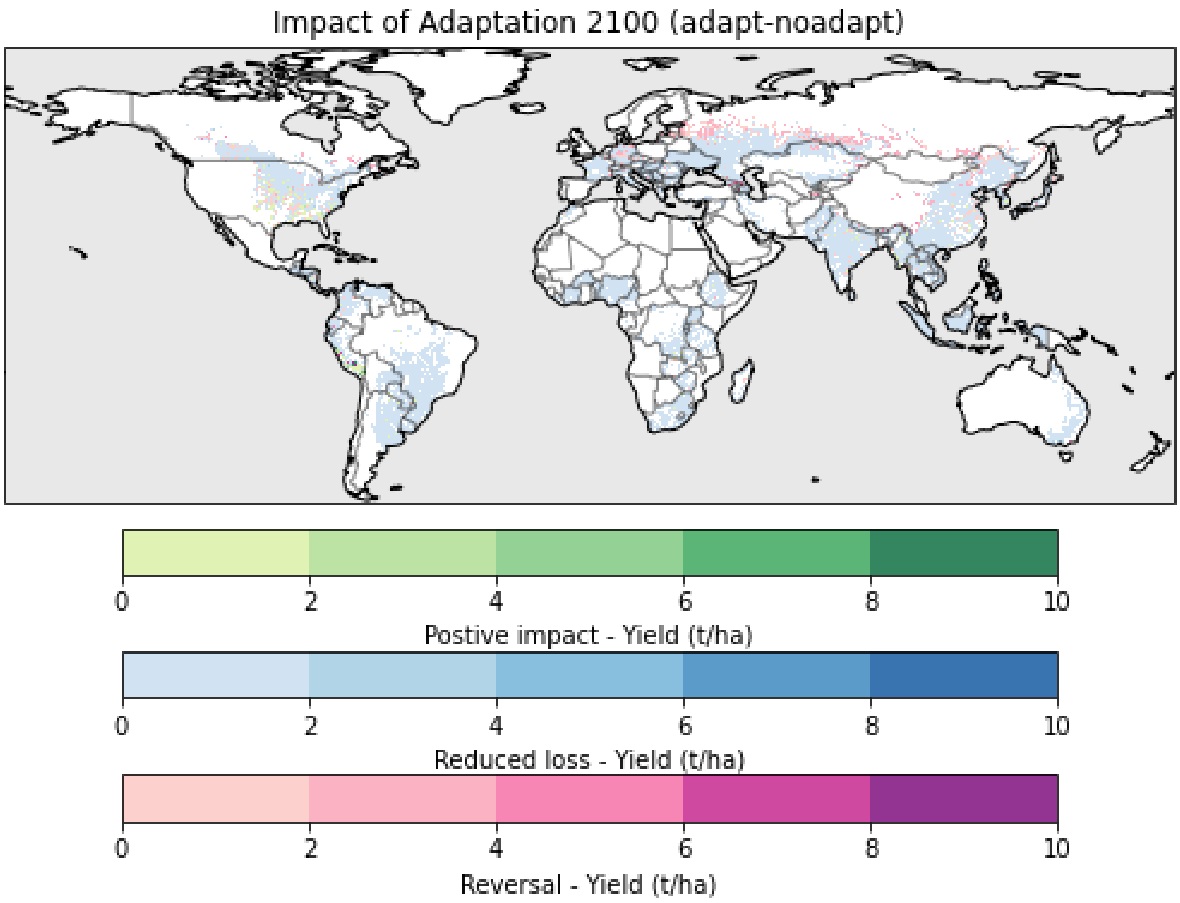

Figure S68: Global rainfed soybean reliability: Impact of adaptation on 2100 yields

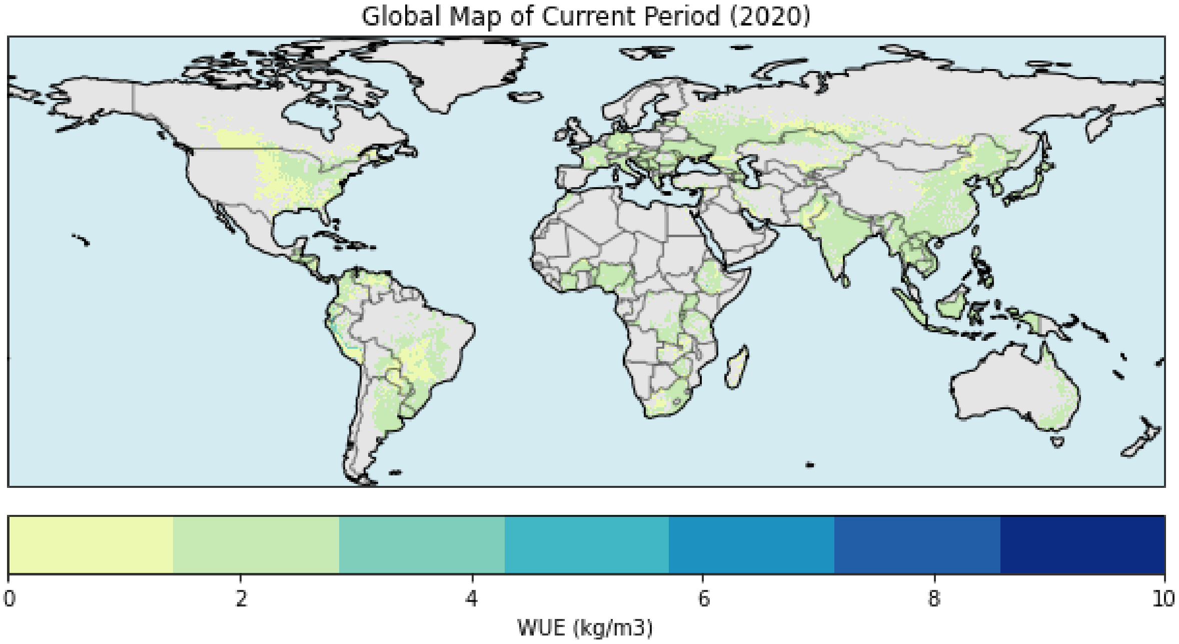

Figure S69: Global rainfed soybean water use efficiency: Baseline water use efficiency in 2020

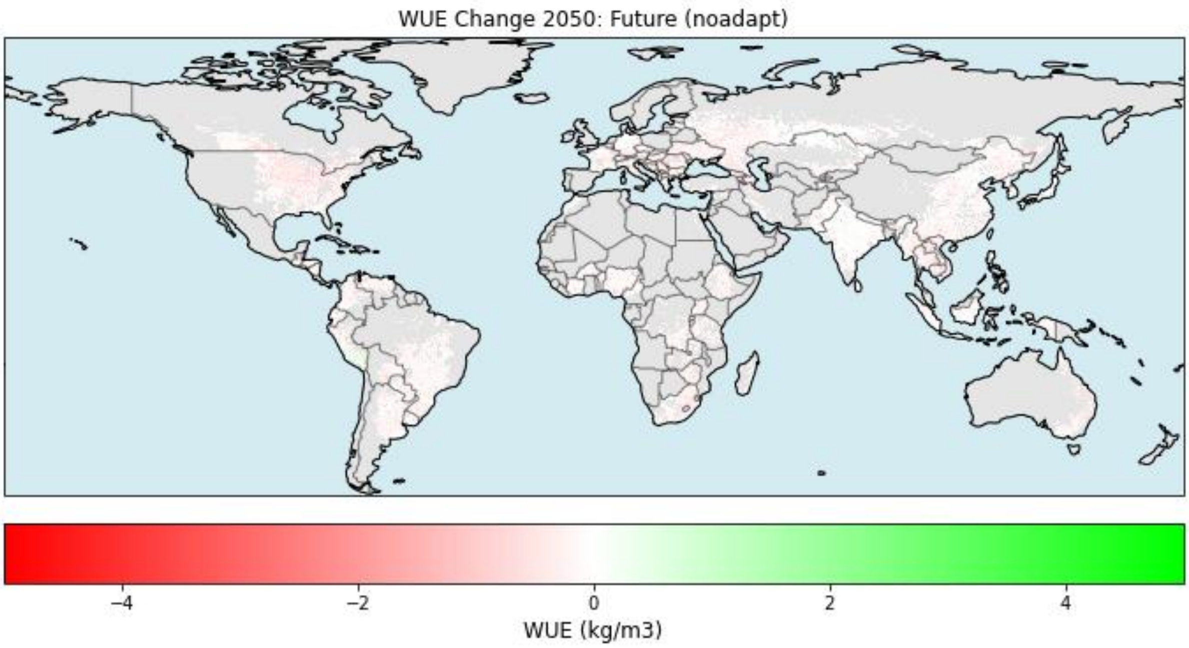

Figure S70: Global rainfed soybean water use efficiency: Projected water use efficiency change by 2050 without adaptation

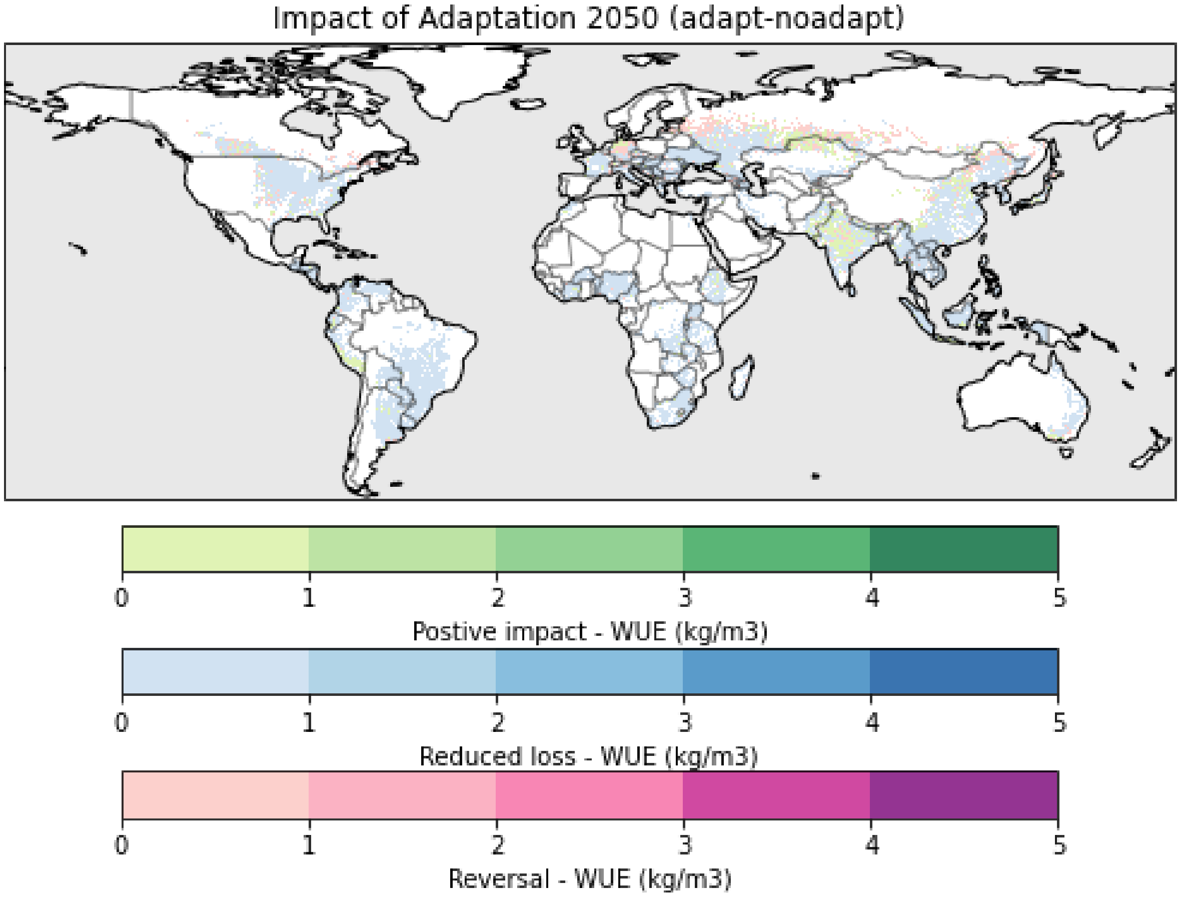

Figure S71: Global rainfed soybean water use efficiency: Impact of adaptation on 2050 water use efficiency

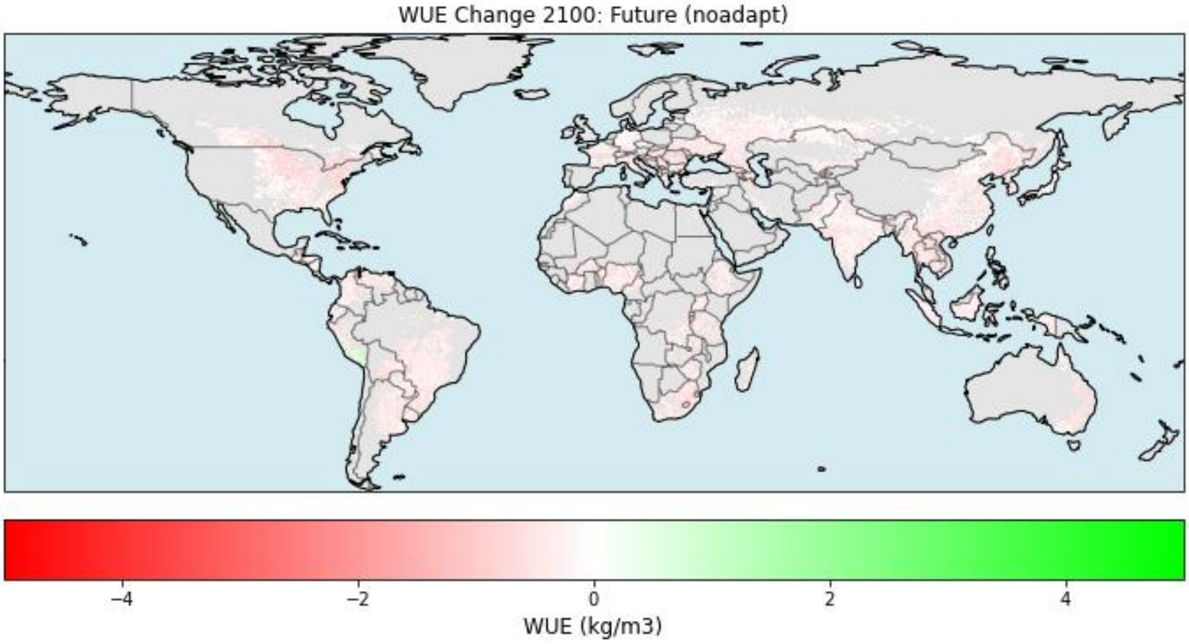

Figure S72: Global rainfed soybean water use efficiency: Projected water use efficiency change by 2100 without adaptation

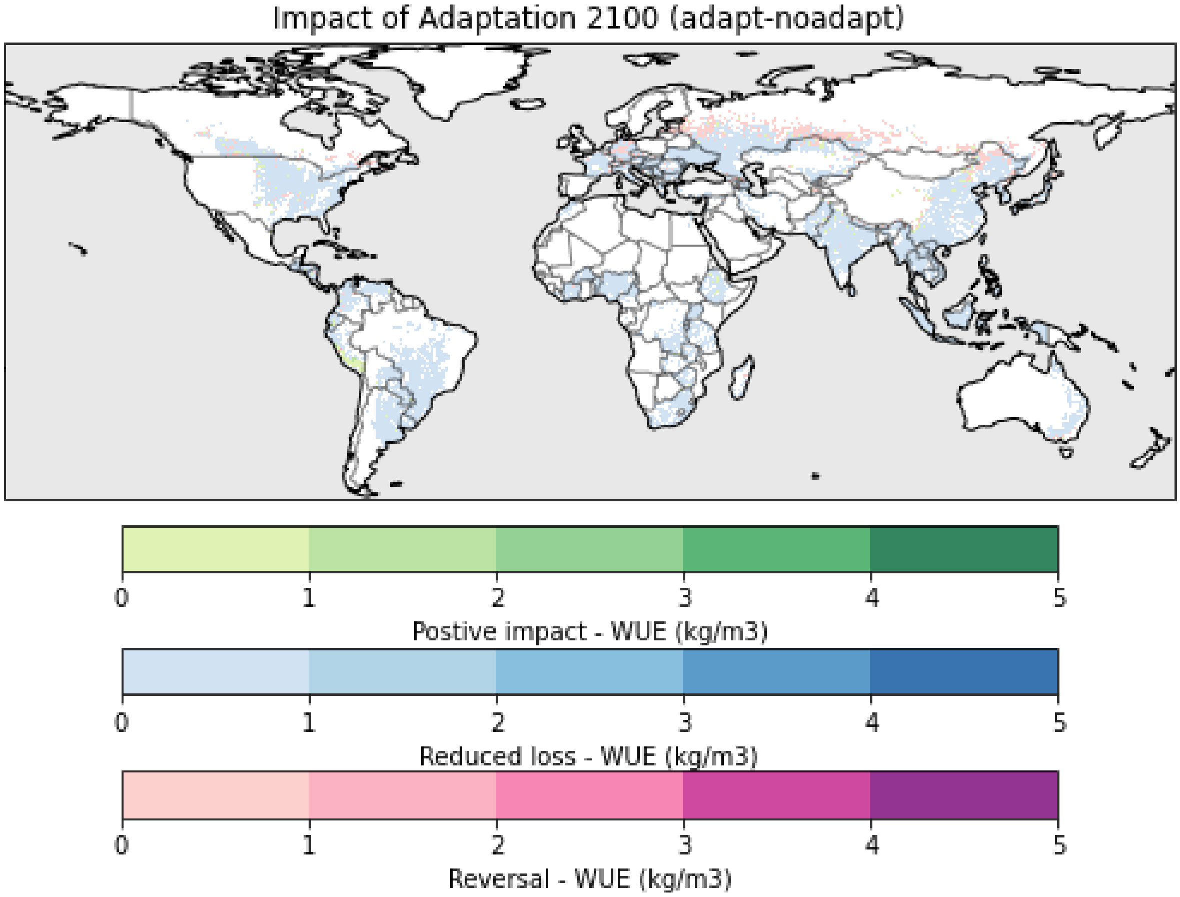

Figure S73: Global rainfed soybean water use efficiency: Impact of adaptation on 2100 water use efficiency

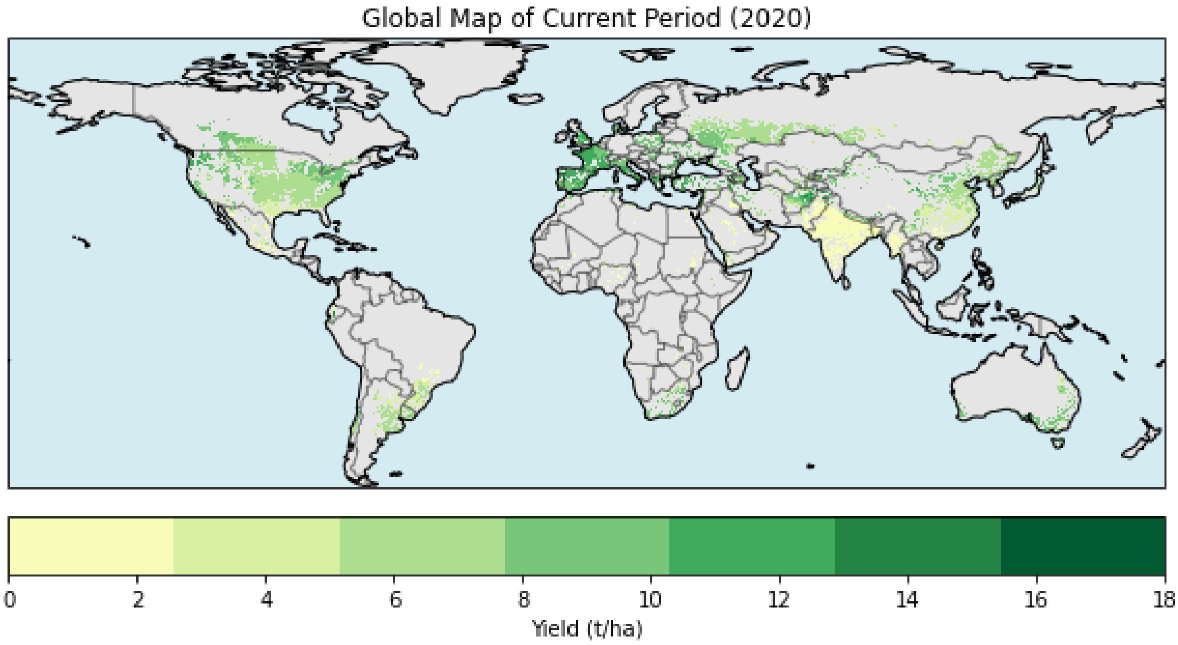

Figure S74: Global irrigated winter wheat productivity: Baseline yield in 2020

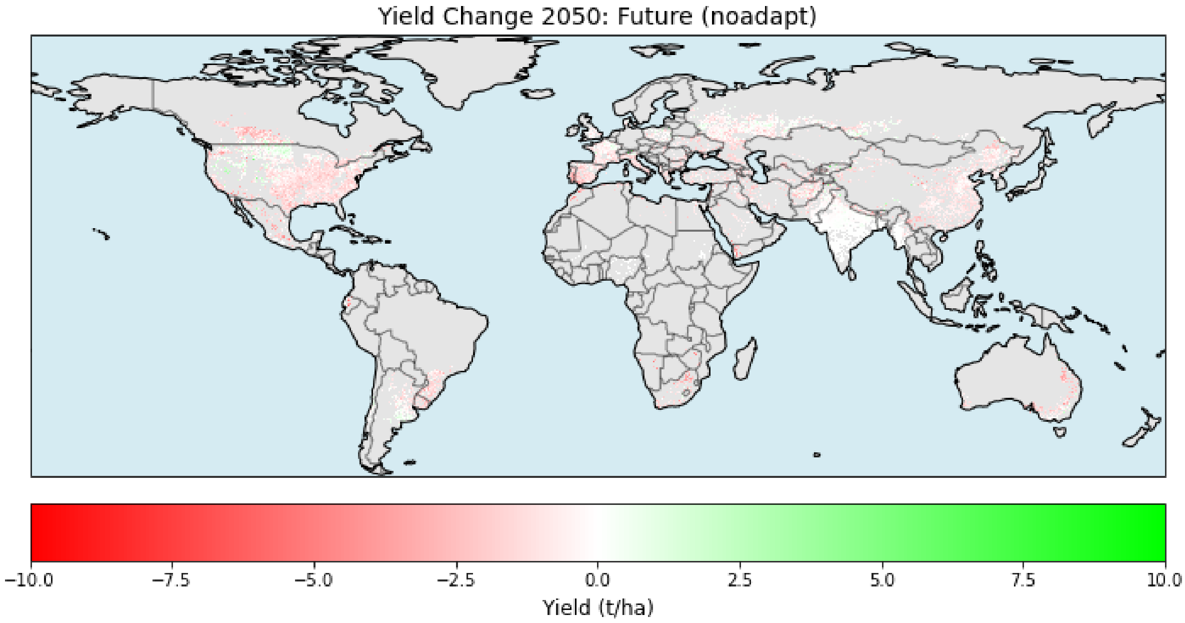

Figure S75: Global irrigated winter wheat productivity: Projected yield change by 2050 without adaptation

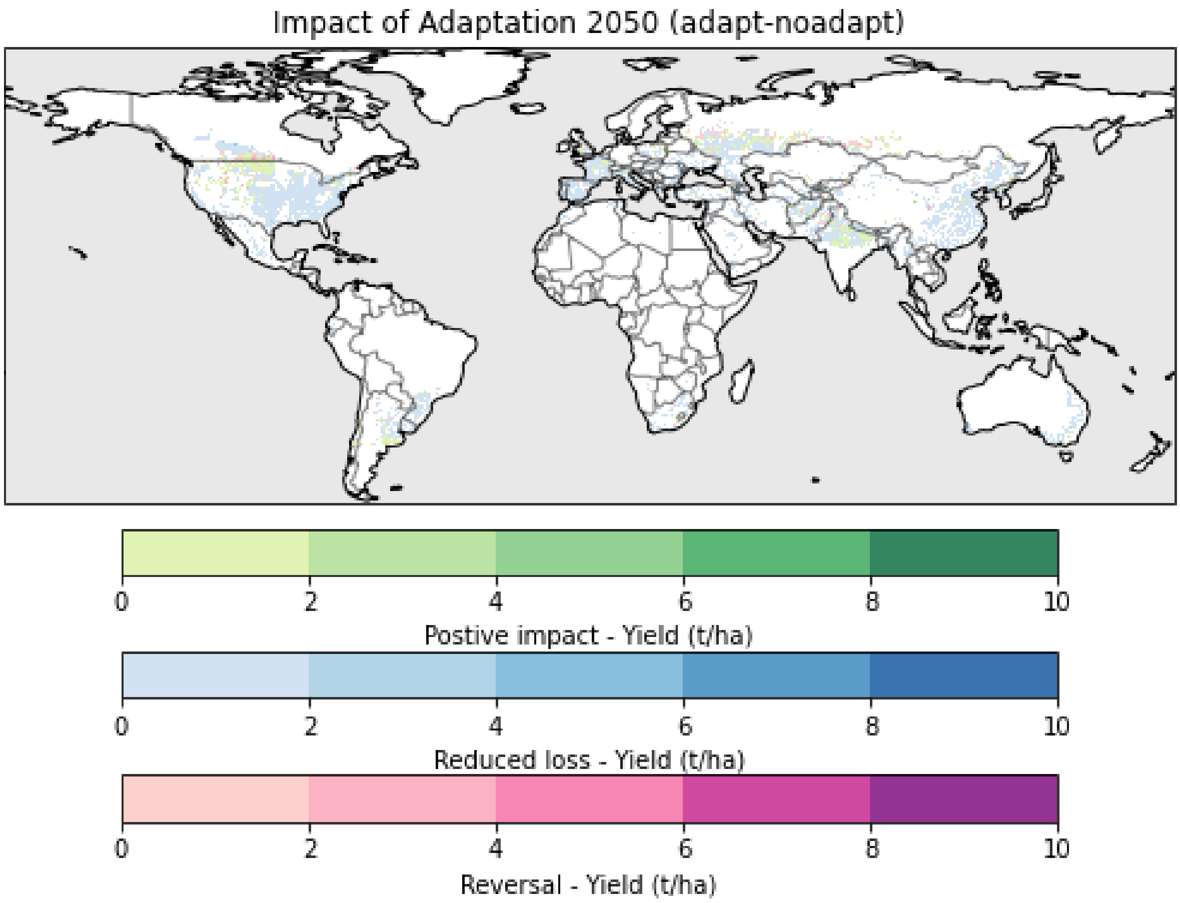

Figure S76: Global irrigated winter wheat productivity: Impact of adaptation on 2050 yields

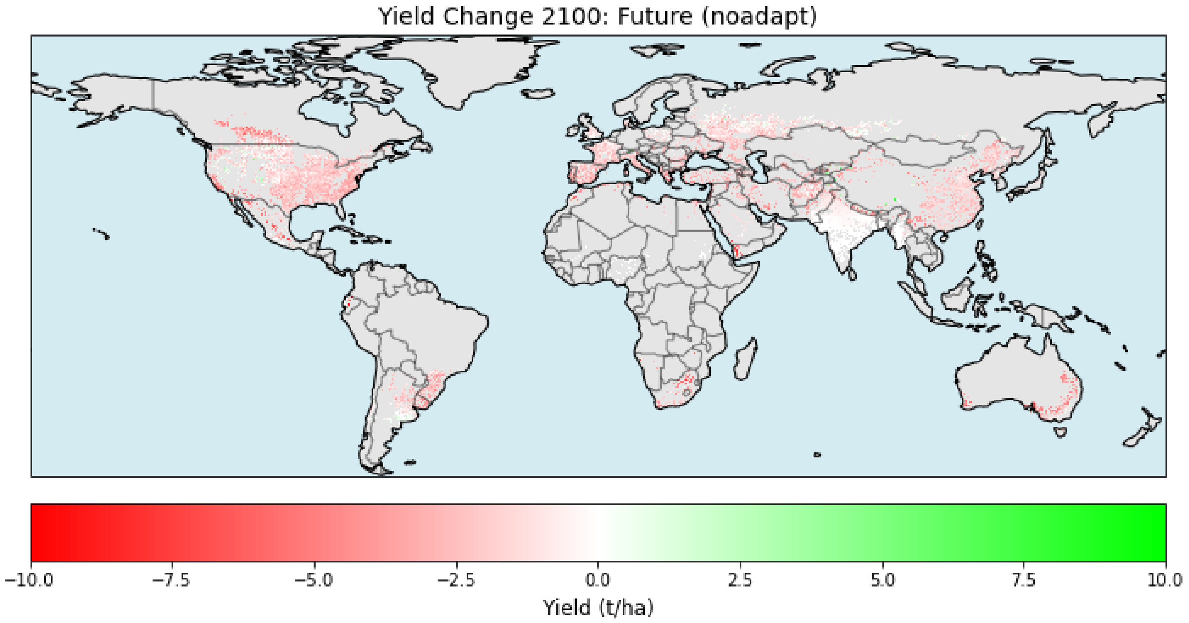

Figure S77: Global irrigated winter wheat productivity: Projected yield change by 2100 without adaptation

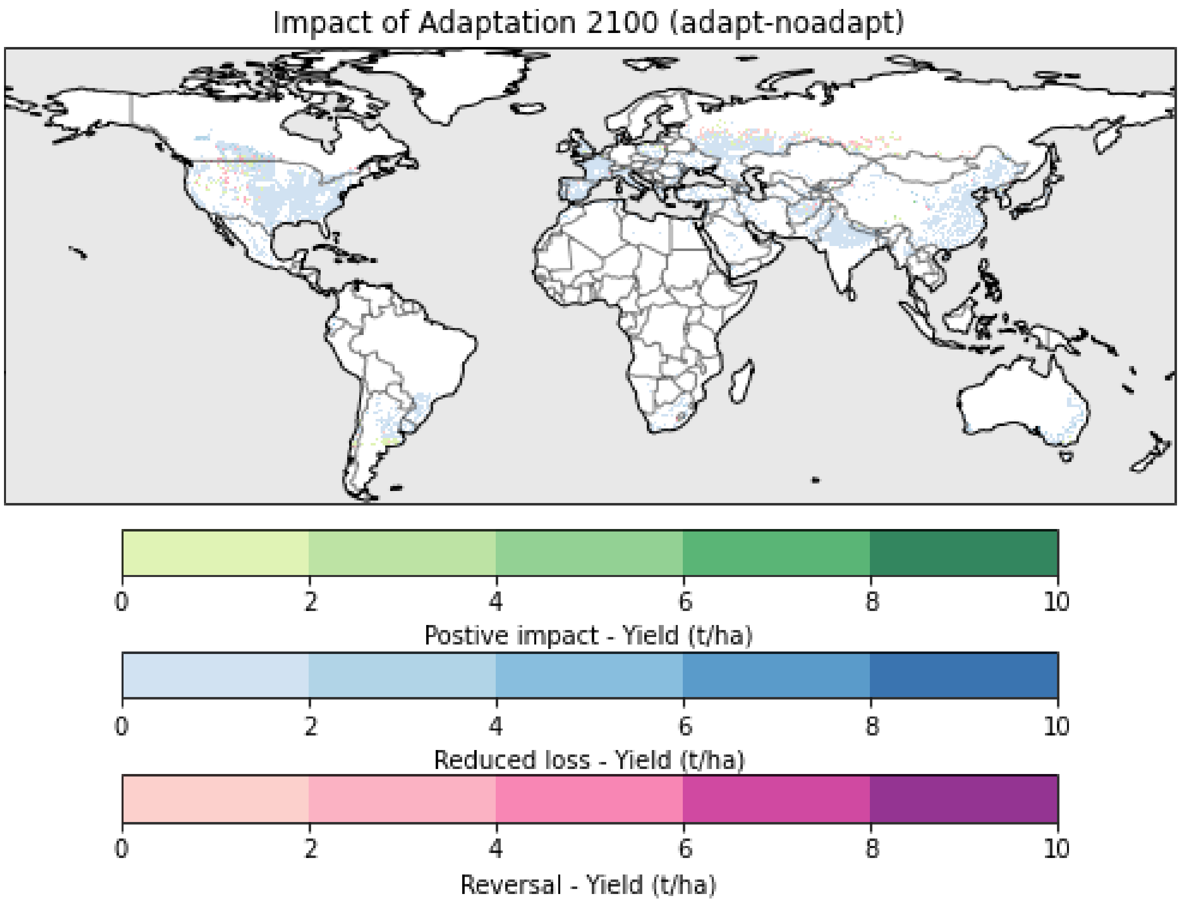

Figure S78: Global irrigated winter wheat productivity: Impact of adaptation on 2100 yields

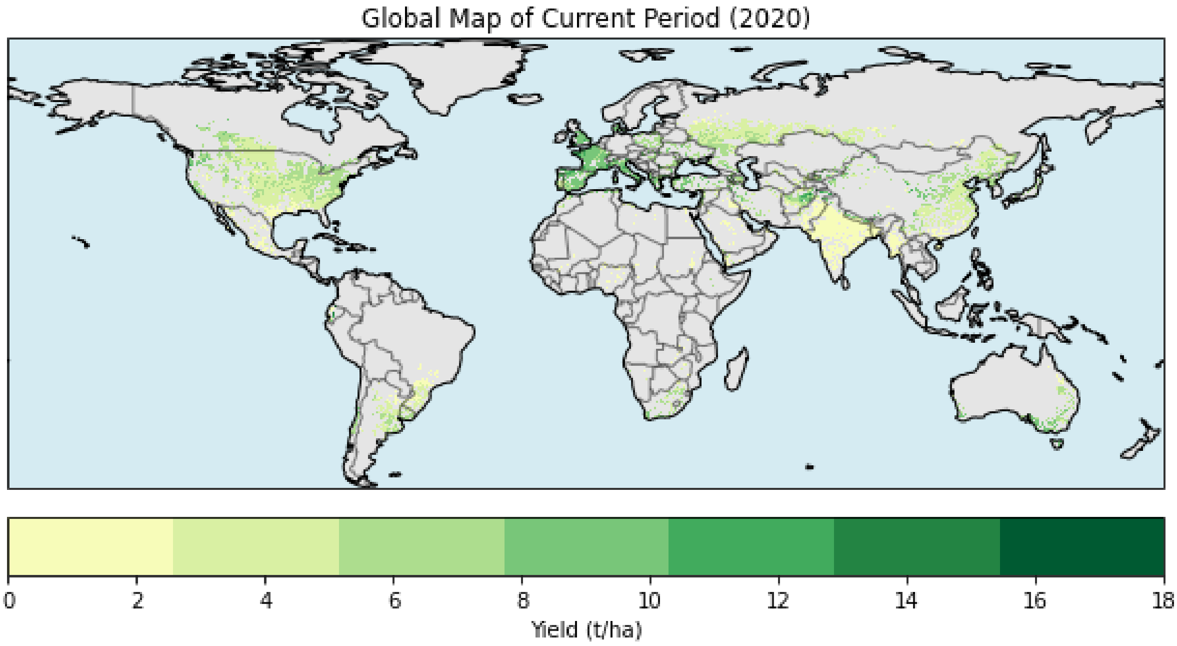

Figure S79: Global irrigated winter wheat reliability: Baseline yield in 2020

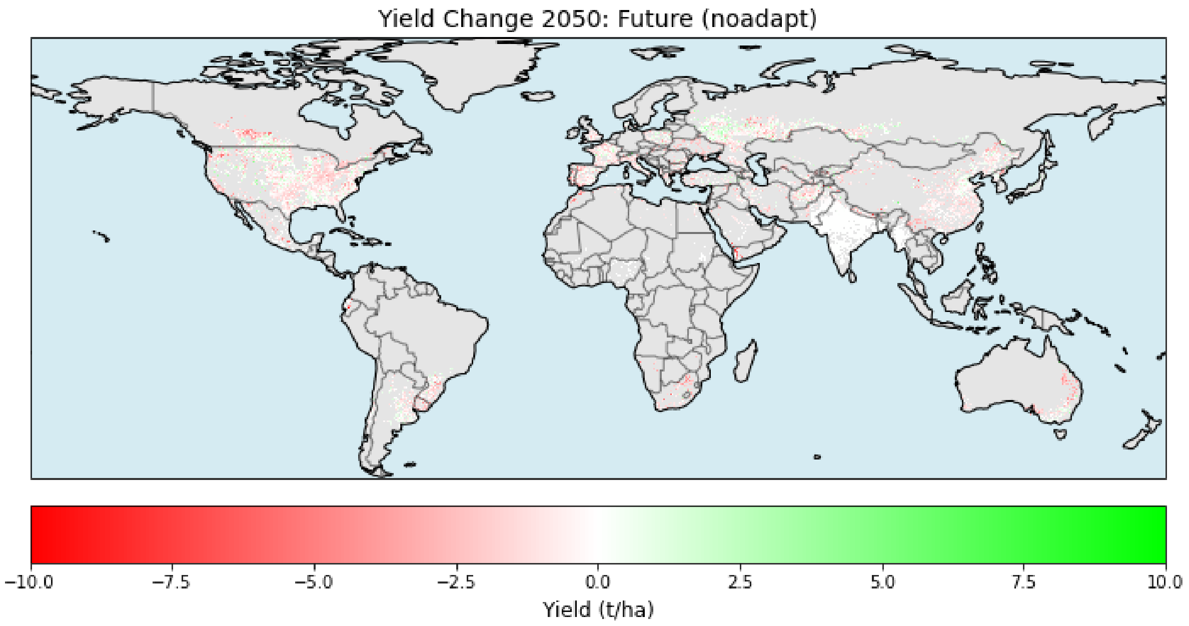

Figure S80: Global irrigated winter wheat reliability: Projected yield change by 2050 without adaptation

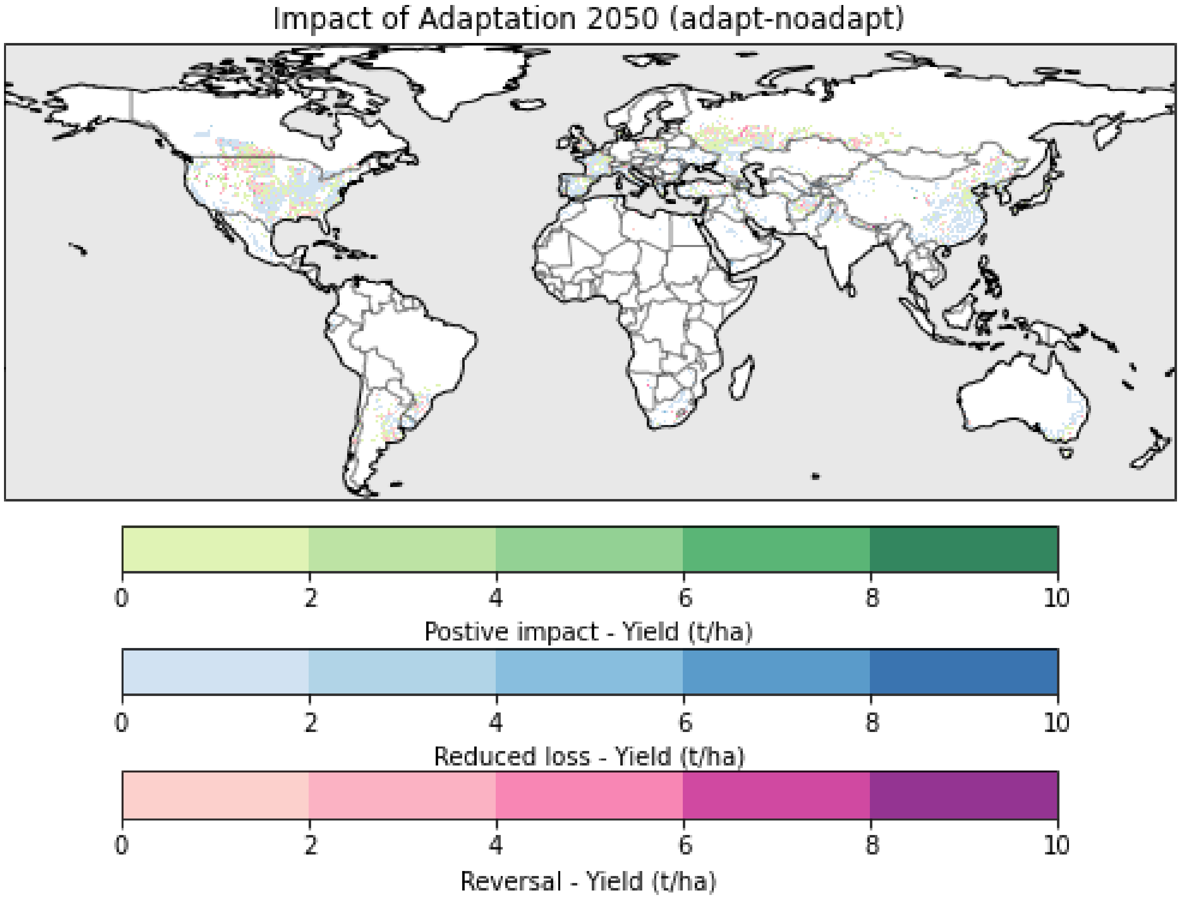

Figure S81: Global irrigated winter wheat reliability: Impact of adaptation on 2050 yields

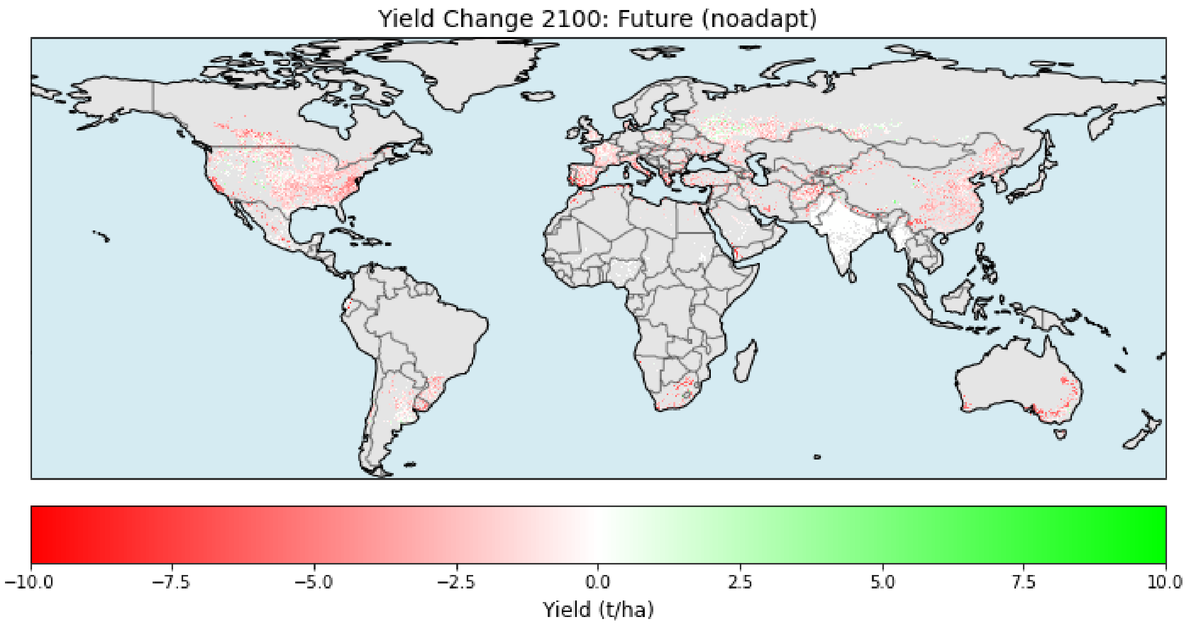

Figure S82: Global irrigated winter wheat reliability: Projected yield change by 2100 without adaptation

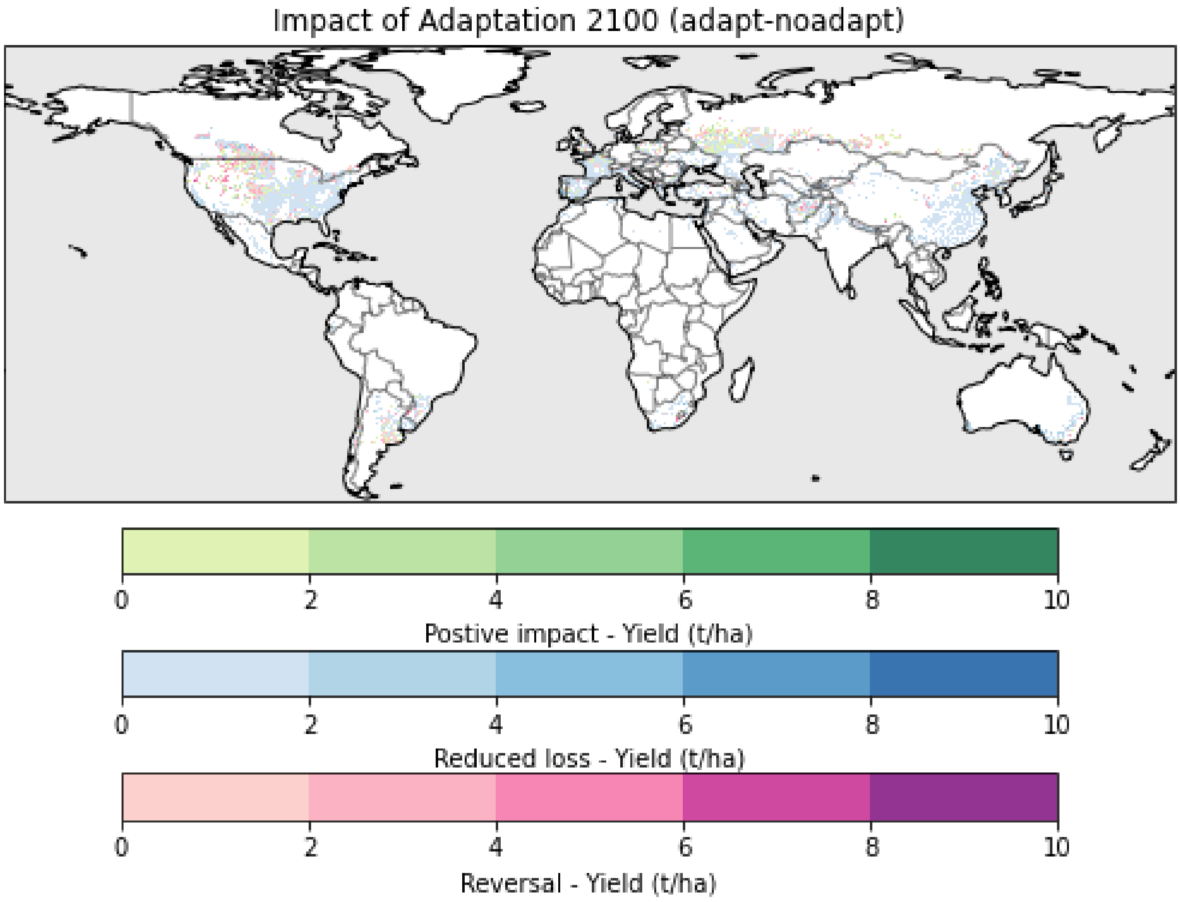

Figure S83: Global irrigated winter wheat reliability: Impact of adaptation on 2100 yields

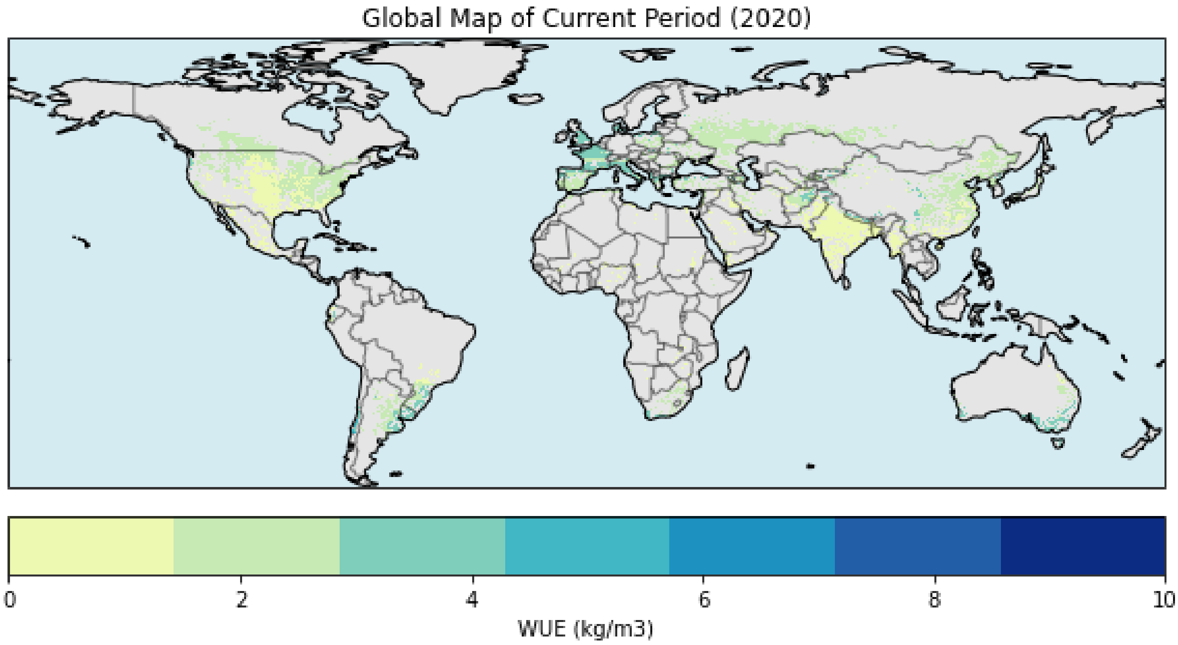

Figure S84: Global irrigated winter wheat water use efficiency: Baseline water use efficiency in 2020

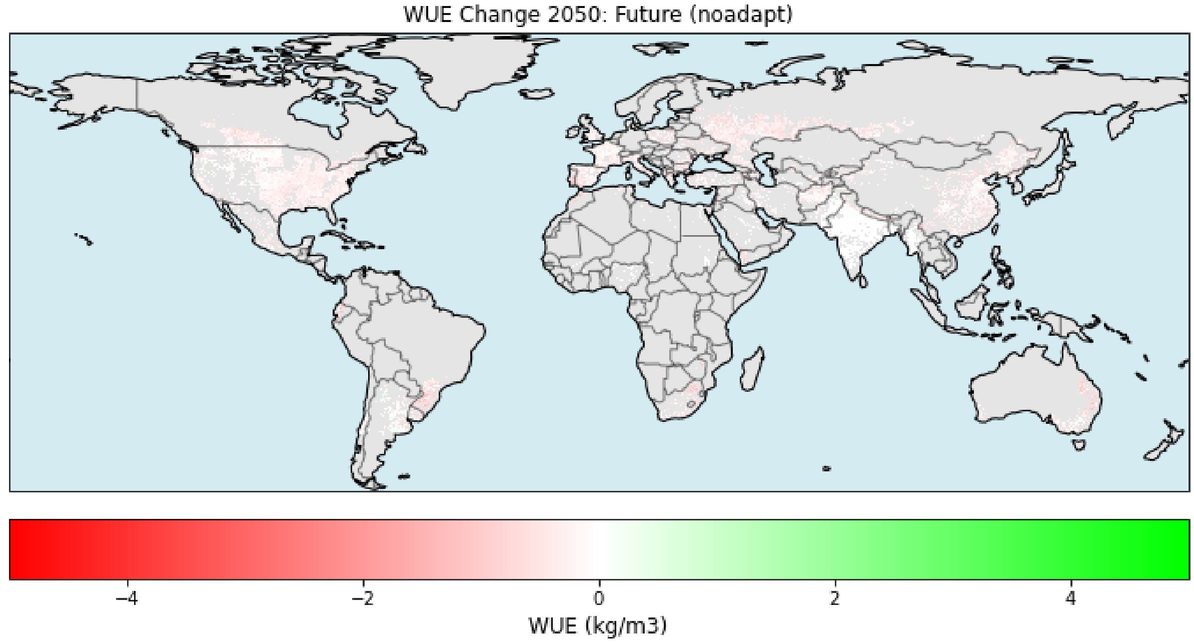

Figure S85: Global irrigated winter wheat water use efficiency: Projected water use efficiency change by 2050 without adaptation

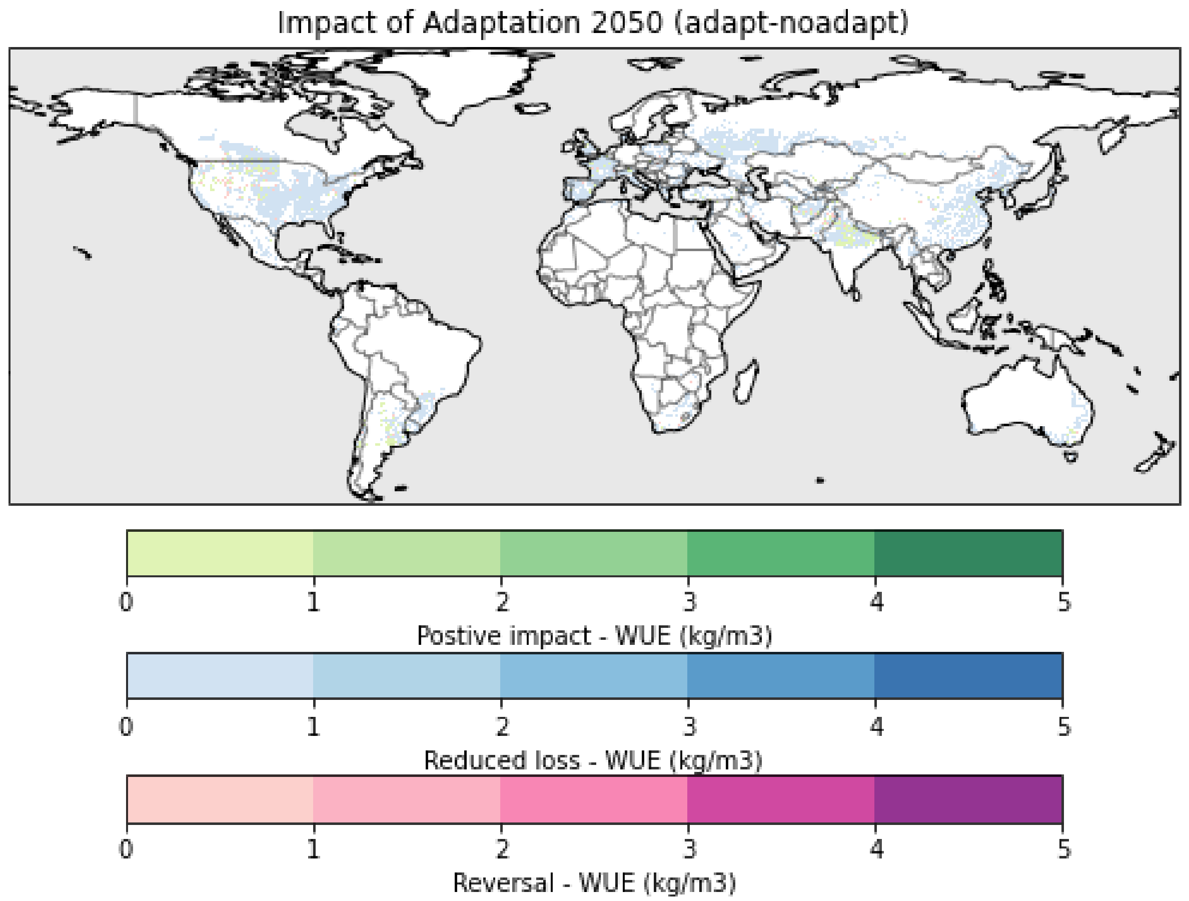

Figure S86: Global irrigated winter wheat water use efficiency: Impact of adaptation on 2050 water use efficiency

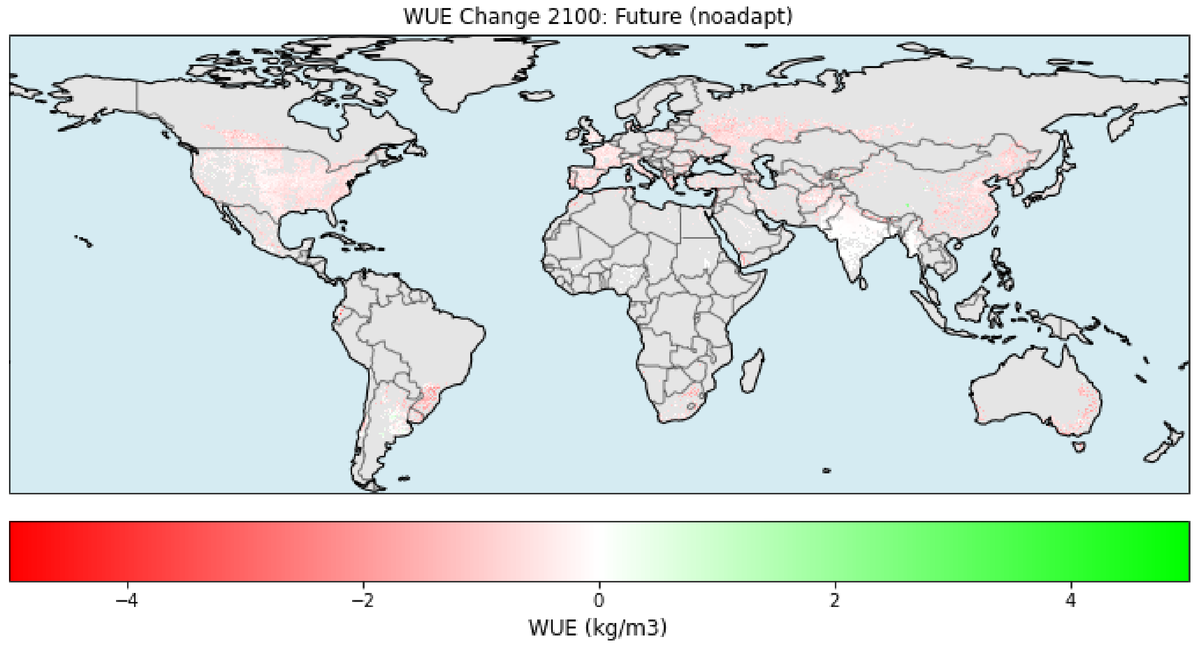

Figure S87: Global irrigated winter wheat water use efficiency: Projected water use efficiency change by 2100 without adaptation

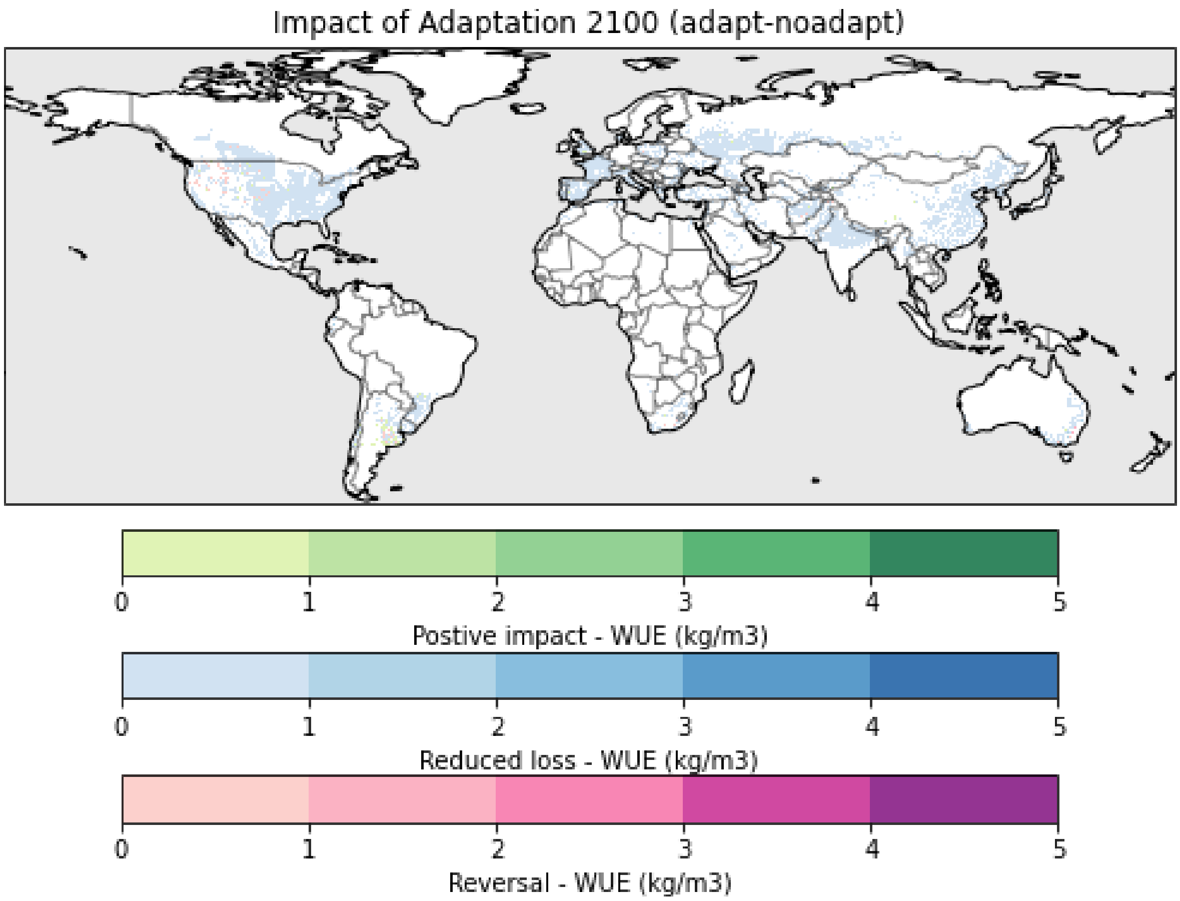

Figure S88: Global irrigated winter wheat water use efficiency: Impact of adaptation on 2100 water use efficiency

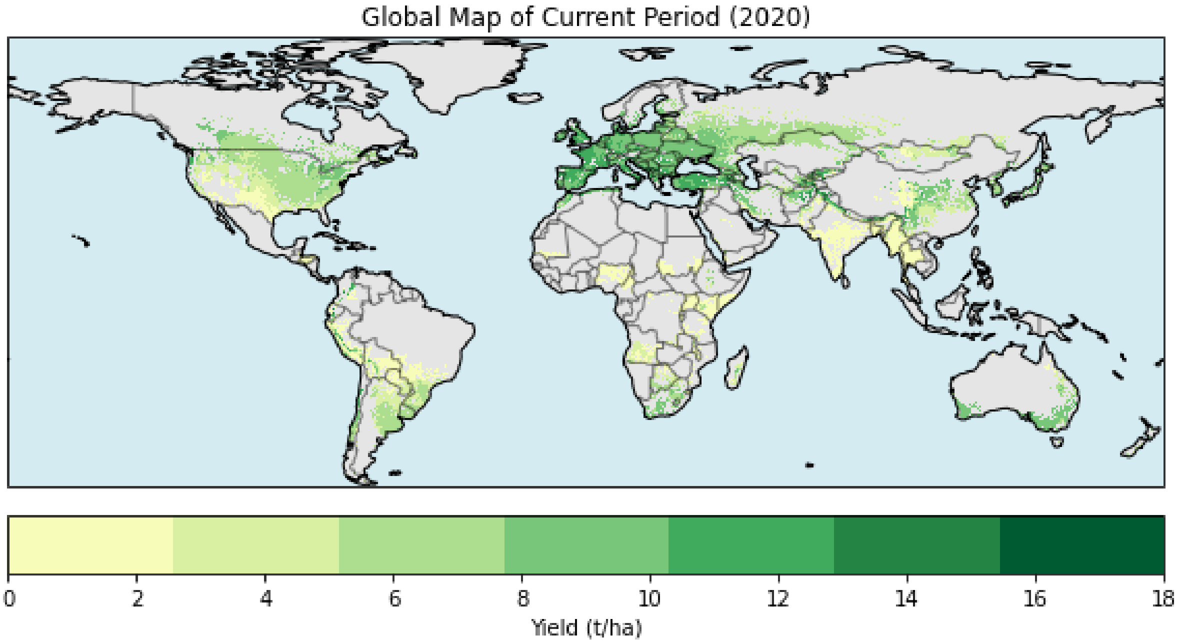

Figure S89: Global rainfed winter wheat productivity: Baseline yield in 2020

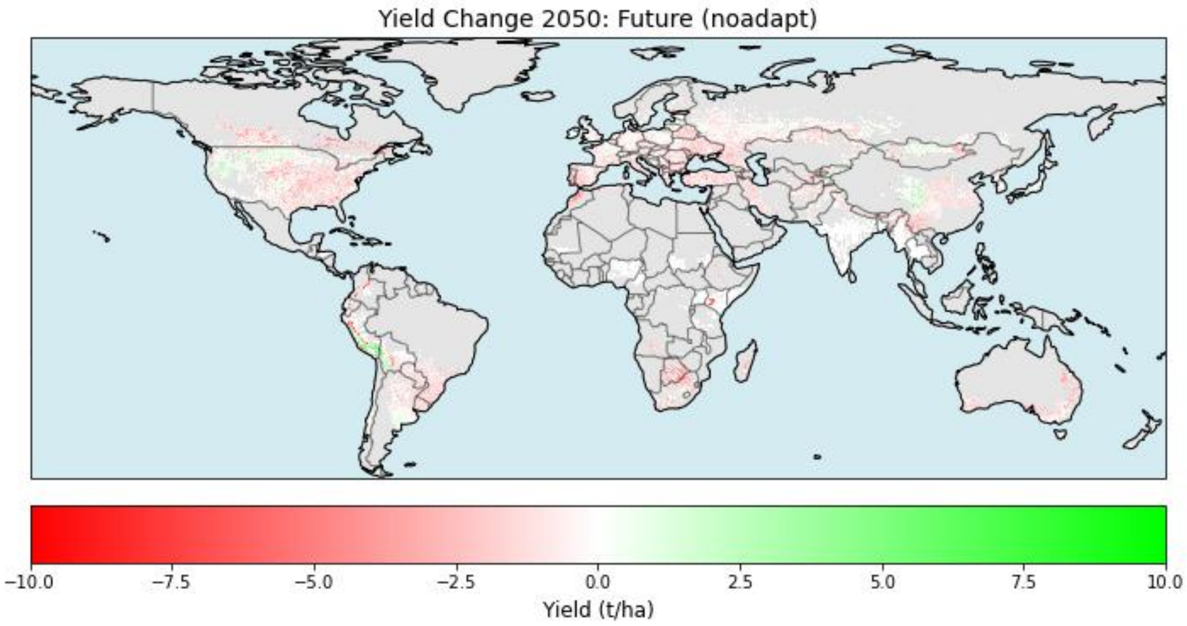

Figure S90: Global rainfed winter wheat productivity: Projected yield change by 2050 without adaptation

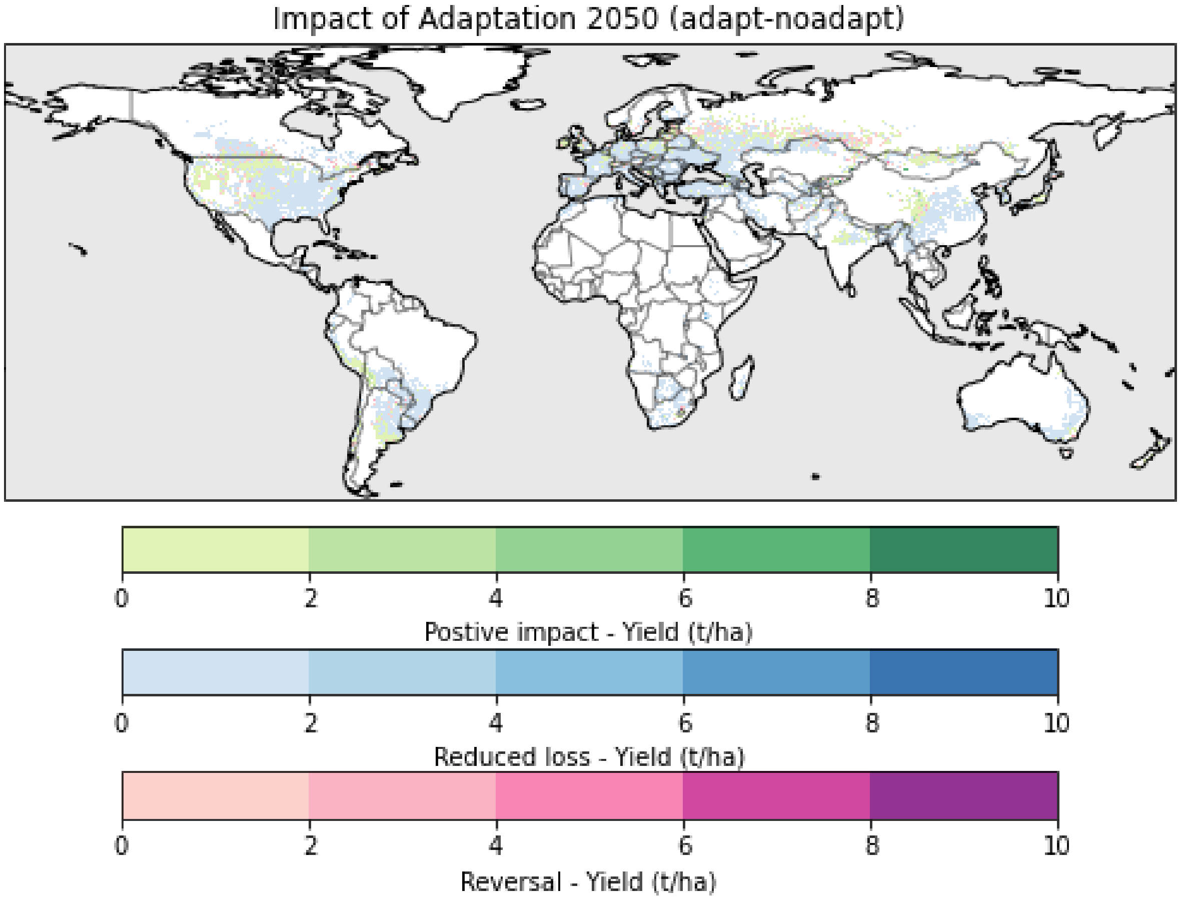

Figure S91: Global rainfed winter wheat productivity: Impact of adaptation on 2050 yields

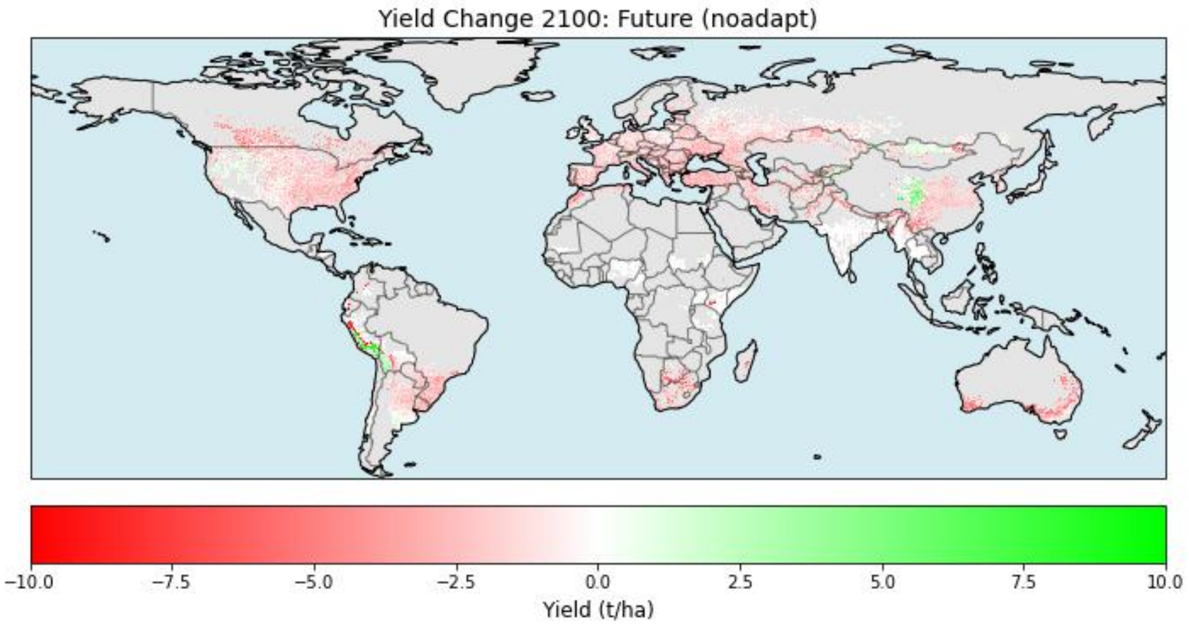

Figure S92: Global rainfed winter wheat productivity: Projected yield change by 2100 without adaptation

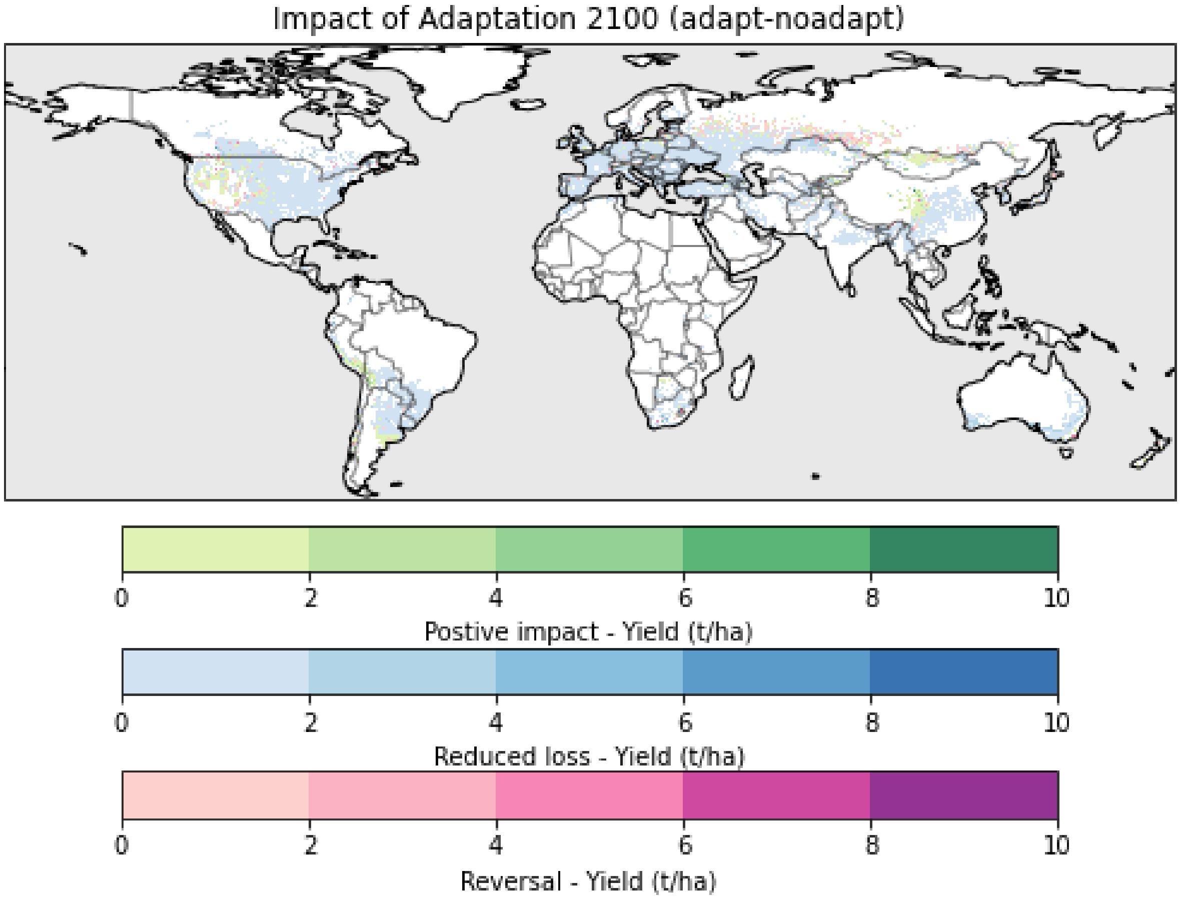

Figure S93: Global rainfed winter wheat productivity: Impact of adaptation on 2100 yields

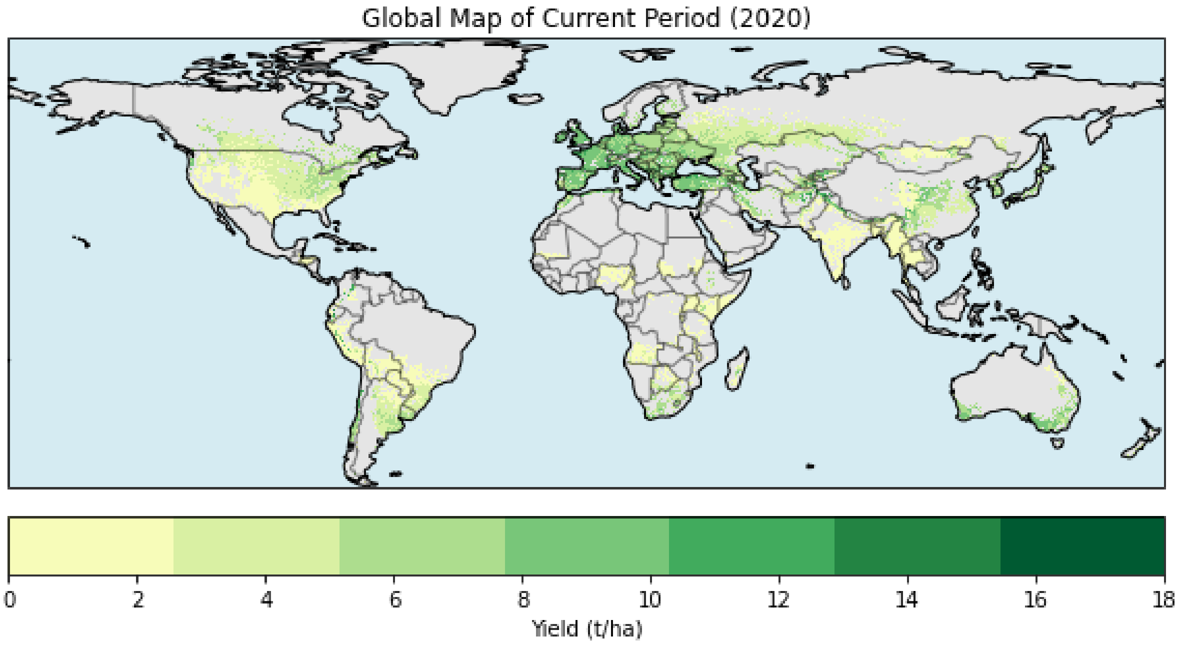

Figure S94: Global rainfed winter wheat reliability: Baseline yield in 2020

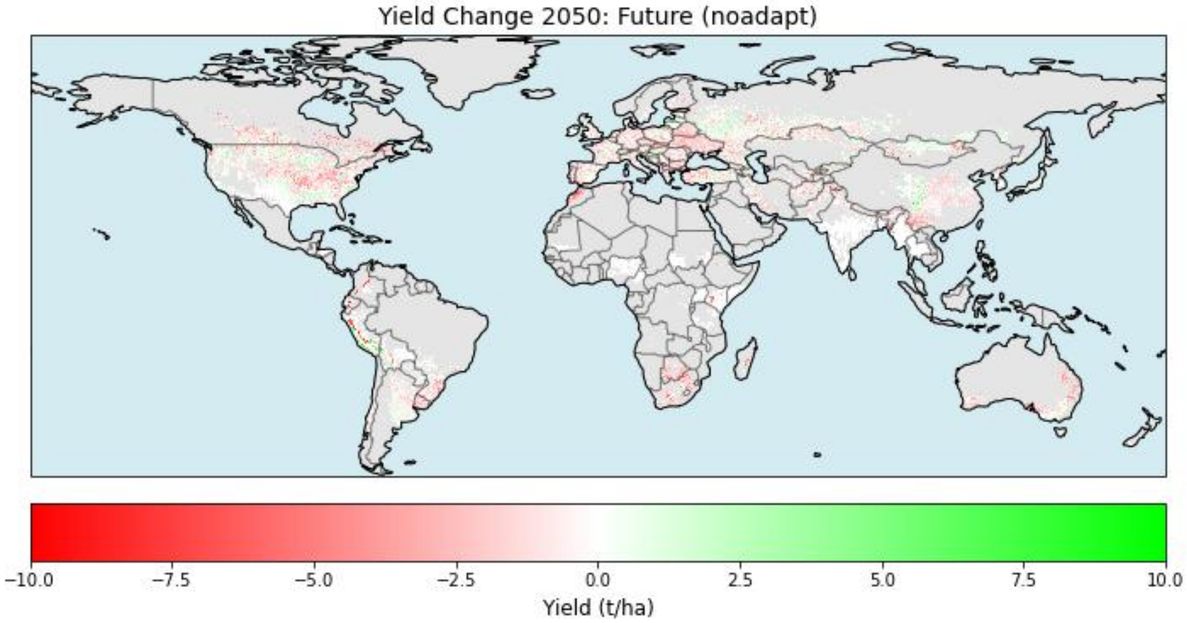

Figure S95: Global rainfed winter wheat reliability: Projected yield change by 2050 without adaptation

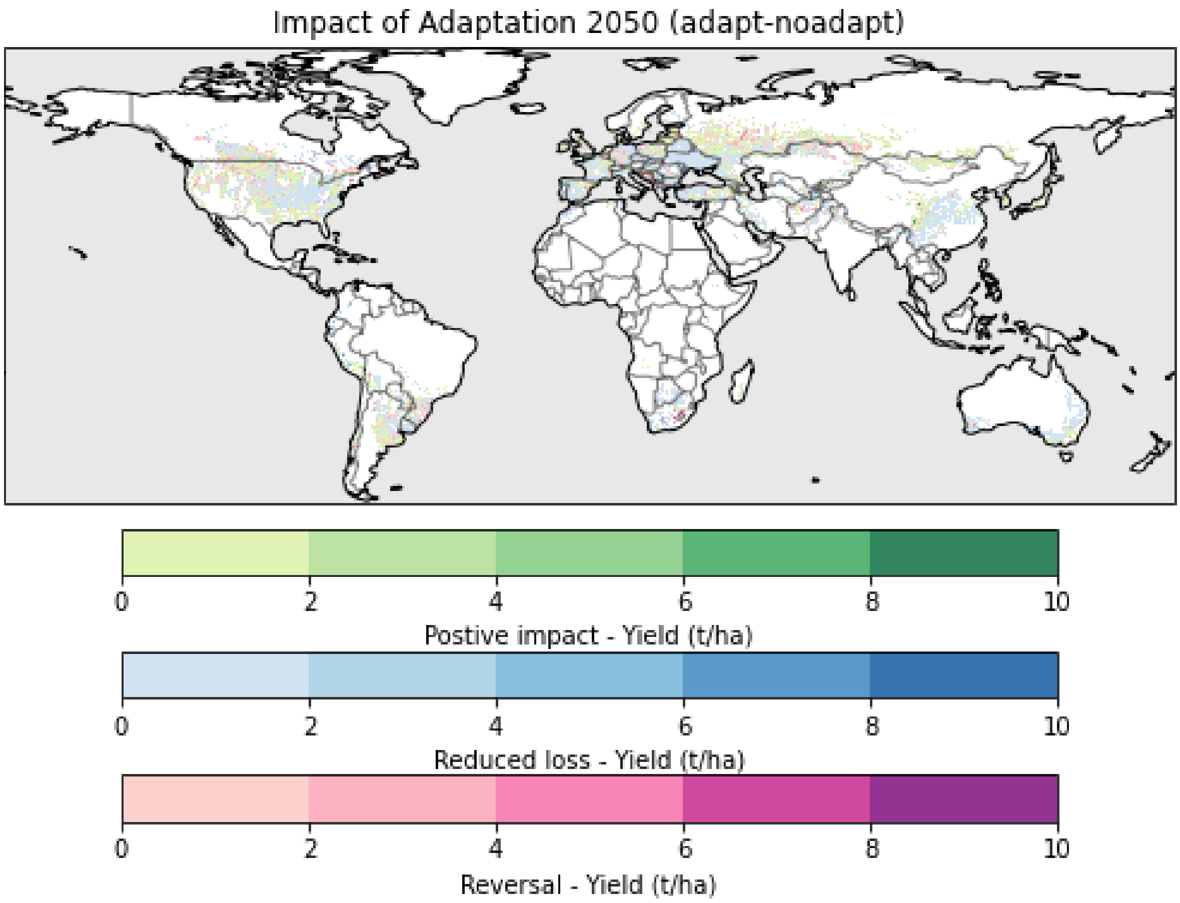

Figure S96: Global rainfed winter wheat reliability: Impact of adaptation on 2050 yields

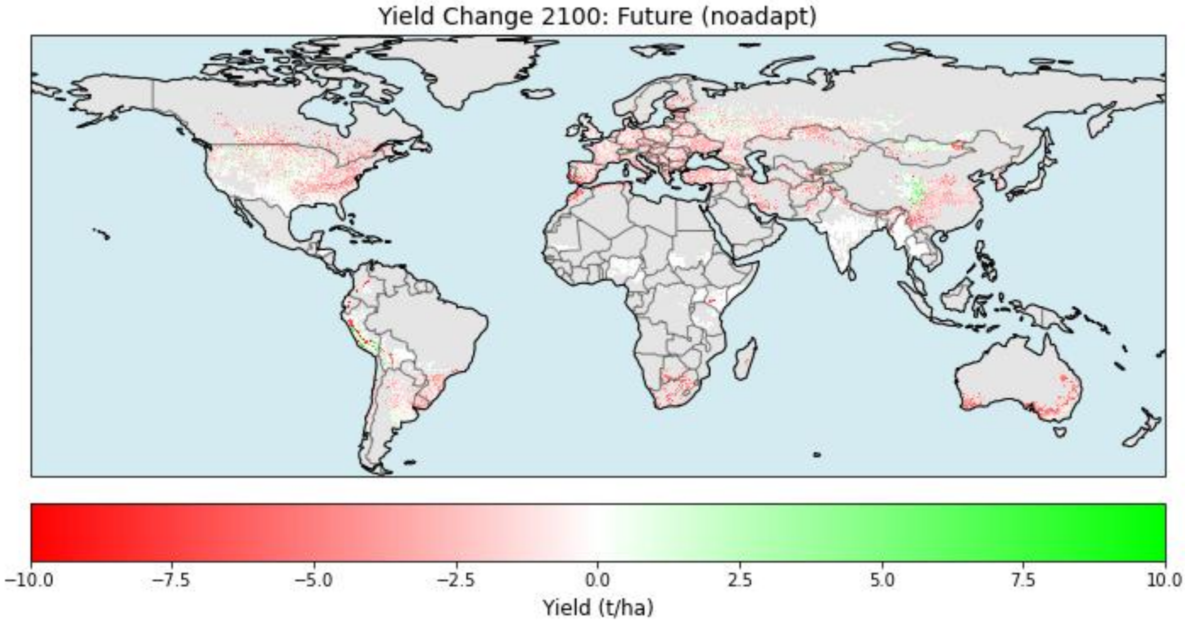

Figure S97: Global rainfed winter wheat reliability: Projected yield change by 2100 without adaptation

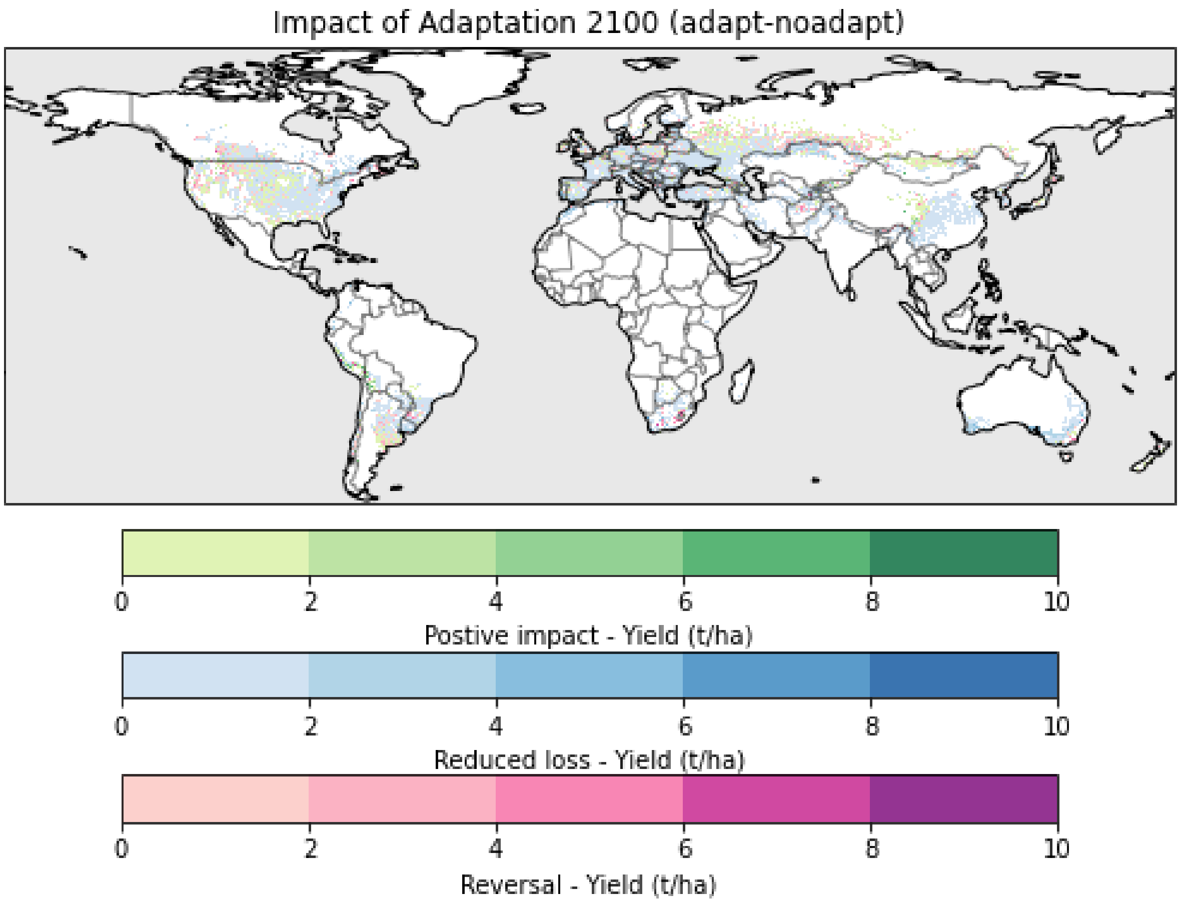

Figure S98: Global rainfed winter wheat reliability: Impact of adaptation on 2100 yields

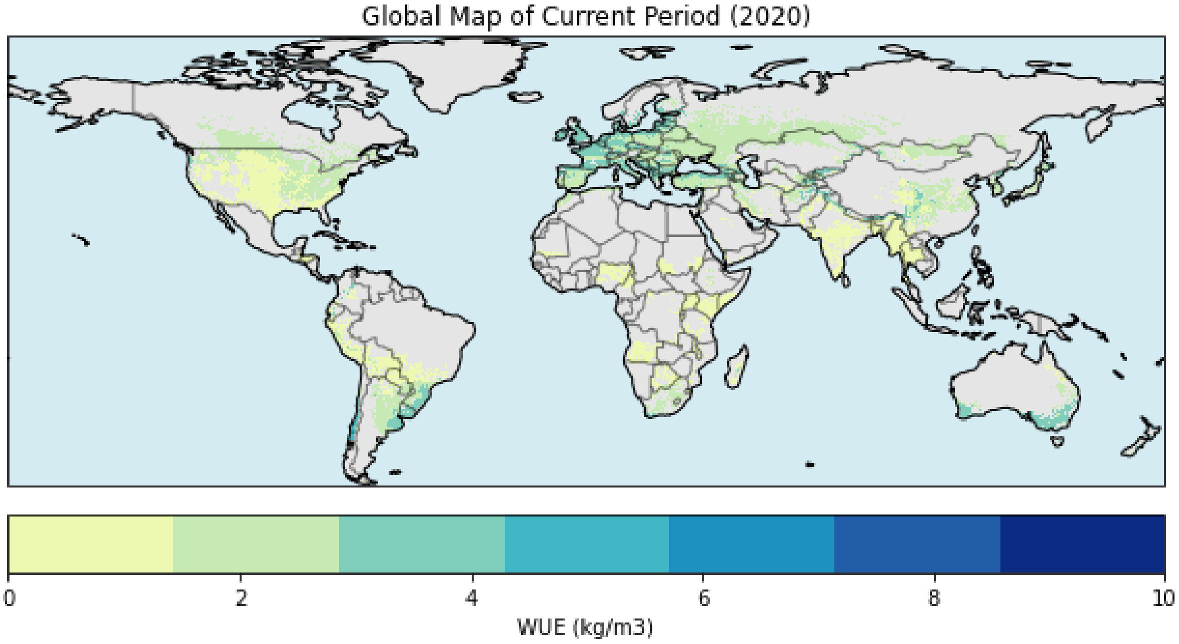

Figure S99: Global rainfed winter wheat water use efficiency: Baseline water use efficiency in 2020

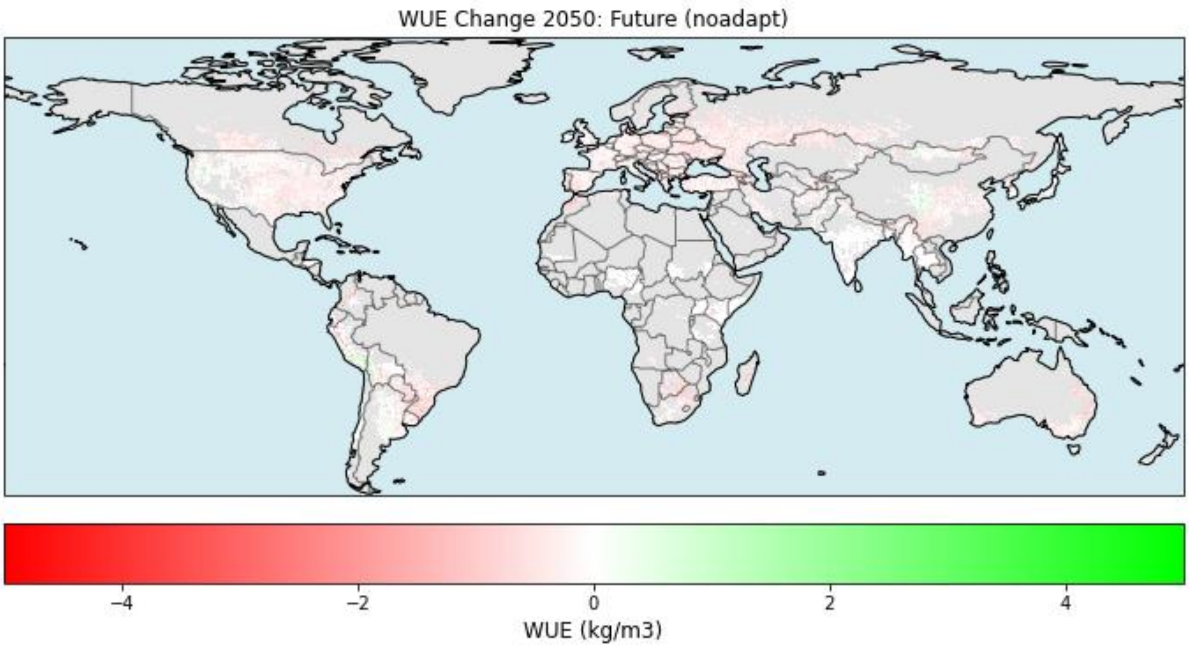

Figure S100: Global rainfed winter wheat water use efficiency: Projected water use efficiency change by 2050 without adaptation

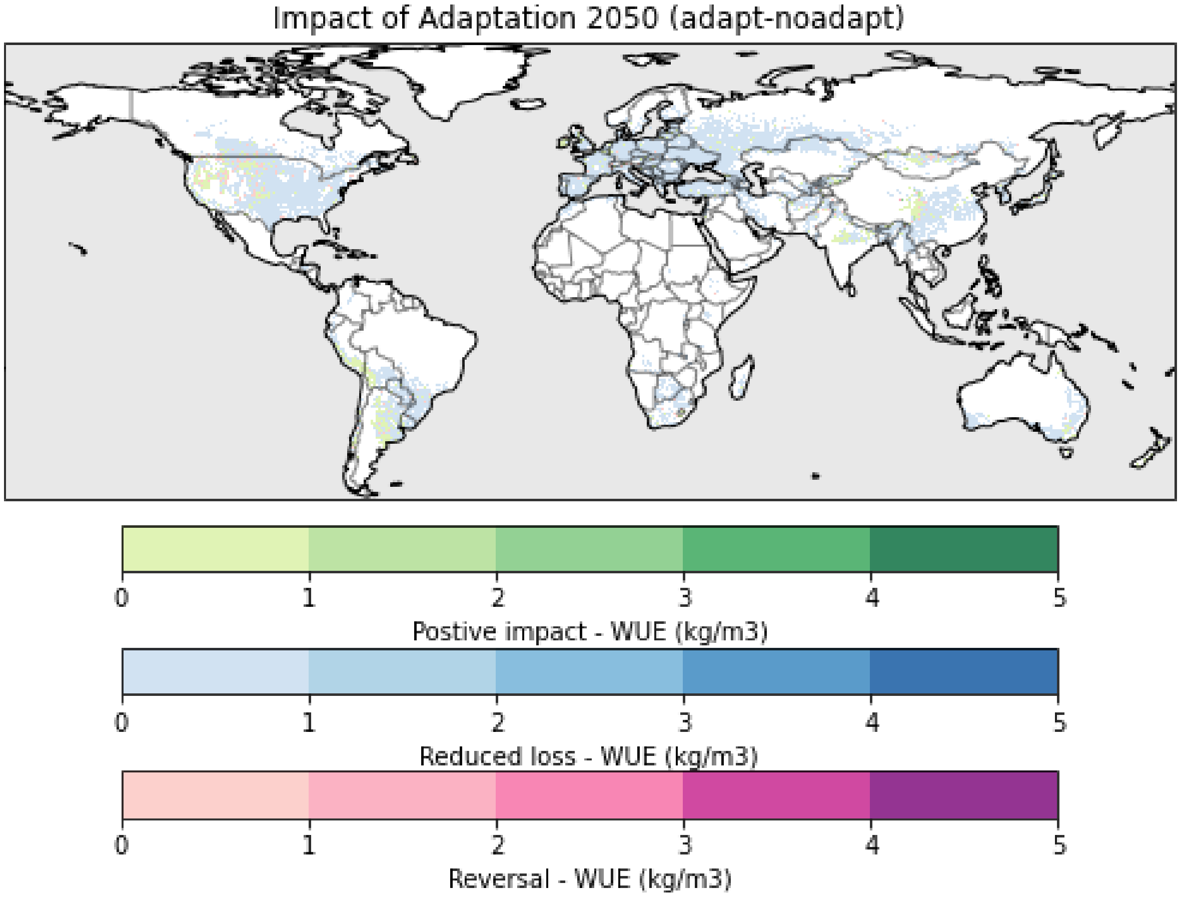

Figure S101: Global rainfed winter wheat water use efficiency: Impact of adaptation on 2050 water use efficiency

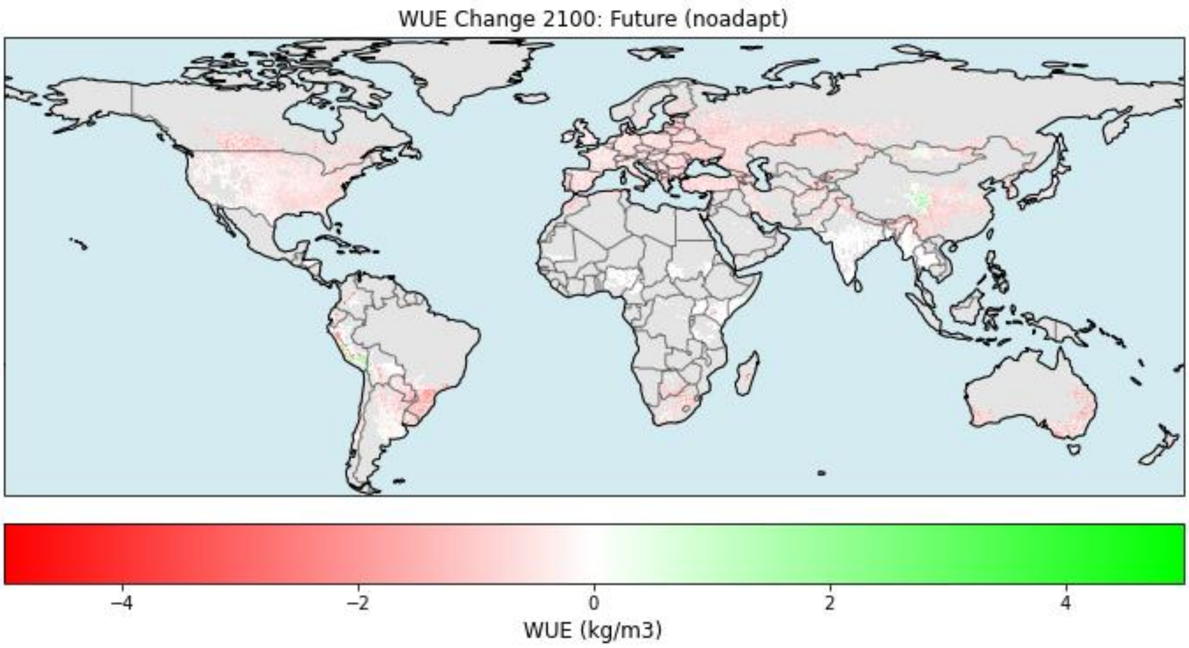

Figure S102: Global rainfed winter wheat water use efficiency: Projected water use efficiency change by 2100 without adaptation

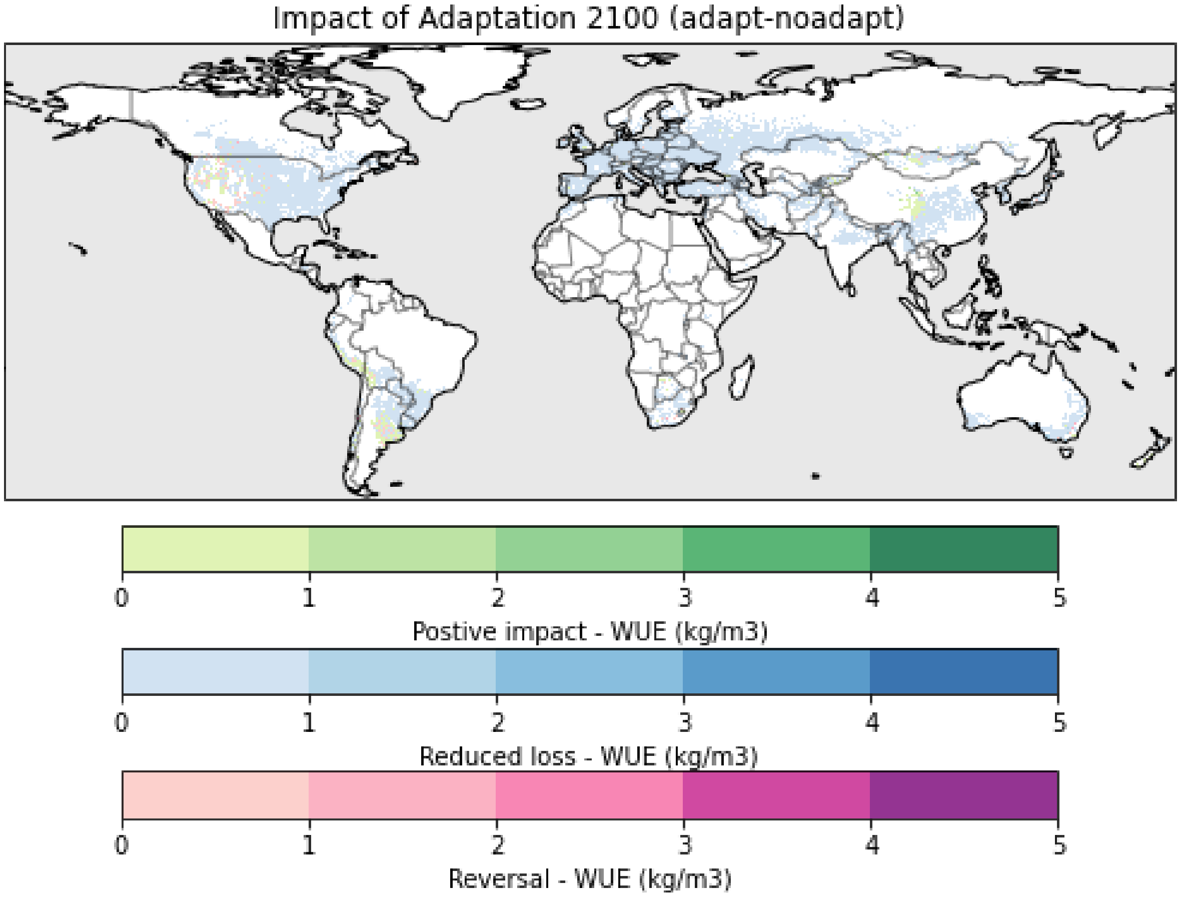

Figure S103: Global rainfed winter wheat water use efficiency: Impact of adaptation on 2100 water use efficiency

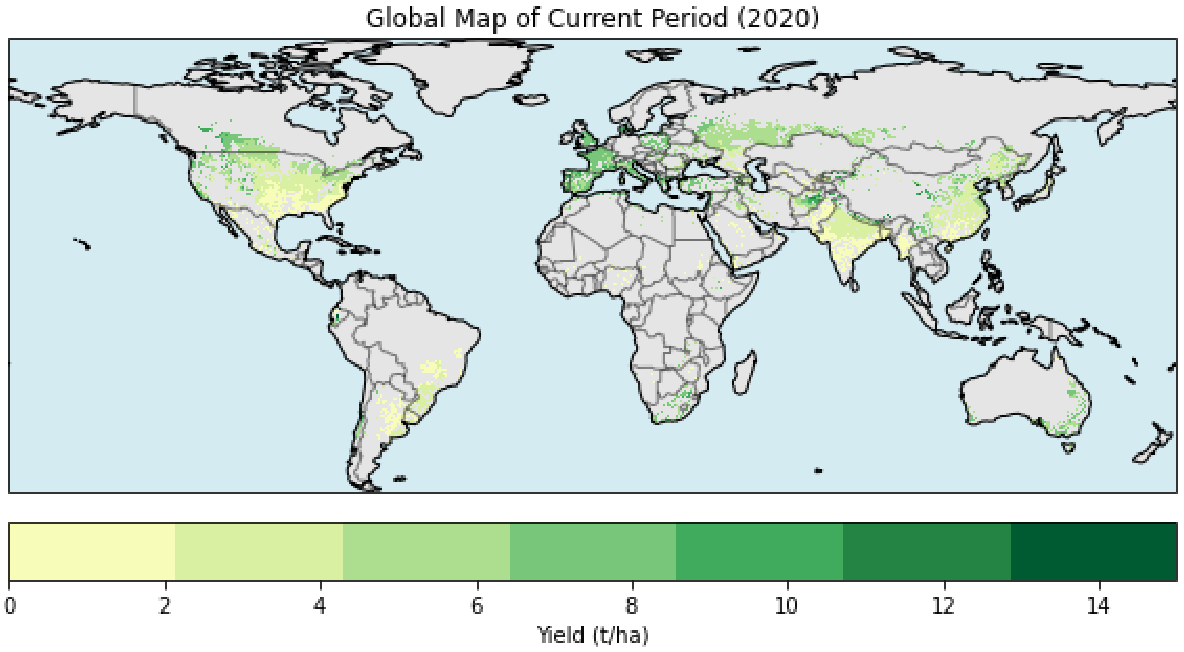

Figure S104: Global irrigated spring wheat productivity: Baseline yield in 2020

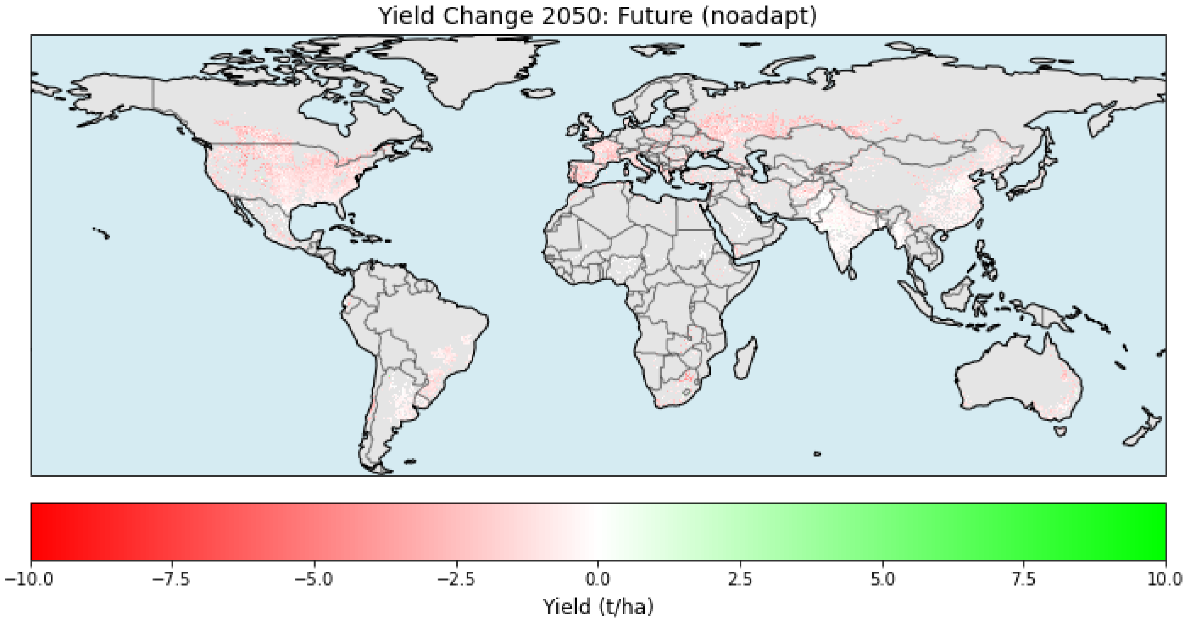

Figure S105: Global irrigated spring wheat productivity: Projected yield change by 2050 without adaptation

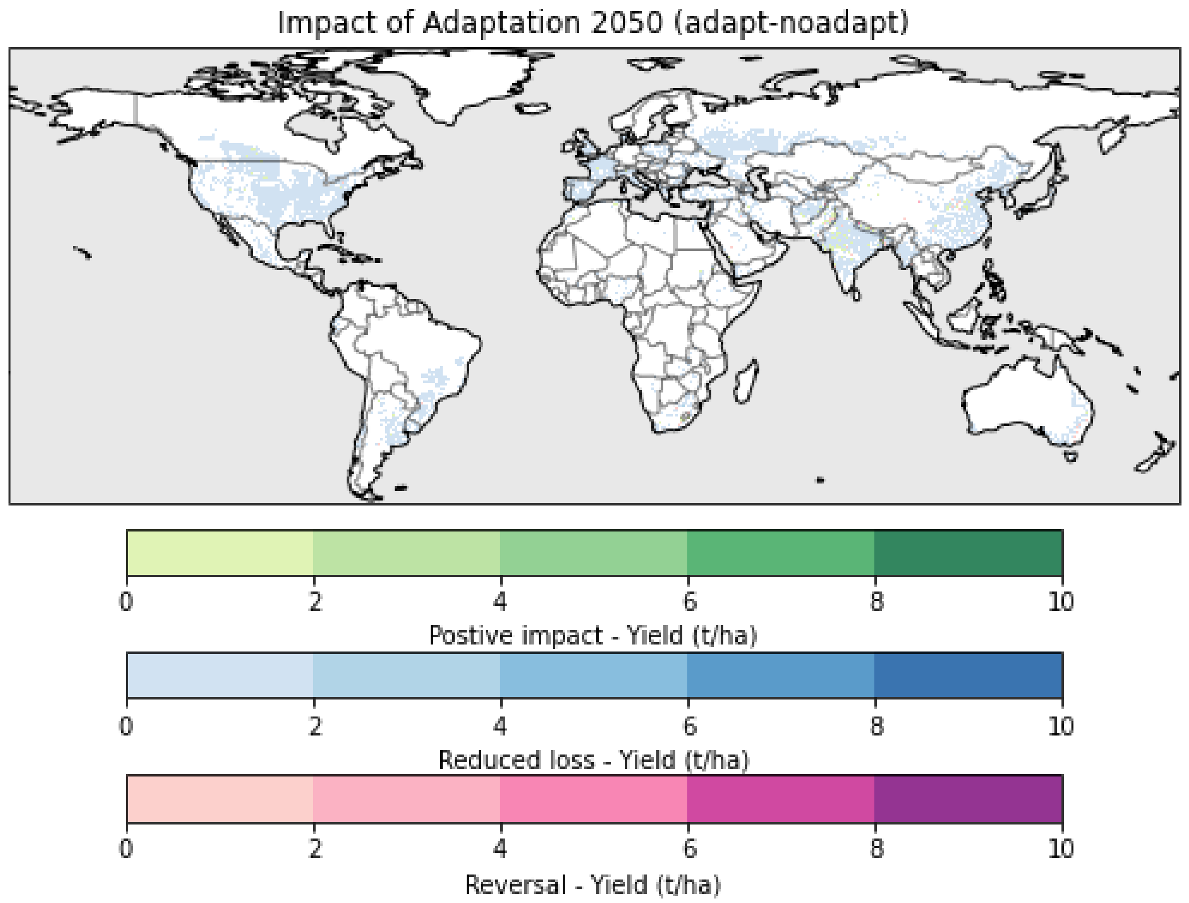

Figure S106: Global irrigated spring wheat productivity: Impact of adaptation on 2050 yields

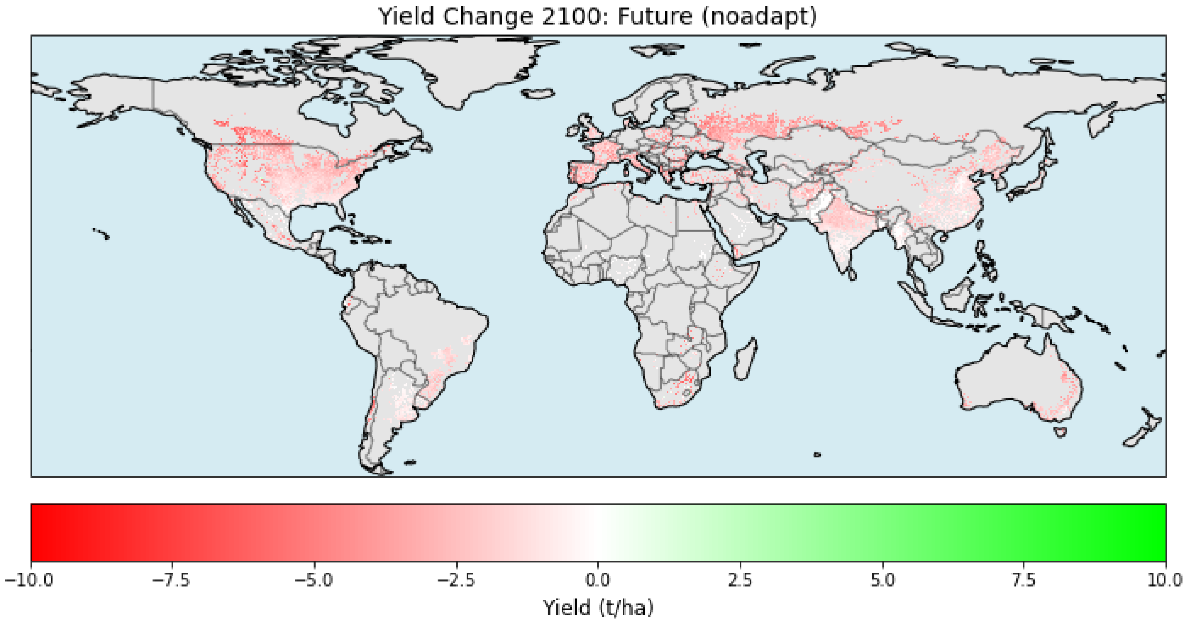

Figure S107: Global irrigated spring wheat productivity: Projected yield change by 2100 without adaptation

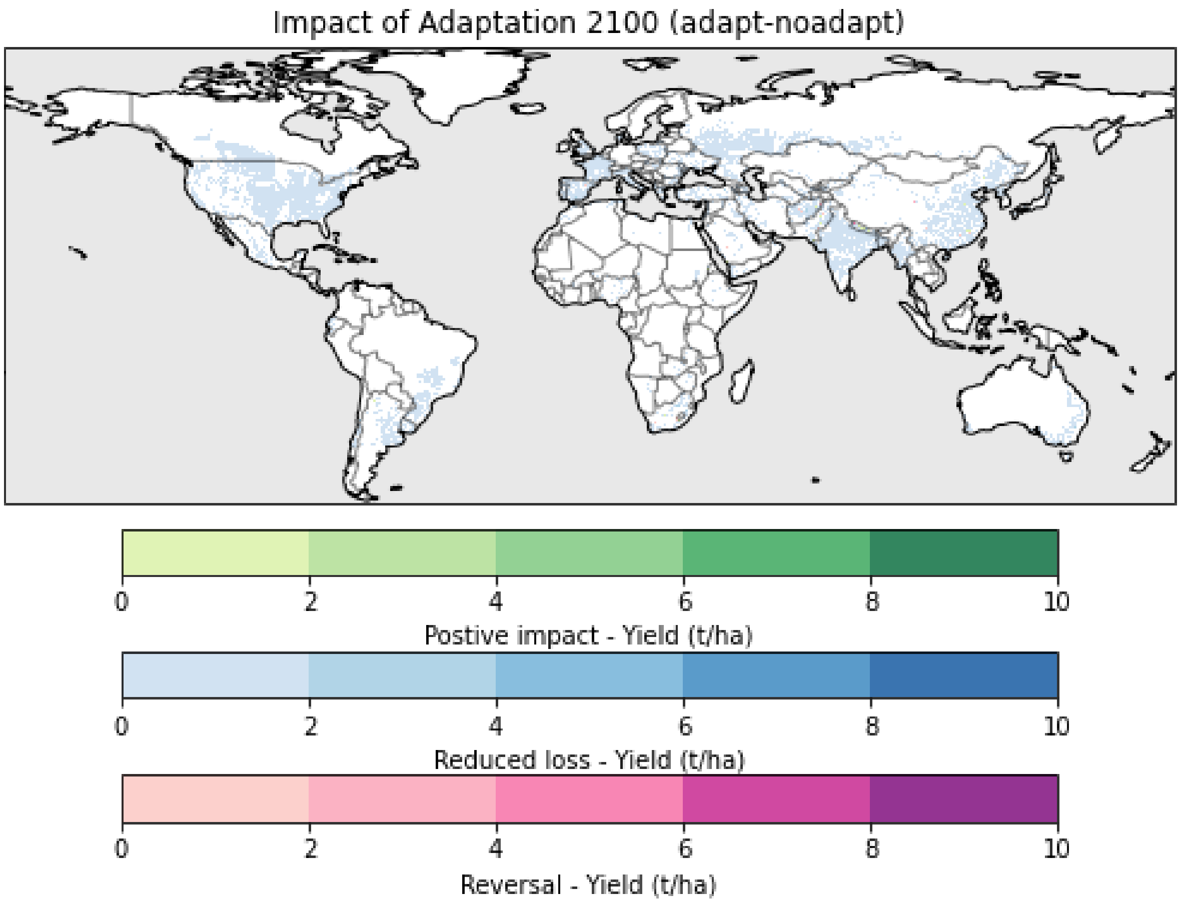

Figure S108: Global irrigated spring wheat productivity: Impact of adaptation on 2100 yields

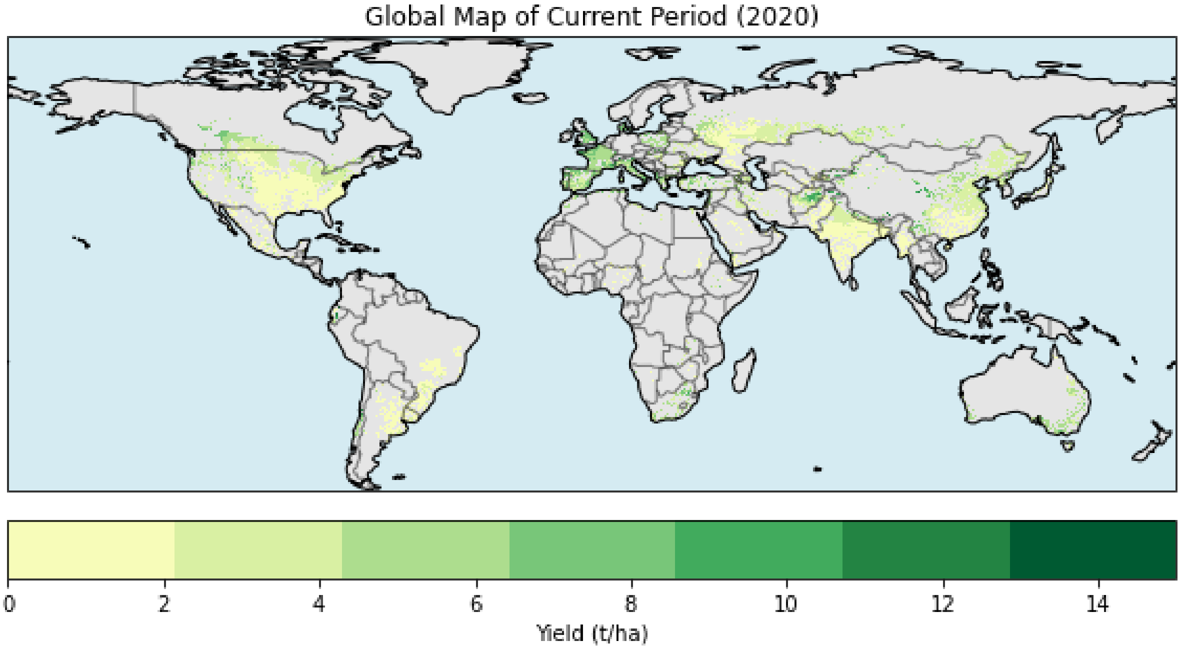

Figure S109: Global irrigated spring wheat reliability: Baseline yield in 2020

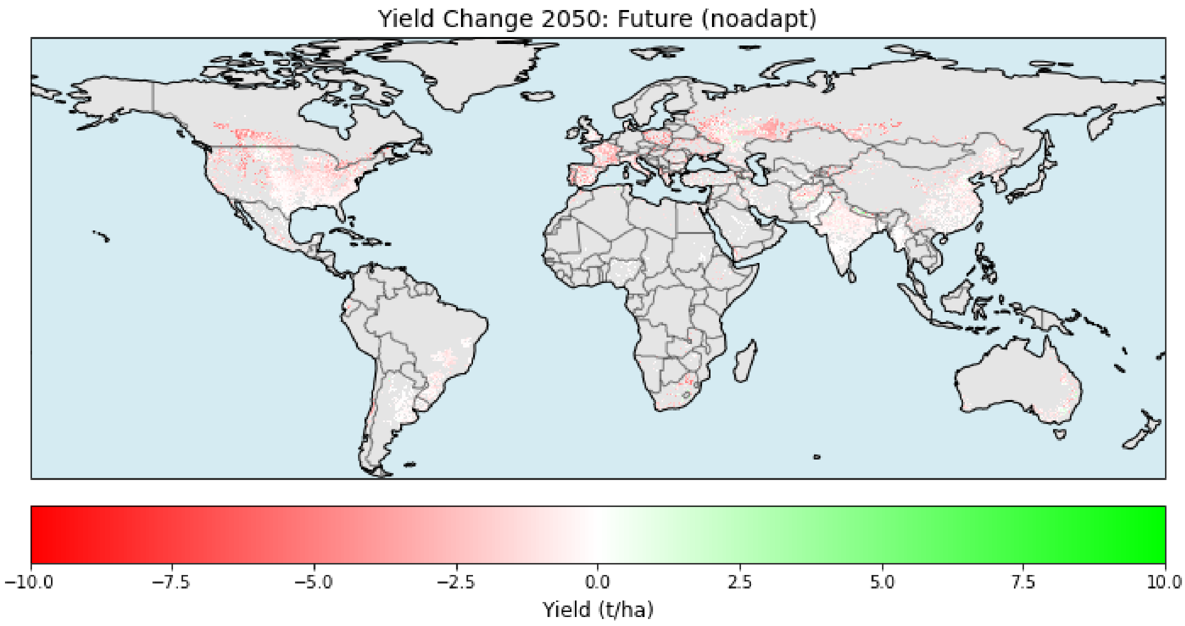

Figure S110: Global irrigated spring wheat reliability: Projected yield change by 2050 without adaptation

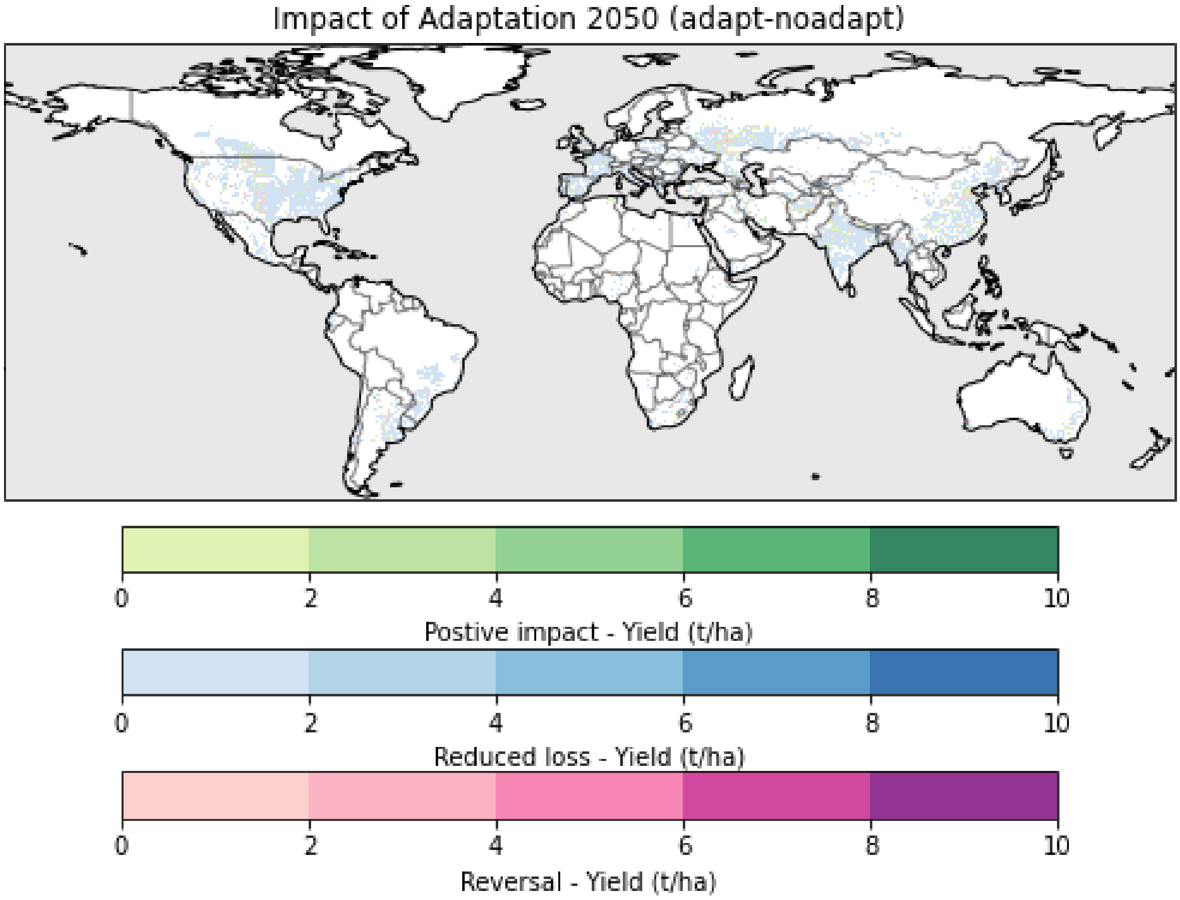

Figure S111: Global irrigated spring wheat reliability: Impact of adaptation on 2050 yields

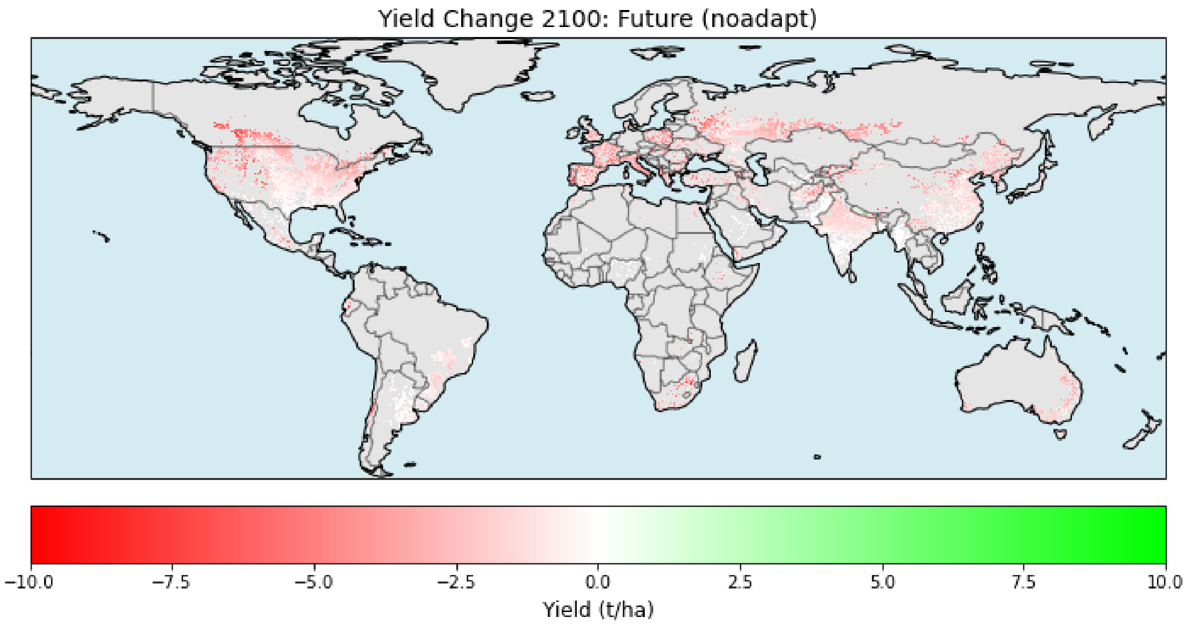

Figure S112: Global irrigated spring wheat reliability: Projected yield change by 2100 without adaptation

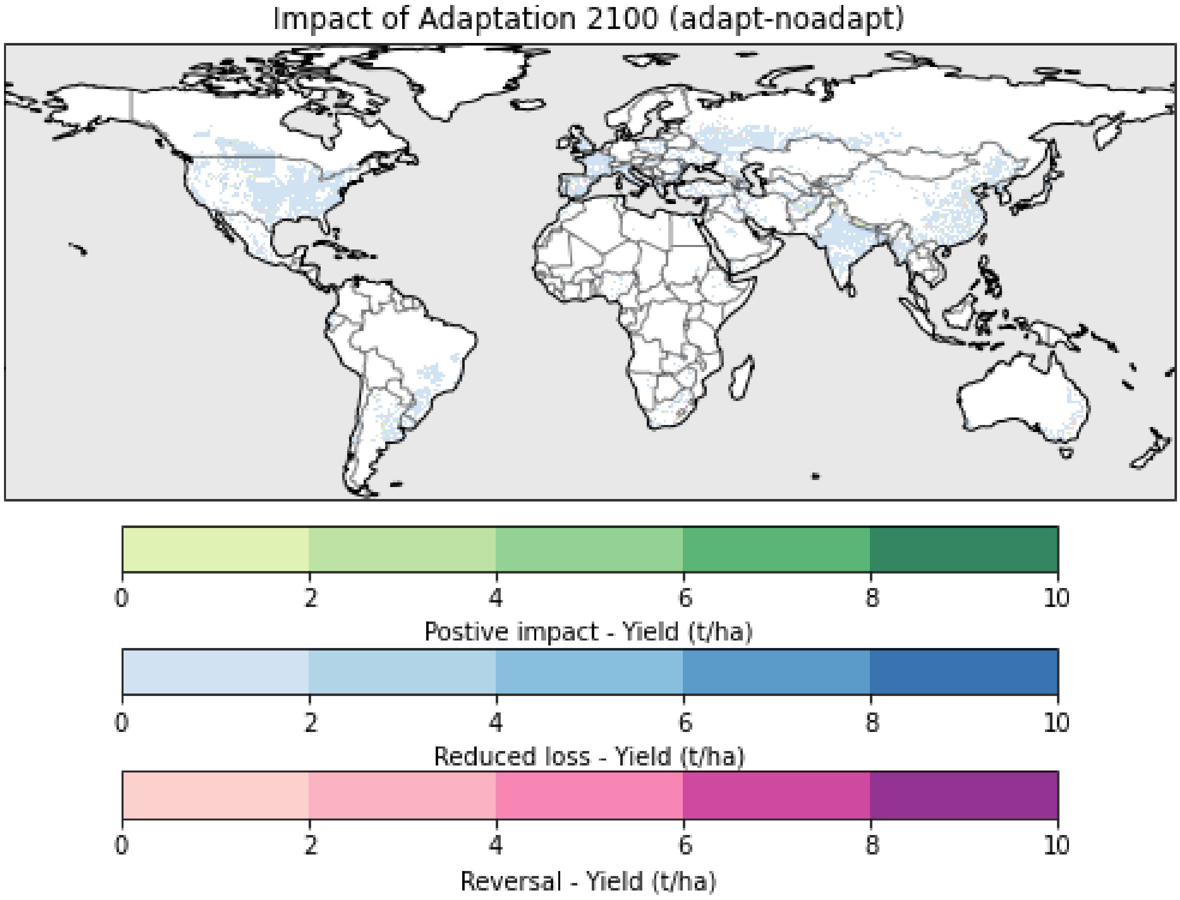

Figure S113: Global irrigated spring wheat reliability: Impact of adaptation on 2100 yields

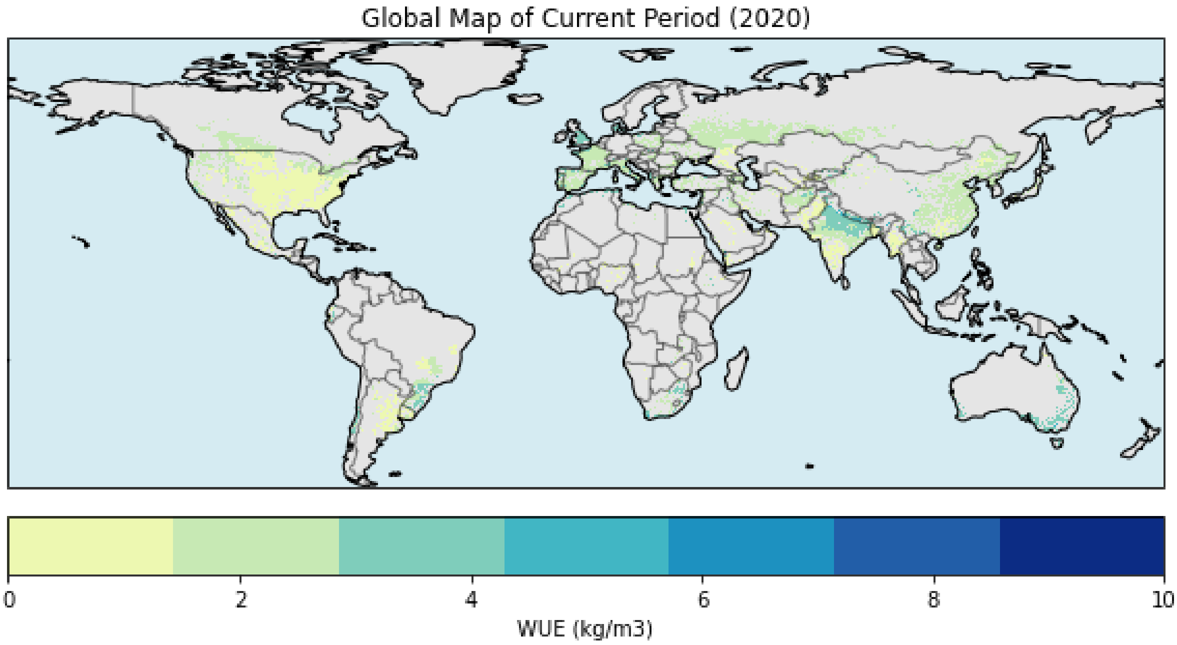

Figure S114: Global irrigated spring wheat water use efficiency: Baseline water use efficiency in 2020

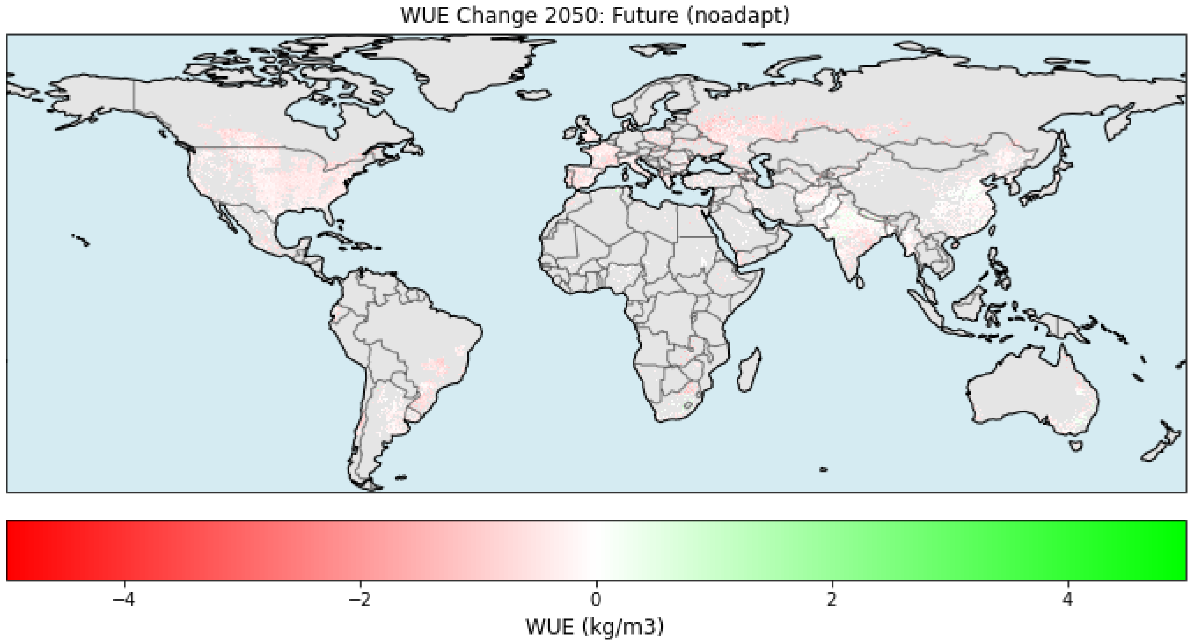

Figure S115: Global irrigated spring wheat water use efficiency: Projected water use efficiency change by 2050 without adaptation

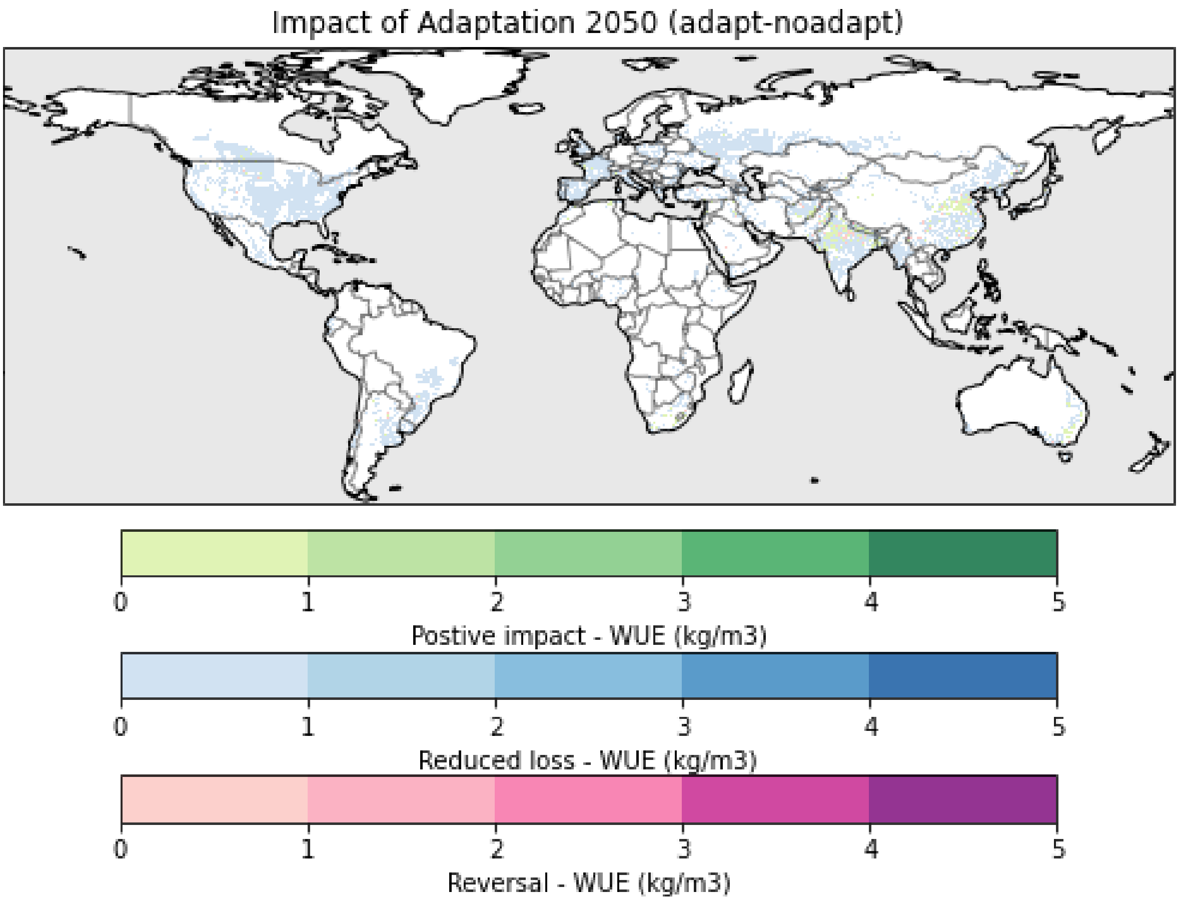

Figure S116: Global irrigated spring wheat water use efficiency: Impact of adaptation on 2050 water use efficiency

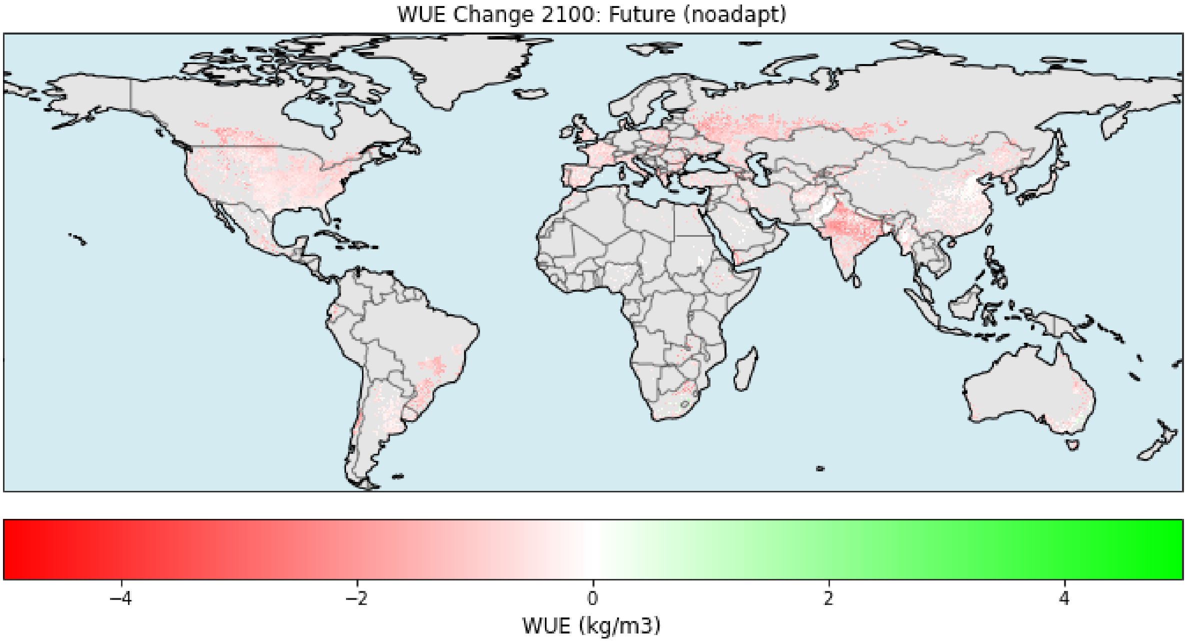

Figure S117: Global irrigated spring wheat water use efficiency: Projected water use efficiency change by 2100 without adaptation

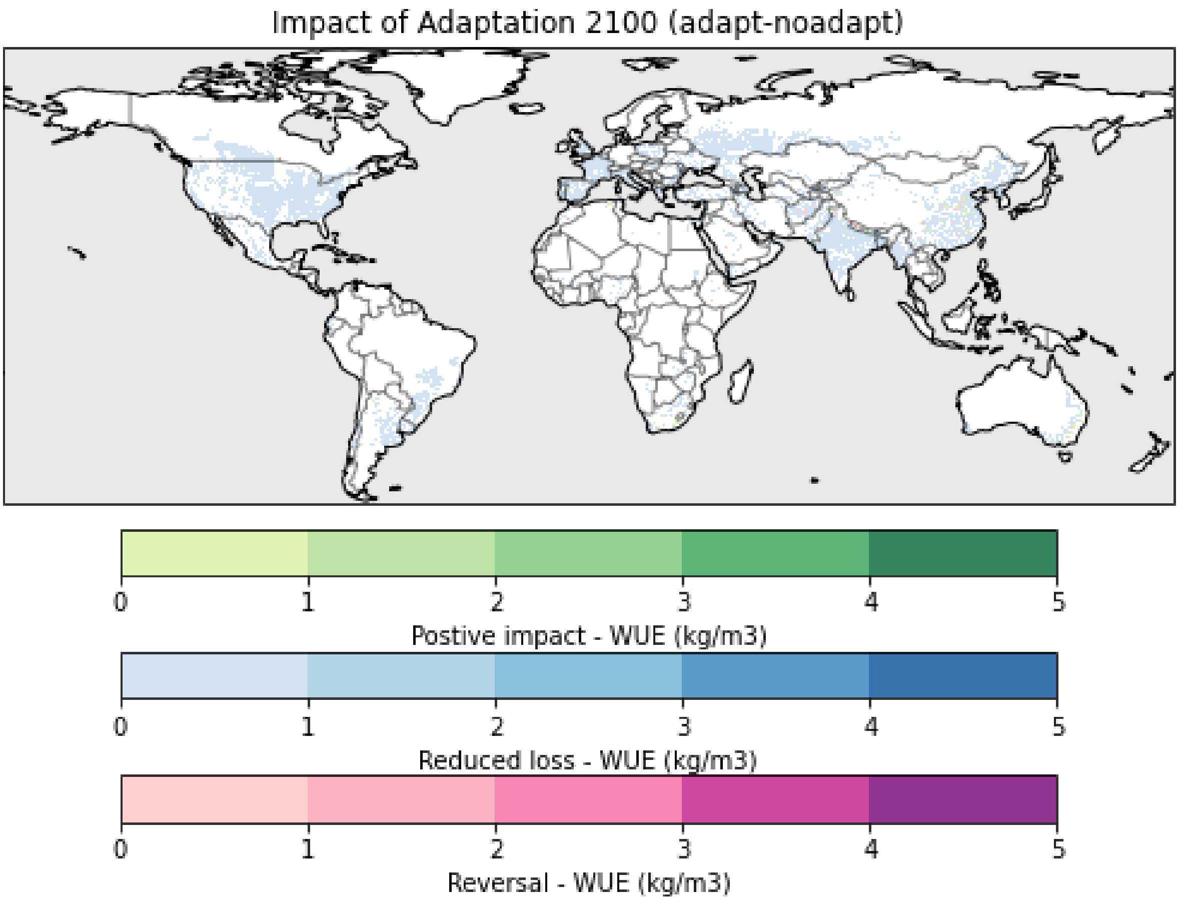

Figure S118: Global irrigated spring wheat water use efficiency: Impact of adaptation on 2100 water use efficiency

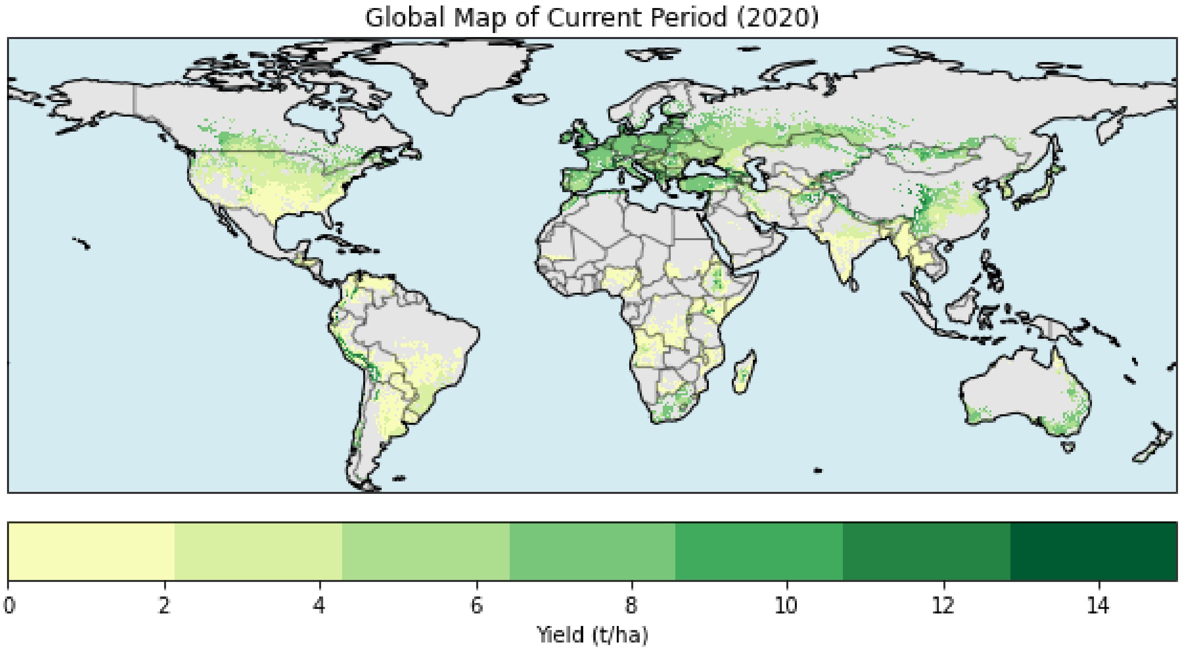

Figure S119: Global rainfed spring wheat productivity: Baseline yield in 2020

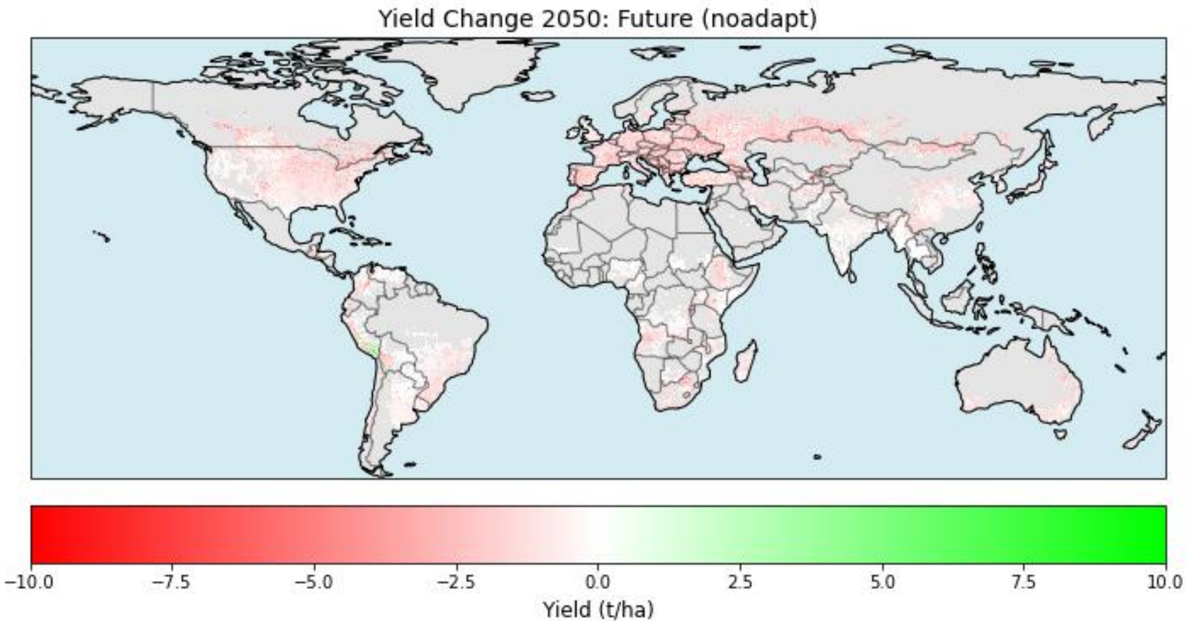

Figure S120: Global rainfed spring wheat productivity: Projected yield change by 2050 without adaptation

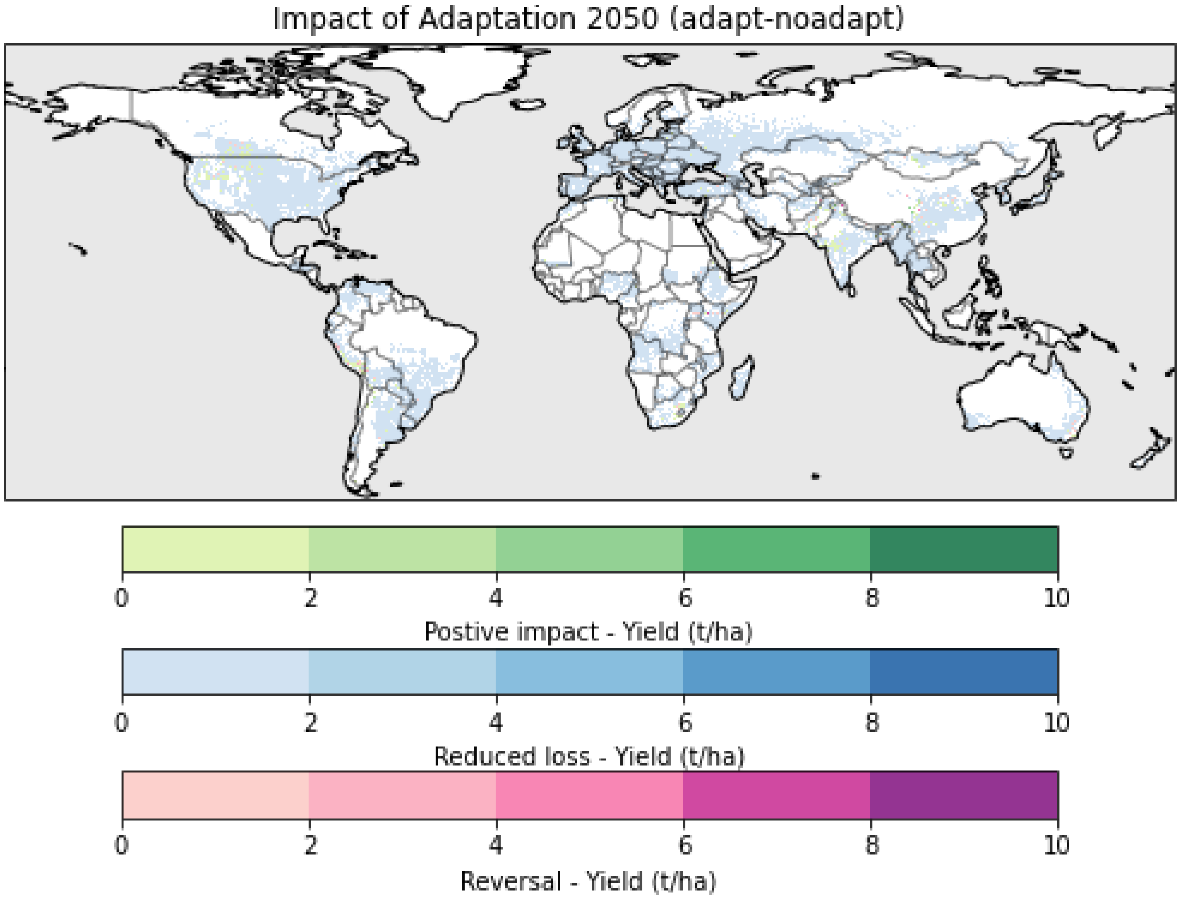

Figure S121: Global rainfed spring wheat productivity: Impact of adaptation on 2050 yields

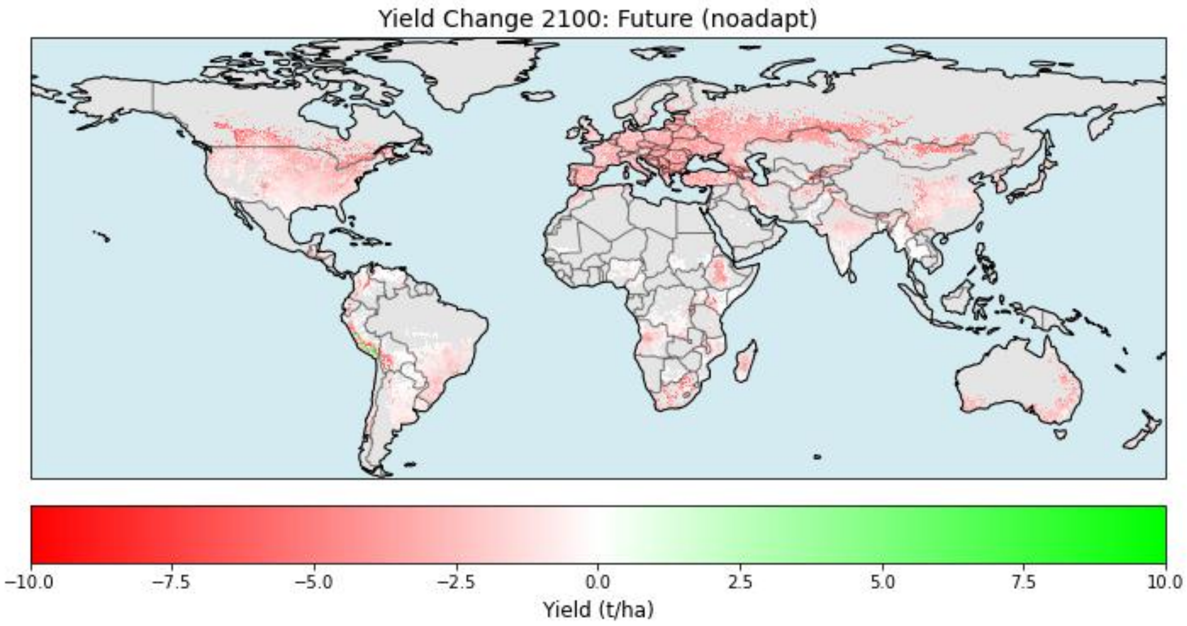

Figure S122: Global rainfed spring wheat productivity: Projected yield change by 2100 without adaptation

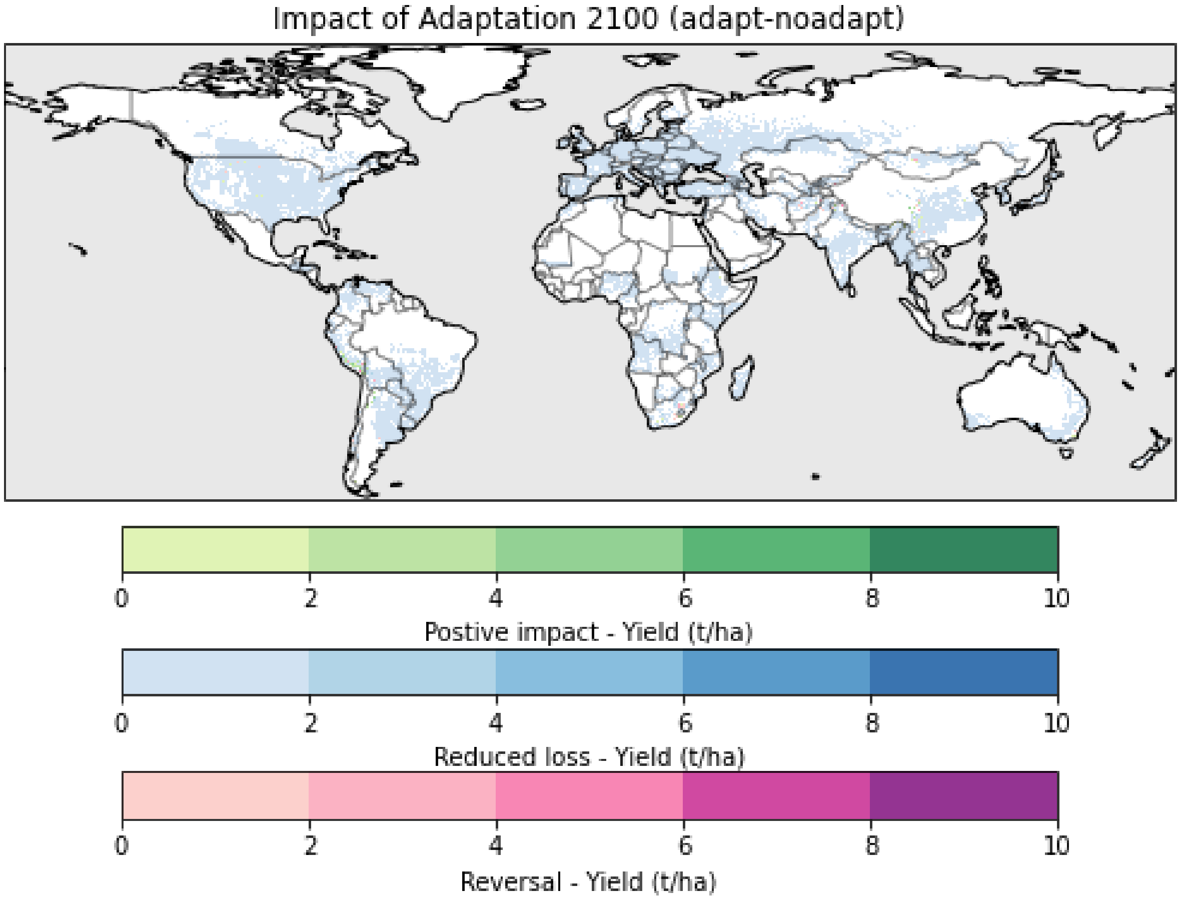

Figure S123: Global rainfed spring wheat productivity: Impact of adaptation on 2100 yields

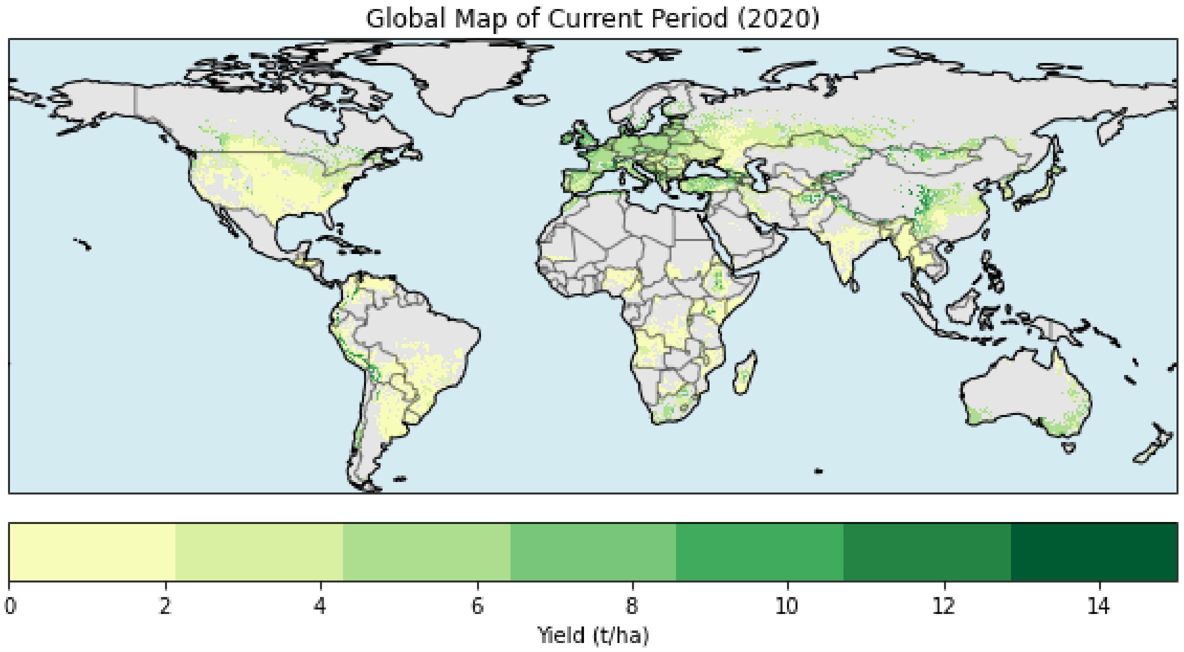

Figure S124: Global rainfed spring wheat reliability: Baseline yield in 2020

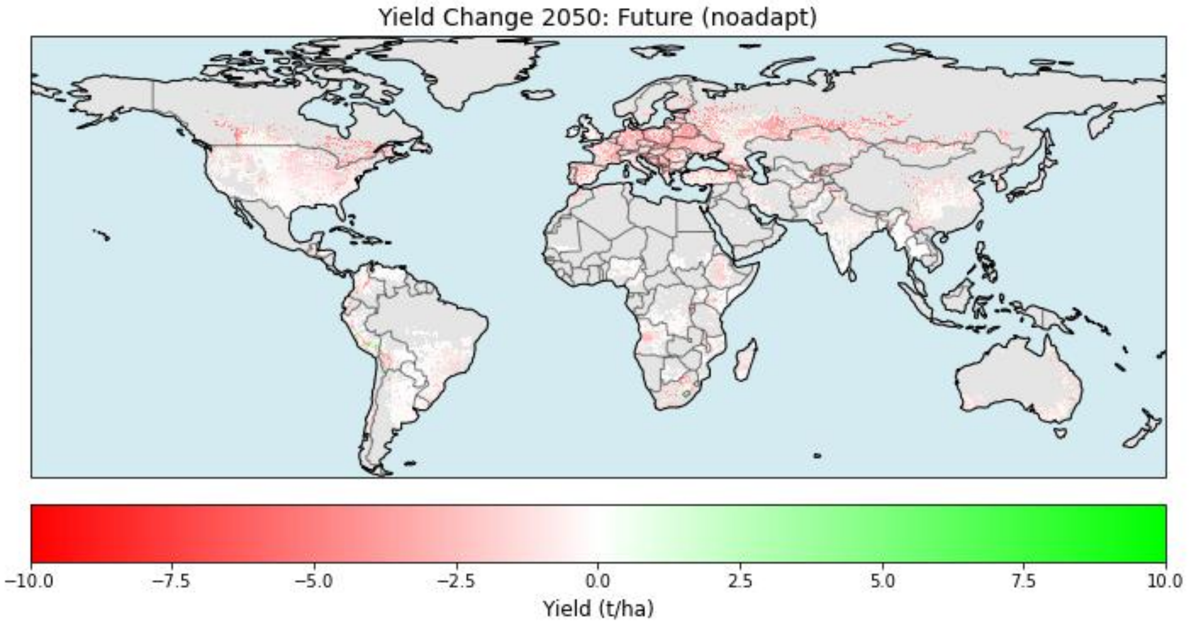

Figure S125: Global rainfed spring wheat reliability: Projected yield change by 2050 without adaptation

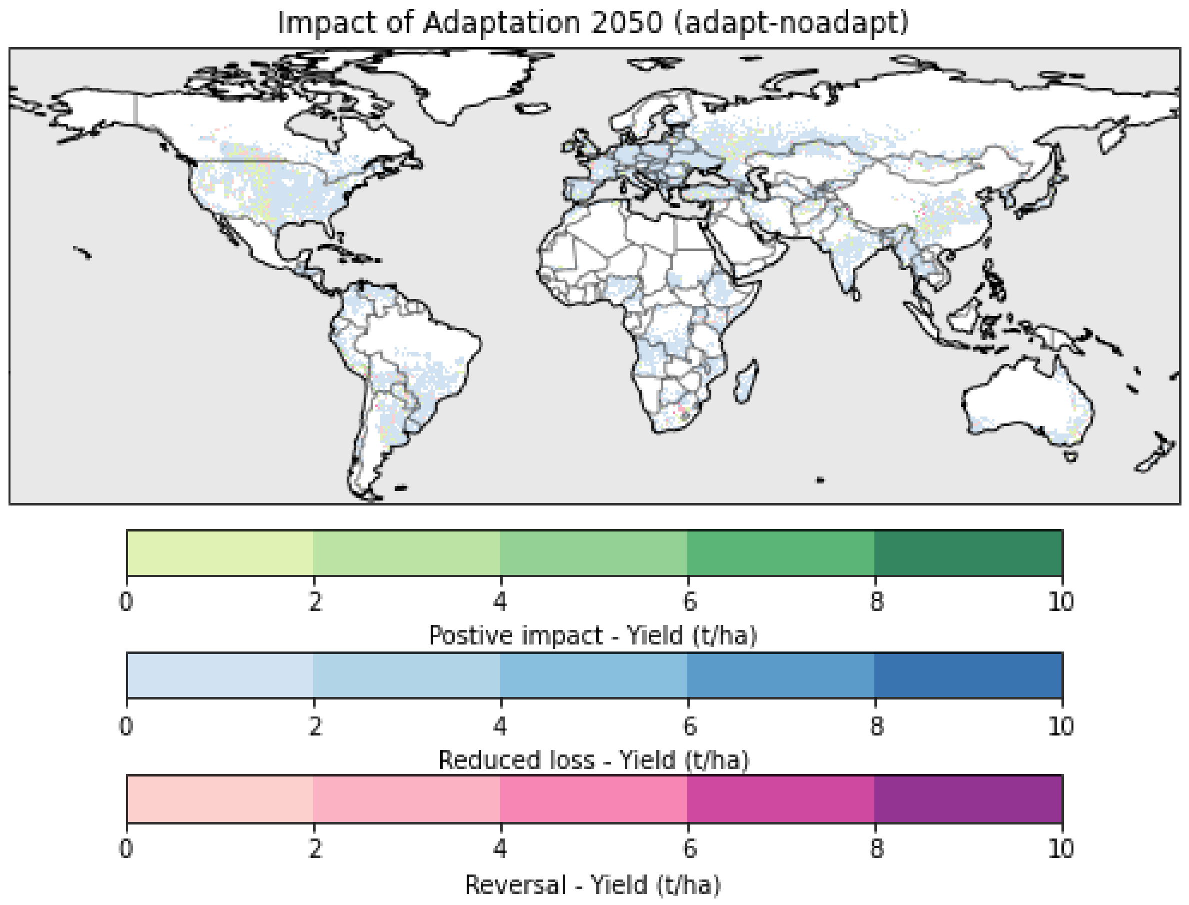

Figure S126: Global rainfed spring wheat reliability: Impact of adaptation on 2050 yields

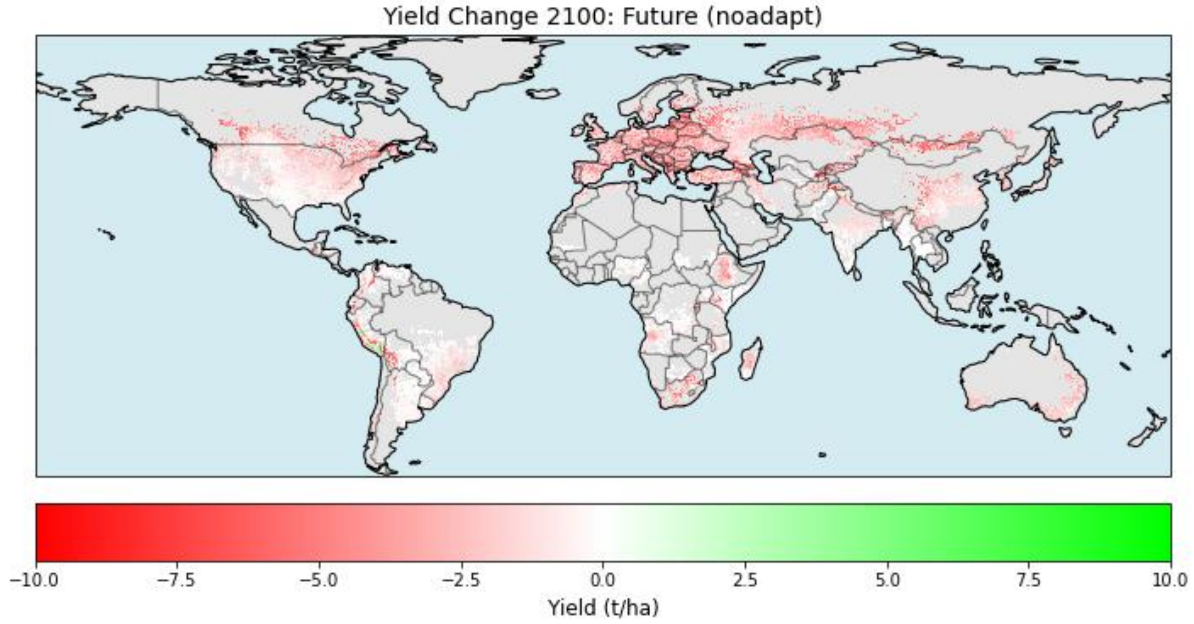

Figure S127: Global rainfed spring wheat reliability: Projected yield change by 2100 without adaptation

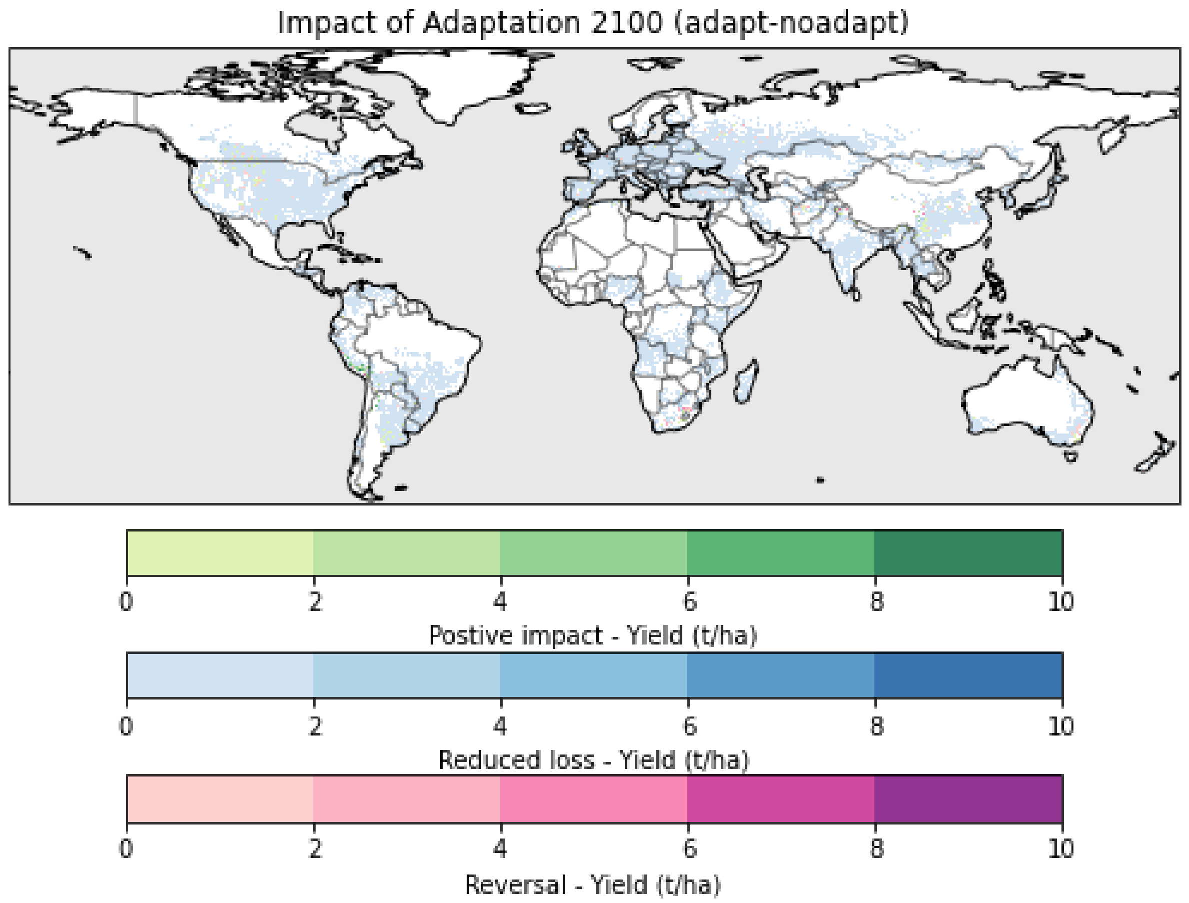

Figure S128: Global rainfed spring wheat reliability: Impact of adaptation on 2100 yields

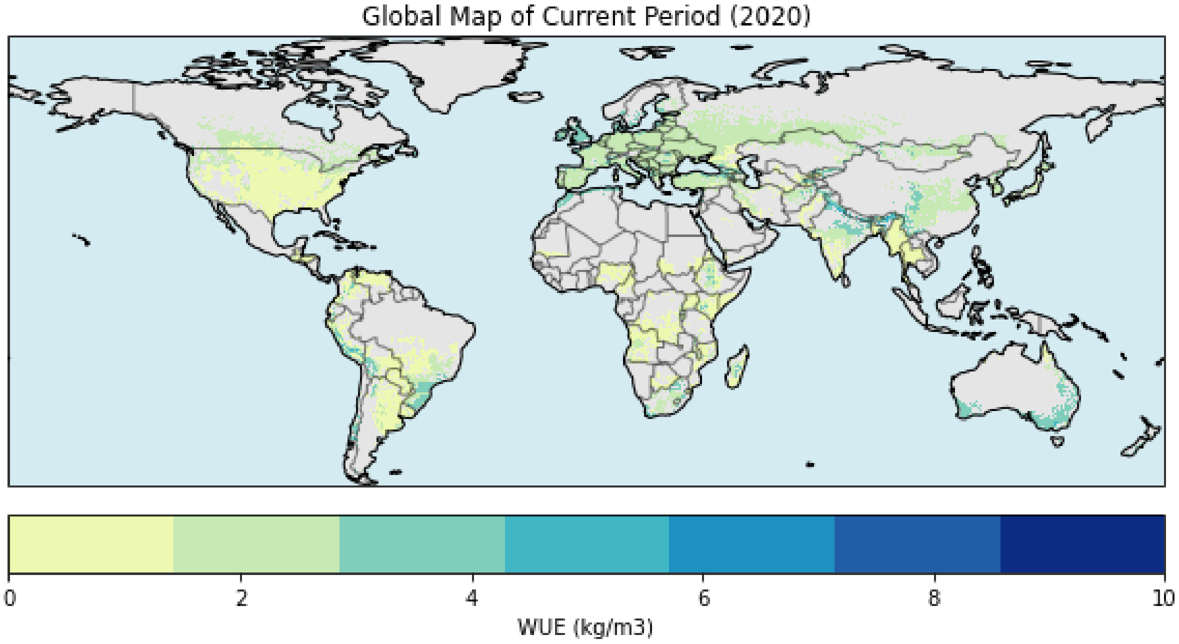

Figure S129: Global rainfed spring wheat water use efficiency: Baseline water use efficiency in 2020

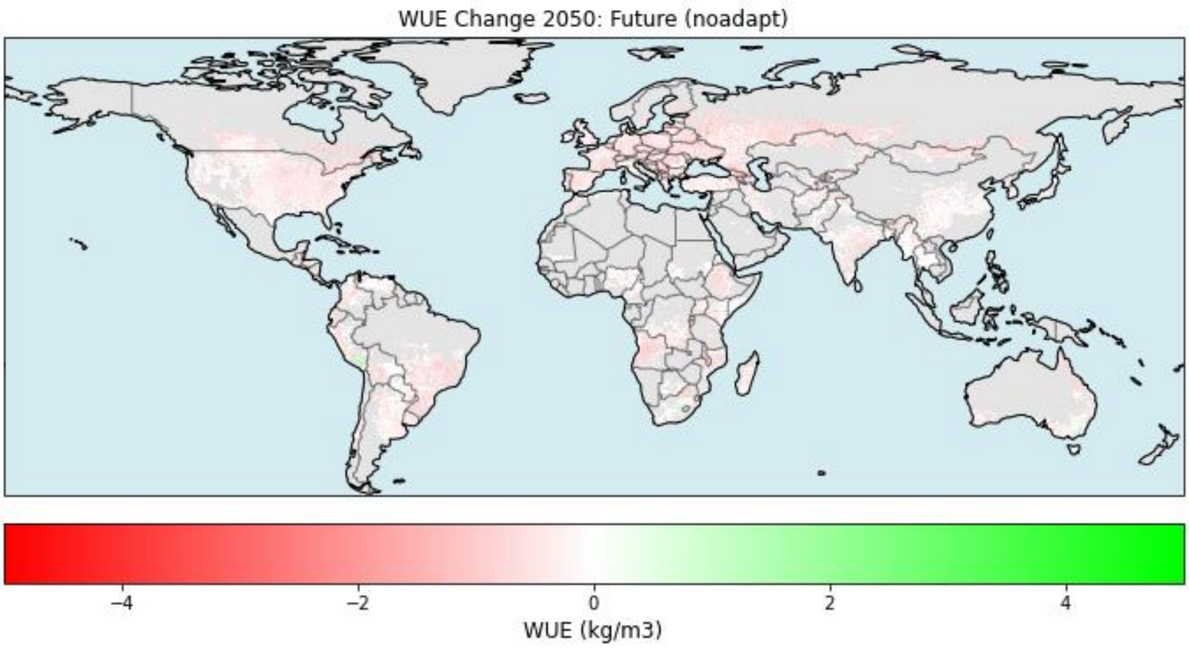

Figure S130: Global rainfed spring wheat water use efficiency: Projected water use efficiency change by 2050 without adaptation

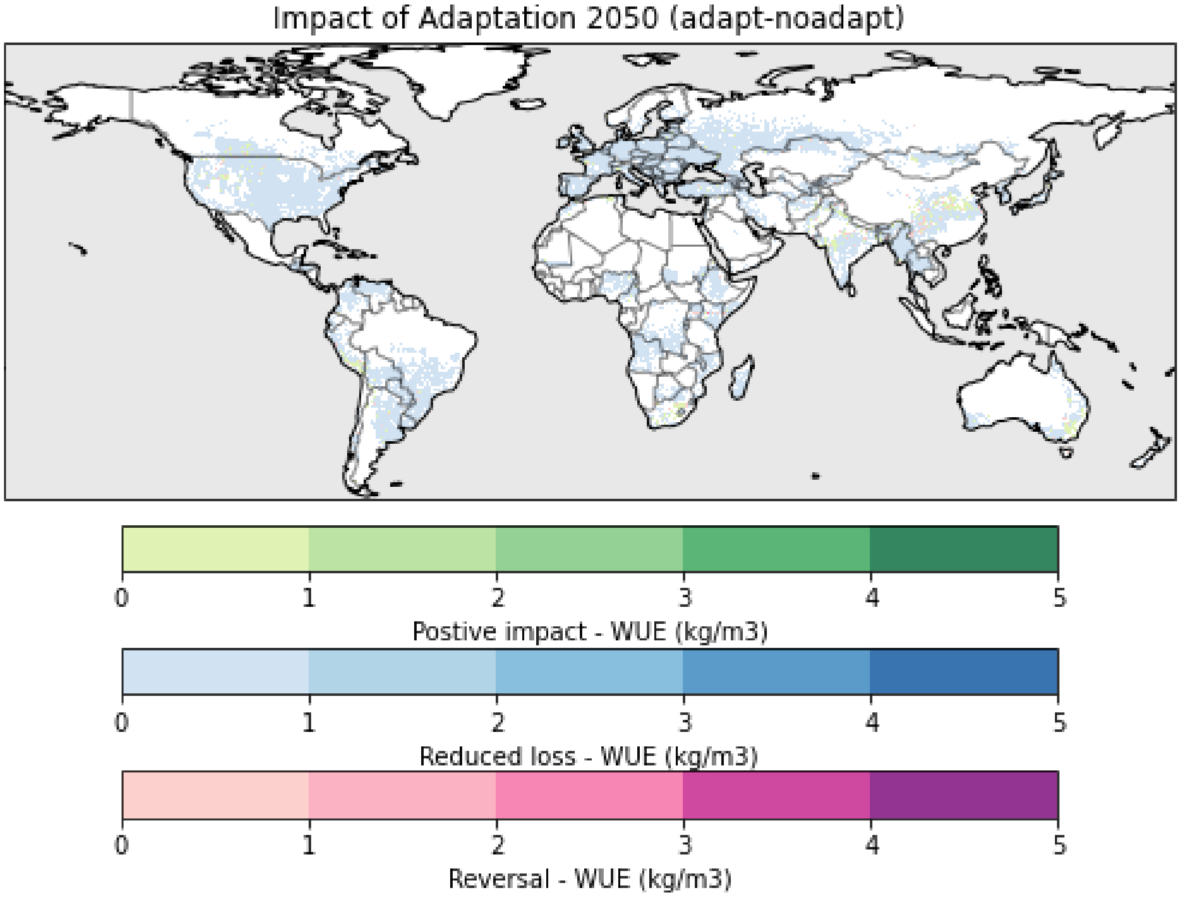

Figure S131: Global rainfed spring wheat water use efficiency: Impact of adaptation on 2050 water use efficiency

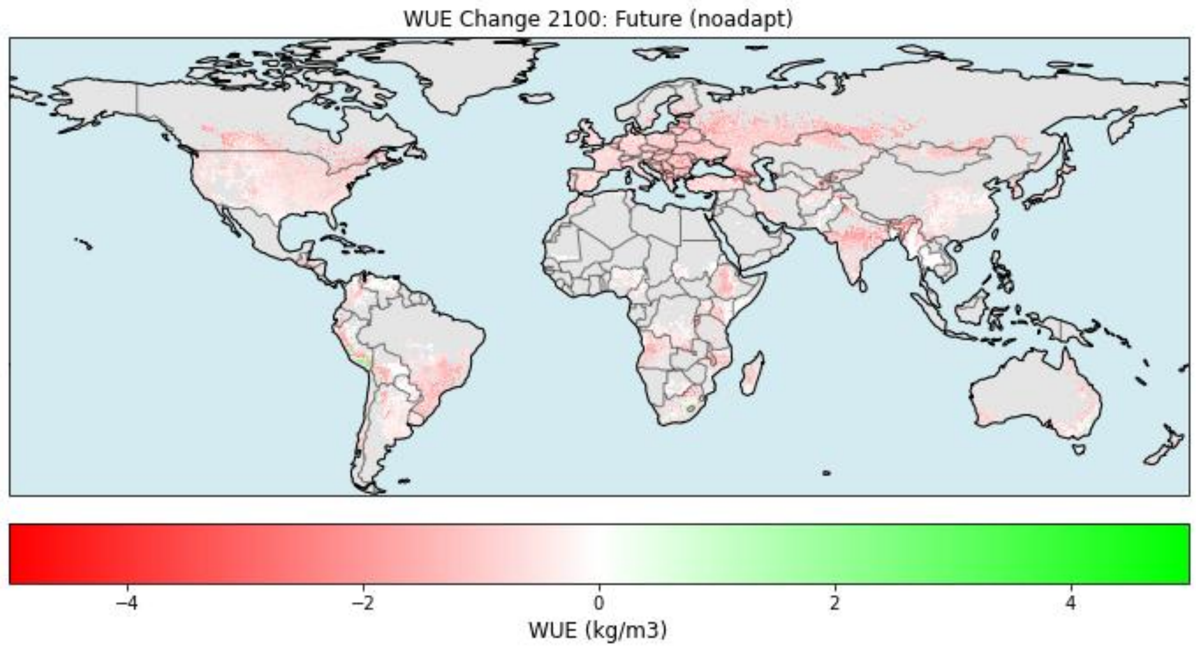

Figure S132: Global rainfed spring wheat water use efficiency: Projected water use efficiency change by 2100 without adaptation

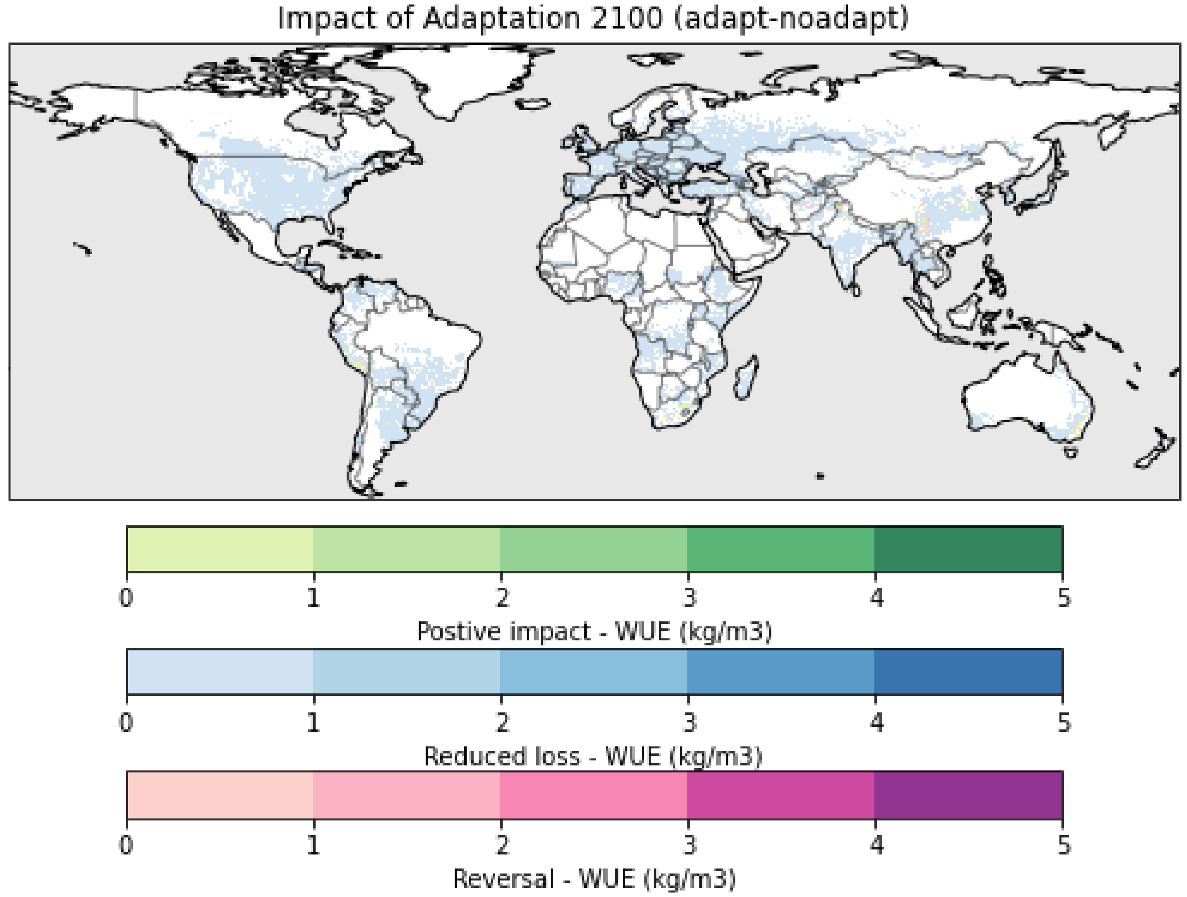

Figure S133: Global rainfed spring wheat water use efficiency: Impact of adaptation on 2100 water use efficiency

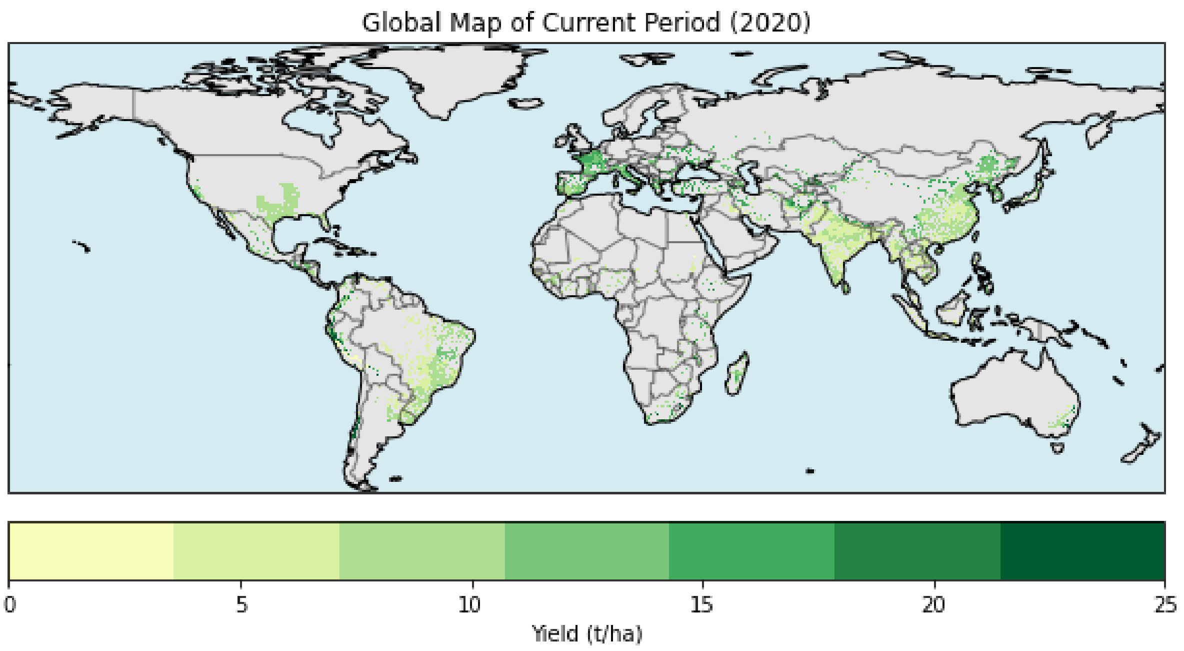

Figure S134: Global irrigated rice (season-1) productivity: Baseline yield in 2020

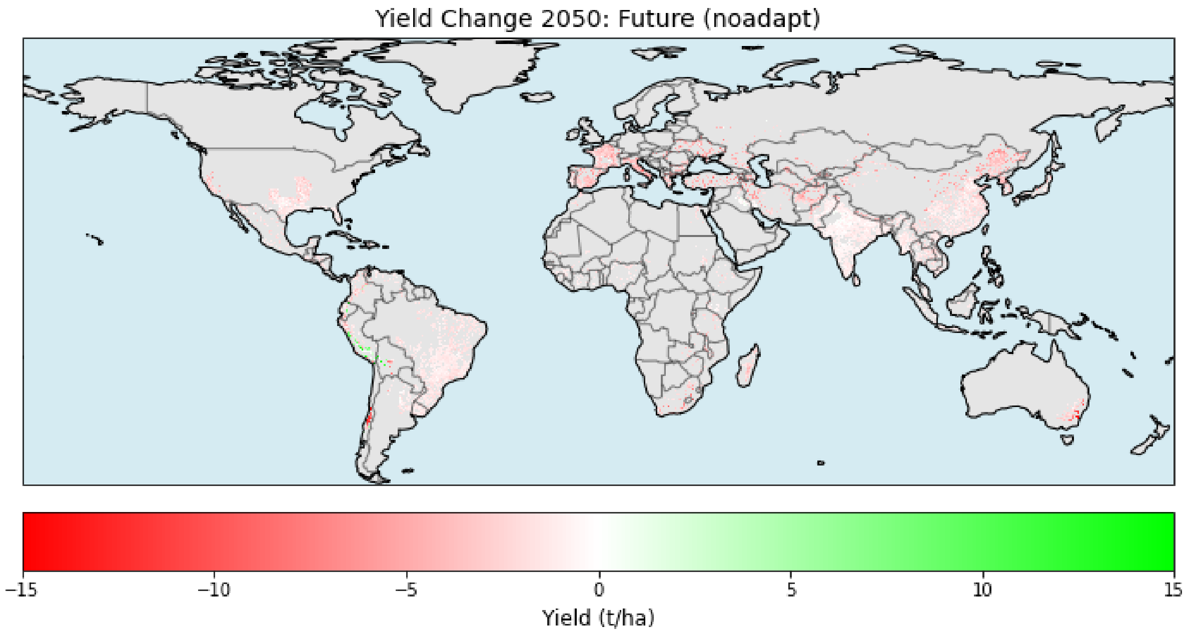

Figure S135: Global irrigated rice (season-1) productivity: Projected yield change by 2050 without adaptation

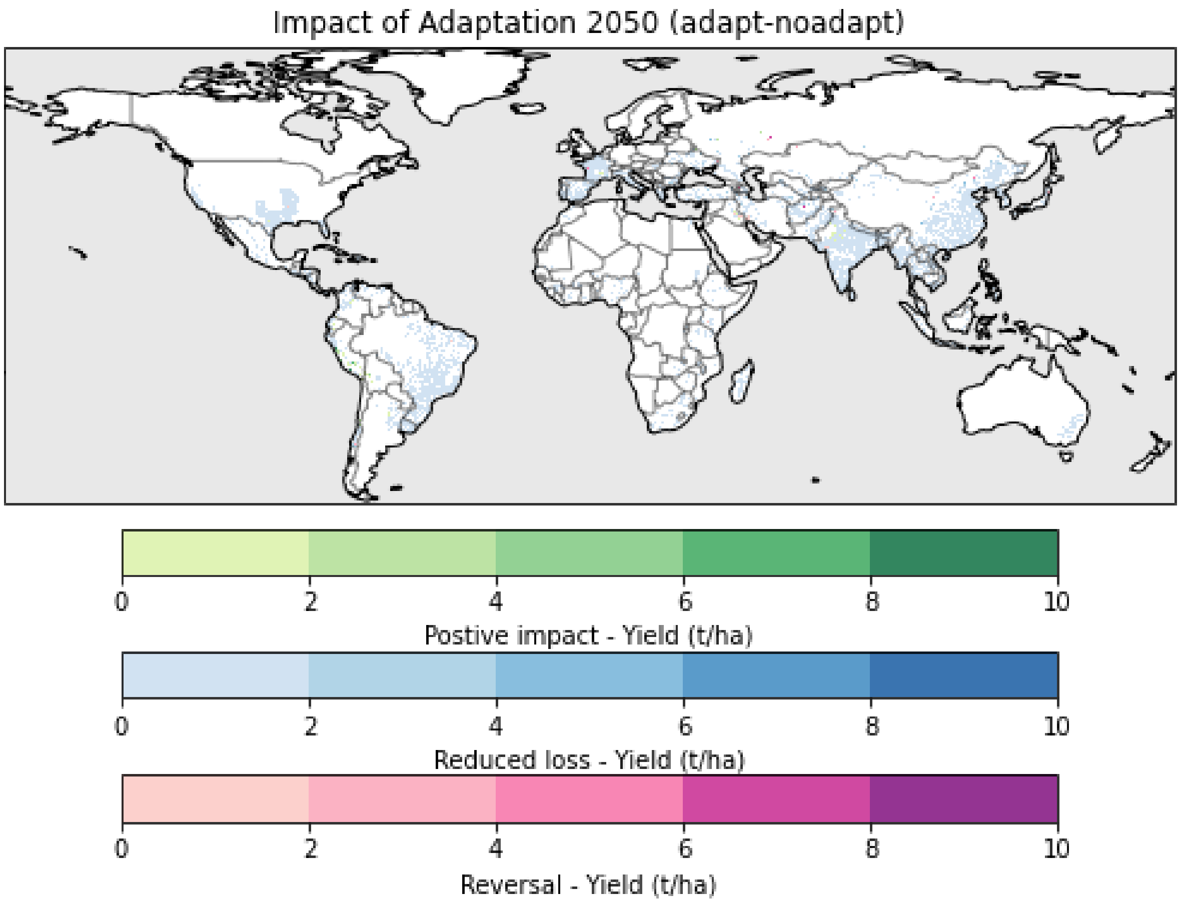

Figure S136: Global irrigated rice (season-1) productivity: Impact of adaptation on 2050 yields

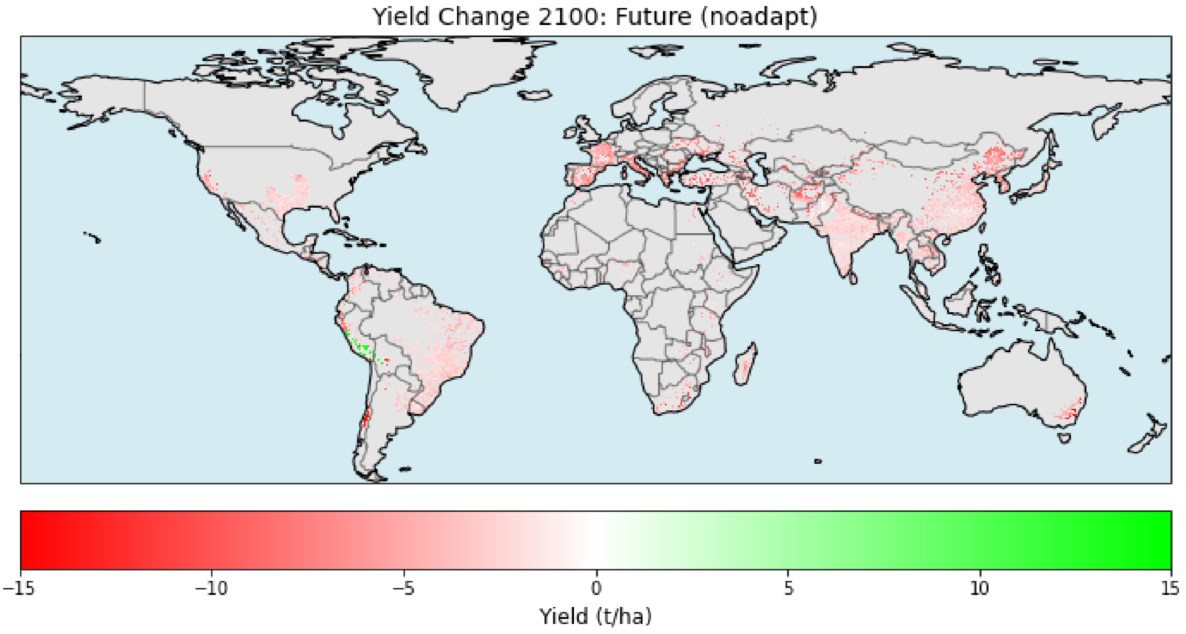

Figure S137: Global irrigated rice (season-1) productivity: Projected yield change by 2100 without adaptation

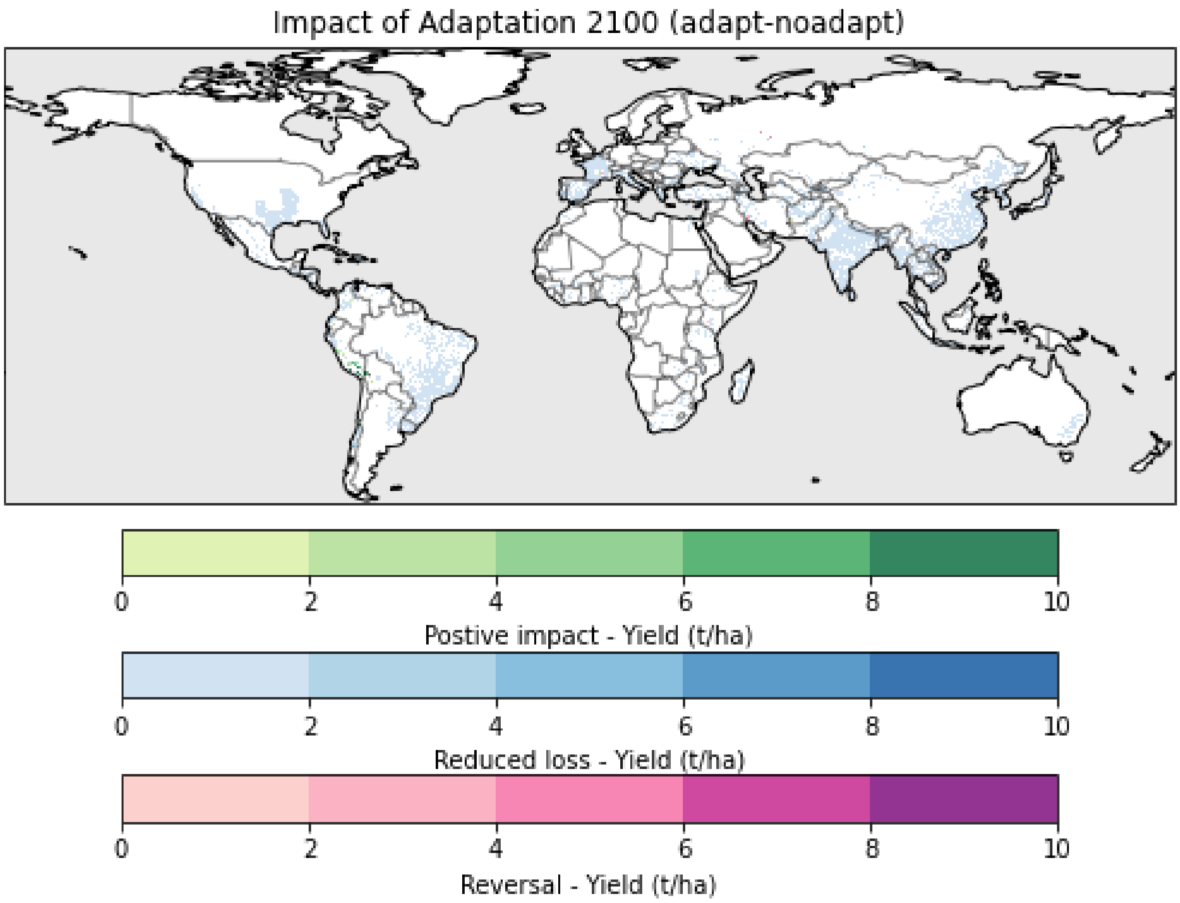

Figure S138: Global irrigated rice (season-1) productivity: Impact of adaptation on 2100 yields

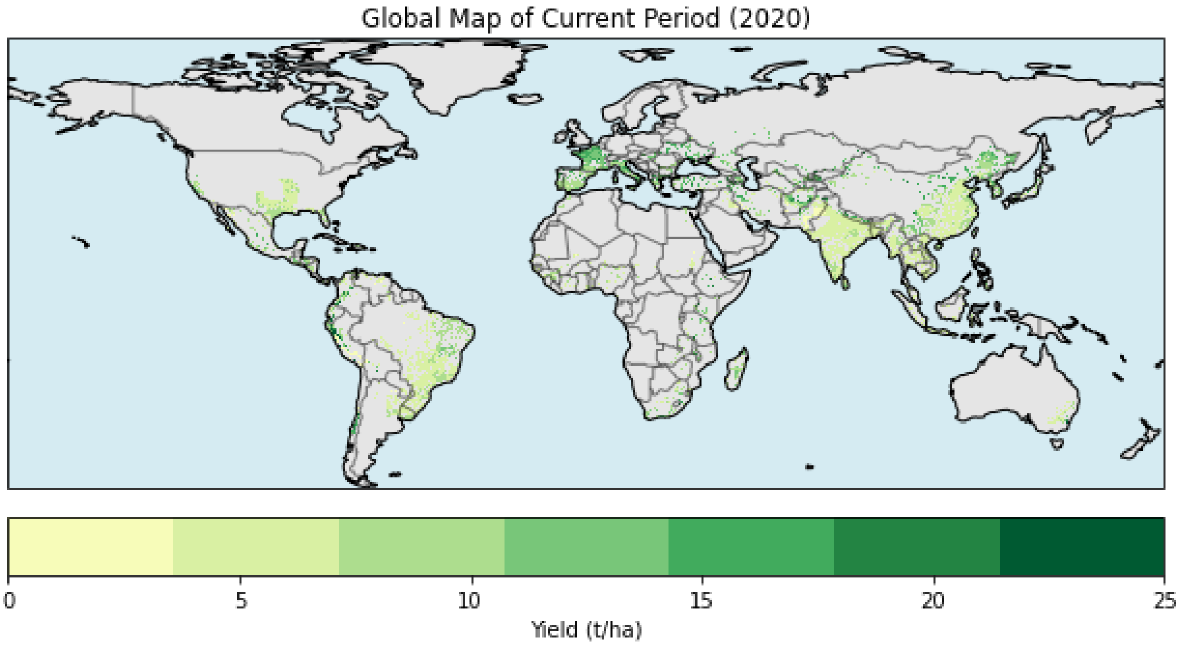

Figure S139: Global irrigated rice (season-1) reliability: Baseline yield in 2020

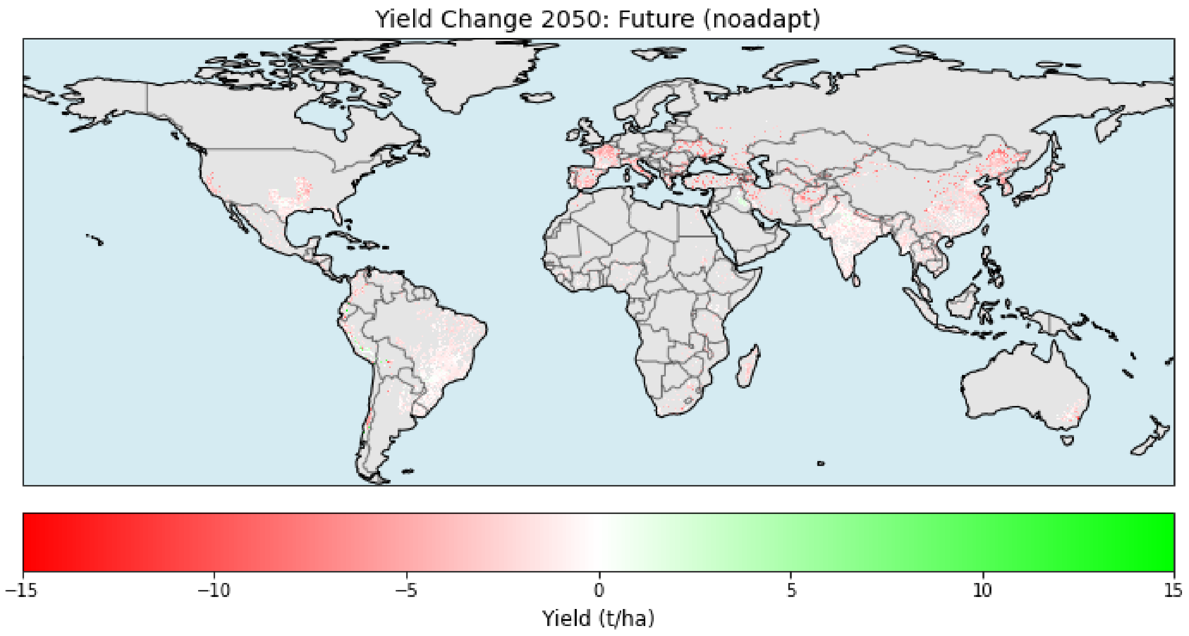

Figure S140: Global irrigated rice (season-1) reliability: Projected yield change by 2050 without adaptation

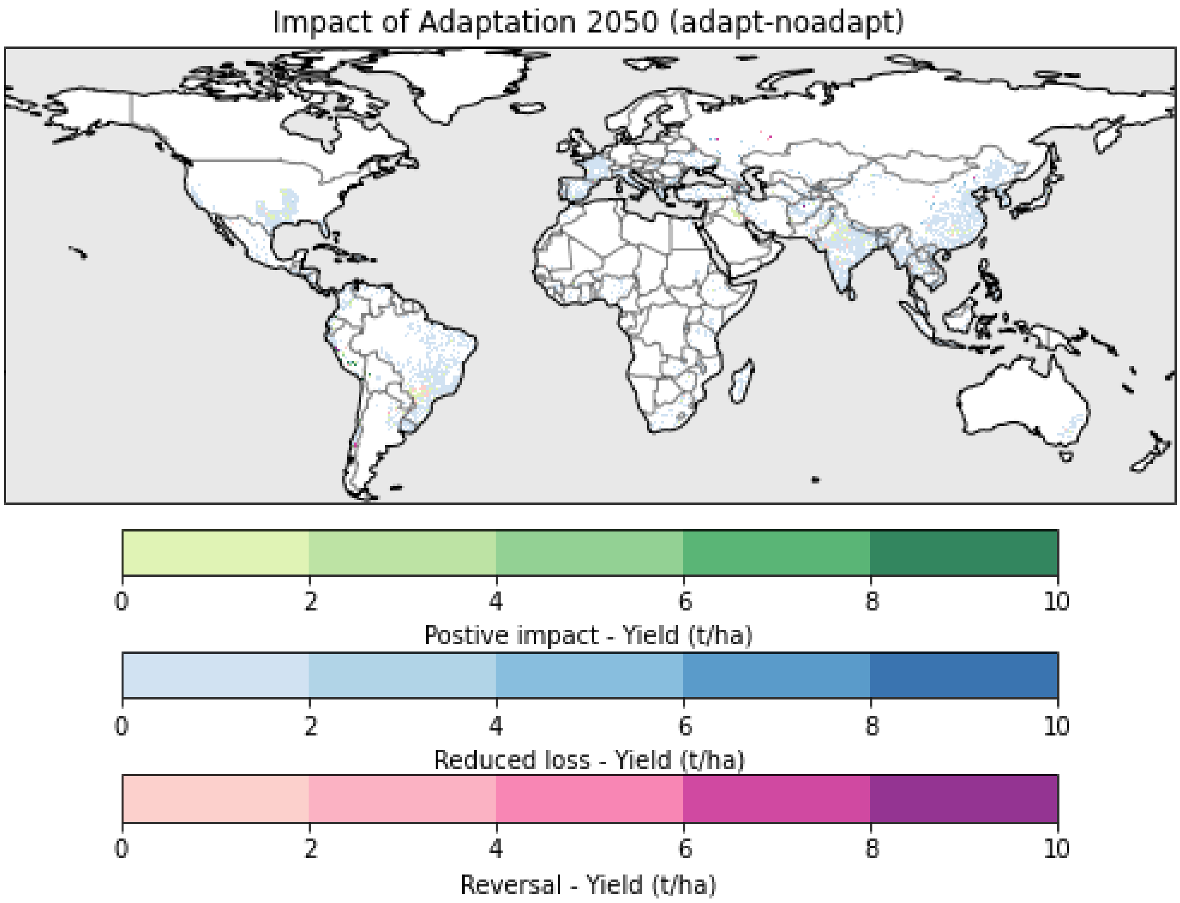

Figure S141: Global irrigated rice (season-1) reliability: Impact of adaptation on 2050 yields

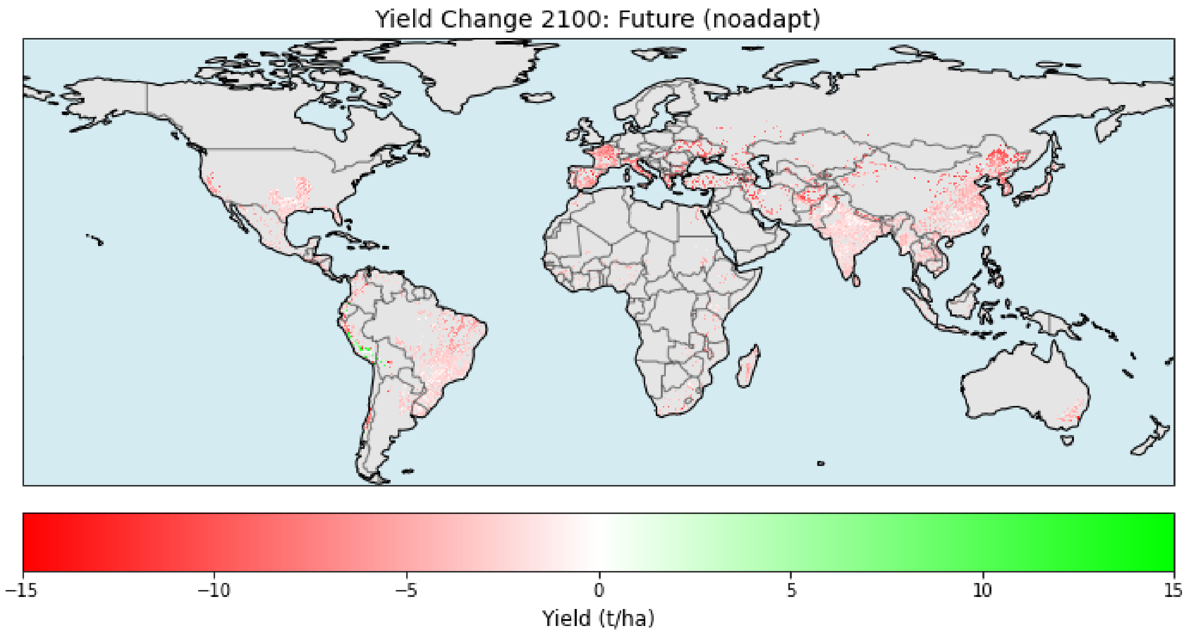

Figure S142: Global irrigated rice (season-1) reliability: Projected yield change by 2100 without adaptation

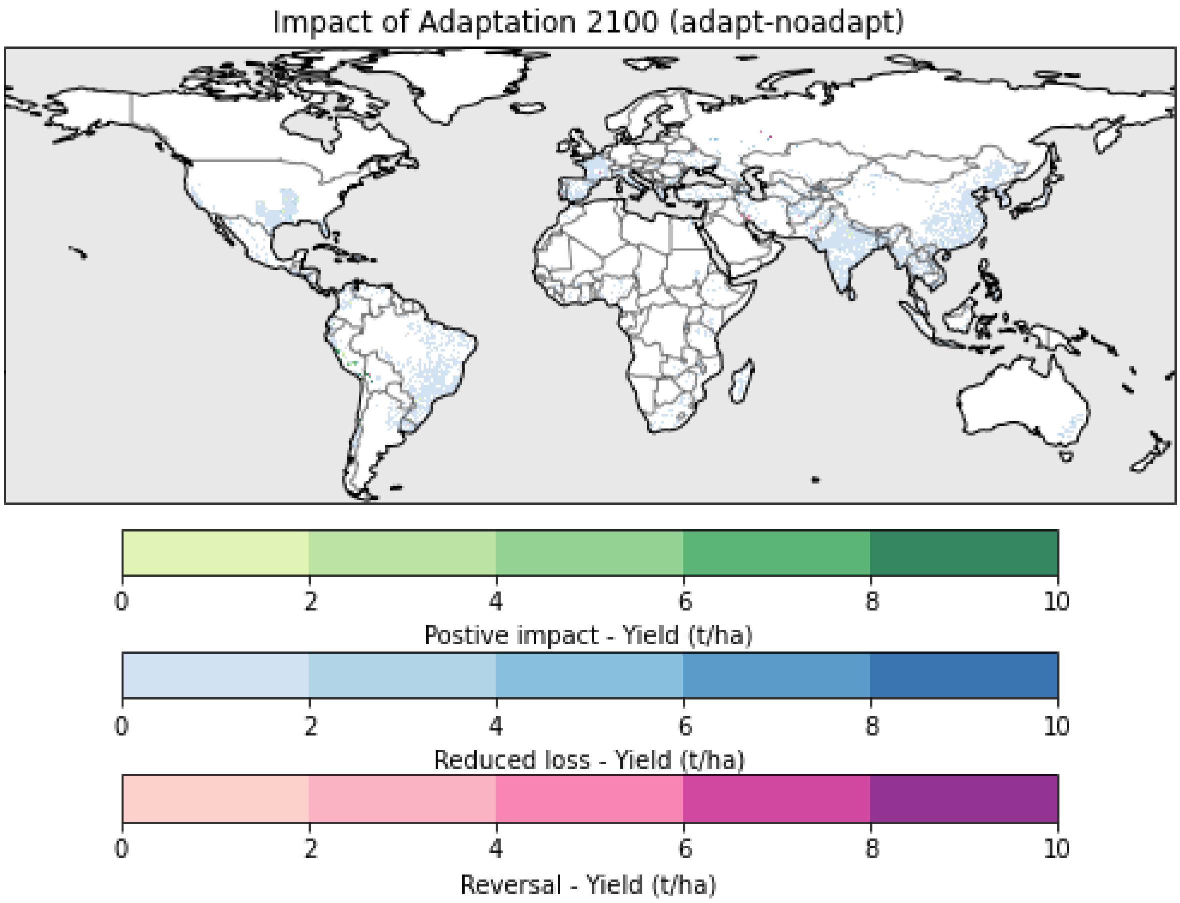

Figure S143: Global irrigated rice (season-1) reliability: Impact of adaptation on 2100 yields

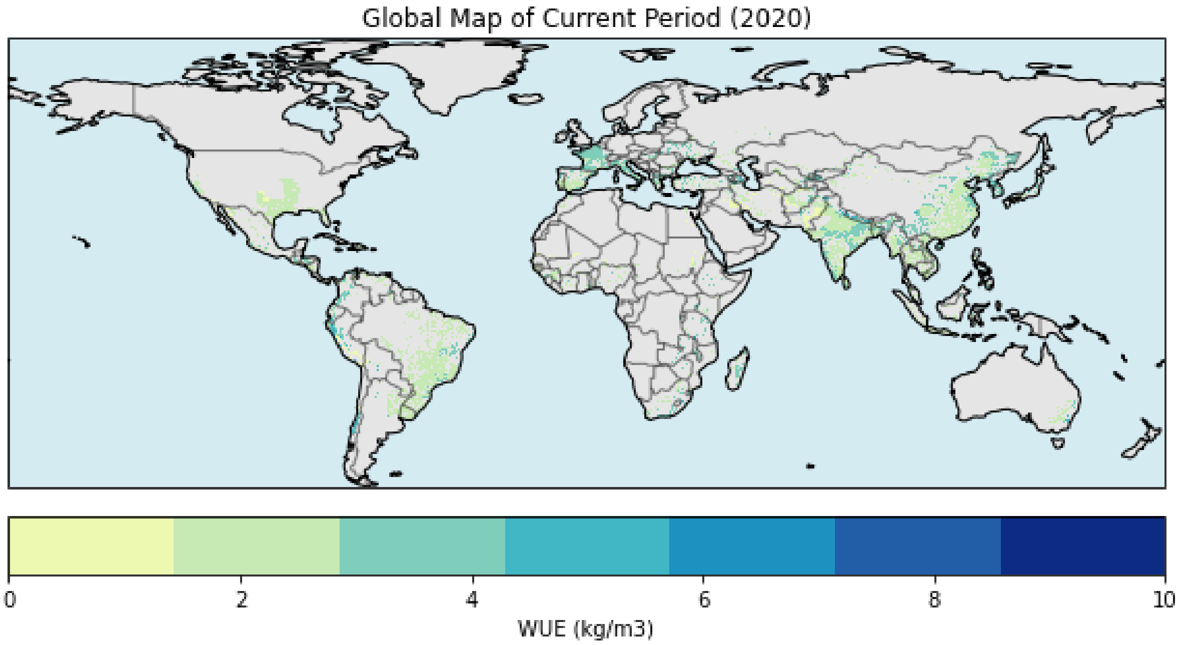

Figure S144: Global irrigated rice (season-1) water use efficiency: Baseline water use efficiency in 2020

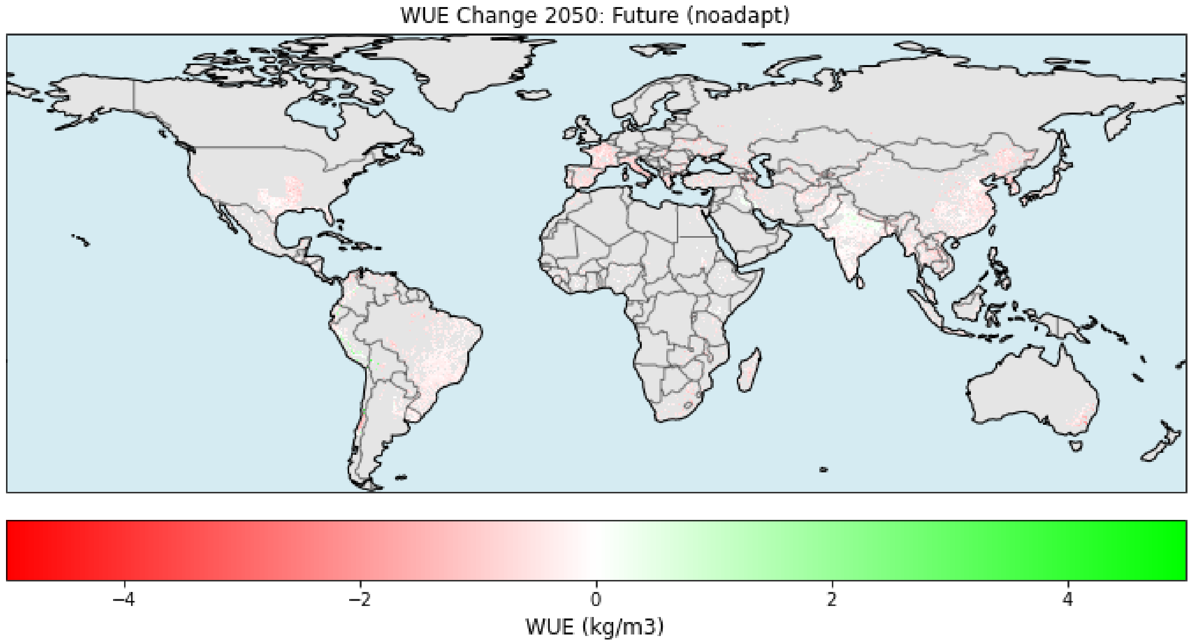

Figure S145: Global irrigated rice (season-1) water use efficiency: Projected water use efficiency change by 2050 without adaptation

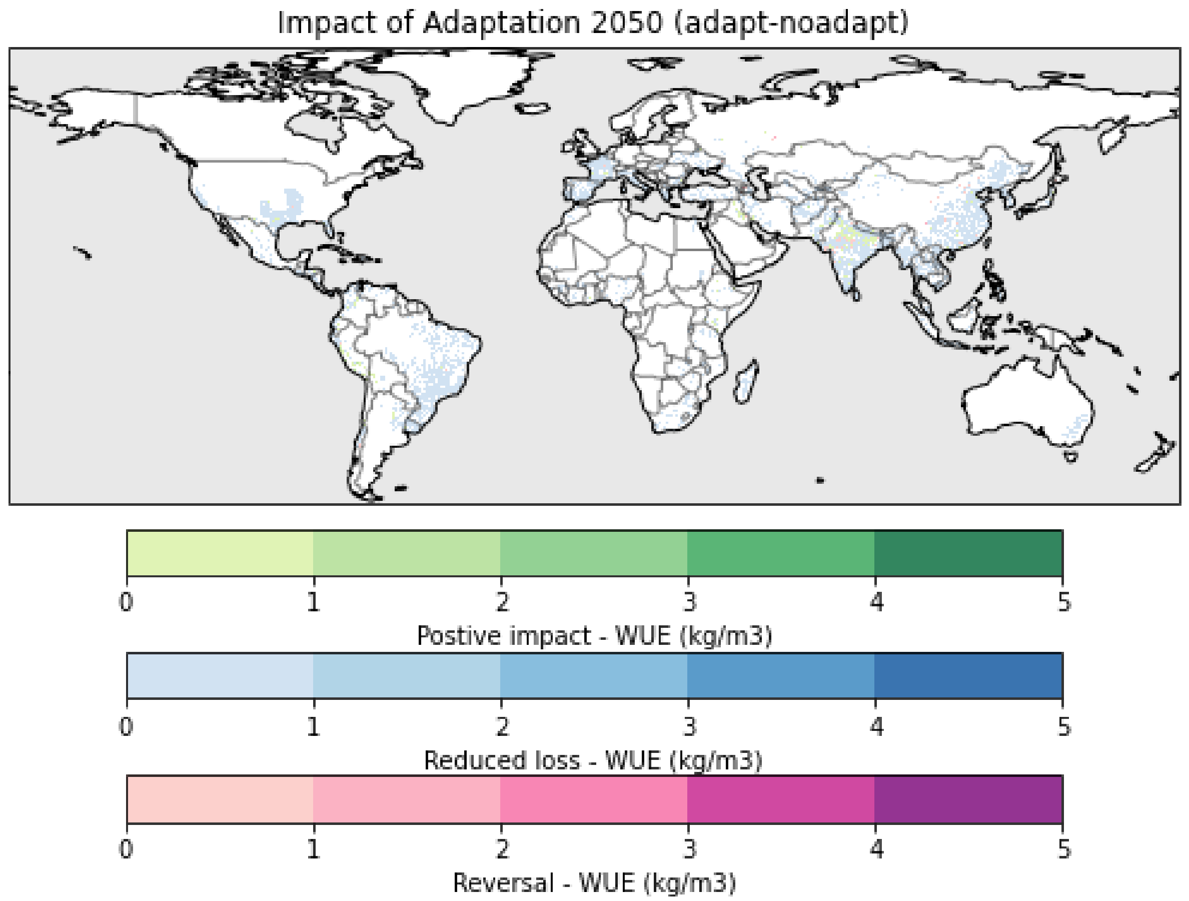

Figure S146: Global irrigated rice (season-1) water use efficiency: Impact of adaptation on 2050 water use efficiency

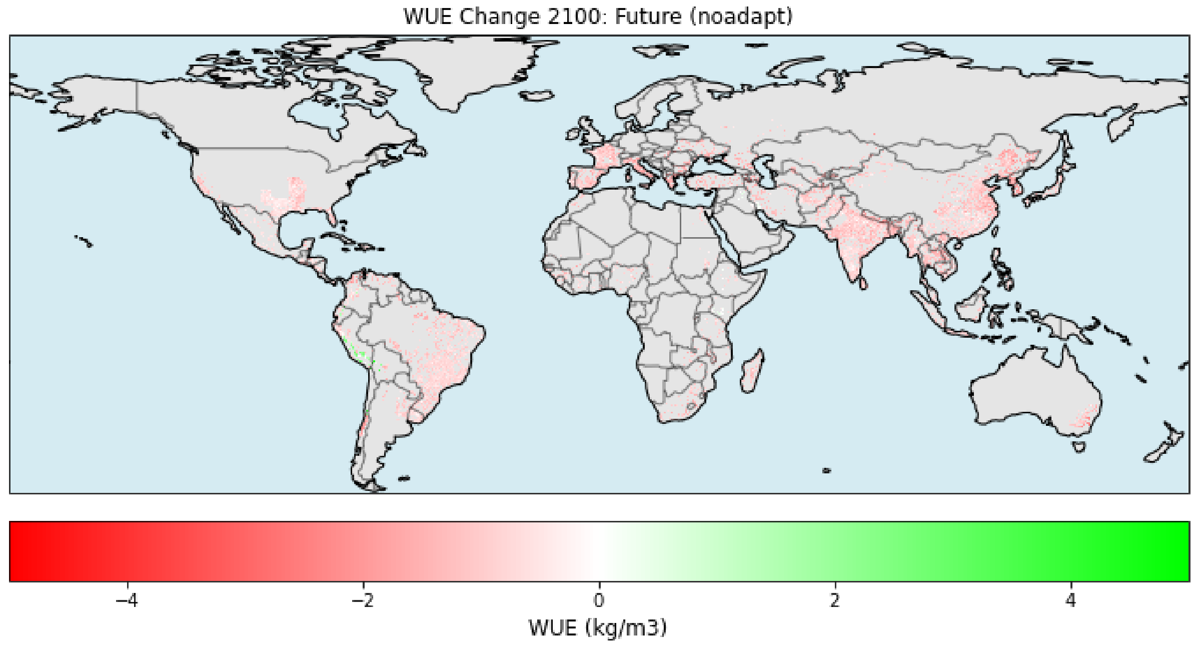

Figure S147: Global irrigated rice (season-1) water use efficiency: Projected water use efficiency change by 2100 without adaptation

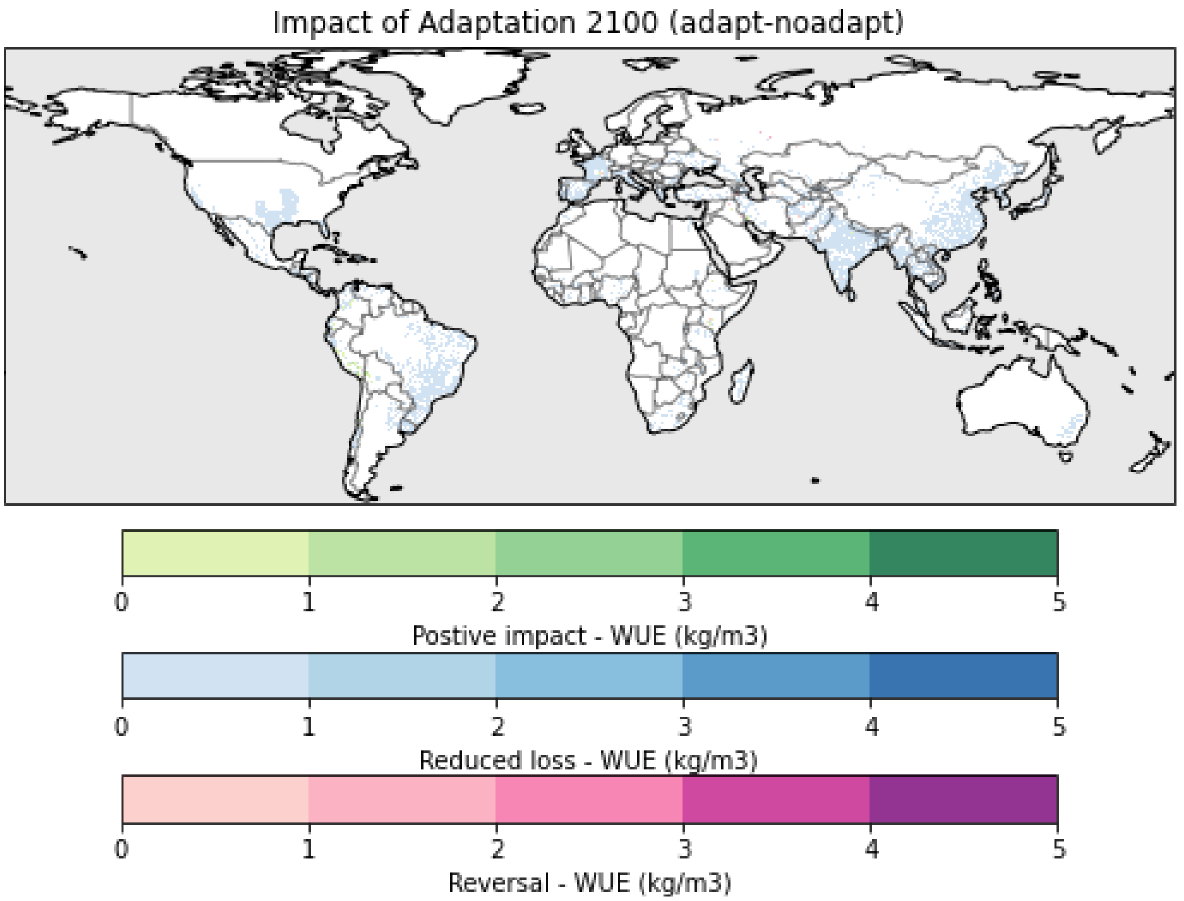

Figure S148: Global irrigated rice (season-1) water use efficiency: Impact of adaptation on 2100 water use efficiency

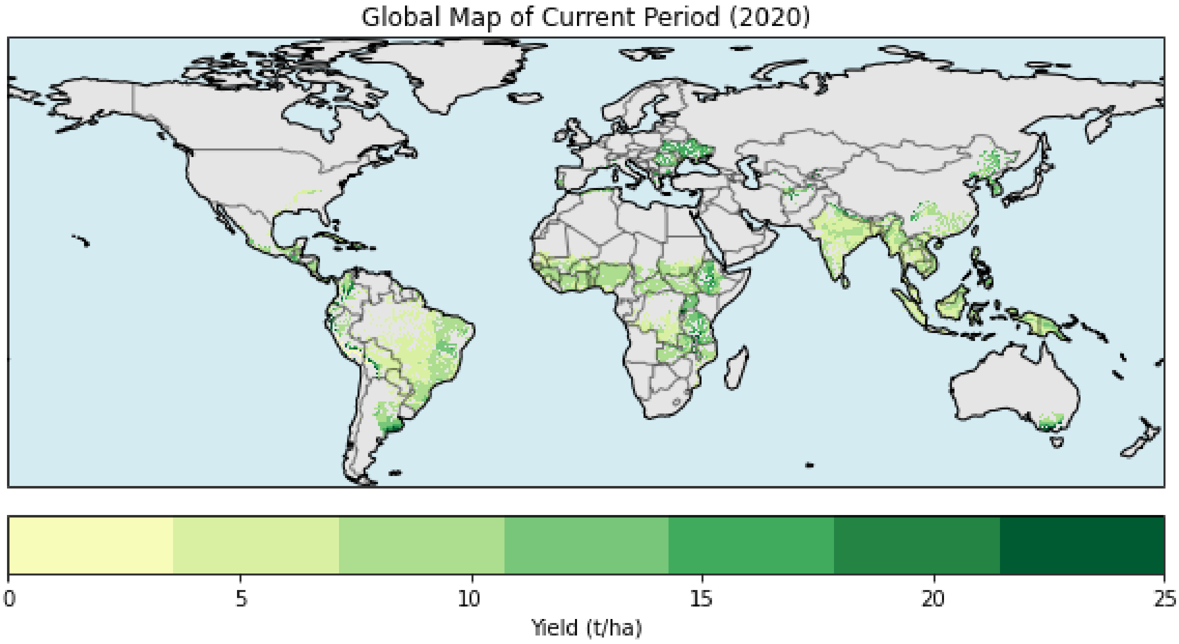

Figure S149: Global rainfed rice (season-1) productivity: Baseline yield in 2020

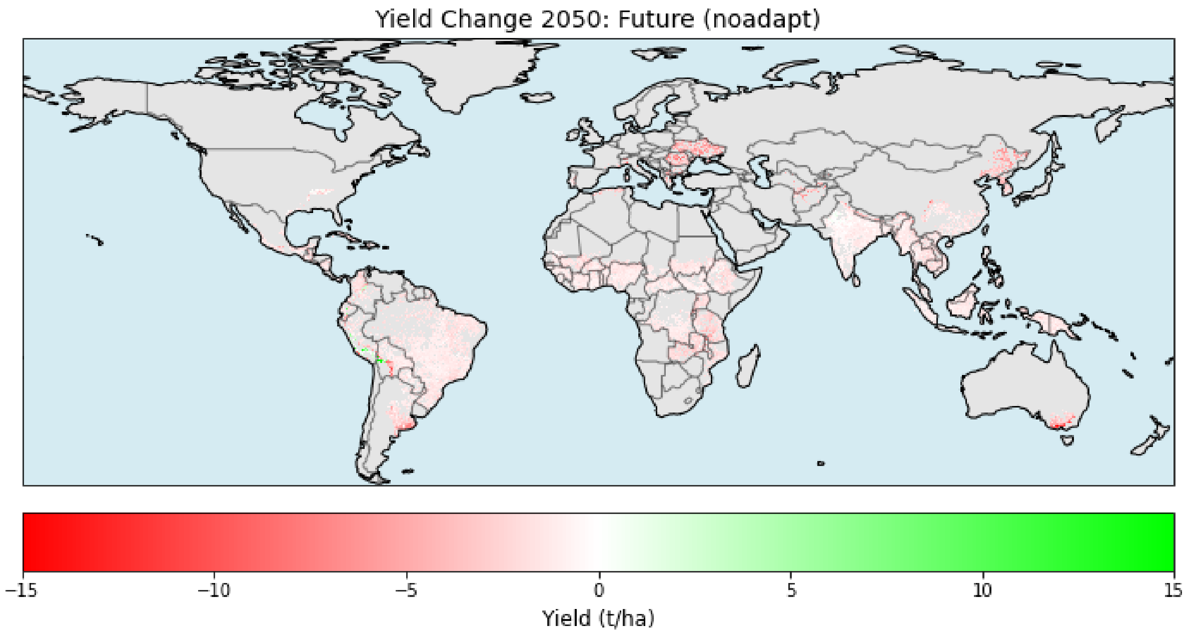

Figure S150: Global rainfed rice (season-1) productivity: Projected yield change by 2050 without adaptation

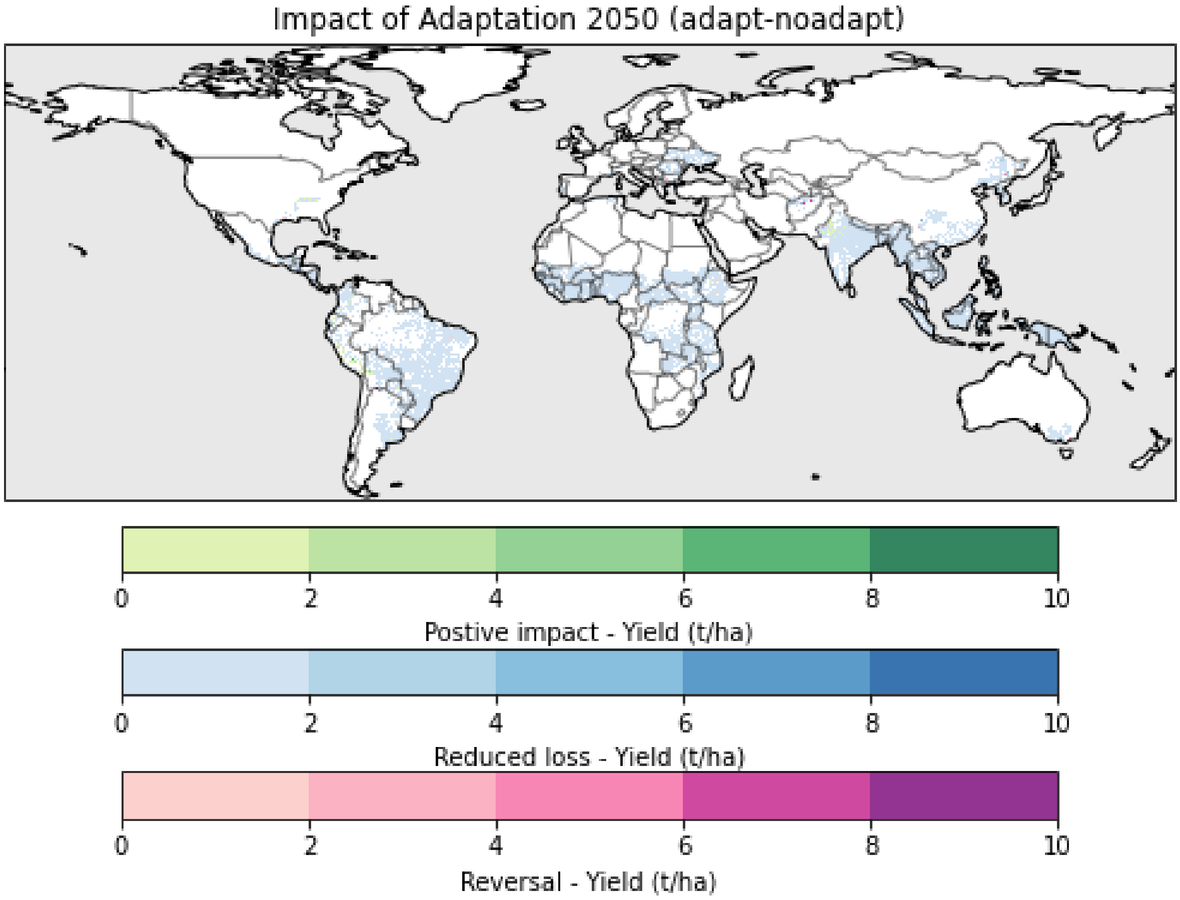

Figure S151: Global rainfed rice (season-1) productivity: Impact of adaptation on 2050 yields

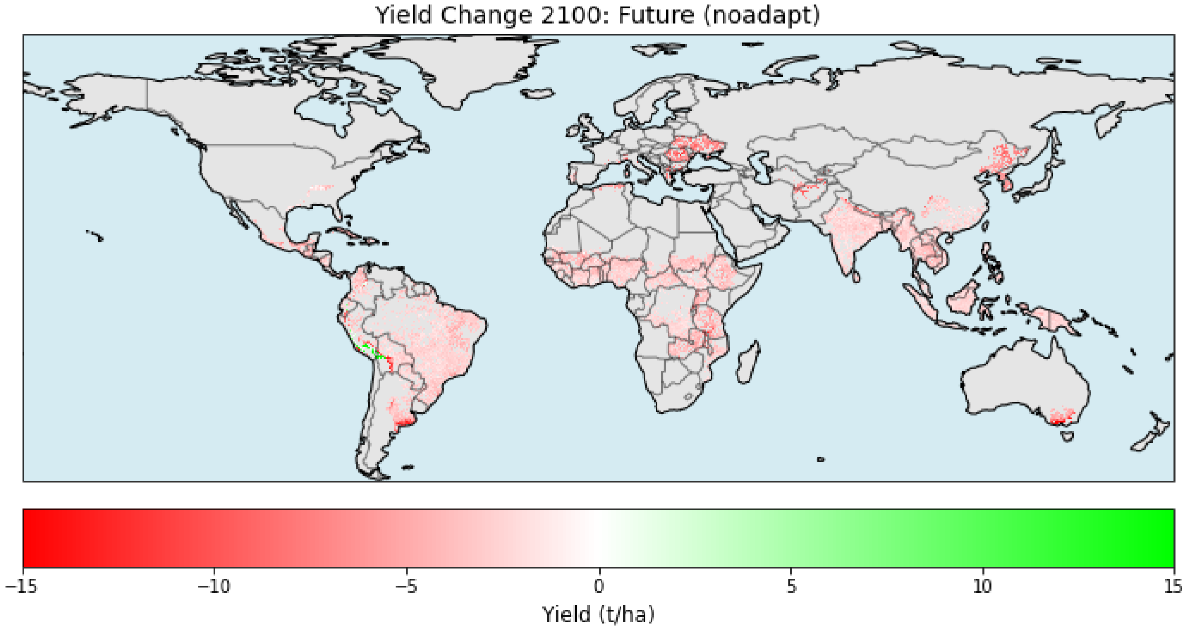

Figure S152: Global rainfed rice (season-1) productivity: Projected yield change by 2100 without adaptation

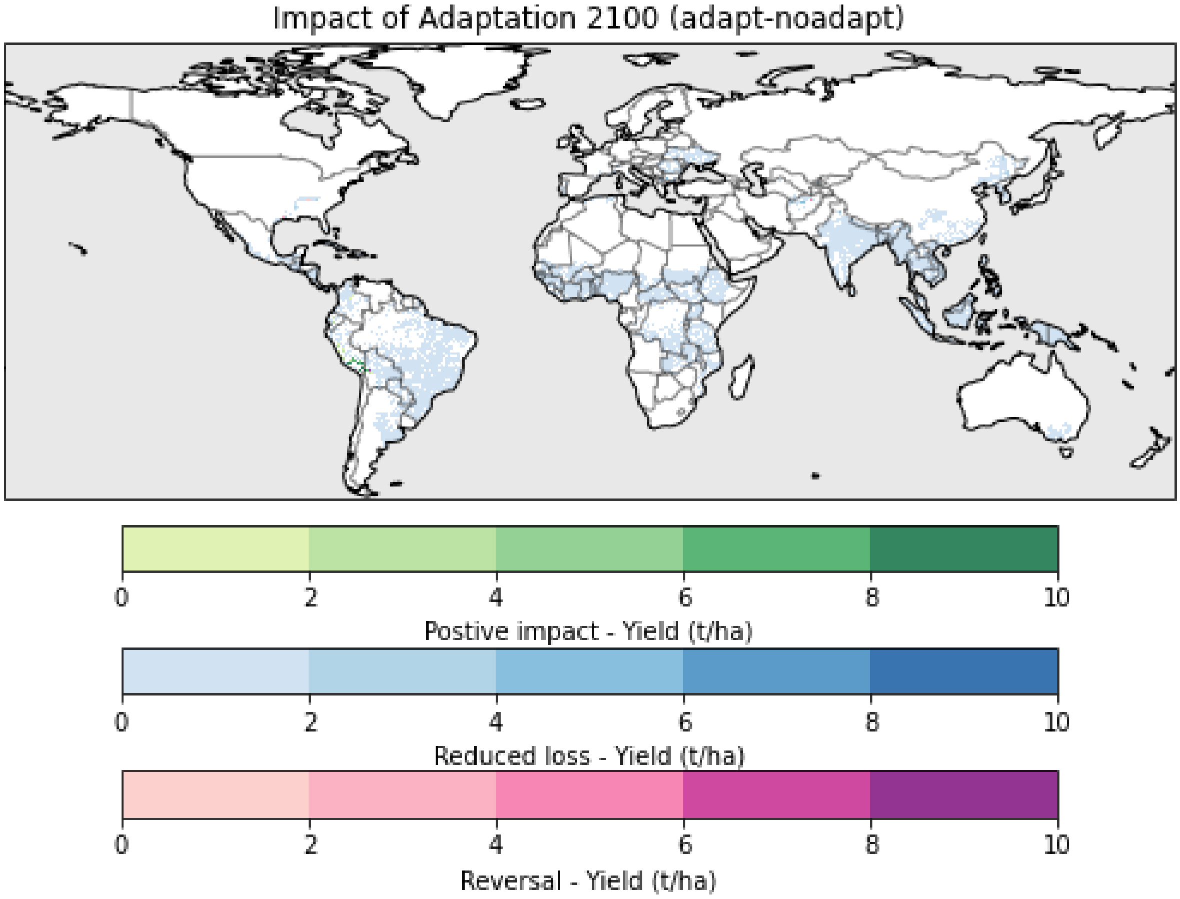

Figure S153: Global rainfed rice (season-1) productivity: Impact of adaptation on 2100 yields

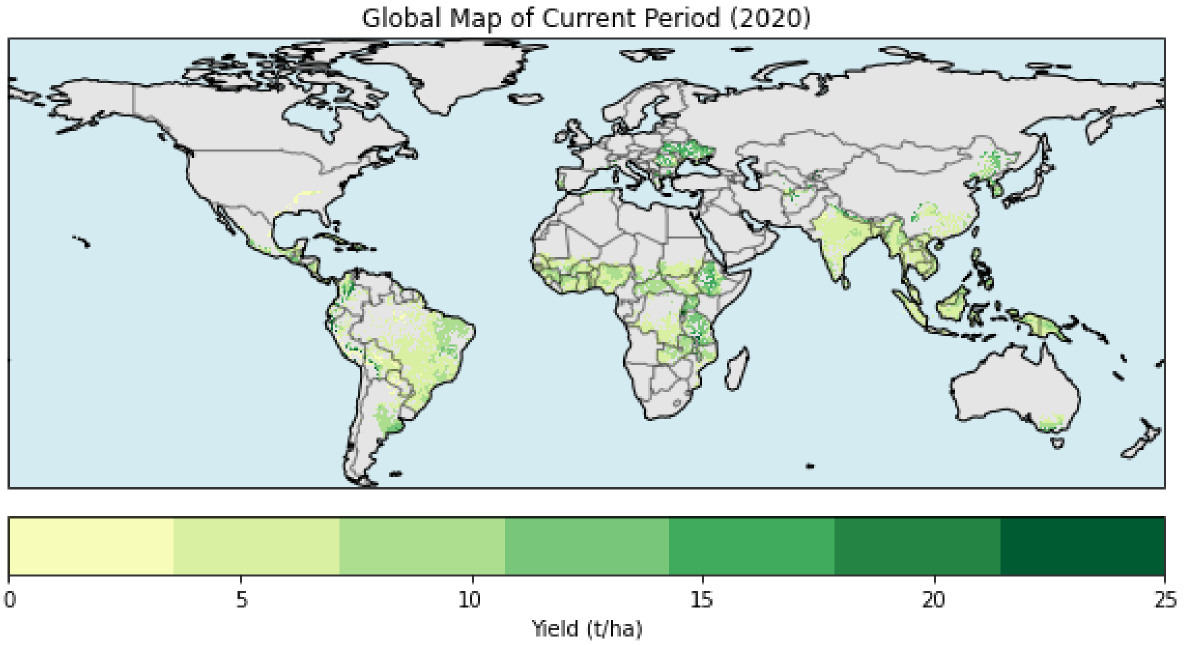

Figure S154: Global rainfed rice (season-1) reliability: Baseline yield in 2020

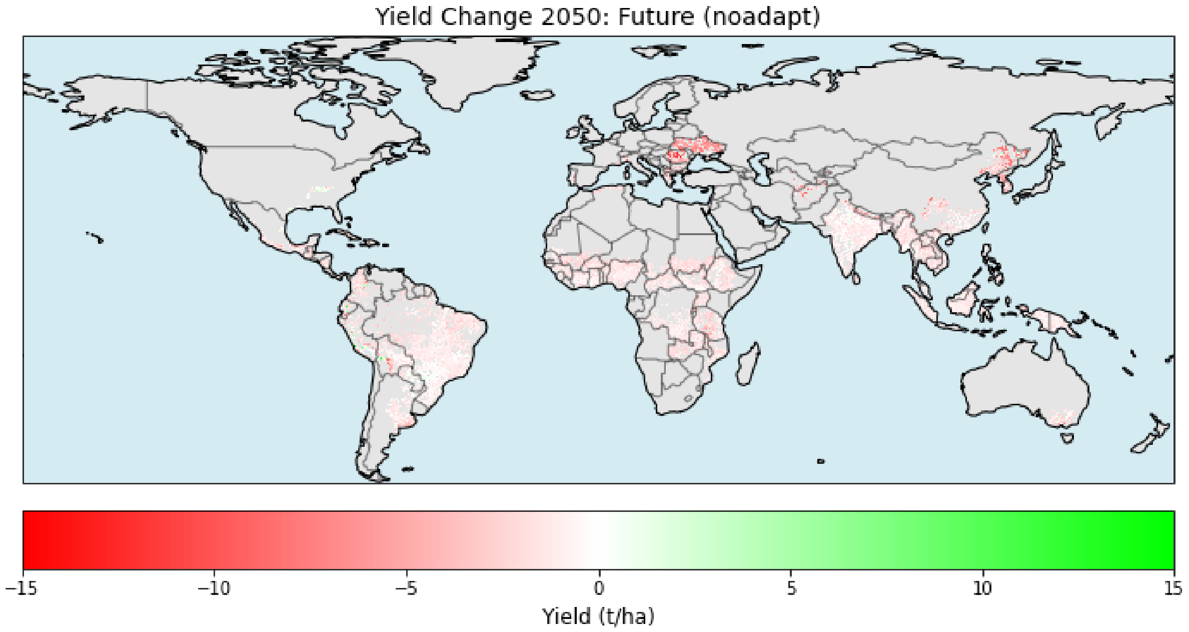

Figure S155: Global rainfed rice (season-1) reliability: Projected yield change by 2050 without adaptation

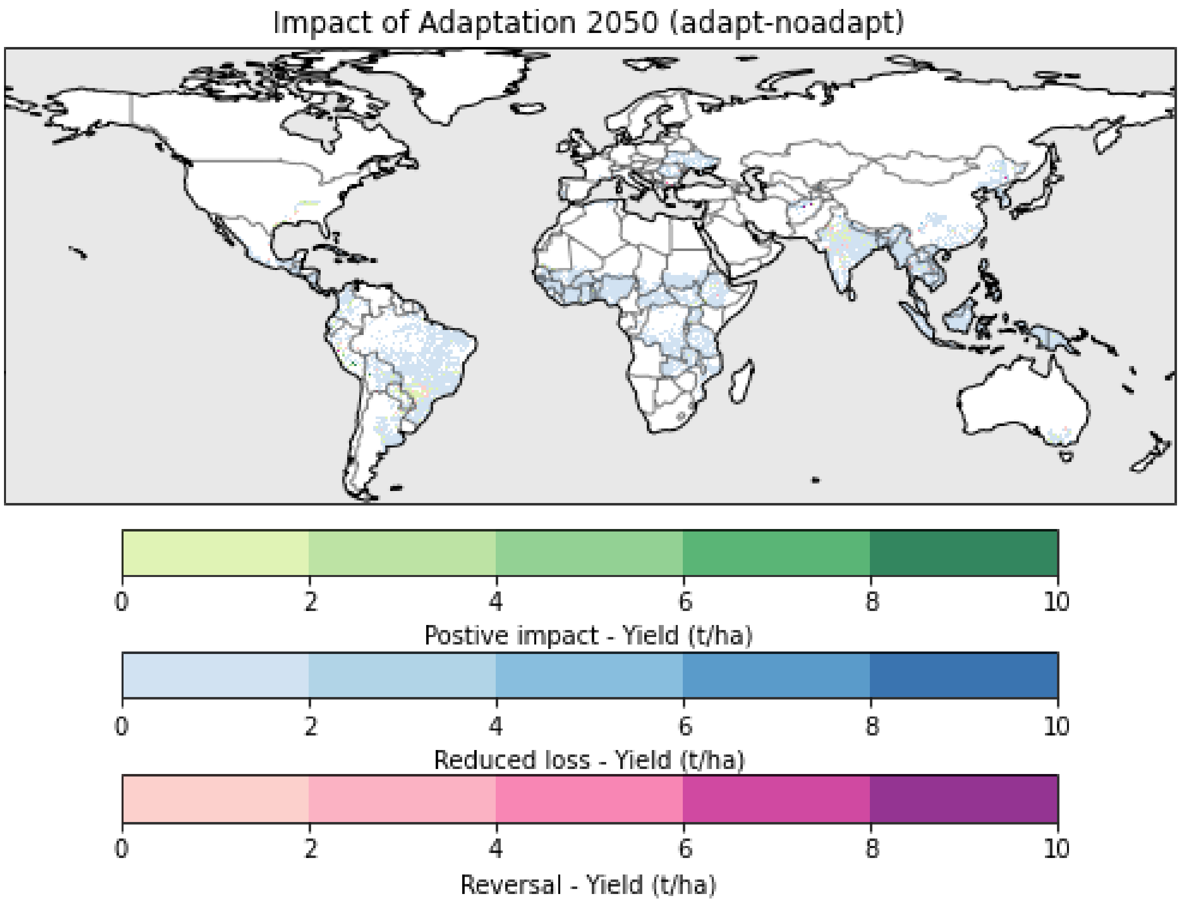

Figure S156: Global rainfed rice (season-1) reliability: Impact of adaptation on 2050 yields

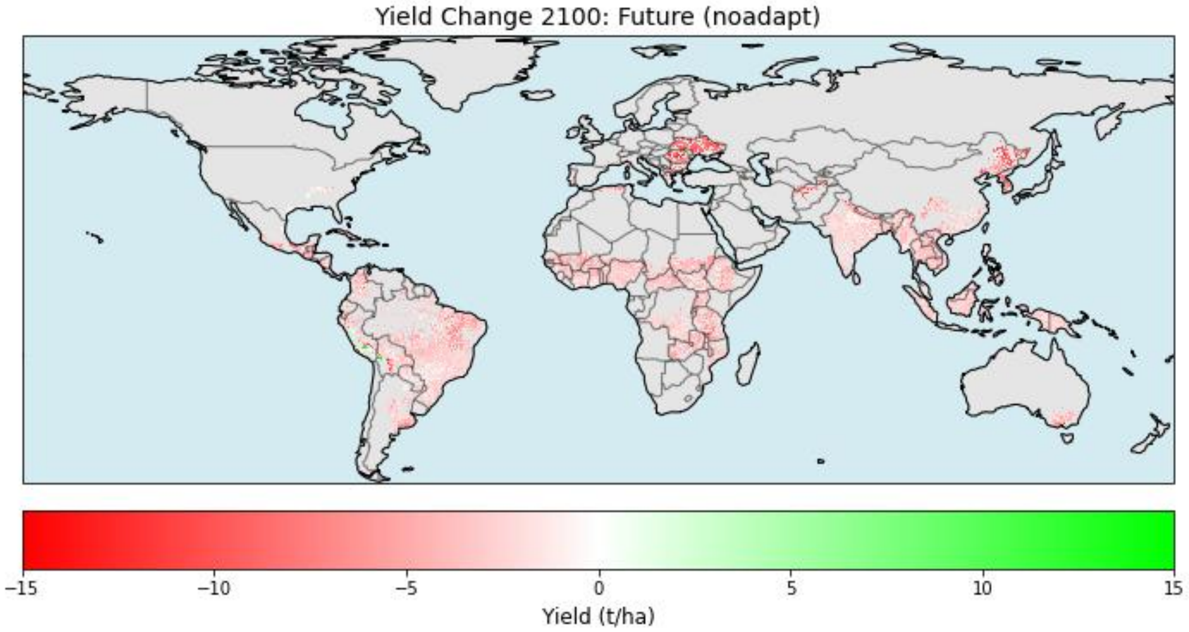

Figure S157: Global rainfed rice (season-1) reliability: Projected yield change by 2100 without adaptation

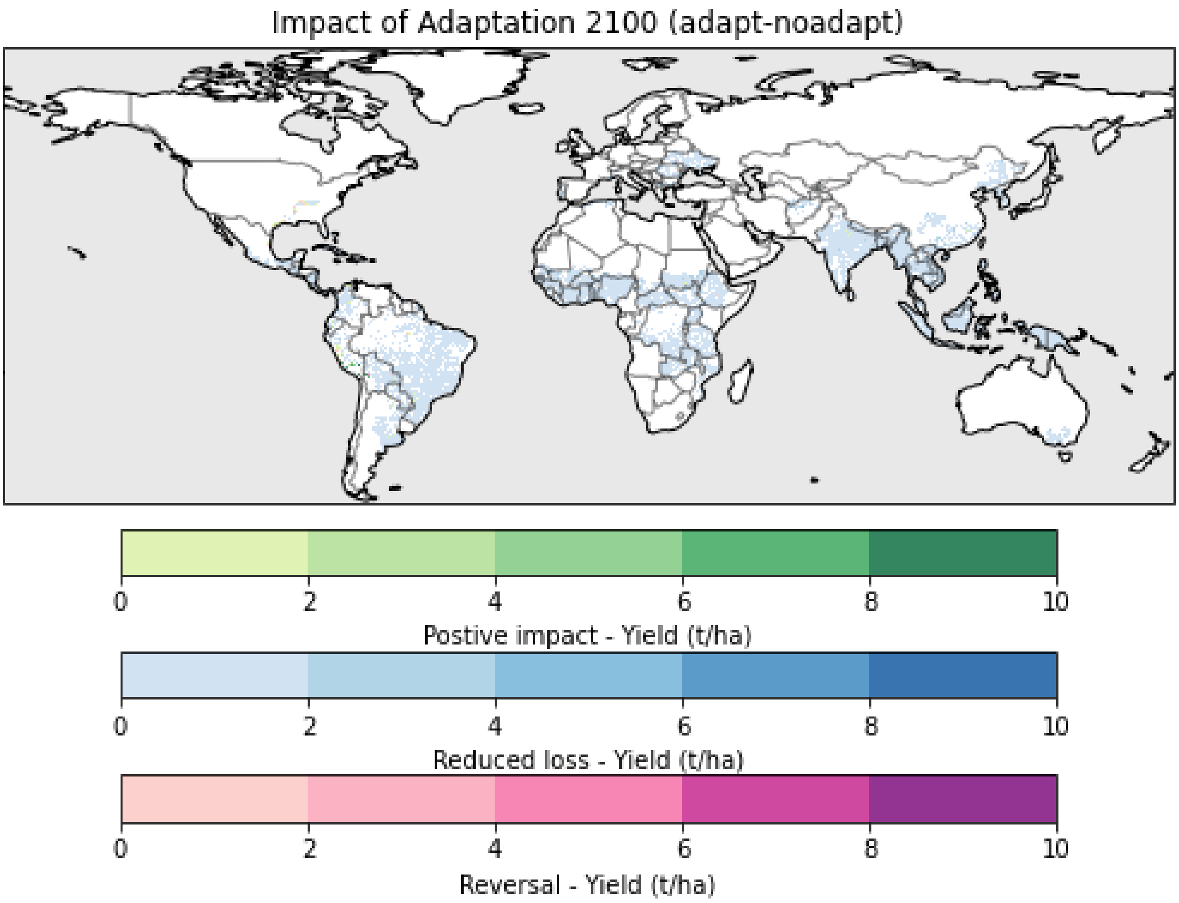

Figure S158: Global rainfed rice (season-1) reliability: Impact of adaptation on 2100 yields

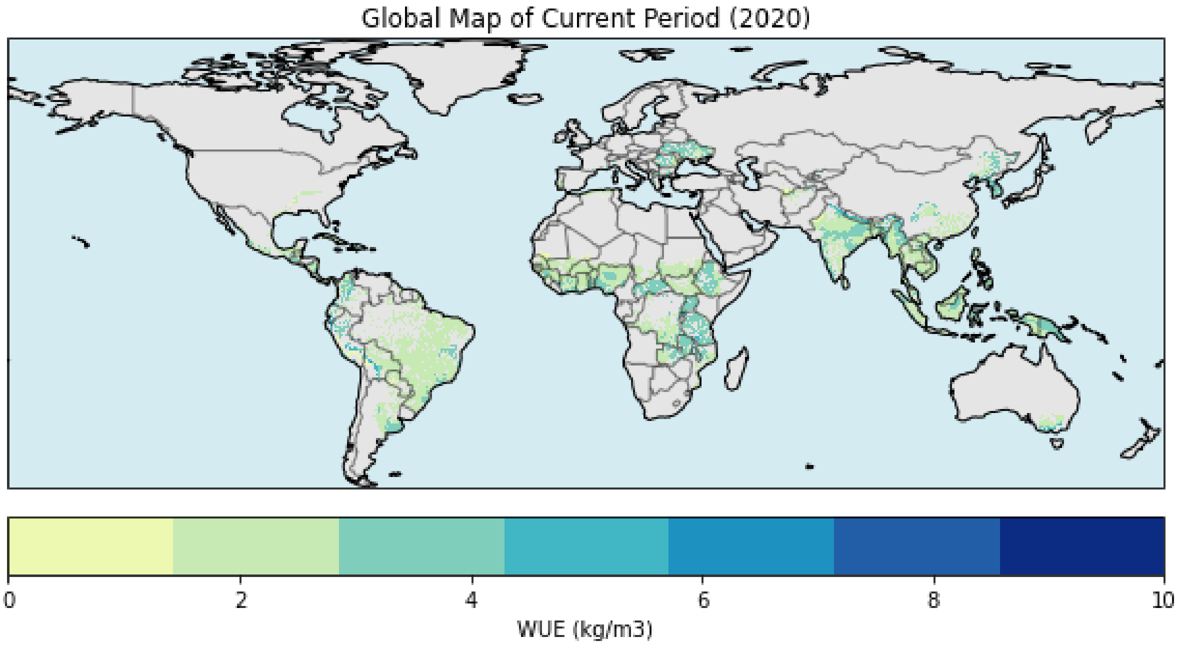

Figure S159: Global rainfed rice (season-1) water use efficiency: Baseline water use efficiency in 2020

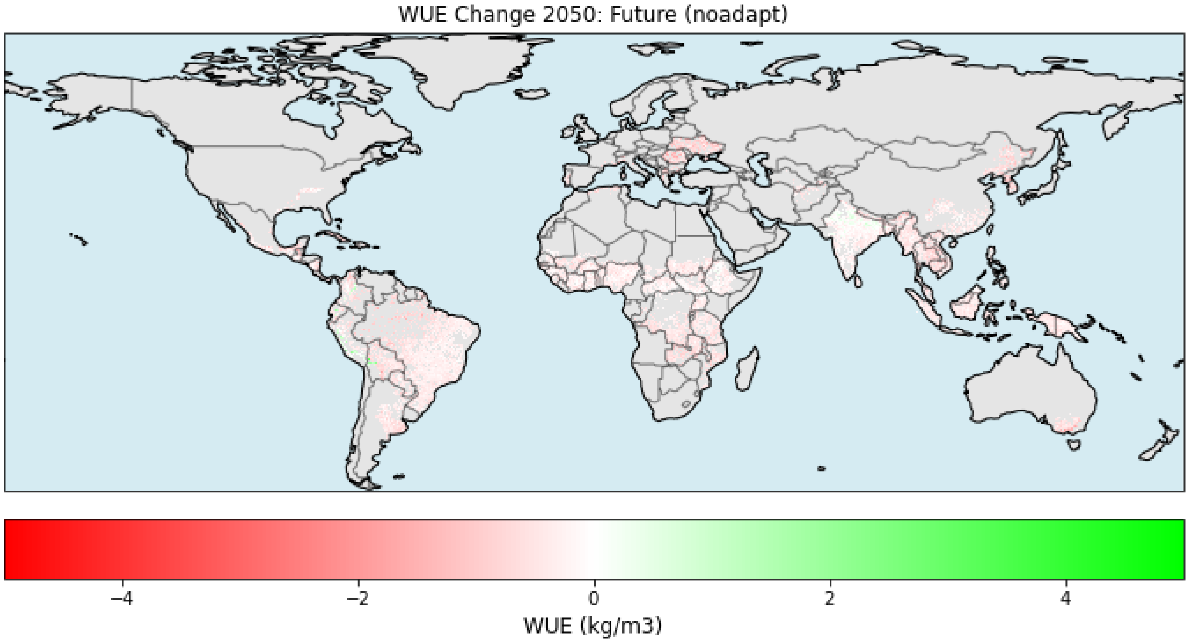

Figure S160: Global rainfed rice (season-1) water use efficiency: Projected water use efficiency change by 2050 without adaptation

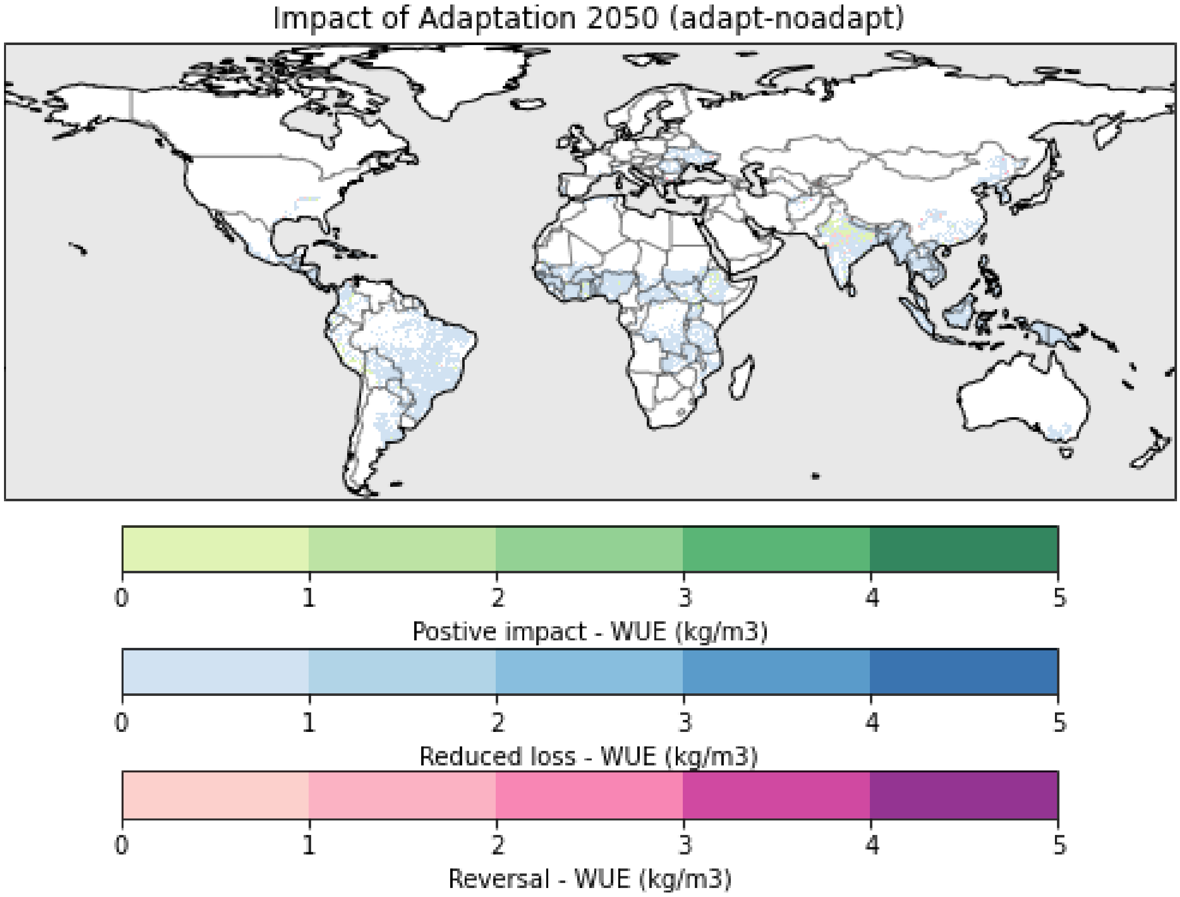

Figure S161: Global rainfed rice (season-1) water use efficiency: Impact of adaptation on 2050 water use efficiency

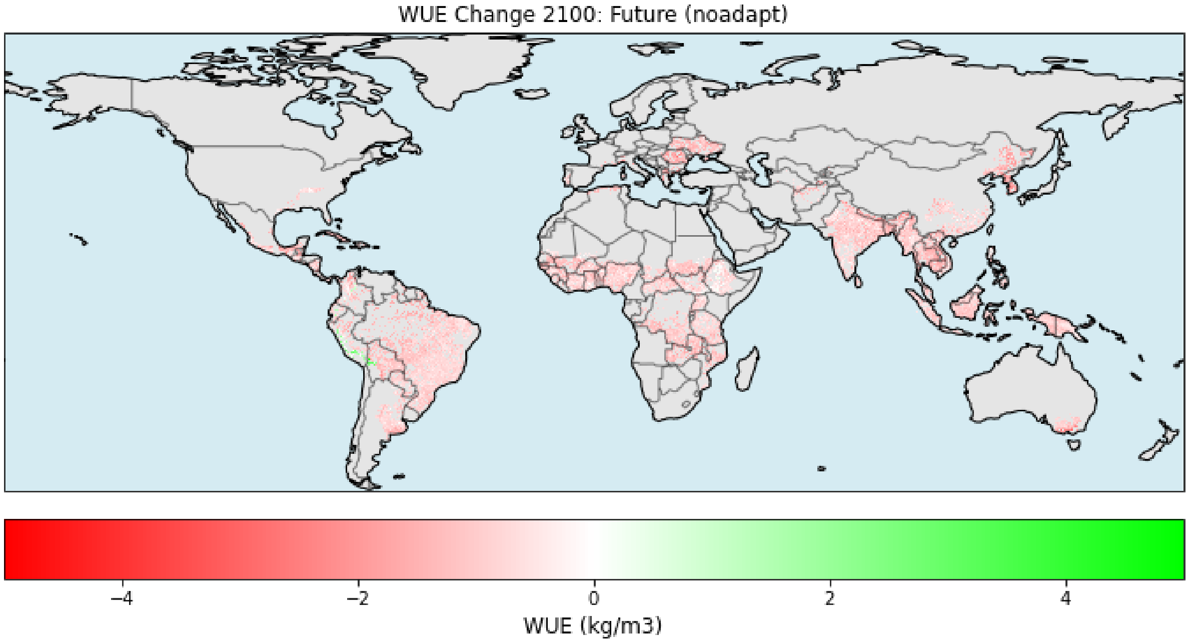

Figure S162: Global rainfed rice (season-1) water use efficiency: Projected water use efficiency change by 2100 without adaptation

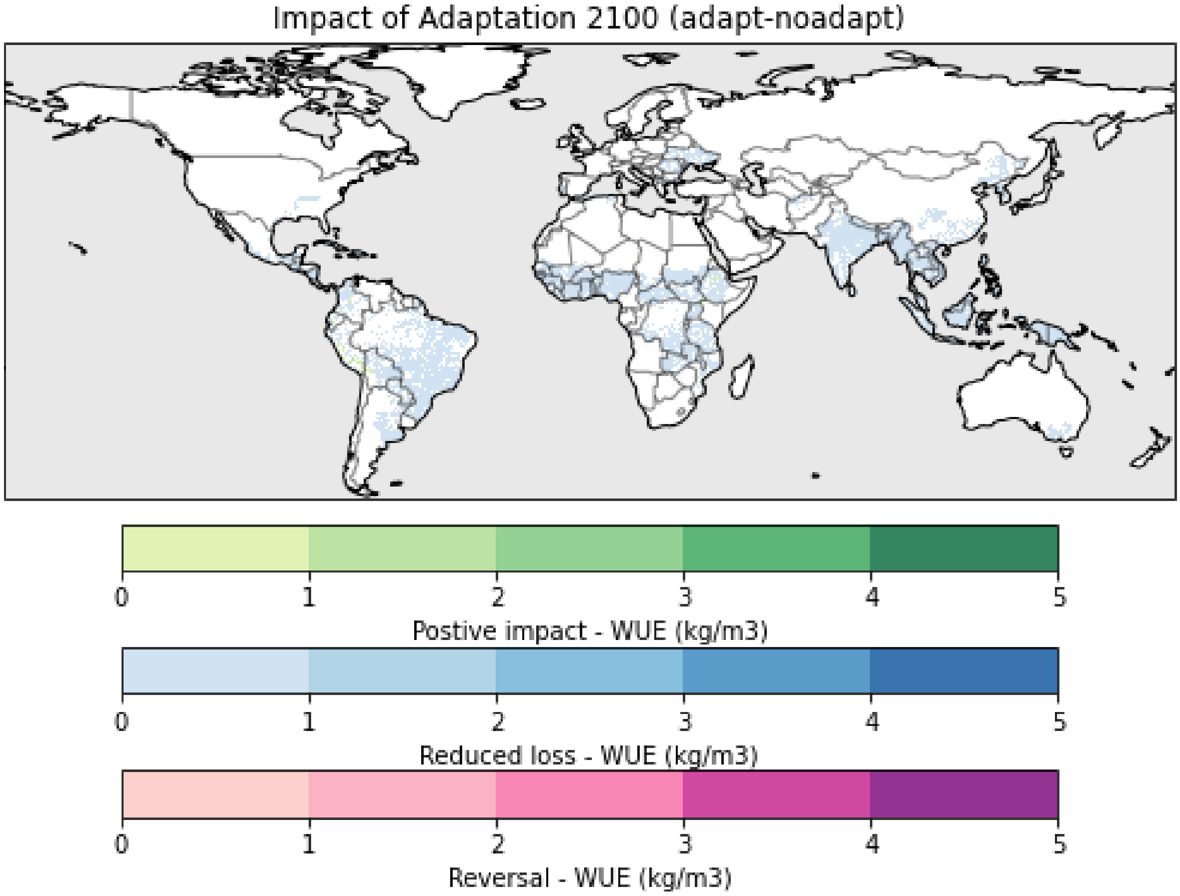

Figure S163: Global rainfed rice (season-1) water use efficiency: Impact of adaptation on 2100 water use efficiency

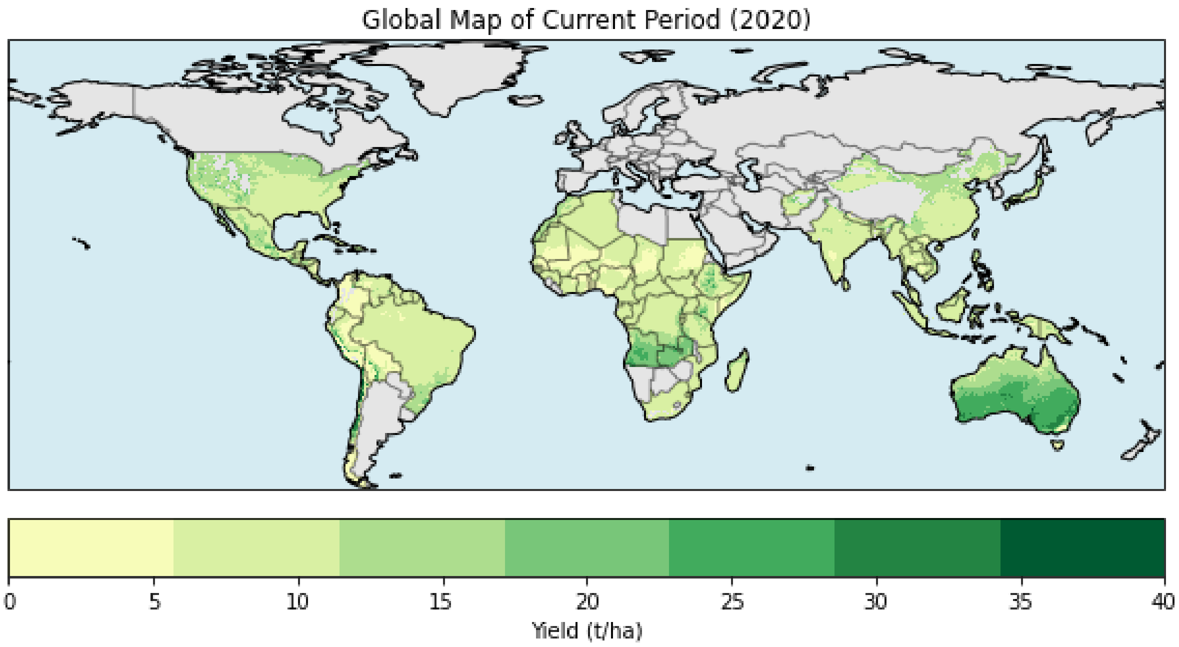

Figure S164: Global irrigated rice (season-2) productivity: Baseline yield in 2020

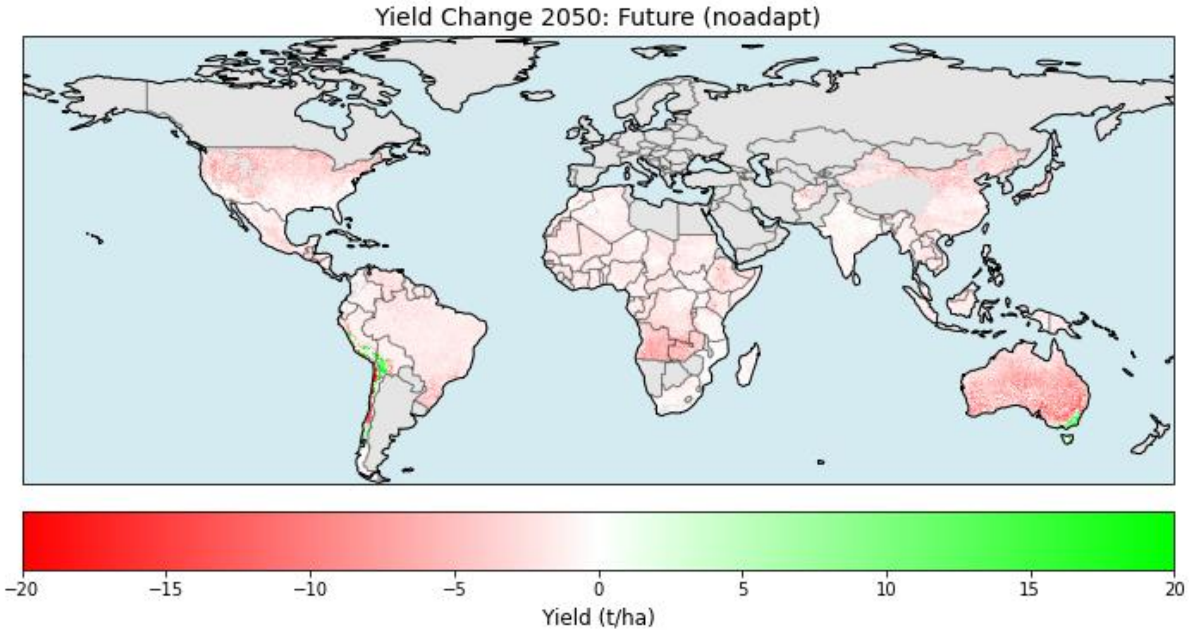

Figure S165: Global irrigated rice (season-2) productivity: Projected yield change by 2050 without adaptation

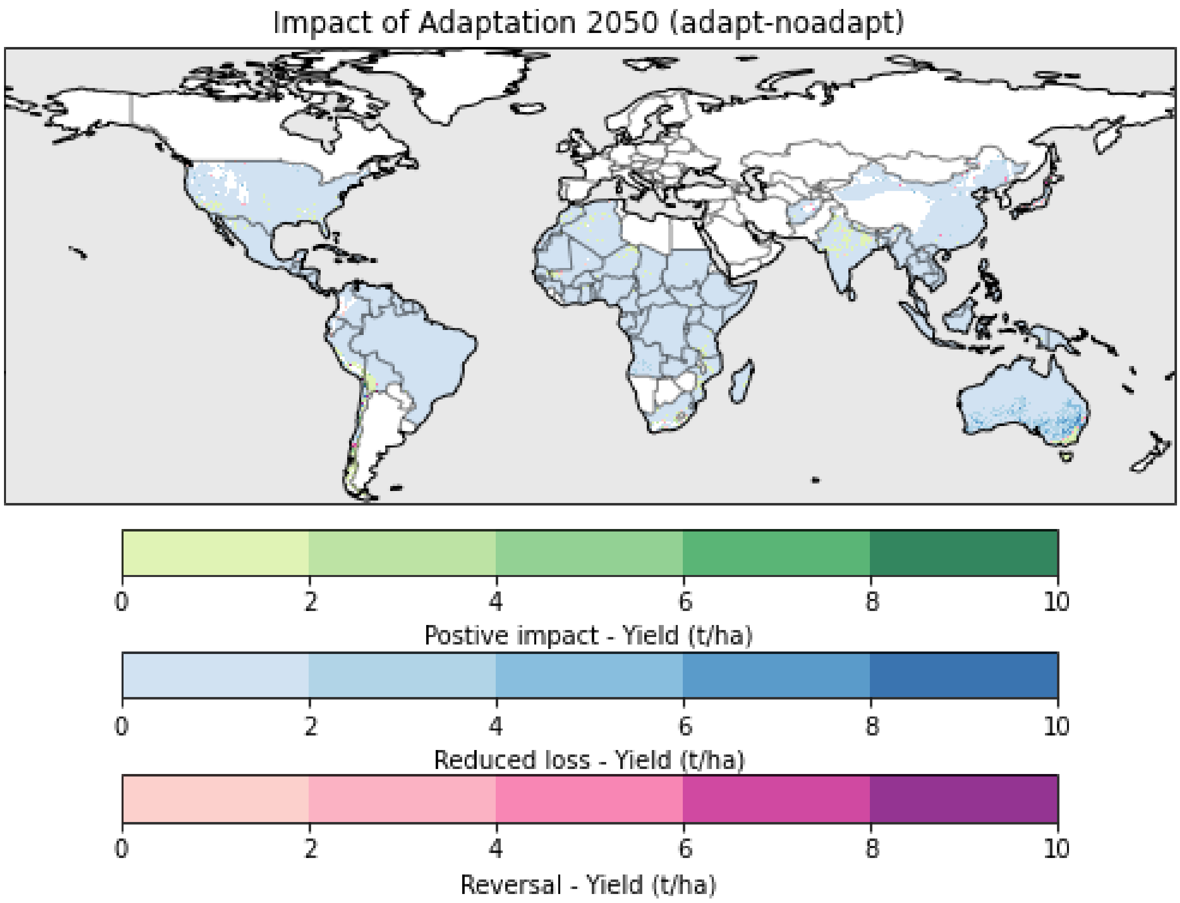

Figure S166: Global irrigated rice (season-2) productivity: Impact of adaptation on 2050 yields

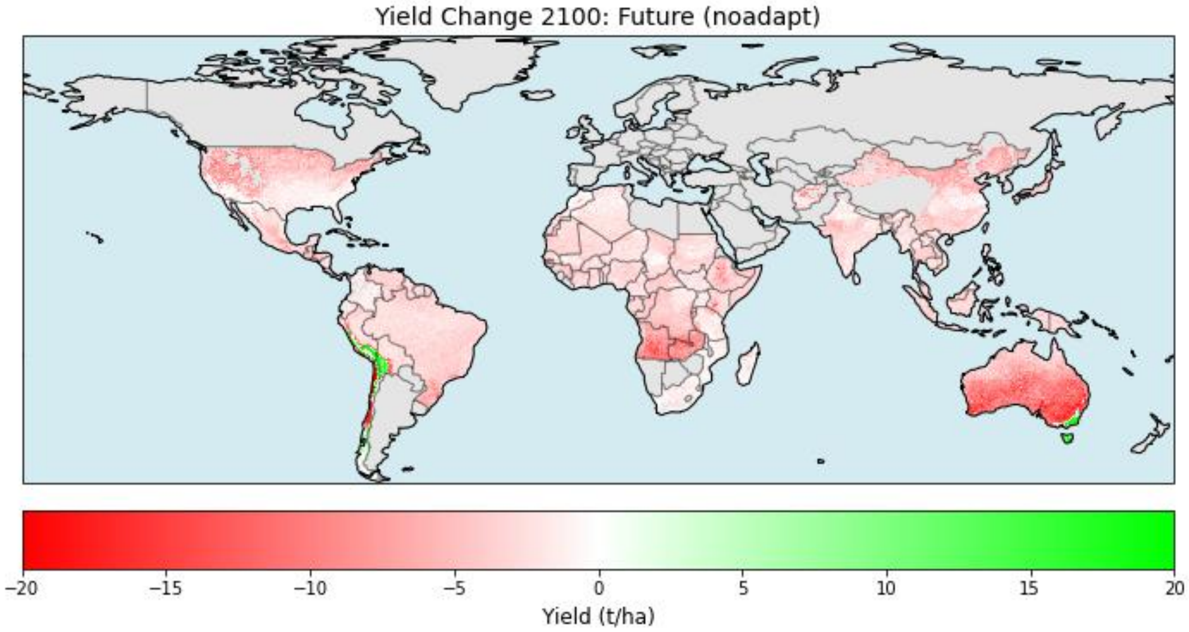

Figure S167: Global irrigated rice (season-2) productivity: Projected yield change by 2100 without adaptation

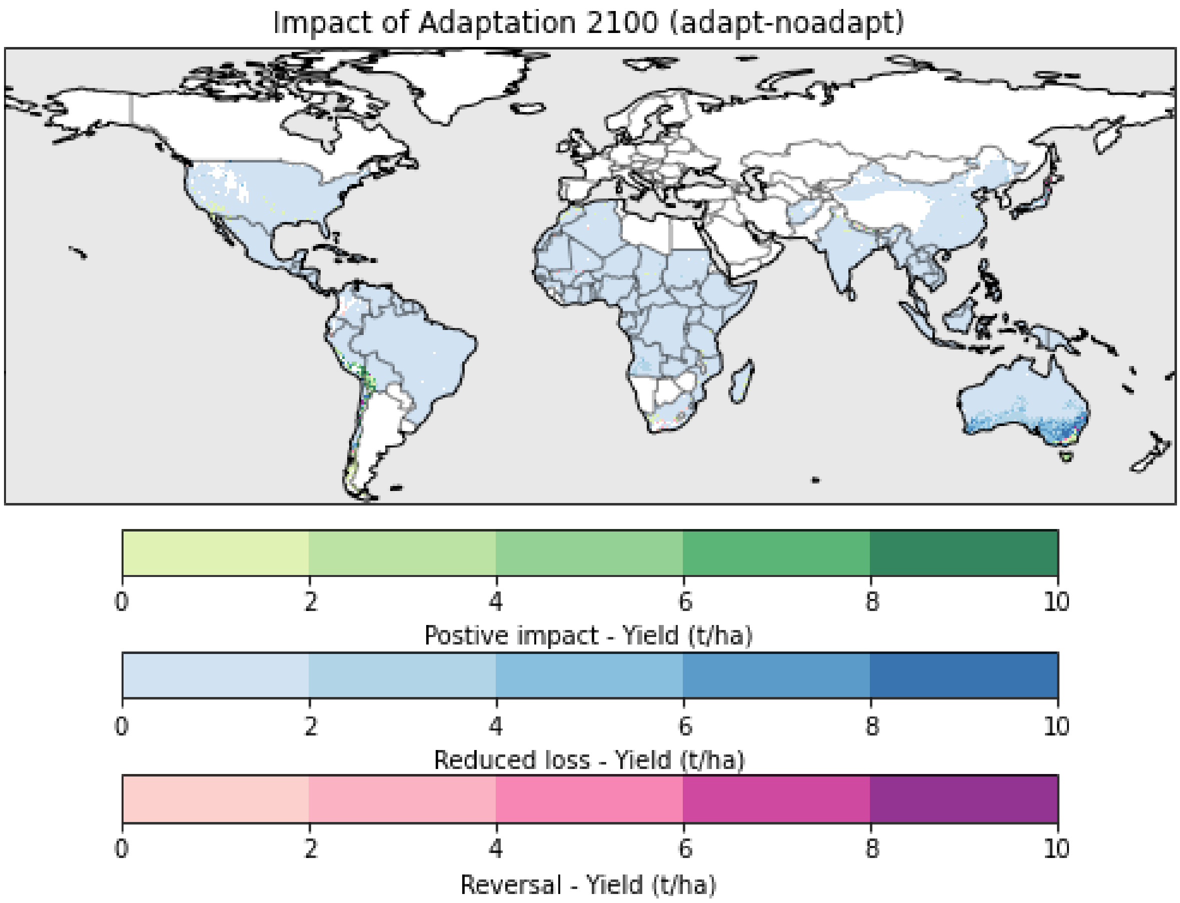

Figure S168: Global irrigated rice (season-2) productivity: Impact of adaptation on 2100 yields

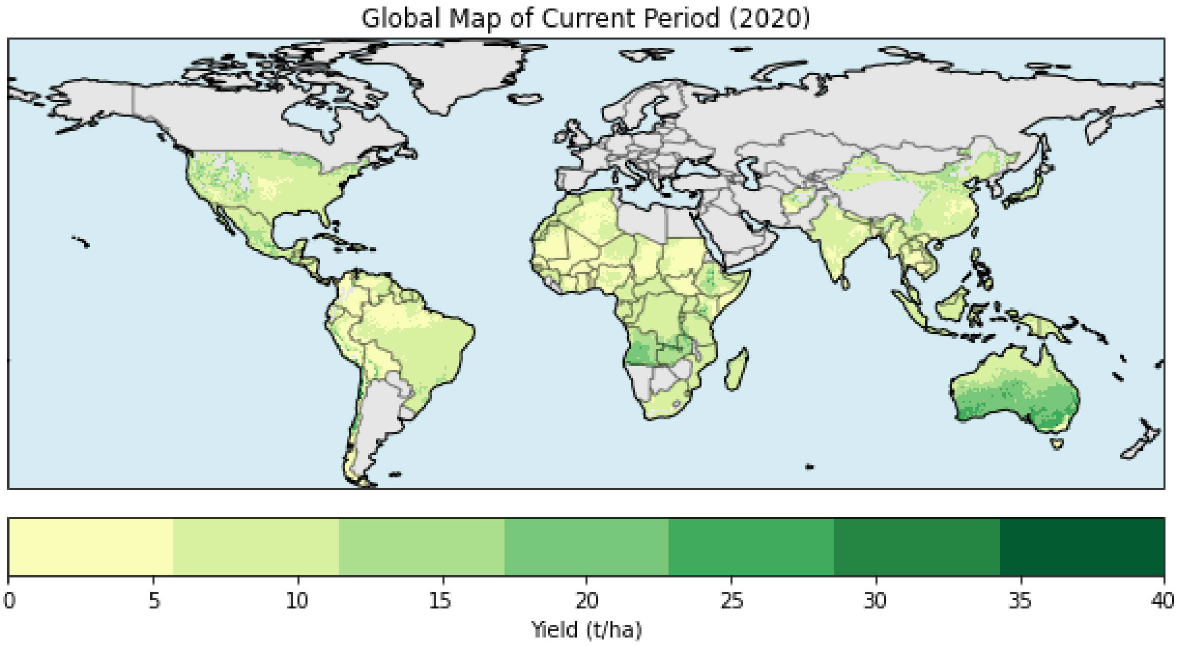

Figure S169: Global irrigated rice (season-2) reliability: Baseline yield in 2020

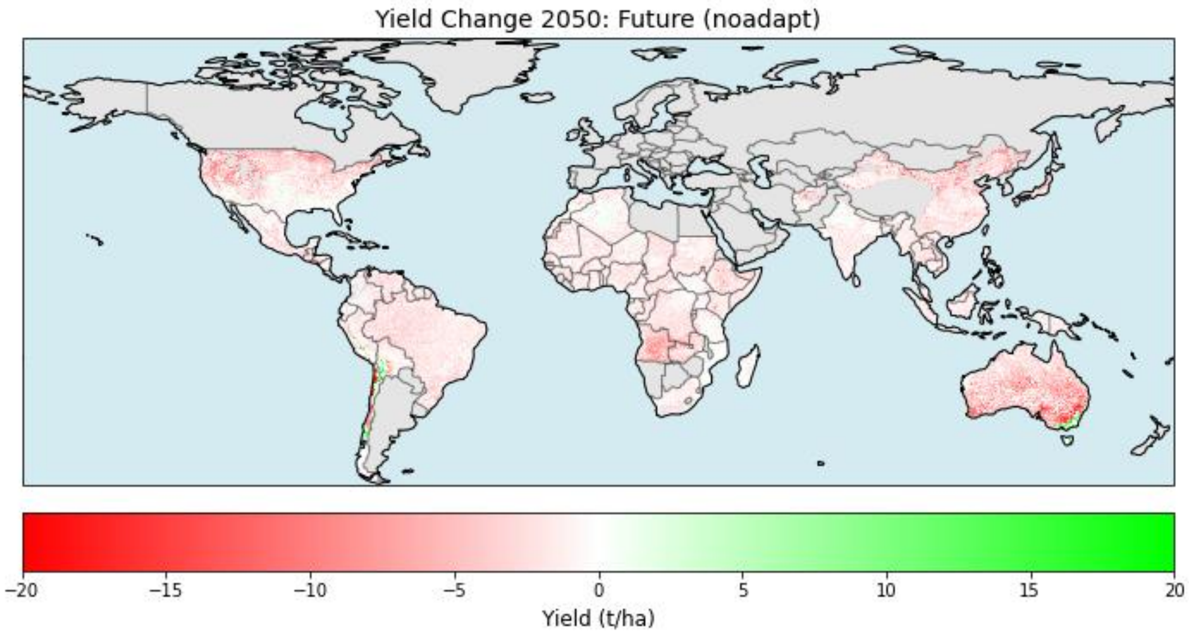

Figure S170: Global irrigated rice (season-2) reliability: Projected yield change by 2050 without adaptation

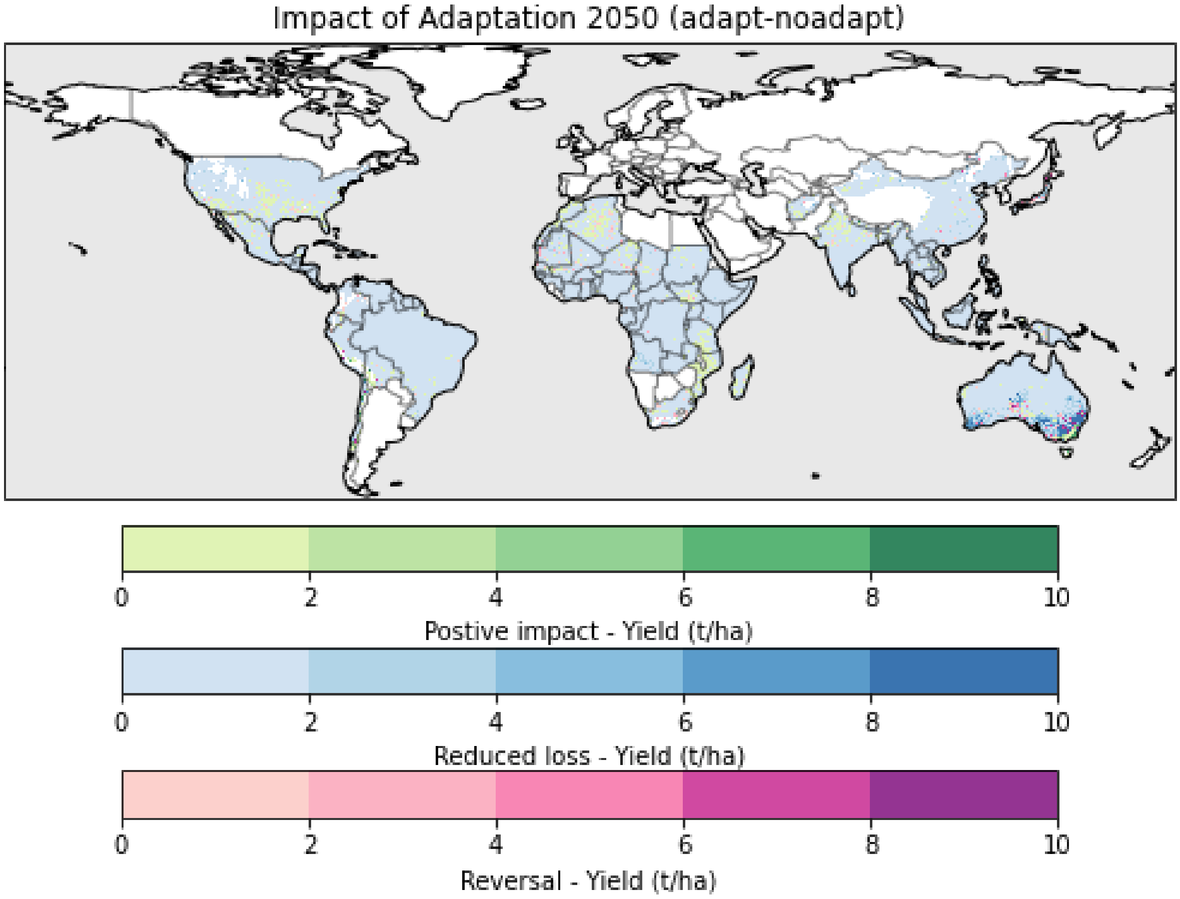

Figure S171: Global irrigated rice (season-2) reliability: Impact of adaptation on 2050 yields

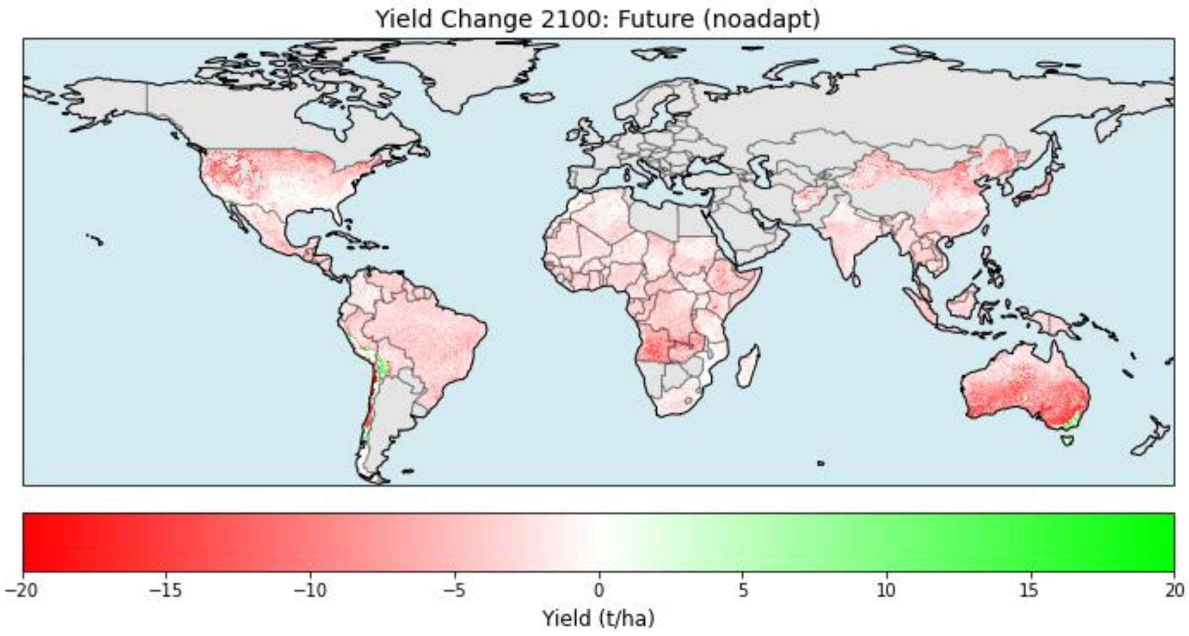

Figure S172: Global irrigated rice (season-2) reliability: Projected yield change by 2100 without adaptation

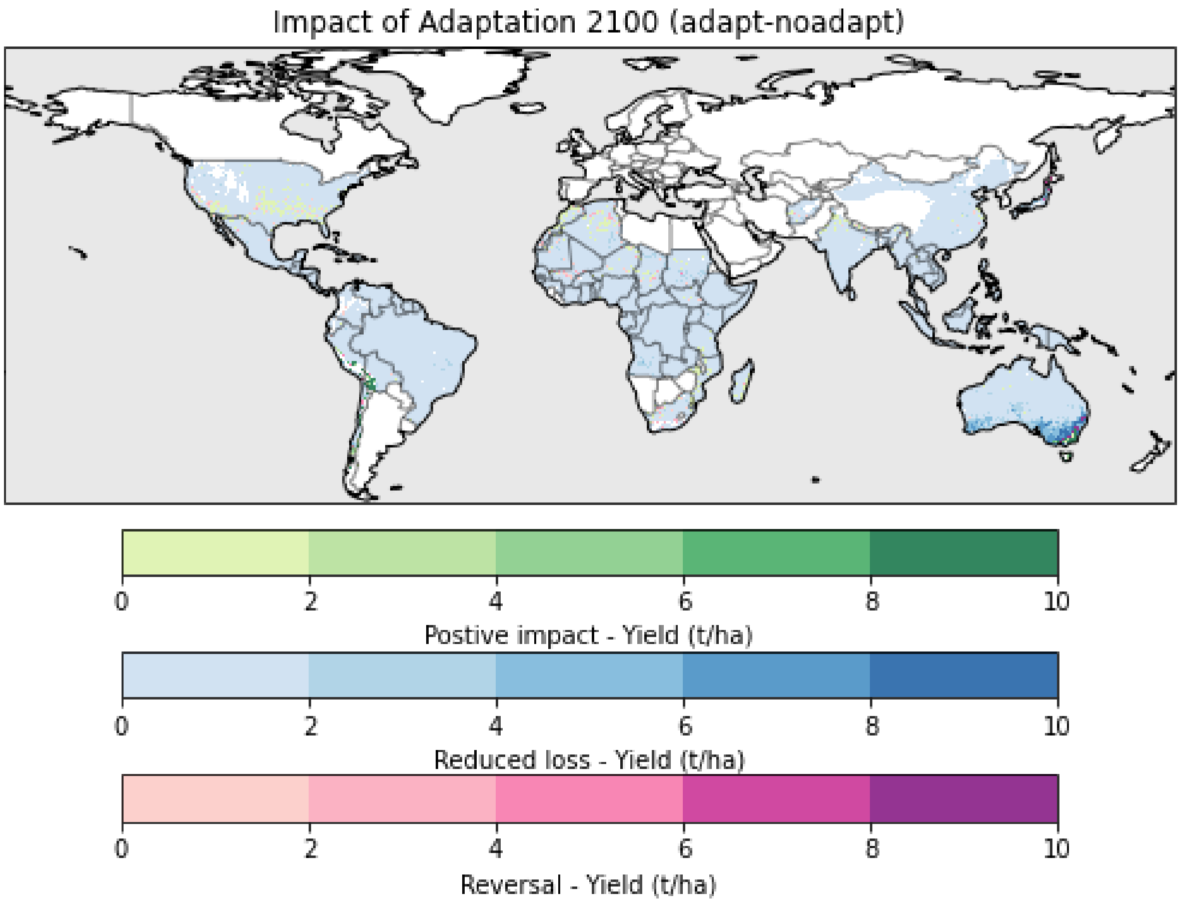

Figure S173: Global irrigated rice (season-2) reliability: Impact of adaptation on 2100 yields

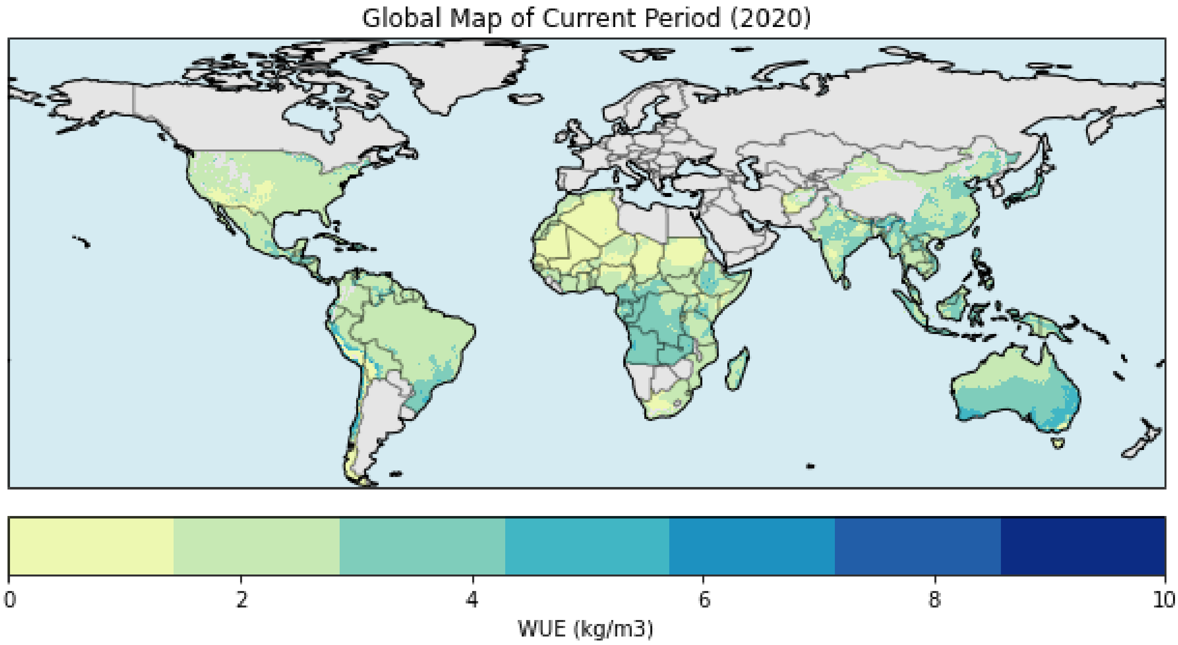

Figure S174: Global irrigated rice (season-2) water use efficiency: Baseline water use efficiency in 2020

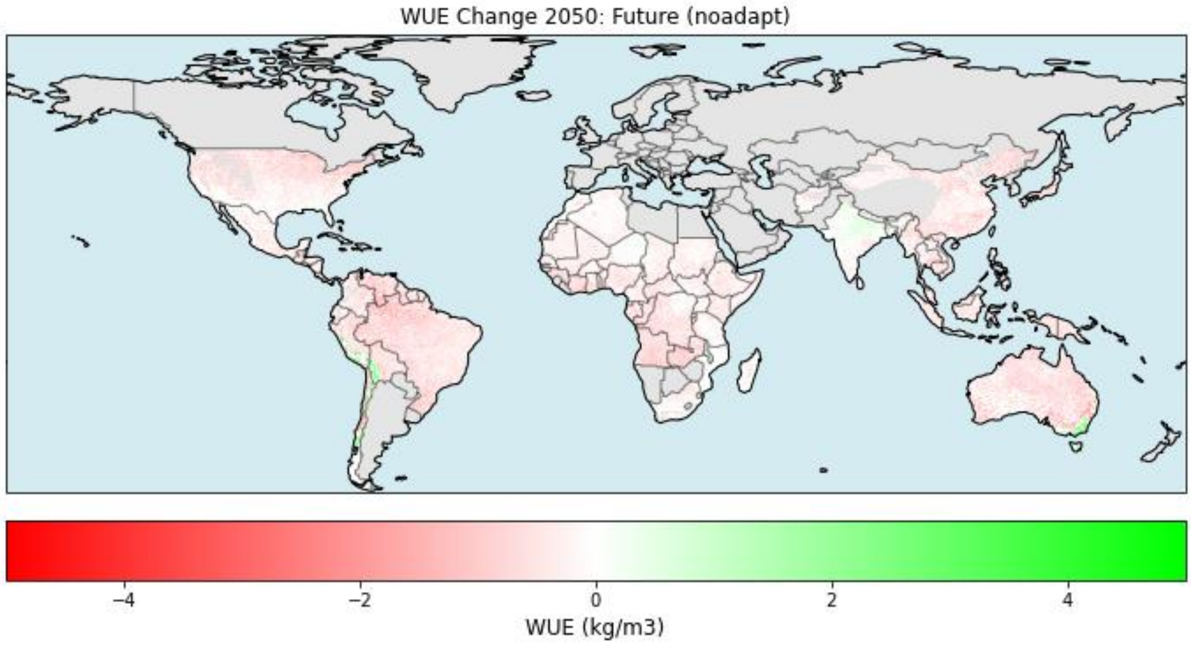

Figure S175: Global irrigated rice (season-2) water use efficiency: Projected water use efficiency change by 2050 without adaptation

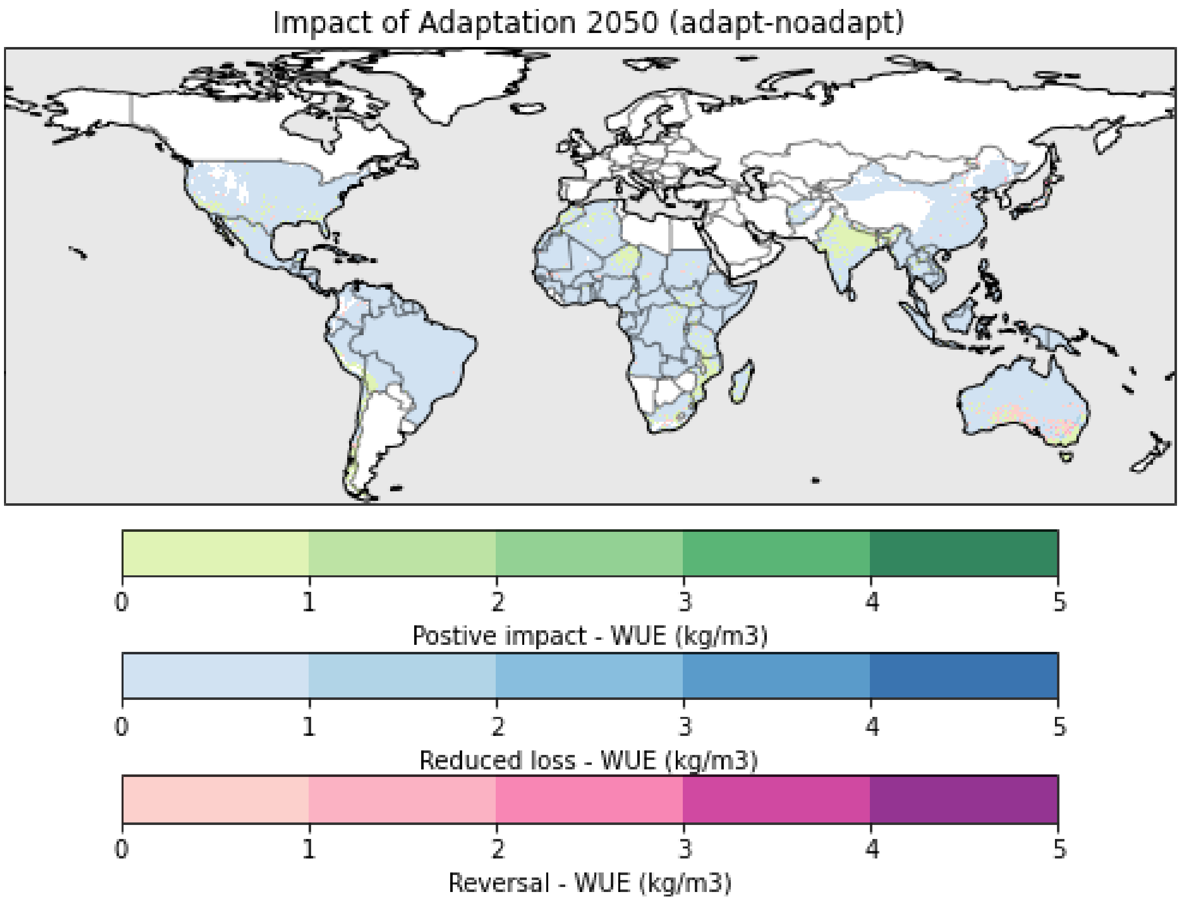

Figure S176: Global irrigated rice (season-2) water use efficiency: Impact of adaptation on 2050 water use efficiency

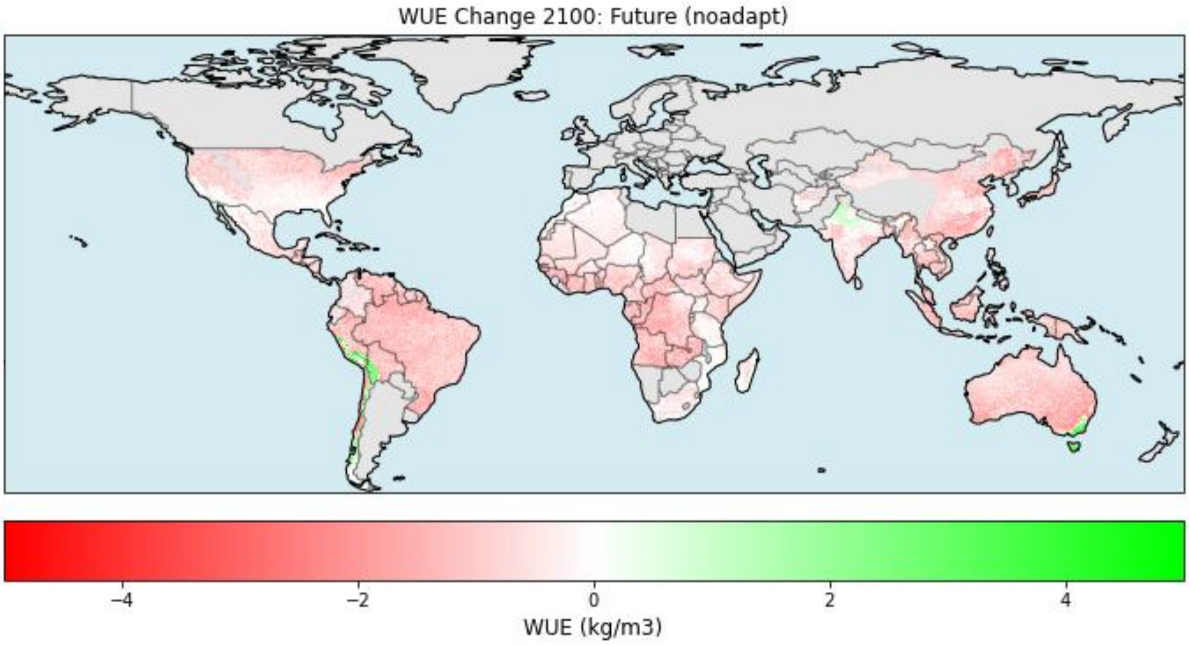

Figure S177: Global irrigated rice (season-2) water use efficiency: Projected water use efficiency change by 2100 without adaptation

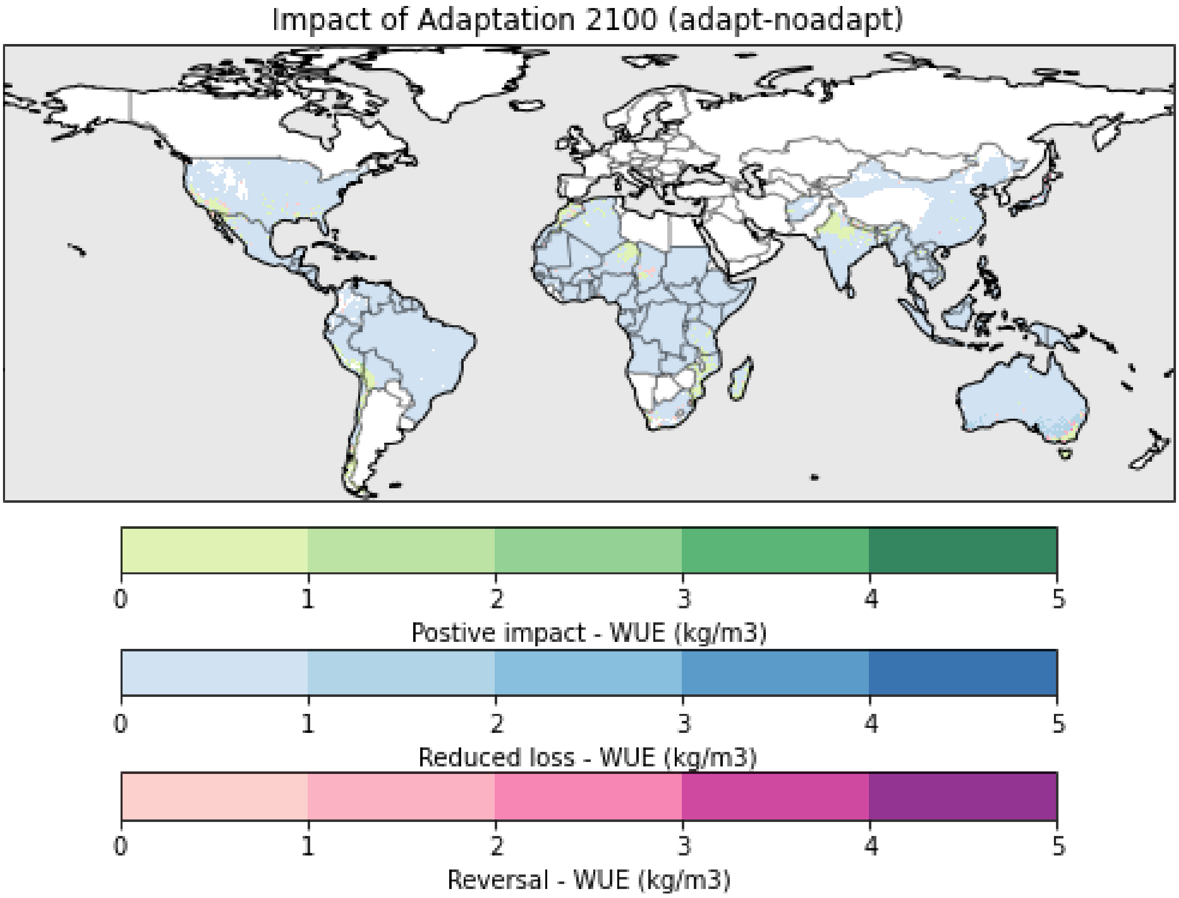

Figure S178: Global irrigated rice (season-2) water use efficiency: Impact of adaptation on 2100 water use efficiency

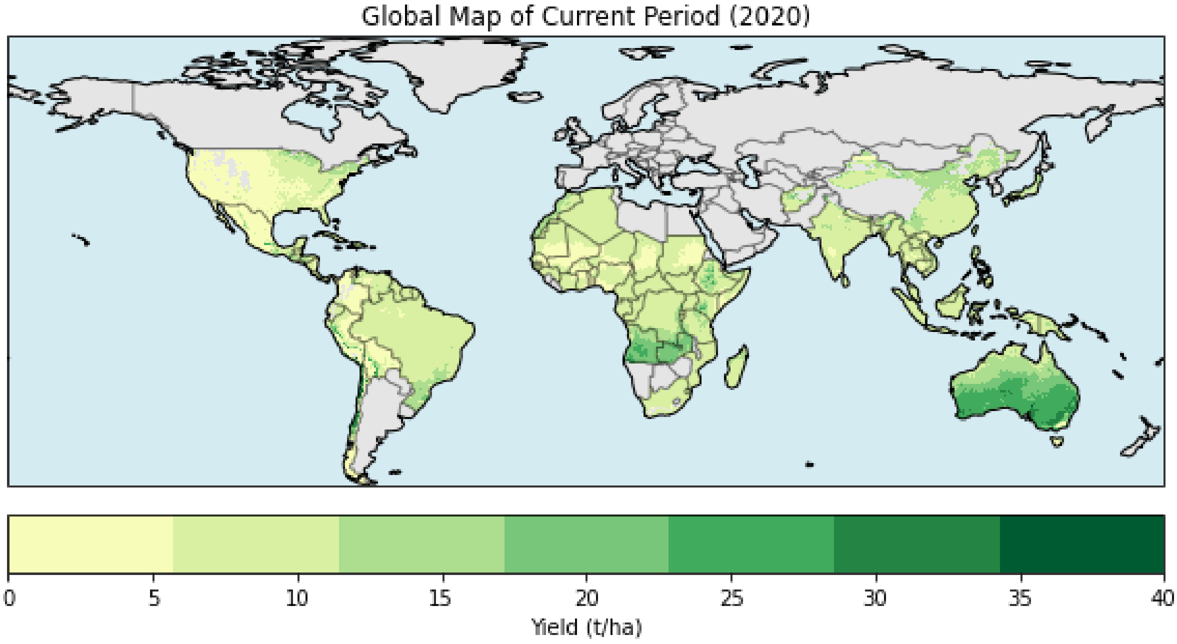

Figure S179: Global rainfed rice (season-2) productivity: Baseline yield in 2020

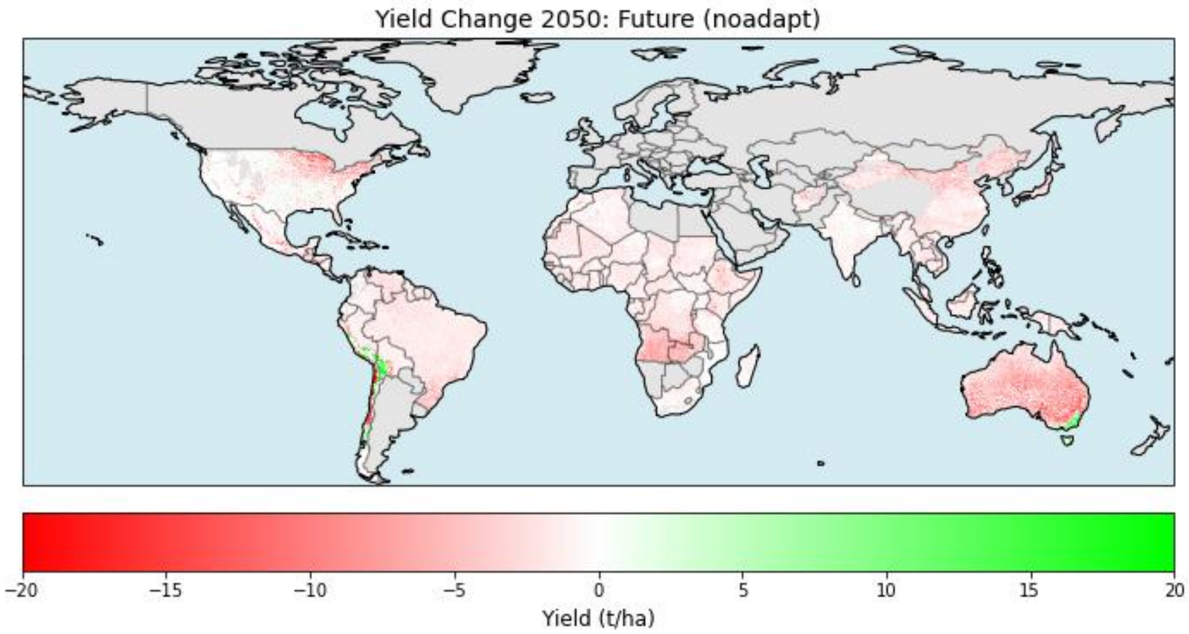

Figure S180: Global rainfed rice (season-2) productivity: Projected yield change by 2050 without adaptation

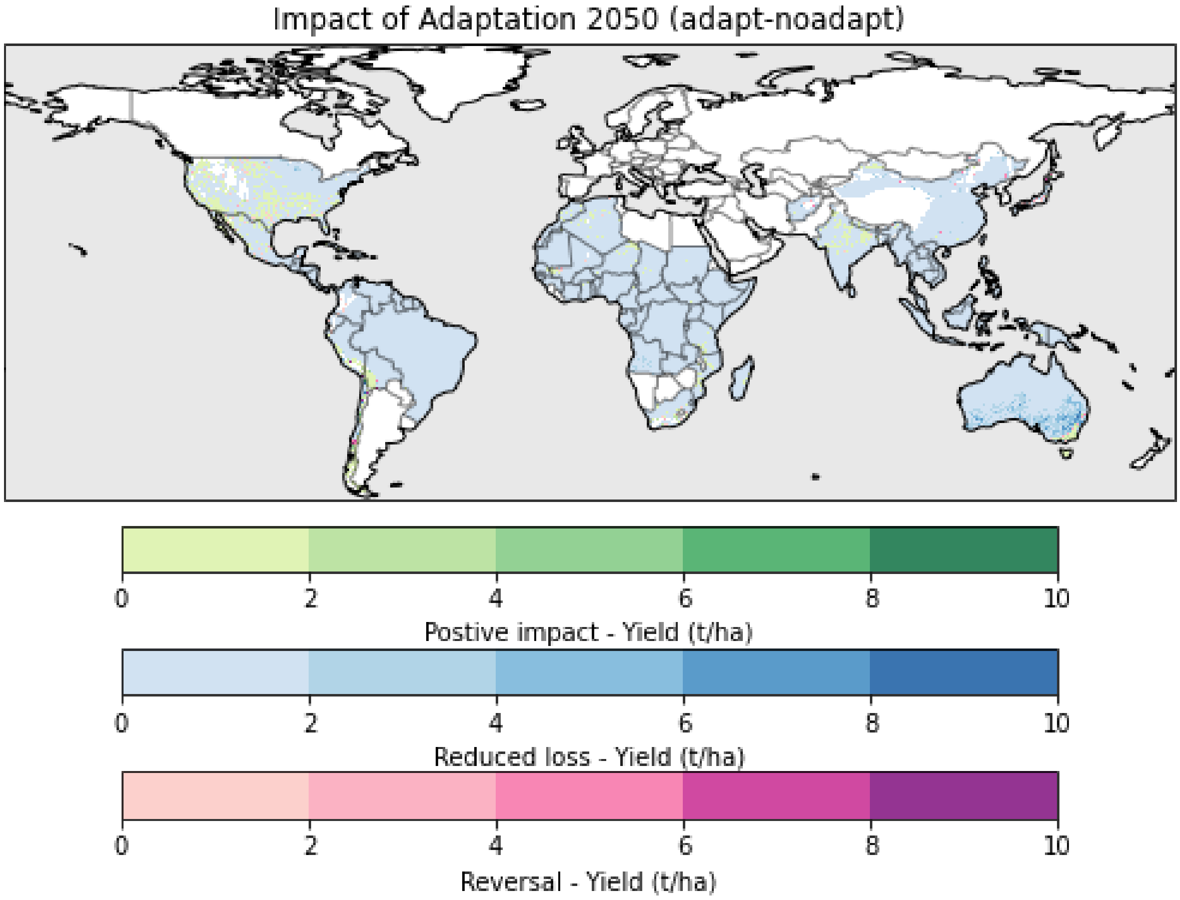

Figure S181: Global rainfed rice (season-2) productivity: Impact of adaptation on 2050 yields

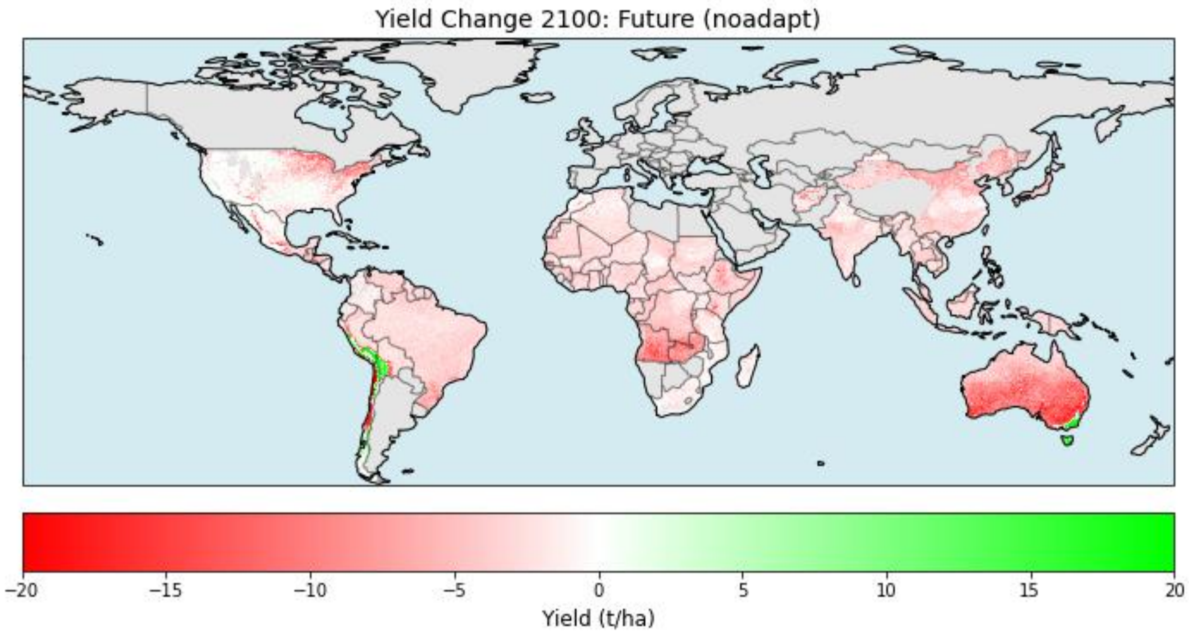

Figure S182: Global rainfed rice (season-2) productivity: Projected yield change by 2100 without adaptation

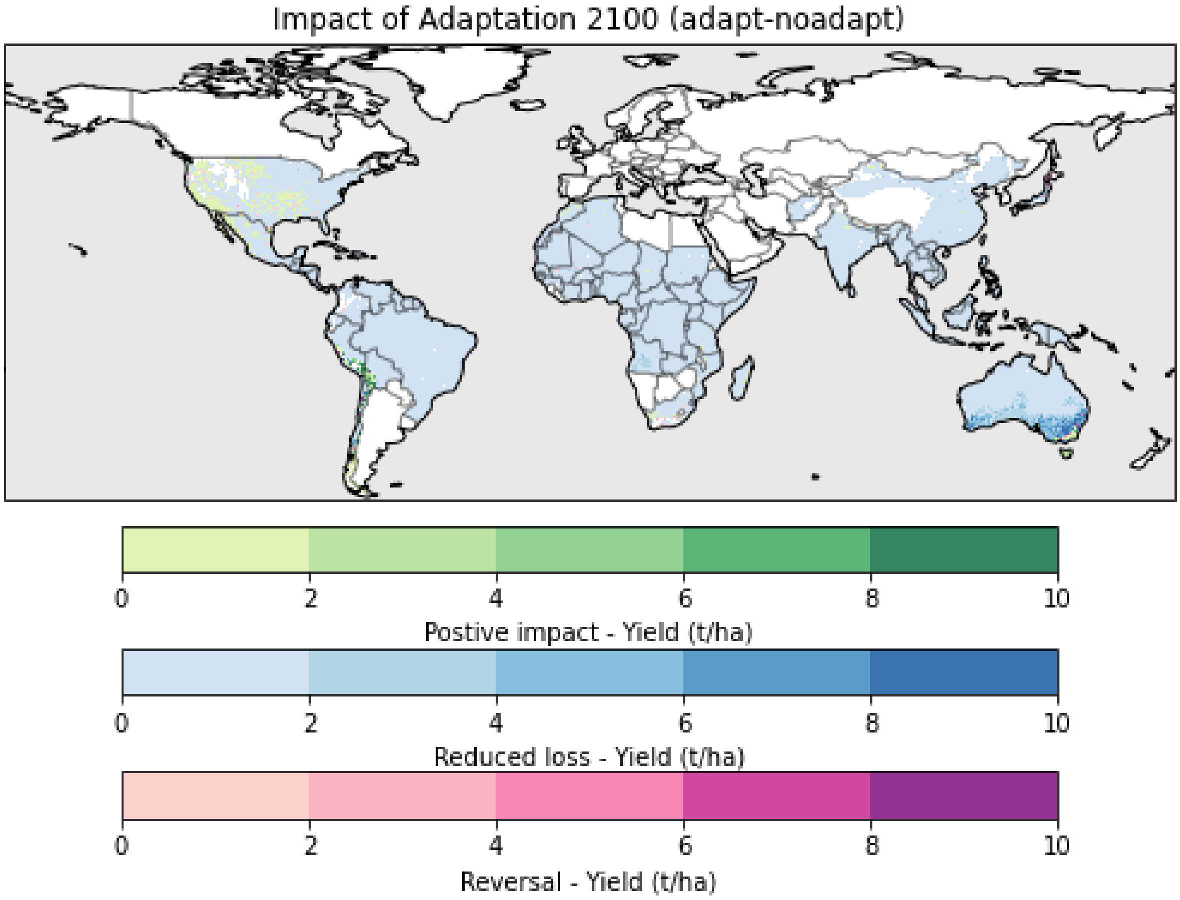

Figure S183: Global rainfed rice (season-2) productivity: Impact of adaptation on 2100 yields

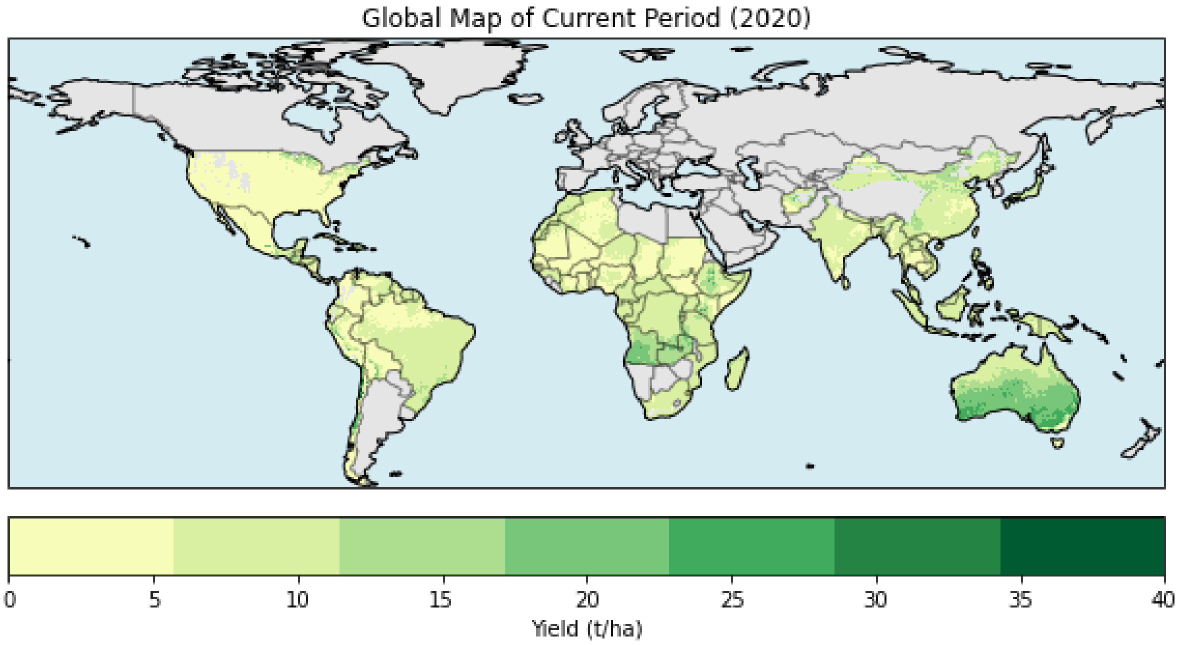

Figure S184: Global rainfed rice (season-2) reliability: Baseline yield in 2020

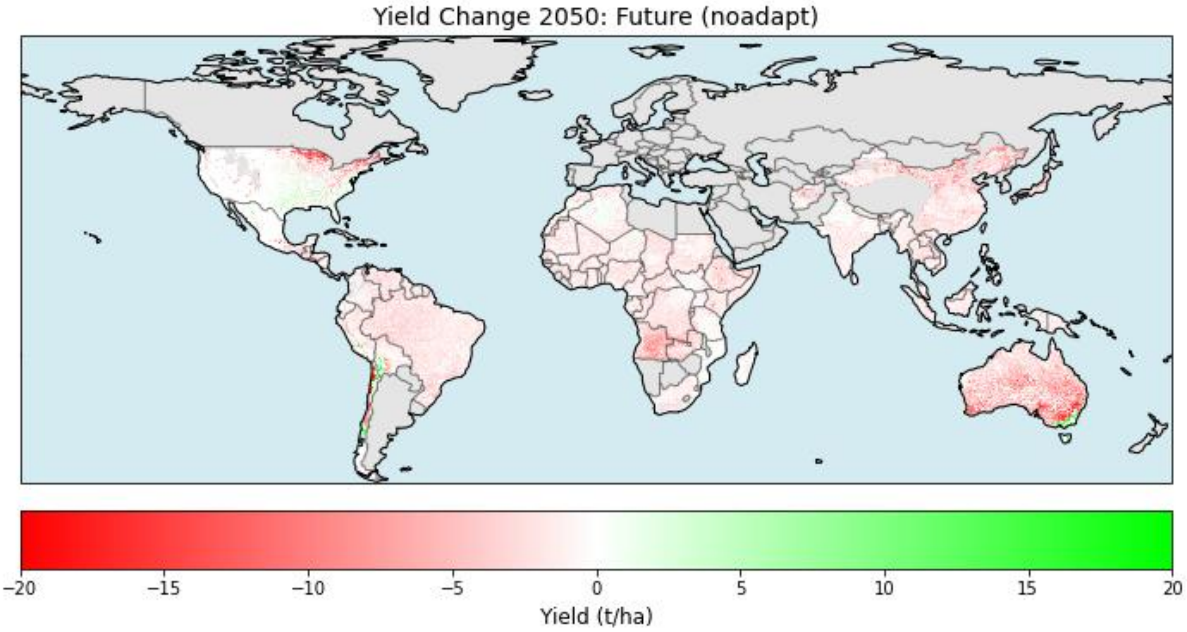

Figure S185: Global rainfed rice (season-2) reliability: Projected yield change by 2050 without adaptation

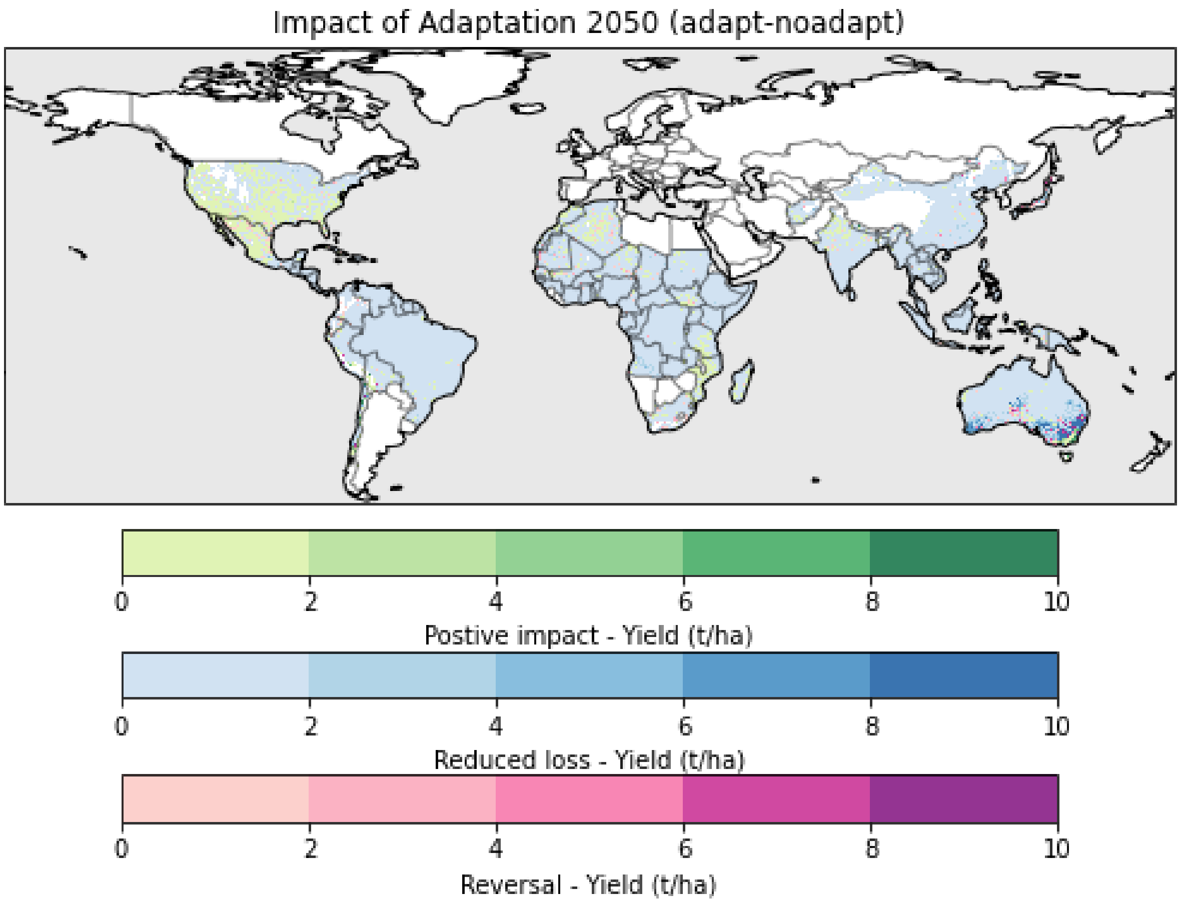

Figure S186: Global rainfed rice (season-2) reliability: Impact of adaptation on 2050 yields

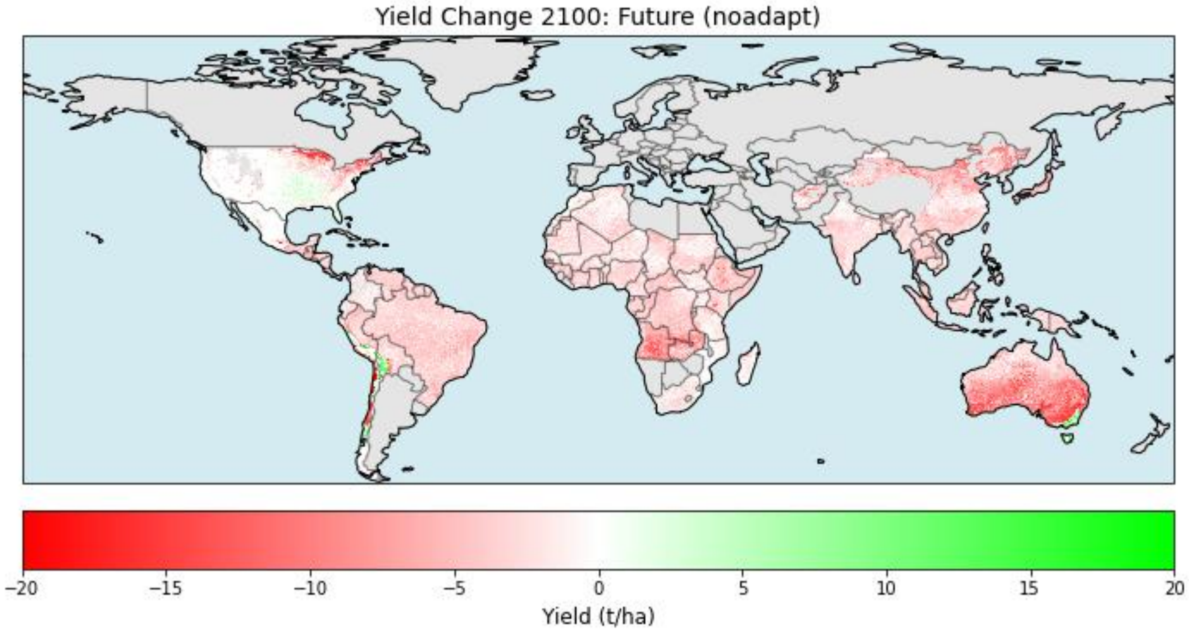

Figure S187: Global rainfed rice (season-2) reliability: Projected yield change by 2100 without adaptation

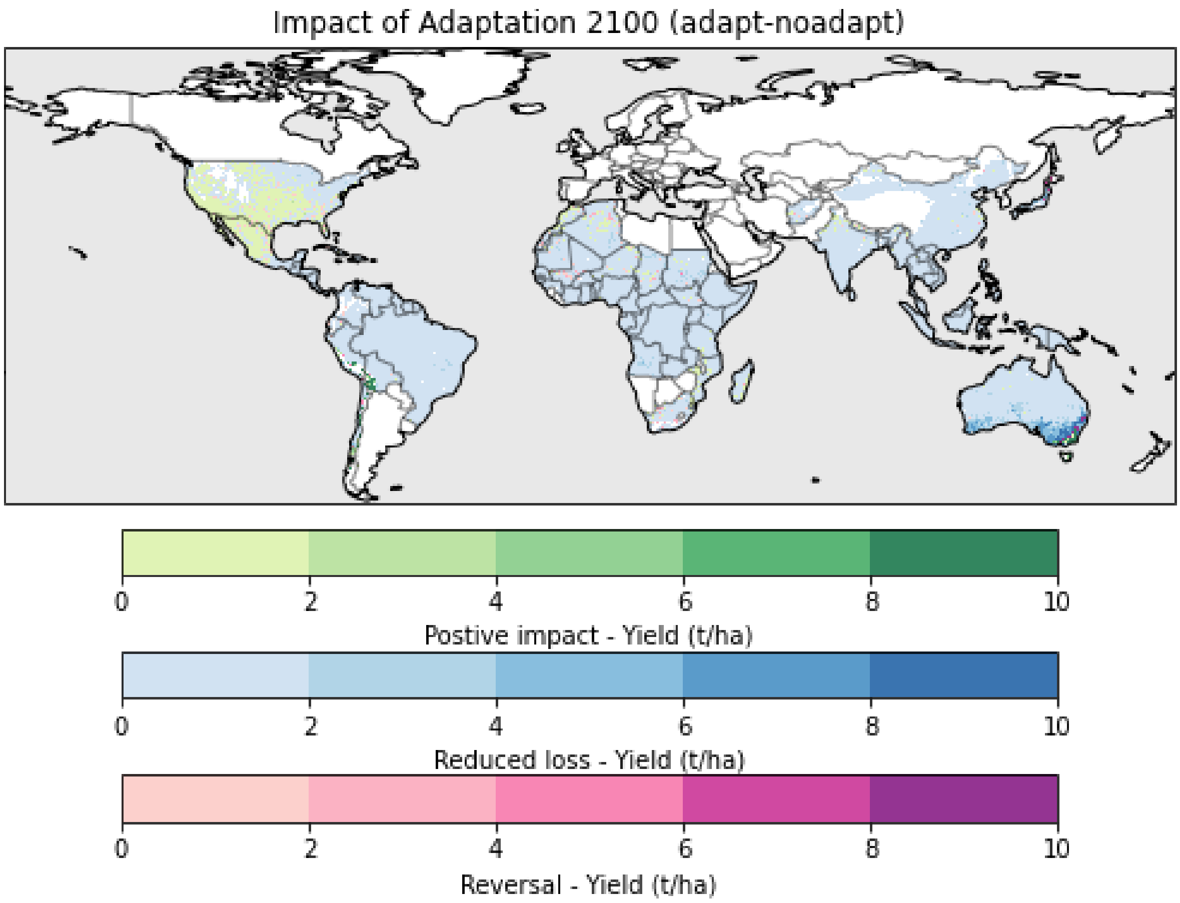

Figure S188: Global rainfed rice (season-2) reliability: Impact of adaptation on 2100 yields

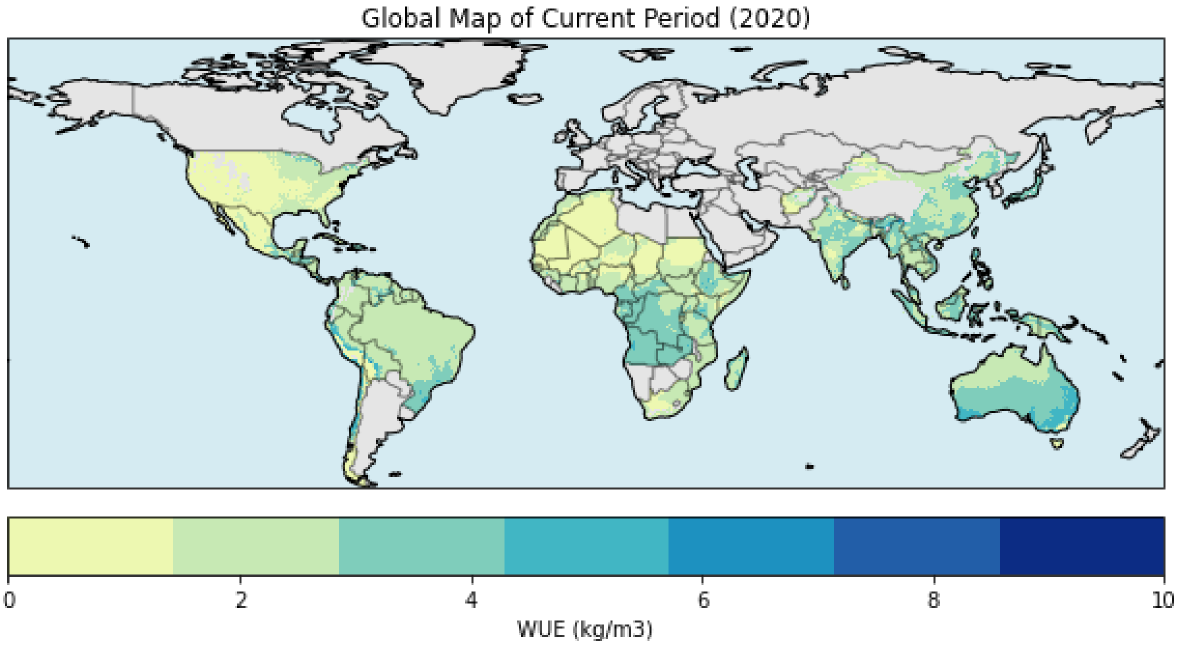

Figure S189: Global rainfed rice (season-2) water use efficiency: Baseline water use efficiency in 2020

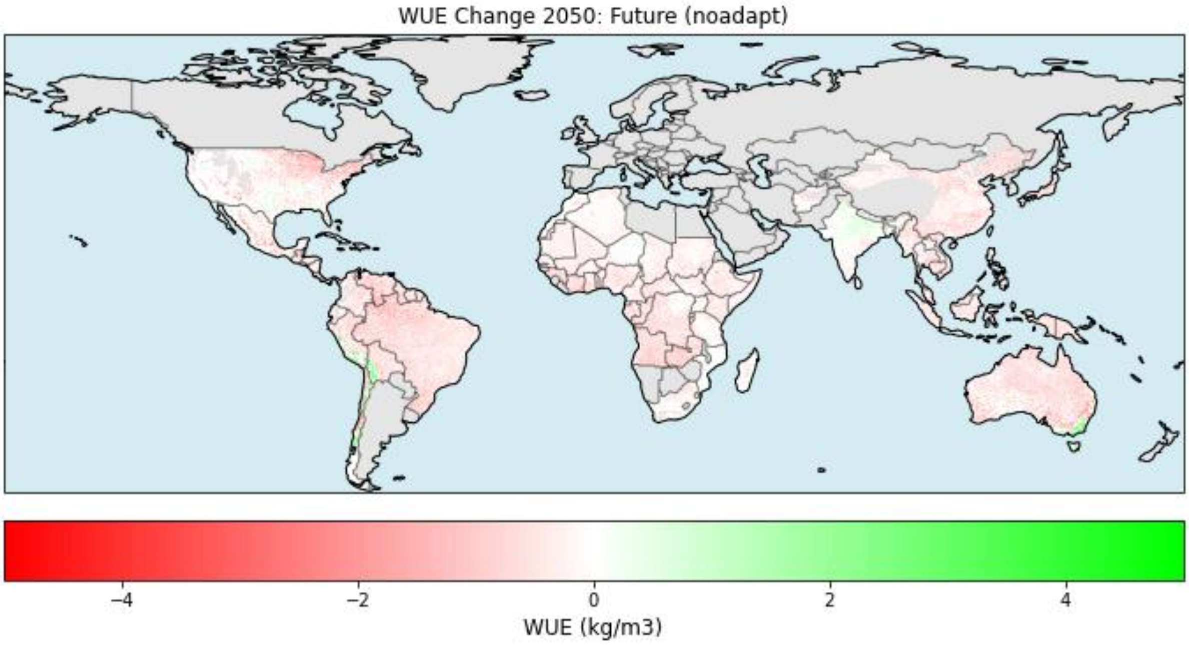

Figure S190: Global rainfed rice (season-2) water use efficiency: Projected water use efficiency change by 2050 without adaptation

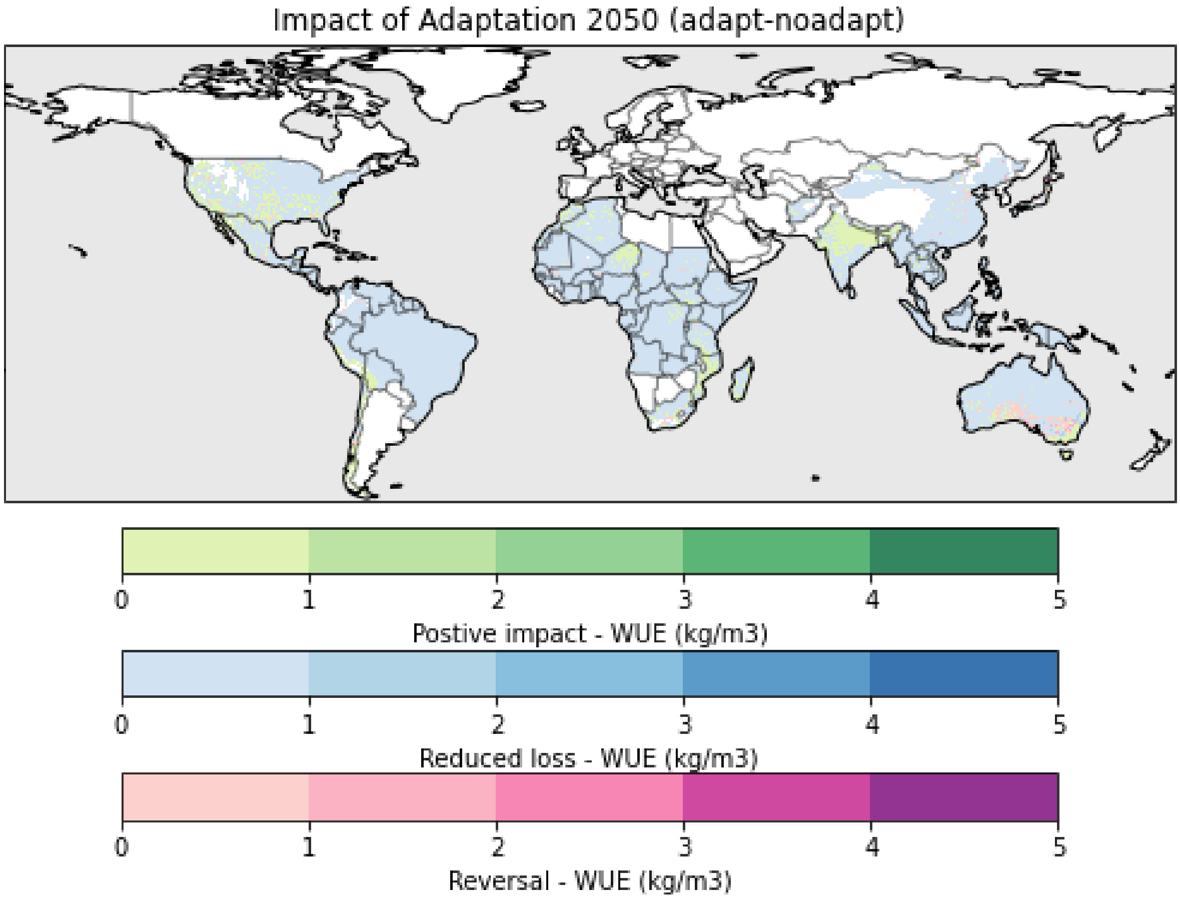

Figure S191: Global rainfed rice (season-2) water use efficiency: Impact of adaptation on 2050 water use efficiency

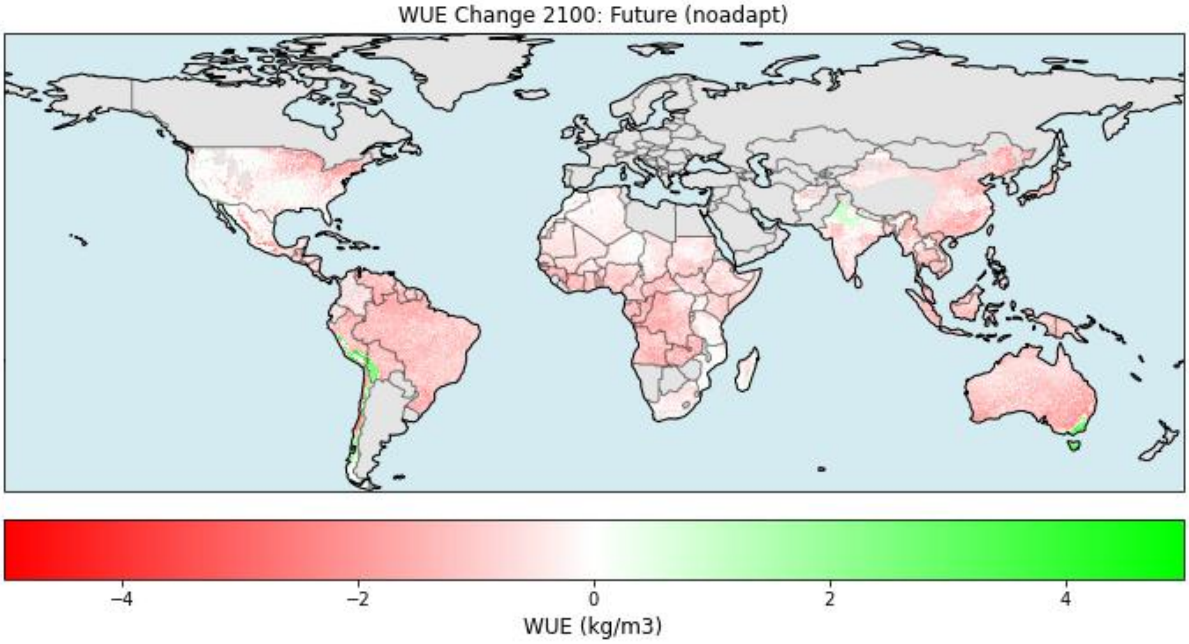

Figure S192: Global rainfed rice (season-2) water use efficiency: Projected water use efficiency change by 2100 without adaptation

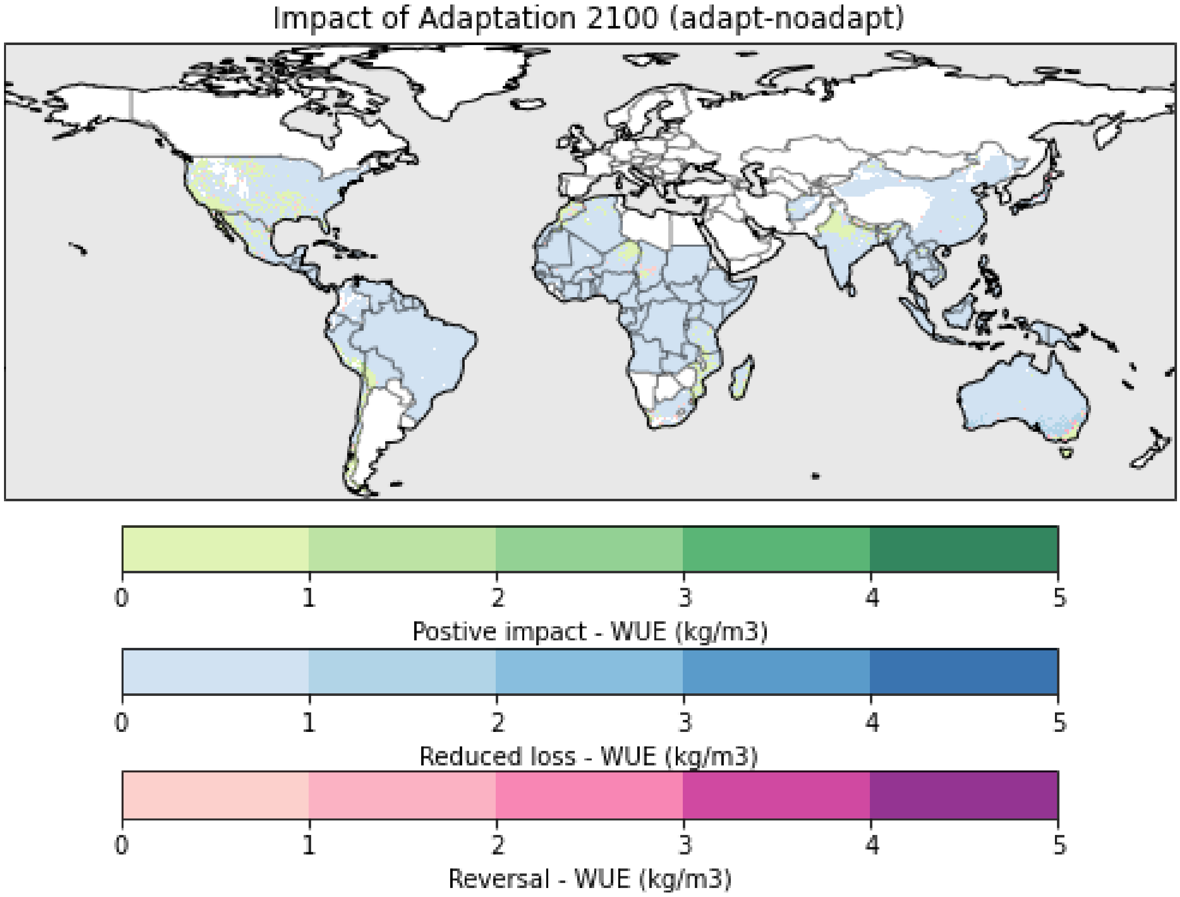

Figure S193: Global rainfed rice (season-2) water use efficiency: Impact of adaptation on 2100 water use efficiency
